# Unprecedented Antimicrobial and Cytotoxic Polyketides from Cultures of *Diaporthe africana* sp. nov.

**DOI:** 10.3390/jof9070781

**Published:** 2023-07-24

**Authors:** Blondelle Matio Kemkuignou, Christopher Lambert, Marc Stadler, Simeon Kouam Fogue, Yasmina Marin-Felix

**Affiliations:** 1 Department of Microbial Drugs, Helmholtz Centre for Infection Research (HZI) and German Centre for Infection Research (DZIF), Partner Site Hannover/Braunschweig, Inhoffenstrasse 7, 38124 Braunschweig, Germany; blondelle.matiokemkuignou@helmholtz-hzi.de (B.M.K.); christopher.lambert@helmholtz-hzi.de (C.L.); marc.stadler@helmholtz-hzi.de (M.S.); 2 Institute of Microbiology, Technische Universität Braunschweig, Spielmannstraße 7, 38106 Braunschweig, Germany; 3 Department of Cell Biology, Helmholtz Centre for Infection Research (HZI), Inhoffenstrasse 7, 38124 Braunschweig, Germany; 4 Department of Chemistry, Higher Teacher Training College, University of Yaoundé I, Yaoundé P.O. Box 47, Cameroon; kfogue@yahoo.com

**Keywords:** Diaporthaceae, Diaporthales, bioactivity, new taxon, secondary metabolites

## Abstract

Four unprecedented polyketides named isoprenylisobenzofuran B (**2**), isoprenylisobenzofuran C_1_/C_2_ (**3**), diaporisoindole F_1_/F_2_ (**4**), and isochromophilonol A_1_/A_2_ (**7**) were isolated from ethyl acetate extracts of the newly described endophytic fungus *Diaporthe africana*. Additionally, the previously reported cyclic depsipeptide eucalactam B (**1**) was also identified, along with the known compounds diaporisoindole A/B (**5**), tenellone B (**6**) and beauvericin (**8**). The taxonomic identification of the fungus was accomplished using a polyphasic approach combining multi-gene phylogenetic analysis and microscopic morphological characters. The structures **1**–**8** were determined by a detailed analysis of their spectral data, namely high-resolution electrospray ionization mass spectrometry (HR-ESIMS), 1D/2D nuclear magnetic resonance (NMR) spectroscopy, as well as electronic circular dichroism (ECD) spectra. In addition, chemical methods such as Marfey’s analysis were also employed to determine the stereochemistry in compound **1**. All the compounds obtained were evaluated for antimicrobial and in vitro cytotoxic properties. Compounds **3**–**8** were active against certain fungi and Gram-positive bacteria with MIC values of 8.3 to 66.6 µg/mL. In addition, **3**–**5** displayed cytotoxic effects (22.0 ≤ IC_50_ ≤ 59.2 µM) against KB3.1 and L929 cell lines, whereas compounds **6**–**8** inhibited the growth of seven mammalian cancer cell lines with IC_50_ ranging from 17.7 to 49.5 µM (**6**), 0.9 to 12.9 µM (**7**) and 1.9 to 4.3 µM (**8**).

## 1. Introduction

Fungi belonging to the genus *Diaporthe* (including its anamorph *Phomopsis*) have proven to be prolific sources of bioactive secondary metabolites with potential applications in pharmaceutical and agricultural fields [1,2]. In recent decades, more than 335 bioactive natural products have been discovered from *Diaporthe* spp. [1]. Polyketides plausibly constitute the largest and most structurally diverse class of secondary metabolites reported in this genus and account for ~64% of the hitherto isolated compounds in this fungal group [1]. These polyketides mainly include compounds belonging to the family of macrolides, azaphilones, benzofuranones, quinones, chromones, chromanones, xanthones, pyrones, phenols, and oblongolides, as classified by Xu et al. and Chepkirui et al. [1,2]. These substances have diverse biological properties, and the main bioactivities reported involve cytotoxic, antibacterial, and antifungal effects [1]. Given the emergence of multidrug resistance in many pathogens, drug-resistant cancer cells, and the emergence of life-threatening viral diseases, there is an urgent demand for the discovery of potential new pharmacological drugs [3]. Attributed to the widespread chemical and biological diversity of its secondary metabolites, the extensive exploitation of this fungal genus remains opportune for discovering new lead compounds that could possibly be developed as novel pharmaceutical drugs and/or biocontrol agents [4,5]. For instance, the compound emodin, when isolated from the endophytic fungus *Diaporthe lithocarpi*, has demonstrated remarkable cytotoxic activity against P-388 murine leukemia cells [6]. Additionally, isochromophilones A, G, and 5-chloroisorotiorin, derived from *Diaporthe perseae*, have shown potent antibacterial effects against various human pathogens, including the Gram-negative bacteria *Escherichia coli* and *Pseudomonas aeruginosa* [7]. Noteworthy are pestalotiopsones B and F produced by *Diaporthe* sp. SCSIO 41011, which have displayed significant antiviral activity against three subtypes of the influenza A virus [8]. These striking examples not only emphasize the potential of utilizing these compounds as valuable precursors for developing new derivatives with enhanced potency but also shed light on the vast potential of *Diaporthe*-derived secondary metabolites in the realm of drug discovery.

As part of our search for new bioactive secondary metabolites from selected endophytic fungal strains inhabiting Cameroonian medicinal plants, numerous metabolites have been isolated from the culture of *Diaporthe breyniae* [9], some of which displayed antimicrobial and cytotoxic effects. In our continuous endeavors, a new species of *Diaporthe* was recently discovered, and the investigation of its ethyl acetate (EtOAc) extracts showed moderate antimicrobial activities as well as interesting HPLC-DAD/MS (high-performance liquid chromatography coupled with diode array detection and mass spectrometry) chromatograms, possibly containing unknown compounds. These results prompted an in-depth investigation of this fungus, and we herein report upon the taxonomic identification as well as the isolation, structural elucidation, and biological properties of its secondary metabolites.

## 2. Materials and Methods

### 2.1. General Experimental Procedures

Optical rotations were measured in methanol (Uvasol, Merck, Darmstadt, Germany) using an Anton Paar MCP-150 polarimeter (sodium D line, a Nickel alloy sample cell 100 mm/3 mm, 0.7 mL volume, Seelze, Germany) at 20 °C. UV-Vis spectra were acquired using methanol (Uvasol, Merck, Darmstadt, Germany) with a Shimadzu UV-Vis 2450 spectrophotometer (Kyoto, Japan). ECD spectra were recorded on a J-815 spectropolarimeter (JASCO, Pfungstadt, Germany) using a 0.5 mm quartz cuvette and MeOH as the solvent. Nuclear magnetic resonance (NMR) spectra were recorded at a temperature of 298 K with an Avance III 500 spectrometer (Bruker, Billerica, MA, USA, ^1^H-NMR: 500 MHz and ^13^C-NMR: 125 MHz) and an Ascend 700 spectrometer equipped with 5 mm TCI cryoprobe (Bruker, Billerica, MA, USA, ^1^H-NMR: 700 MHz and ^13^C-NMR: 175 MHz). Chemical shifts were given in parts per million (ppm) and coupling constants in hertz (Hz). NMR spectra were referenced to residual solvent signals with resonances at δ_H_/_C_ 2.50/39.5 for DMSO and at δ_H_/_C_ 7.26/77.2 for CDCl_3_. Electrospray ionization mass spectra (ESI-MS) were acquired with an UltiMate 3000 Series uHPLC (Thermo Fischer Scientific, Waltman, MA, USA) equipped with a C18 Acquity UPLC BEH column (2.1 × 50 mm, 1.7 µm; Waters, Milford, CT, USA) connected to an amaZon speed ESI-Iontrap-MS (Bruker, Billerica, MA, USA). The HPLC parameters were set as follows: solvent A: MilliQ H_2_O + 0.1% formic acid, solvent B: acetonitrile (ACN) + 0.1% formic acid, gradient: 5% B for 0.5 min increasing to 100% B in 19.5 min, then an isocratic condition at 100% B for 5 min with a flow rate of 0.6 mL/min, and Diode-Array Detection (DAD) at 210 nm and 190–600 nm. High-resolution electrospray ionization mass spectra (HR-ESIMS) were recorded with an Agilent 1200 Infinity Series HPLC–UV system (Agilent Technologies, Santa Clara, CA, USA) connected to a MaXis ESI-TOF mass spectrometer (scan range 100–2500 *m*/*z*, capillary voltage 4500 V, dry temperature 200 °C). The column and HPLC parameters remained consistent with those used for ESI-MS, and the DAD was set in the range of 200–640 nm.

### 2.2. Fungal Isolation and Identification

Two strains of endophytic fungi were isolated from the bark of the terrestrial plants *Asystasia macrophylla* and *Polyscias fulva*, which were collected in Kala Mountain (Center region in Cameroon) and Tonga (West Cameroon), respectively, following the protocol previously described [9].

Hyphal material (1 mm diam) was scratched of actively growing cultures on a YM 6.3 agar (malt extract 10 g/L, yeast extract 4 g/L, D-glucose 4 g/L, agar 20 g/L, pH 6.3 before autoclaving) and was transferred onto a 9-cm-diam Petri dished containing 2% tap water agar supplemented with sterile pine needles (PNA) [10], potato dextrose agar (PDA), oatmeal agar (OA) and malt extract agar (MEA) [11]. The plates were incubated at 21 °C in darkness. Pigment production, colony diameters, and colony characters on PDA, OA, and MEA were noted after 15 d. Colony colors were described using the color chart of the Royal Horticultural Society London (1966) [12]. An examination of morphological characters was conducted by mounting fungal structures in clear lactic acid, and 30 measurements of each structure were conducted. Photomicrographs were taken using an eclipse Ni compound microscope, using a DS-Fi3 (Nikon, Tokyo, Japan) and NIS-Elements imaging software v. 5.20, and a Keyence VHX-970F microscope (Neu-Isenburg, Germany).

The sequences of five loci in total (ITS, *cal*, *his3*, *tef1*, *tub2*; see [9] for details) were generated and subsequently checked for their phylogenetic placement first in a broad phylogenetic study following the maximum Likelihood criterion implemented in IQTree V2.1.3 [13], assessing statistical support by bootstrapping [14]. In total, 370 taxa were used (retrieved from GenBank, Supplementary Material Table S5) for the gblocks [15] curated MAFFT [16] (as implemented in Geneious 7.1.9) alignment using distinct nucleotide substitution models [17,18]. After the evaluation of the phylogenetic relationship of strain CBS 150080, the data matrix was restricted to a well-supported clade, including 66 *Diaporthe* spp. (Table 1). The original full-length sequences were aligned using MAFFT, and misalignments were corrected manually. Trees were inferred from both the Maximum-Likelihood criterion using IQTree and Bayesian methodology (parallelized version of MrBayes 3.2.7a [19,20]; nucleotide model testing with PartitionFinder2 as implemented in Phylosuite V1.2.2 [21,22]), checked for congruence and support values over 70% (bootstrap, bs) and 0.95 (posterior probability, pp) mapped onto the ML tree. The sequences generated in this study were deposited in GenBank (Table 1). The alignments used in the phylogenetic analysis are included in the Supplementary Material.

The herbarium-type material and the ex-type strain of the new species were maintained at the collection of the Westerdijk Fungal Biodiversity Institute (CBS), Utrecht, The Netherlands.

### 2.3. Fermentation and Extraction

*Diaporthe africana* was cultivated in both, liquid and solid media. The liquid fermentation was conducted in a yeast malt glucose medium (YMG, 10 g malt extract, 4 g yeast extract, 4 g D-glucose in 1 L of deionized water with pH adjusted to 6.3 before sterilization). In detail, five plugs (obtained with a 7 mm cork borer) of the mycelial culture grown on YMG agar (composition as reported above, with 20 g/L of agar and without adjusting the pH) plates were used to inoculate 200 mL of liquid YMG medium contained in 5 × 500 mL Erlenmeyer flasks. The flasks were incubated at 23 °C under shaking conditions at 140 rpm on a rotary shaker. The growth of the fungus was monitored by checking the amount of free glucose daily using Medi-Test glucose strips (Macherey-Nagel, Düren, Germany). On the 6th day, the glucose was completely depleted, and fermentation was aborted 3 days afterward. The fungal mycelia were harvested by filtration, and the resulting supernatant was extracted three times with an equal amount of EtOAc using a separating funnel. After filtration over anhydrous sodium sulfate (Na_2_SO_4_), the ethyl acetate solution was evaporated to dryness in vacuo (40 °C) to afford 28 mg of the extract. The wet mycelia or biomass was extracted thrice with acetone in an ultrasonic bath (Sonorex Digital 10 P, Bandelin Electronic GmbH and Co. KG, Berlin, Germany) at 40 °C for 30 min. The obtained solvent was concentrated in vacuo using a rotary evaporator to yield an aqueous phase, which was extracted three times with the same amount of ethyl acetate. The ethyl acetate fraction was then filtered over anhydrous Na_2_SO_4_ and was evaporated to dryness to afford 198 mg of the mycelial extract.

The fungus was also cultivated in 10 × 500 mL flasks containing a solid autoclaved rice-based medium (BRFT: brown rice 28 g with 0.1 L base liquid (yeast extract 1 g/L, sodium tartrate 0.5 g/L, KH_2_PO_4_ 0.5 g/L) per flask) [55]. Each flask was inoculated with 10 mL of the seed culture, which was prepared beforehand. To obtain the seed culture, three small pieces of mycelial culture grown on a YMG agar were used to inoculate a 250 mL Erlenmeyer flask containing 100 mL of the liquid YMG medium. The seed culture was incubated for 5 days at 23 °C under shaking conditions at 140 rpm, and was subsequently used to inoculate the flasks containing the solid rice medium. The flasks were incubated under static conditions at 23 °C for 28 days. The fermented cultures were extracted following the same previously described methodology as for the mycelia obtained from the liquid culture in YMG, to afford 3 g of orange-colored extract.

### 2.4. Isolation of Compounds ***1**–**8***

The EtOAc extracts obtained from liquid and solid fermentation were fractionated separately, as they contained different secondary metabolites. Following analytical HPLC-DAD profiling, the supernatant extract (28 mg) dissolved in methanol was purified using the preparative reverse phase HPLC (Büchi, Pure C-850, 2020, Flawil, Switzerland) with the following elution gradient: 20−45% solvent B in 20 min, 45−65% B in 30 min, 65−100% B in 10 min and thereafter isocratic conditions at 100% B for 10 min. A VP 250/21 Nucleodur C18 Htec 10 µm column (Macherey-Nagel) was used as a stationary phase with a flow rate of 15 mL/min. The mobile phase was constituted of two solvents, A (MilliQ H_2_O + 0.1% formic acid (FA)) and B (acetonitrile (ACN) + 0.1% FA). UV detection was set up to 210 and 350 nm. Four fractions (F1−F4) were collected according to the observed peaks, and their purity was examined using HPLC-DAD-MS. This resulted in the acquisition of two compounds: **3** (1.2 mg, *t_R_* = 40 min) and **6** (3.2 mg, *t_R_* = 57.9 min) from F2 to F4, respectively. The mycelial extract (198 mg) obtained from the liquid fermentation was discarded as it did not contain any metabolites of interest.

A portion of the extract (1 g) obtained from the solid rice medium was dissolved in a sufficient amount of methanol (100 mL) and was filtered using an SPME Strata™-X 33 u Polymeric RP cartridge (Phenomenex, Inc., Aschaffenburg, Germany). The resulting solution was evaporated in vacuo and re-dissolved in an adequate amount of methanol prior to purification using preparative RP-HPLC (VP 250/40 Nucleodur C18 Htec 10 µm column, flow rate of 30 mL/min, elution gradient from 5 to 80% solvent B in 70 min, 80−100% B in 20 min, and finally isocratic conditions at 100% B for 5 min). Three runs were performed on the RP-HPLC, and the obtained fractions (F1−F10) were combined according to UV absorption at 210 and 350 nm and concurrent HPLC-MS analysis. The fractionation yielded three compounds: **4** (1.5 mg, *t_R_* = 36.2 min), **5** (1.1 mg, *t_R_* = 45.4 min), and **8** (1.6 mg, *t_R_* = 86.5 min). F3 (11 mg), F8 (28 mg), and F9 (20 mg) were further purified using preparative RP-HPLC with an XBridge^TM^ Trifunctional C18, 250 × 19 mm, 135 Å, 5 μm column (Waters, Eschborn, Germany) at a flow rate of 15 mL/min. F3 (elution gradient: 5–10% solvent B in 10 min, then in an isocratic condition at 50% B for 30 min and finally 50–100% B in 10 min) yielded compound **1** (9.7 mg, *t_R_* = 16 min), whereas F8 (elution gradient: 5–60% solvent B in 10 min, then in an isocratic condition at 60% B for 30 min and finally 60–100% B in 5 min) and F9 (elution gradient: 5−65% solvent B in 10 min, followed by an isocratic condition at 65% B for 30 min and finally 65–100% B in 10 min) afforded compounds **2** (11.9 mg, *t_R_* = 17.6 min) and **7** (10 mg, *t_R_* = 27.5 min), respectively. A flow chart of the purification procedure can be found in the Supplementary Materials (Figure S76).

### 2.5. Determination of Amino Acid Stereochemistry

The stereochemistry of threonine in compound **1** was determined using Marfey’s analysis following the protocol described by Harms et al. [56] with slight modification. In detail, compound **1** (1 mg) was hydrolyzed in 6 N HCl (1 mL) at 90 °C for 18 h. The hydrolysate was evaporated to dryness using a speedVac vacuum connected to a chemistry hybrid pump (Wertheim, Germany), which was then dissolved in 400 μL Milli-Q H_2_O and divided into two individual vials. 1 M NaHCO_3_ (20 μL) and 1% N-(2,4-dinitro-5-fluorophenyl)-L-valinamide (_L_-FDVA, 100 μL in acetone) were added into one vial, and the other vial was supplemented with the same amount of 1 M NaHCO_3_ and 1% _D_-FDVA. Concurrently, 2 mg of the authentic amino acids: _L_-threonine, _D/L_-threonine, and _L_-*allo*-threonine (Sigma-Aldrich, Deisenhofen, Germany) were used as standards and were treated similarly to the hydrolysate of **1**. The mixtures were incubated at 40 °C for 40 min. After cooling to room temperature, the solutions were neutralized with 2 N HCl (20 μL) and evaporated to dryness using the speedVac vacuum. Afterward, the residues were dissolved in 1 mL MeOH and analyzed using uHPLC connected to an amaZon speed ESI-Iontrap mass spectrometer (column and conditions were described in General Experimental Procedures). The stereochemistry of threonine in **1** was finally deduced as _L-_Thr by comparison with the retention times of the _L_-and _D_-FDVA-derivatized hydrolysate of **1** (_L_-Thr-_L_-FDVA *t_R_* 6.17 min; L-Thr-_D_-FDVA *t_R_* 7.30 min) with that of Marfey’s derivatized authentic amino acids (Tables S3 and S4 and Figures S72 and S73).

### 2.6. Antimicrobial and Cytotoxic Activities

The Minimum Inhibitory Concentration (MIC) of the isolated metabolites (**1**–**8**) was evaluated using serial dilution assays in a 96-well microtiter plate against a panel of Gram-positive bacteria, namely *Bacillus subtilis* DSM 10, *Mycobacterium smegmatis* ATCC 700084 and *Staphylococcus aureus* DSM 346; Gram-negative bacteria, including *Acinetobacter baumannii* DSM 30008, *Chromobacterium violaceum* DSM 30191, *Escherichia coli* DSM 1116 and *Pseudomonas aeruginosa* PA14 and fungal cultures of *Candida albicans* DSM 1665, *Mucor hiemalis* DSM 2656, *Rhodotorula glutinis* DSM 10134, *Schizosaccharomyces pombe* DSM 70572 and *Wickerhamomyces anomalus* DSM 6766, according to previously described protocols [57]. The MTT-based cytotoxicity assay was performed against several cancer cell lines (human endocervical adenocarcinoma KB 3.1, mouse fibroblasts L929, squamous cancer A431, breast cancer MCF-7, lung cancer A549, ovary cancer SK-OV-3, and prostate cancer PC-3) in accordance with our previously established protocols [57,58].

## 3. Results and Discussion

### 3.1. Molecular Phylogeny and Taxonomy

**Figure 1:**
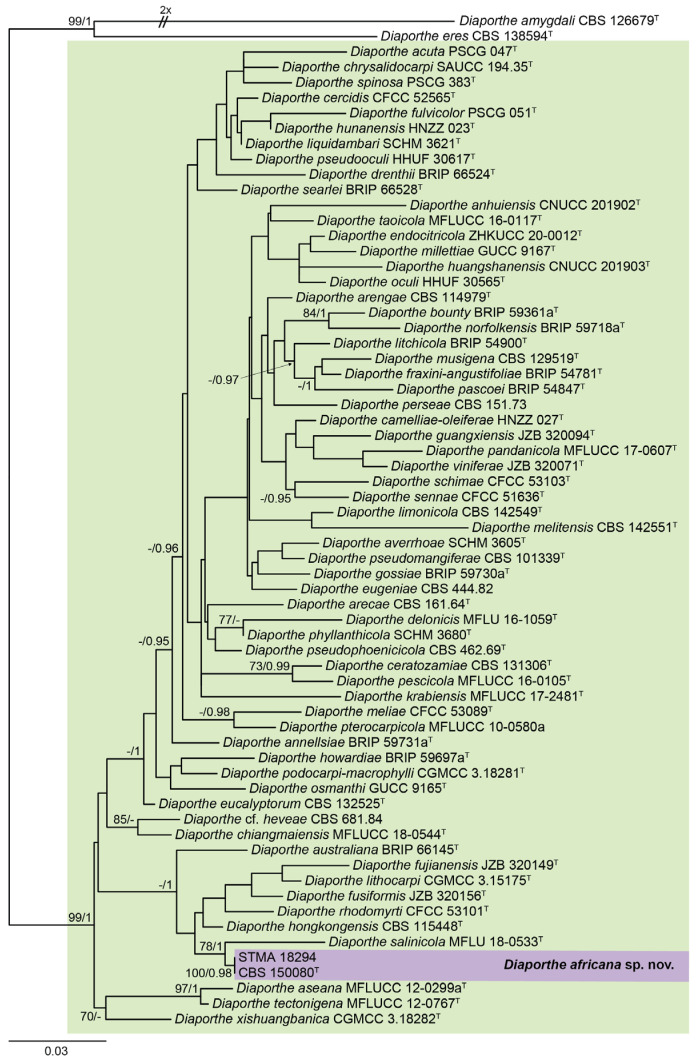
ML phylogram obtained from a combined ITS, *cal*, *his3*, *tef1,* and *tub2* dataset, including sequences of our strain and a selection of related *Diaporthe* spp., restricted according to the phylogenetic study of Supplementary Material (Figure S70). *Diaporthe amygdali* CBS 126679^T^ and *D. eres* CBS 138594^T^ were used as outgroup. Bootstrap support values ≥ 70/Bayesian posterior probability scores ≥ 0.95 are indicated along the branches. Branch lengths are proportional to the distance. The new taxon is indicated in bold. Sequences derived from the type material of the different species are indicated with ^T^.

The generated sequences were checked for their phylogenetic placement first in a broad phylogenetic inference (see Supplementary Material for more details). Subsequently, we restricted the phylogenetic inference to a well-supported clade comprising 66 taxa for a more detailed study to save computing time. This new five-loci dataset was manually curated and yielded a data matrix that was used for the phylogenetic study comprising in total of 2449 (ITS: 554; *cal*: 423; *his3*: 359; *tef1*: 392; *tub2*: 721) sites. Both trees inferred by the Maximum-Likelihood criterion (l Ln = −14,653.6590) using IQTree and Bayesian methodology revealed a congruent tree (Figure 1). Here, the characterized strains formed a well-supported clade (100% bs/0.98 pp), and clustered with *D. salinicola* (78. bs/1 pp). The position of the newly formed clade consisting of *D. salinicola* and *D. africana* was not resolved. This was in agreement with single locus trees that were calculated for comparison.

**Figure 2:**
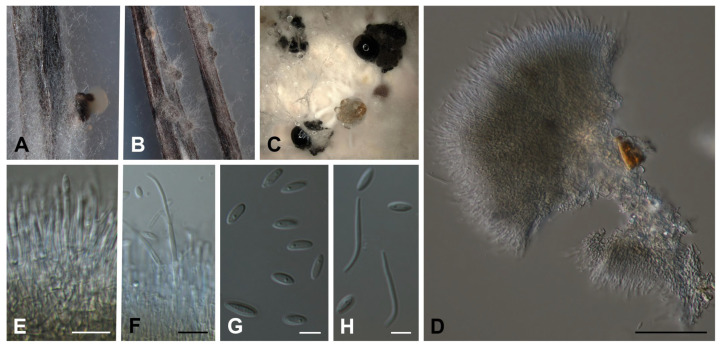
*Diaporthe africana* CBS 150080^T^. (**A**,**B**) Conidiomata in PNA. (**C**) Conidiomata in OA. (**D**–**F**) Conidiophores, conidiogenous cells and conidia. (**G**) Alpha conidia. (**H**) Alpha and beta conidia. Scale bars: (**D**) = 50 µm; (**E**,**F**) = 10 µm; (**G**,**H**) = 5 µm.

***Diaporthe africana*** Y. Marín & C. Lamb., **sp. nov.** MycoBank MB849047. (Figure 2A–H).

*Etymology*: Refers to the continent from which the fungus has been isolated.

*Type material*: CAMEROON: Tonga, from the bark of *Polyscias fulva*, April 2019, S.F. Kouam (holotype CBS H-25264; ex-type cultures CBS 150080 = STMA 18293).

*Additional material*: CAMEROON: Kala Mountain, from the bark of *Asystasia macrophylla*, 3 January 2019, S.F. Kouam, A.M. Elodie Gisele and S.C.N. Wouamba, STMA 18294.

Conidiomata pycnidial in culture on PNA, globose or irregular, dark brown to black, solitary or in groups, embedded, erumpent, 190–390(–430) μm diam, white to yellow conidial drops exuded from ostioles; conidiomatal wall brown to dark brown, composed of 1–2 layers, *textura angularis* to *epidermoidea*. Conidiophores cylindrical to subcylindrical, base subhyaline to pale yellow, apex hyaline to subhyaline, straight, densely aggregated, smooth-walled, 1–2 septate, (12–)16–26(–28) × 1.5–3 μm. Conidiogenous cells phialidic, cylindrical, tapering towards the apex, hyaline to subhyaline, terminal, 7.5–16(–18) × 1.5–2.5 μm. Paraphyses not observed. Alpha conidia ovoid to ellipsoidal, hyaline, apex rounded, base acutate, multiguttulate, aseptate, (5.5–)6–7.5(–8) × 2–2.5 μm. Beta conidia filiform, straight to curved, apex rounded, tapering towards base, hyaline, not guttulate, aseptate, (10–)13.5–21(–22.5) × 1–2 μm. Gamma conidia not observed.

Culture characters: Colonies on PDA covering a 9 cm Petri dish in 2 weeks, white with margins transparent to pale yellow (11D), cottony, raised, margins fringed; reverse yellow (161B–D). Colonies on MEA reaching 85–90 in 2 weeks, white to greyed white (156A–D), cottony, crateriform, margins fringed; reverse greyed yellow (162B–D) with center and a ting surrounding it grey brown (199A–D) to black (202A–B). Colonies on OA covering all Petri dish in 2 weeks, white to yellow-white (158B–C) with center grey (201A), cottony, slightly crateriform, margins fringed; reverse transparent to greyed green (198C–D).

Notes: *Diaporthe africana* formed a well-supported basal clade (100% bs/0.98 pp) together with *D. salinicola*. For this latter species, only the sexual morph has been reported up to now. Our new species did not form the sexual morph in any of the media tested. Moreover, *D. salinicola* has been only reported from Thailand, Asia [42].

*Diaporthe africana* is also phylogenetically related to *D. hongkongensis*. Both species were so far characterized to form alpha and beta conidia. However, *D. hongkongensis* could be easily distinguished by the production of gamma conidia [24], which were not observed in *D. africana*. *Diaporthe hongkongensis* has been reported only from Asia, including China, Japan, Taiwan, and Turkey [24,59,60,61,62].

### 3.2. Structural Elucidation of Compounds ***1**–**8***

**Figure 3:**
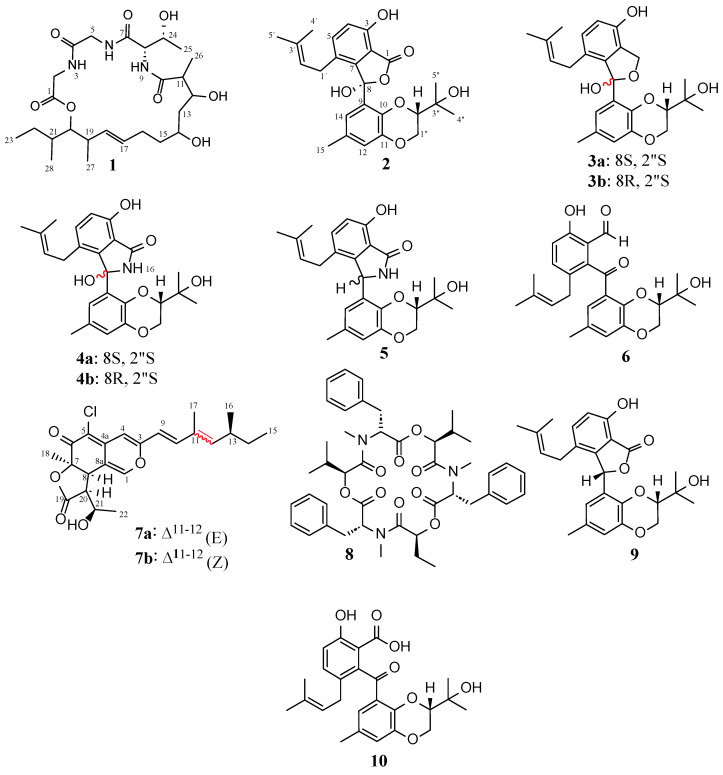
Chemical structures of compounds **1**–**8** isolated from *Diaporthe africana* and the known compounds isoprenylisobenzofuran A (**9**) and tenellone C (**10**).

Purification of the EtOAc extracts of *D. africana* obtained from cultures in liquid YMG and solid rice media yielded 8 compounds, including two cyclic depsipeptides (**1**, **8**), five tenellone derivatives (**2**–**6**), and one azaphilone (**7**) (Figure 3). The known compounds **5**, **6,** and **8** were identified as the mixture of diaporisoindoles A and B [63,64], tenellone B [65], and beauvericin [66,67], respectively. This identification was achieved through an analysis of their spectral data (ESI-MS, HR-ESIMS, 1D/2D NMR, Figures S54–S71) and specific rotation as well as a comparison with previously published data. Interestingly, this is the first NMR-confirmed report of the occurrence of beauvericin (**8**) in endophytes belonging to the genus *Diaporthe*. This cyclic hexadepsipeptide (**8**) was initially obtained from the entomopathogenic fungus *Beauveria bassiana* [66] but was also found to be produced by many species belonging to the genus *Fusarium* and some *Isaria* spp. [68].

Compounds **2**–**4**, **7** were isolated for the first time in the present study, and their structures were established after an exhaustive examination of their HR-ESIMS, NMR, and ECD spectra. It should be mentioned that although compound **1** had been previously characterized, the data regarding its structure elucidation have been included in this manuscript as well.

Compound **1** was obtained as a colorless oil. The molecular formula of C_25_H_43_N_3_O_8_ (6 degrees of unsaturation) was established based on (+) HR-ESIMS *m*/*z* 514.3124 [M + H]^+^ (calcd for 514.3123 C_25_H_44_N_3_O_8_^+^). An analysis of the ^1^H-NMR and ^1^H-^13^C HSQC spectra recorded in DMSO-d_6_ of **1** revealed clear signals corresponding to five methyl groups resonating at δ_H_ 0.83 (H_3_-28), δ_H_ 0.88 (H_3_-27), δ_H_ 1.20 (H_3_-26), δ_H_ 1.02 (H_3_-25), and δ_H_ 0.84 (H_3_-23). On the same spectrum, signals attributable to four methylene groups (δ_H_ 1.65, H_2_-13; δ_H_ 1.38, H_a_-15; δ_H_ 1.48, H_b_-15; δ_H_ 1.88, H_a_-16; δ_H_ 1.99, H_b_-16; δ_H_ 1.15, H-22), two N-bearing methylenes (δ_H_ 3.94, H_a_-2; δ_H_ 3.69, H_b_-2; δ_H_ 3.77, H_2_-5), three methines (δ_H_ 2.36, H-11; δ_H_ 2.32, H-19; δ_H_ 1.62, H-21), one N-bearing methine (δ_H_ 3.98, H-8), four O-bearing methines (δ_H_ 3.83, H-12; δ_H_ 3.57, H-14; δ_H_ 4.67, H-20, δ_H_ 4.18, H-24), two olefinic protons (δ_H_ 5.45, H-17; δ_H_ 5.09, H-18) and three amide hydrogens (δ_H_ 8.36, NH-3; δ_H_ 7.43, NH-6; δ_H_ 7.85, NH-9,) were observed. The signal-to-signal comparison of ^13^C NMR with ^1^H-^13^C HSQC spectrum of **1** confirmed 25 carbon signals which were classified into four carbonyl groups (δ_C_ 169.3, C-1; 168.6, C-4; 170.4, C-7 and 175.3, C-10), included two olefinic carbons (δ_C_ 130.7, C-17; δc 131.6, C-18), eight methines (including one N-bearing and four O-bearing ones), six methylenes (including two N-bearing ones) and five methyl groups (Table 2).

**Figure 4:**
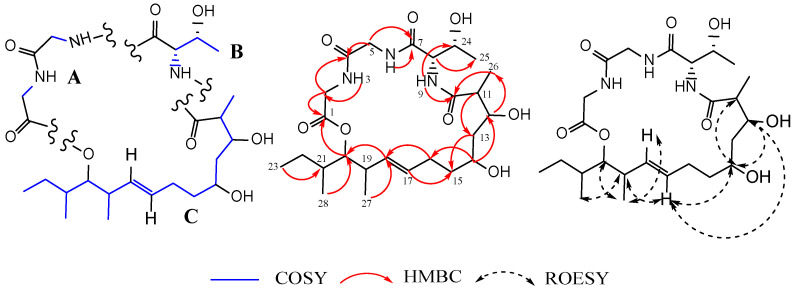
Fragments A−C**:** key ^1^H-^1^H COSY, ^1^H-^13^C HMBC and ^1^H-^1^H ROESY correlations of compound **1**.

The careful interpretation of 2D NMR data and, more importantly, of the ^1^H-^1^H COSY and ^1^H-^13^C HMBC spectra enabled the construction of the gross structure of **1**. On the COSY and HMBC spectra, three fragments (A–C) were unambiguously determined based on the observed correlations, as depicted in Figure 4. The HMBC correlations of NH-3 to C-2/C-4, NH-6 to C-4/C-5, and H_2_-2 to C-1/C-4 suggested that fragment A comprised two glycine residues. Similarly, a second amino acid residue (fragment B) was deduced as threonine based on key HMBC correlations of the amide proton NH-9 with C-8/C-24, alongside H-8 with carbonyl C-7, oxymethine C-24 and methyl C-25 (Figure 4). The downfield shifted chemical value of C-11, which resonated at δ_C_ 45.1, suggested its connection to a carbonyl group. This was further supported by a strong HMBC correlation between H-11 and the carbonyl C-10. Furthermore, the HMBC cross peaks of H-11, H-12, and H-26 to C-10 enabled the incorporation of the carbonyl group C-10 into the fatty acid residue (fragment C). On the HMBC spectrum, the key correlations to determine the linkage of moieties A and B were those of NH-6 and H-5 with C-7. Correlations of H-8 and NH-9 with C-10 validated the linkage of moieties B and C. The presence of four carbonyl groups and one olefinic bond, which accounted for five out of the six indices of hydrogen deficiency, as depicted by the molecular formula, suggested the existence of a ring in compound **1**. The strong HMBC correlation observed between the oxymethine proton H-20 and the carbonyl C-1 corroborated the connection of moieties C and A, thus leading to the formation of a 21-membered macrocyclic core ring. Additionally, we investigated the absolute stereochemistry of fragment B. By using Marfey’s method (as described in the experimental section), the L-configuration (8*S*,24*R*) was assigned to the threonine residue by a comparison of the retention times of the _L_-and _D_-FDVA-derivatized hydrolysate of **1** (_L_-Thr-_L_-FDVA *t_R_* 6.17 min; L-Thr-_D_-FDVA *t_R_* 7.30 min) with that of Marfey’s derivatized authentic amino acids (Tables S3 and S4). In addition, the large coupling constant (*J* = 15.4 Hz) indicated an *E*-configuration for the ∆^17–18^ double bond. This was also confirmed by the absence of the ROESY correlation between protons H-17 and H-18, providing further evidence for their *trans*-relationship. It is worth mentioning that despite some important correlations observed on the ROESY spectrum of **1**, as depicted in Figure 4, the determination of the relative stereochemistry at positions C-11, C-12, C-14, C-19, C-20, and C-21 remained inconclusive. This could be attributed to the inherent flexibility of the 21-membered macrolide ring, which could adopt various conformations and orientations, making it challenging to establish definitive stereochemical relationships at these positions. Based on the available evidence, the structure of compound **1** was successfully deduced, although its stereochemistry could only be partially determined. Curiously, while the current study has been under review, we became aware of a publication by Gao et al. [69], who reported a similar planar structure. Subsequently, a signals-to-signals comparison of the ^1^H NMR spectrum of **1** (recorded in CDCl_3_, Figure S9) with that of eucalactam B isolated from *Diaporthe eucalyptorum* showed a perfect match of chemical shifts (Table S1), suggesting that compound **1** was identical to eucalactam B [69]. However, while the relative configuration at positions C-12 and C-14 in eucalactam B were determined through a semisynthetic approach, revealing a *syn* stereochemistry [69], the absolute configuration of molecule **1** remains unresolved and requires further investigation. Previous attempts to employ X-ray crystallography and crystalline sponge methods to establish the absolute stereochemistry of this compound have proven unsuccessful, as reported by Gao et al. [69]. Therefore, other alternative methodologies must be explored, including chemical synthesis or computational approaches such as molecular modeling and density functional theory (DFT) calculations.

**Figure 5:**
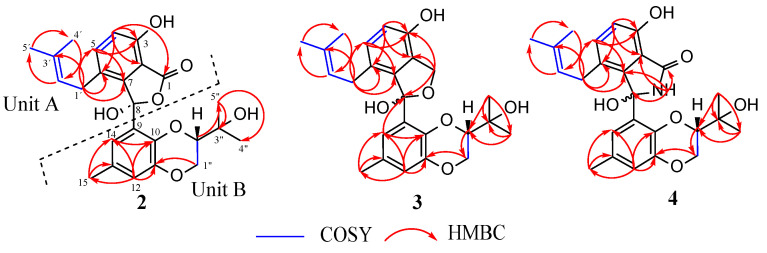
Key ^1^H-^1^H COSY and ^1^H-^13^C HMBC correlations of compounds **2**–**4**.

Compound **2** was obtained as pale yellow oil. Its molecular formula was assigned as C_25_H_28_O_7_ based on (+) HR-ESIMS *m*/*z* 441.1905 [M + H]^+^ (calcd for 441.1908 C_25_H_29_O_7_^+^), indicating 12 degrees of unsaturation. An analysis of its 1D (Table 3) and 2D NMR spectroscopic data revealed similar features to those of the known isoprenylisobenzofuran A (**9**) [70]. In comparison to **9** (molecular formula: C_25_H_28_O_6_), the HR-ESIMS of compound **2** showed an increase in 16 Da, and the obtained molecular formula for **2** suggested that a hydrogen atom in **9** was possibly replaced by a hydroxyl group. The ^1^H NMR data of **2** recorded in CDCl_3_ showed broad signals, which led to the absence of important correlations in the ^1^H-^13^C HMBC spectrum. To solve this issue, a new set of NMR data were recorded in CDCl_3_ + 1%TFA, which facilitated the acquisition of well-defined ^1^H signals. The ^1^H−^1^H COSY and ^1^H-^13^C HMBC spectra supported the existence of these fragments: 1,2,3,4-tetrasubstituted benzene ring, isoprenyl, and a 2,3-dihydrobenzo[*b*][1, 4]dioxane moiety (Figure 5). The characteristics of HMBC correlations observed in compound **2** exhibited striking similarities to those reported for compound **9**. However, the absence of the key signal C-8 in the ^13^C NMR spectrum, as well as the absence of the important HMBC correlation of H-14 to C-8 (to confirm the linkage point of units A and B), made establishing the structure of **2** ambiguous. In light of these observations, it was also conceivable to propose the structure of the previously known compound tenellone C (**10**) for compound **2**. To further investigate this possibility, the NMR spectrum of compound **2** was recorded in deuterated methanol (MeOH-d_4_) for a more comprehensive comparison with tenellone C, which has been previously analyzed in that same solvent [63]. Interestingly, there were some significant differences in their NMR spectra (Figures S20–S22). Remarkably, compound **2** lacked some signals observed in tenellone C, such as the ^13^C signal of the carbonyl group at C-8. Additionally, the HMBC correlation between H-14 and C-8, which was evident in tenellone C, was not observed in compound **2**. These findings effectively ruled out the hypothesis that compound **2** was identical to tenellone C. Conversely, the absence of a chemical shift at C-8 and the lack of an HMBC correlation between H-14 and C-8 have been observed in various instances where the isobenzofuran ring or indole rings were formed. This was notably observed in compounds such as isoprenylisobenzofuran A, as well as diaporisoindoles A and B [63,70]. However, it is important to mention that the structures and absolute configurations of these compounds (isoprenylisobenzofuran A and diaporisoindoles A and B) were confirmed through X-ray diffraction experiments. In the present study, an X-ray diffraction analysis was not pursued, and thus, the proposed planar structure of compound **2** was established based on the very close similarities of its spectral data (HR-ESIMS, 1D, and 2D NMR) with that of compound **9** [70]. As suggested by their molecular formula, the only remarkable difference was the incorporation of a hydroxyl group at C-8 in the structure of **2** instead of the hydrogen atom, as in **9**. To determine the absolute configuration of **2**, its ECD spectrum (Figure S24) was recorded and compared with that of 8-epi-isoprenylisobenzofuran A (lithocarol F) [71]. The Cotton effect pattern observed for compound **2** matched with that reported for lithocarol F [71] and enabled the assignment of the stereochemistry of **2** as 8*R*,2″*S*. The structure of compound **2** was therefore proposed and named isoprenylisobenzofuran B.

Compound **3** was isolated as pale yellow oil. Its purity was examined using HPLC-DAD-HR-ESIMS data (DAD detection at 210 nm/200–640 nm) which indicated the presence of a single compound (Figure S26). Its unique molecular formula C_25_H_30_O_6_ (indicating an index of hydrogen deficiency of 11) was, therefore, determined based on (+) HR-ESIMS *m*/*z* 449.1938 [M + Na]^+^ (calcd for C_25_H_30_NaO_6_^+^ 449.1935) and (−) HR-ESIMS *m*/*z* 425.1972 [M − H]^−^ (calcd for C_25_H_29_O_6_^−^ 425.1970). However, the features of the ^1^H and ^13^C-NMR spectra, which showed duplicated signals in a ratio of about 1.2:1, revealed that **3** was isolated as a mixture of stereoisomers. A detailed analysis of the 1D and 2D NMR spectroscopic data of **3** enabled the assignment of all protons and carbons (Table 3) corresponding to both isomers (**3a** and **3b**). The careful interpretation of 1D and 2D NMR data of compound **3** displayed close similarities to that of **2**. The main difference was the appearance of an O-bearing methylene group C-1 (δ_H_ 4.86 (H_a_-1), δ_H_ 4.97 (H_b_-1) and δ_C_ 69.1/69.2 (C-1)) in the structure of **3** (**3a** and **3b**) instead of the C-1 carbonyl group (δ_C_ 171.9), as observed in **2**. The structure of compound **3** was finally validated by the comprehensive analysis of its ^1^H-^13^C HMBC spectrum, which displayed key correlations with the methylene protons H_a_-1 and H_b_-1 with C-2/C-3/C-7/C-8 (Figure 5). The clear HMBC correlation of H-14 with C-8 that was observed in **3** further supports the linkage points of units A and B in its structure but also in that of its biogenetically related analog **2** described above, thus clarifying any ambiguity in the proposed structure of **2**. Furthermore, the stereochemistry of each isomer in the mixture was tentatively determined. Among the hitherto reported tenellone derivatives isolated from *Diaporthe* spp., the stereogenic carbon at C-2″ in the [1, 4] dioxane core was found to be *S*-configured [63,65,70,71,72,73,74,75]. Taking into account biosynthetic considerations, which suggested that the formation of the [1, 4] dioxane ring occurred stereo-specifically, an *S*-configuration at C-2″ for both isomers of compound **3** was therefore proposed. This implies that **3** is a mixture of epimers differing only in the stereochemistry of the O-bearing quaternary carbon C-8. The configuration of 8*S*,2″*S* was thus arbitrarily assigned to the major isomer **3a,** and the stereochemistry of the minor isomer **3b** (or 8-epi-**3a**) was deduced to be 8*R*,2″*S*. The structures of compound **3** were therefore characterized and assigned the trivial names of isoprenylisobenzofuran C_1_ (**3a**) and isoprenylisobenzofuran C_2_ (**3b**).

Compound **4** was obtained as a pale yellow oil with the molecular formula of C_25_H_29_NO_6_ (12 degrees of unsaturation), which was assigned based on (+) HR-ESIMS *m*/*z* 440.2064 [M + H]^+^ (calcd for 440.2068 C_25_H_30_NO_6_^+^). Its purity was ascertained by using HPLC-DAD-HR-ESIMS data (DAD detection at 210 nm/200–640 nm) which showed ion clusters corresponding to a single molecular formula, and the single peak observed on the UV chromatogram suggested the presence of a pure compound (Figure S35). However, the characteristics of its ^1^H and ^13^C NMR data were similar to those of compound **3** and equally displayed duplicated signals in a ratio of 3:2, suggesting that **4** was also isolated as an epimeric mixture (**4a** + **4b**). The interpretation of HR-ESIMS data of **4** (C_25_H_29_NO_6_) compared to that of **2** (C_25_H_28_O_7_) led to the hypothesis that the five-membered lactonic ring in **2** was possibly replaced by a five-membered lactam ring in **4**. This assumption was verified by a detailed analysis of the ^1^H-^13^C HMBC spectrum. The HMBC cross peaks of NH-16 with C-1/C-2/C-7/C-8 formed the key correlations, which validated the presence of the lactam ring in **4** (Figure 5). The full assignment of all ^1^H and ^13^C’s chemical shifts corresponding to both isomers of **4** was finally accomplished by a comprehensive examination of 1D and 2D NMR spectroscopic data (Table 4). As stipulated above, C-2″ was suggested to be biogenetically *S*-configured [63,65,70,71,72,73,74,75], thus indicating arbitrarily an 8*S*,2″*S* stereochemistry for the major isomer **4a** and 8*R*,2″*S* for the minor **4b**. The structures of **4** were thus determined and given the trivial names diaporisoindole F_1_ (**4a**) and diaporisoindole F_2_ (**4b**).

**Figure 6:**
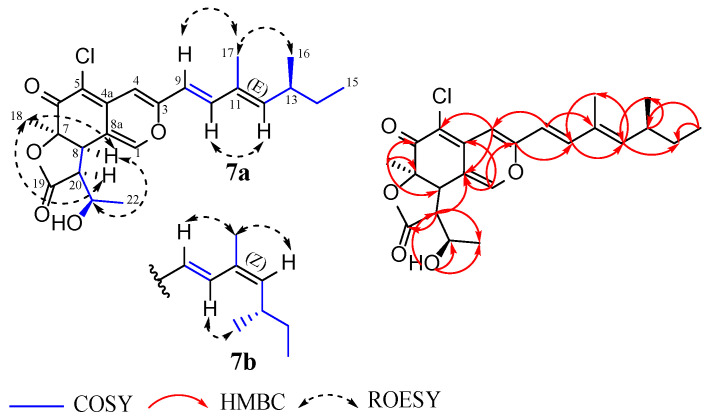
Key ^1^H-^1^H COSY, ^1^H-^13^C HMBC and ^1^H-^1^H ROESY correlations of compound **7**.

Compound **7** was isolated as a yellow amorphous solid. The molecular formula C_23_H_27_ClO_5_ (10 degrees of unsaturation) was deduced from its (+) HR-ESIMS, which showed a cluster of the sodium adduct [M + Na]^+^ at *m*/*z* 441.1437/443.1415 (Calcd for C_23_H_27_ClNaO_5_^+^ 441.1439) with a ratio of 3:1, which is indicative of a monochlorinated compound. Although an examination of its HPLC-DAD-MS chromatogram confirmed the purity of compound **7**, the ^1^H and ^13^C NMR spectra (recorded in DMSO-d_6_) rather exhibited duplicated signals, revealing that it was a mixture of isomers. The UV-Vis and ^1^H NMR spectra of **7** (Figures S45 and S52) displayed a typical pattern of an azaphilone skeleton [76]. A close inspection of its 1D NMR and ^1^H-^13^C HSQC spectra revealed characteristics that perfectly matched those of isochromophilonol: an azaphilone previously isolated from *Arcopilus cupreus* (syn. *Chaetomium cupreum*) RY202 [77]. The planar structure of this metabolite was further confirmed to be identical to that of isochromophinol by a careful analysis of its ^1^H-^1^H COSY and ^1^H-^13^C HMBC correlations (Figure 6). To make a proper comparison between compound **7** and the known isochromophilonol, the ^1^H NMR spectrum of compound **7** was additionally recorded in CDCl_3_ (Figure S51). Upon comparing the proton chemical shifts of compound **7** with those of isochromophilonol, noteworthy discrepancies, particularly for protons H-20, H-18, and H-22 (Table S2), were observed. This strongly suggests that compound **7** and isochromophinol did not have the same stereochemistry. In addition, the slight difference recorded between both isomers of **7** was rapidly detected on 1D NMR and ROESY spectra. A careful analysis of these spectra revealed a clear case of cis/trans isomerism through the observed variations in chemical shifts and correlations (^1^H and ^13^C NMR data of each isomer are summarized in Table 5). For the major isomer **7a**, an *E*-geometry for the ∆^11–12^ double bond was established based on ROESY correlations of H-16 with H-17 and H-10 with H-12. Concurrently, ROESY cross-peaks between H-12 and H-17, H-10 and H-16 were in favor of a Z-geometry for the ∆^11–12^ double bond in the minor isomer **7b** (Figure 6) [78]. Additionally, a large coupling constant *J*_9,10_ = 15.8 Hz indicated an *E*-geometry for the ∆^9–10^ double bond in both isomers. Furthermore, an investigative effort into the stereochemistry of compound **7** was completed by the detailed interpretation of its ^1^H-^1^H ROESY data and by comparison with its ECD spectra and its specific rotation with that of isochromophilonol [77]. On the ROESY spectrum of **7**, important correlations were observed (for both isomers) between H-18 and H-8/H-20 and H-8 and H-21, indicating that those protons were located on the same side (Figure 6). Noteworthy, the stereochemistry at position C-7 of the azaphilones has already been firmly established by optical rotations, circular dichroism, and X-ray analysis [79,80,81]. On the basis of the ECD spectra and the known 7*S* configuration of isochromophilonol [77], the 7*R* configuration was assigned to compound **7**. Explicitly, the ECD spectrum of **7** (Figure S53) showed a positive Cotton effect at ~315 nm (∆ε + 6.75), contrary to that of isochromophilonol, whose spectrum showed a negative cotton effect at the same wavelength [77]. Moreover, previous research by Whalley and co-workers has demonstrated that the sign of the specific rotation of azaphilones is seemingly influenced by the absolute configuration at the C-7 position [80]. Against this background, the opposite signs of specific rotation observed for **7** (${\left[\alpha \right]}_{D}^{20}$ + 839) compared to that of isochromophilonol (${\left[\alpha \right]}_{D}^{27}\;$− 141.6) further confirmed the 7*R* configuration assigned to this compound. Using C-7 as a reference, the absolute configuration at C-8, C-20, and C-21 was deduced as 8*R*, 20*S*, and 21*R* based on the aforementioned ROESY correlations. Taking into account biogenetic considerations, the absolute configuration at C-13 was proposed to be *S* since stereogenic carbon at C-13 in the aliphatic side chain was *S*-configured among the hitherto reported azaphilones; this harbored a branched C-7 side chain anchored at C-3 [64]. The structures of compound **7** were thus fully characterized and turned out to be unprecedented stereoisomers of isochromophilonol for which the trivial names isochromophilonol A_1_ (**7a**) and isochromophilonol A_2_ (**7b**) were assigned.

### 3.3. Physicochemical Data for Compounds ***1**–**4**, **7***

Eucalactam B (**1**): colorless oil.$\;{\left[\alpha \right]}_{D}^{20}\;-$ 7 (c 0.35, MeOH), UV (MeOH, c = 0.02 mg/mL) λ_max_ (log ε) 201 (4.1) nm. CD (c = 1.9 × 10^−3^ M, MeOH) λ_max_ (Δε) 200 (−6.03) nm. (+) HR-ESIMS *m*/*z* 536.2935 [M + Na]^+^, *m*/*z* 1049.5973 [2 M + Na]^+^, *m*/*z* 1027.6173 [2 M + H]^+^, *m*/*z* 496.3018 [M + H − H_2_O]^+^, *m*/*z* 514.3124 [M + H]^+^ (calcd for C_25_H_44_N_3_O_8_^+^ 514.3123). *t_R_* = 8.48 min (HR-LC-ESIMS). For NMR data (^1^H: 700 MHz, ^13^C: 175 MHz, DMSO-d_6_), see Table 2.

Isoprenylisobenzofuran B (**2**): pale yellow oil.$\;{\left[\alpha \right]}_{D}^{20}\;$+ 34 (c 0.25, MeOH), UV (MeOH, c = 0.01 mg/mL) λ_max_ (log ε) 312 (3.8) 269 (3.9) 208 (4.6) nm. CD (c = 2.3 × 10^−3^ M, MeOH) λ_max_ (Δε) 225 (−6.3) 208 (+2.3) nm. (+) HR-ESIMS *m*/*z* 463.1726 [M + Na]^+^, *m*/*z* 903.3537 [2 M + Na]^+^, *m*/*z* 423.1797 [M + H − H_2_O]^+^, *m*/*z* 441.1905 [M + H]^+^ (calcd for C_25_H_29_O_7_^+^ 441.1908). *t_R_* = 10.99 min (HR-LC-ESIMS). For NMR data (^1^H: 700 MHz, ^13^C: 175 MHz, CDCl_3_ + 1%TFA), see Table 3.

Isoprenylisobenzofuran C_1_ (**3a**)/C_2_ (**3b**): pale yellow oil. UV (MeOH, c = 0.01 mg/mL) λ_max_ (log ε) 284 (3.7) 204 (4.6) nm. (+) HR-ESIMS *m*/*z* 875.3981 [2 M + Na]^+^, *m*/*z* 409.2015 [M + H − H_2_O]^+^, *m*/*z* 449.1936 [M + Na]^+^ (calcd for C_25_H_30_NaO_6_^+^ 449.1935). (−) HR-ESIMS *m*/*z* 425.1972 [M − H]^−^ (calcd for C_25_H_29_O_6_^−^ 425.1970). *t_R_* = 10.65 min (HR-LC-ESIMS). For NMR data (^1^H: 500 MHz, ^13^C: 125 MHz, DMSO-d_6_), see Table 3.

Diaporisoindole F_1_ (**4a**)/F_2_ (**4b**): pale yellow oil. UV (MeOH, c = 0.01 mg/mL) λ_max_ (log ε) 294 (3.7) 206 (4.6) nm. (+) HR-ESIMS *m*/*z* 462.1882 [M + Na]^+^, *m*/*z* 901.3876 [2 M + Na]^+^, *m*/*z* 422.1959 [M + H − H_2_O]^+^, *m*/*z* 440.2065 [M + H]^+^ (calcd for C_25_H_30_NO_6_^+^ 440.2068). *t_R_* = 10.70 min (HR-LC-ESIMS). For NMR data (^1^H: 700 MHz, ^13^C: 175 MHz, DMSO-d_6_), see Table 4.

Isochromophilonol A_1_ (**7a**)/A_2_ (**7b**): yellow amorphous solid.$\;{\left[\alpha \right]}_{D}^{20}\;$+ 839 (c 0.1, MeOH), UV (MeOH, c = 0.01 mg/mL) λ_max_ (log ε) 409 (4.4), 363 (4.2) 252 (4.2), 209 (4.0) nm. CD (c = 2.4 × 10^−3^ M, MeOH) λ_max_ (Δε) 411 (+7.76) 311 (+6.75) 262 (+0.93) 229 (+3.54) nm. HR-ESIMS *m*/*z* 441.1437 [M + Na]^+^, *m*/*z* 859.2989 [2 M + Na]^+^, *m*/*z* 419.1623 [M + H]^+^ (calcd for C_23_H_28_ClO_5_^+^ 419.1620), *t_R_* = 13.49 min (HR-LC-ESIMS). For NMR data (^1^H: 500 MHz, ^13^C: 125 MHz, DMSO-d_6_), see Table 5.

### 3.4. Biological Activities of Compounds ***1**–**8***

The inhibitory potentials of compounds **1**–**8** against a panel of bacteria and fungi were evaluated. Compound **1** was found to be devoid of any activity against all the tested microorganisms. Except for compound **2**, which showed no activity, the tenellone derivatives (**3**–**6**) exhibited weak to moderate activity against certain microorganisms that were tested, namely *B. subtilis*, *M. hiemalis*, *R. glutinis*, *S. pombe,* and *W. anomalus* with MIC values in the range of 16.6–66.6 µg/mL (Table 6). Tenellone derivatives were previously found to exhibit interesting biological effects, and their main reported properties involved anti-tumor and anti-inflammatory activities [70,71,73,75,82]. However, to the best of our knowledge, their antimicrobial activities have hitherto not been reported. This study is thus the first to outline the antimicrobial effects of this class of compounds. In addition, compound **7** also showed weak to moderate activity against the Gram-positive bacteria *B. subtilis* (MIC 16.6 µg/mL), *M. smegmatis* (MIC 16.6 µg/mL), and *S. aureus* (MIC 66.6 µg/mL). Remarkably, its activity against the Mucoromycota fungus *M. hiemalis* was significant, reaching an MIC of 8.3 µg/mL, which is equal to that of nystatin used as a positive control. However, further investigations need to be conducted to delineate whether only one of the isomers is active or if the reported activity is the result of a synergistic effect. Furthermore, the cyclic hexadepsipeptide (**8**) was also active in the current assay, with MICs ranging from 8.3 to 66.6 µg/mL against some fungi and Gram-positive bacteria (Table 6).

Different mammalian cell lines were used to assess the cytotoxicity of the isolated metabolites **1**–**8**. In this assay, compound **1** did not show any cytotoxic effect under the tested conditions. Amidst the tenellone derivatives (**2**–**6**), compound **2** only demonstrated a slight inhibition of KB3.1 cell proliferation, whereas the other compounds (**3**–**6**) inhibited the growth of KB3.1 and L929 cell lines with IC_50_ values in the range of 20–59.2 µM (Table 7). Among these, compound **6** exhibited the strongest cytotoxic activity against the cancer cell line KB3.1 and the murine fibroblasts of line L929. This compound was thus further evaluated against five other mammalian cell lines, namely A431, MCF-7, A549, SKOV-3, and PC-3, and exhibited cytotoxic effects with IC_50_ values ranging from 17.7 to 42.5 µM. As stipulated in the previous paragraph, several tenellone-derived representatives have proven to possess interesting antitumor properties. Therefore, the cytotoxic activity of compounds **3**–**6** presented in this study corroborates previously published data [71,72,73,75]. This encourages further investigations into the cytotoxic effects of novel target molecules in this class for the discovery of potential antitumor agents. Interestingly, during the evaluation of the biological properties of diaporisoindoles A and B by Cui and their collaborators [63], diaporisoindole A with a configuration of 8*S*,2″*S* showed potent inhibitory activity against MptpB. However, diaporisoindole B (8*R*,2″*S* or 8-epi-diaporisoindole A) failed to show any activity under the test conditions, suggesting that the *S*-configuration at C-8 in these tenellone analogs probably promoted their inhibitory effect. The latter could explain the lack of activity observed for compound **2** (8*R*,2″*S*-configured) in comparison to the epimeric mixtures **3**–**5**. In addition, compounds **7** and **8** exhibited significant growth inhibitory effects against the six cancer and the murine fibroblast cell lines assessed with IC_50_ values in the range of 0.9−12.9 µM and 1.9−3.3 µM, respectively (Table 7). Interestingly, the so-called emerging mycotoxin beauvericin (**8**), which belongs to the enniatin antibiotic family, has attracted a lot of attention over recent years due to its multifaceted nature. This compound has recently been recognized as a promising candidate for anticancer therapy, as reported by Sood et al. [83] and Wu et al. [84]. However, its potential as an emerging mycotoxin and its impact on animals, humans, and the environment still require clarification and further investigation [68]. In addition, beauvericin also displays other biological effects, including antibacterial, antiviral, antifungal, insecticidal, and nematicidal activities, to mention a few [68,84]. Therefore, the herein-reported antimicrobial and cytotoxic activities of metabolite **8** are consistent with previous research findings.

## 4. Conclusions

In our continuous search for new therapeutic molecules, we investigated the secondary metabolism of endophytic fungal strains associated with Cameroonian medicinal plants. Through our extensive screening efforts, we were able to identify a new fungal species named *Diaporthe africana,* from which four novel polyketides were obtained alongside four known compounds. The isolation, characterization, and biological evaluation of these compounds not only demonstrated the potential of the *Diaporthe* species as a source of novel and structurally diverse bioactive compounds but also emphasized the importance of investigating new taxa of even well-studied phylogenetic groups such as *Diaporthe* for discovery of new forms of chemical diversity. In this study, two rationales were utilized to maximize the chances of expanding chemical diversity within the widely explored genus *Diaporthe*. On the one hand, we focused on new endophytic fungal strains from underexplored geographic areas like the tropical rainforests of Cameroon, which are known to possess a rich diversity of plant species that remain widely untapped in terms of the plant–endophyte interaction as well as the metabolites produced by these endophytes. On the other hand, traditional medicinal plants or plants with an ethnopharmacological background were selected for the isolation of these endophytes. These research findings complement previous results in relation to investigating chemical and biological diversity of the genus *Diaporthe* and some of the metabolites herein reported add to the growing pool of bioactive compounds that could have therapeutic applications. In addition, this work provides further evidence of the potential of endophytes as a promising source of new biologically active compounds and underscores the importance of further exploring these microorganisms for the discovery of potentially leading drug candidates.

## Figures and Tables

**Table 1:** Isolates and reference strains of *Diaporthe* spp. included in the phylogenetic study. GenBank accession numbers in bold were newly generated in this study. Taxonomic novelty is indicated in bold italic.

Species	Isolates ^1^	GenBank Accession Numbers ^2^					References
		ITS	*tub2*	*his3*	*tef1*	*cal*	
*Diaporthe acuta*	PSCG 047^T^	MK626957	MK691225	MK726161	MK654802	MK691125	Guo et al. [23]
***D. africana***	**CBS 150080^T^**	**OR198681**	**OR225229**	**OR225231**	**OR225227**	**OR225233**	**Present study**
	**STMA 18294**	**OR198680**	**OR225230**	**OR225232**	**OR225228**	**OR225234**	**Present study**
*D. amygdali*	CBS 126679^T^	KC343022	KC343990	KC343506	KC343748	KC343264	Gomes et al. [24]
*D. anhuiensis*	CNUCC 201902^T^	MN219727	MN227009	MN224550	MN224669	MN224556	Zhou and Hou [25]
*D. annellsiae*	BRIP 59731a^T^	OM918687	OM960614	-	OM960596	-	Tan and Shivas [26]
*D. arecae*	CBS 161.64^T^	KC343032	KC344000	KC343516	KC343758	KC343274	Gomes et al. [24]
*D. arengae*	CBS 114979^T^	KC343034	KC344002	KC343518	KC343760	KC343276	Gomes et al. [24]
*D. aseana*	MFLUCC 12-0299a^T^	KT459414	KT459432	-	KT459448	KT459464	Hyde et al. [27]
*D. australiana*	BRIP 66145^T^	MN708222	MN696530	-	MN696522	-	Wrona et al. [28]
*D. averrhoae*	SCHM 3605^T^	AY618930	-	-	-	-	Chang et al. [29]
*D. bounty*	BRIP 59361a^T^	OM918690	OM960617	-	OM960599	-	Tan and Shivas [26]
*D. camelliae-oleiferae*	HNZZ 027^T^	MZ509555	MZ504718	MZ504696	MZ504707	MZ504685	Yang et al. [30]
*D. ceratozamiae*	CBS 131306^T^	JQ044420	-	-	-	-	Crous et al. [31]
*D. cercidis*	CFCC 52565^T^	MH121500	MH121582	MH121460	MH121542	MH121424	Yang et al. [32]
*D.* cf. *heveae*	CBS 681.84	KC343117	KC344085	KC343601	KC343843	KC343359	Gomes et al. [24]
*D. chiangmaiensis*	MFLUCC 18-0544^T^	OK393703	-	-	OL439483	-	de Silva et al. [33]
*D. chrysalidocarpi*	SAUCC 194.35^T^	MT822563	MT855760	MT855532	MT855876	MT855646	Huang et al. [34]
*D. delonicis*	MFLU 16-1059^T^	MT215490	MT212209	-	-	-	Perera et al. [35]
*D. drenthii*	BRIP 66524^T^	MN708229	MN696537	-	MN696526	-	Wrona et al. [28]
*D. endocitricola*	ZHKUCC 20-0012^T^	MT355682	MT409290	-	MT409336	MT409312	Dong et al. [36]
*D. eres*	CBS 138594^T^	KJ210529	KJ420799	KJ420850	KJ210550	KJ434999	Udayanga et al. [37]
*D. eucalyptorum*	CBS 132525^T^	JX069862	-	-	-	-	Crous et al. [38]
*D. eugeniae*	CBS 444.82	KC343098	KC344066	KC343582	KC343824	KC343340	Gomes et al. [24]
*D. fraxini-angustifoliae*	BRIP 54781^T^	JX862528	KF170920	-	JX852534	-	Tan et al. [39]
*D. fujianensis*	JZB 320149^T^	MW010212	MW056008	-	-	MW205212	Manawasinghe et al. [40]
*D. fulvicolor*	PSCG 051^T^	MK626859	MK691236	MK726163	MK654806	MK691132	Guo et al. [23]
*D. fusiformis*	JZB 320156^T^	MW010218	MW056014	-	MW205234	MW205218	Manawasinghe et al. [40]
*D. gossiae*	BRIP 59730a^T^	OM918693	OM960620	-	OM960602	-	Tan and Shivas [26]
*D. guangxiensis*	JZB 320094^T^	MK335772	MK500168	-	MK523566	MK736727	Manawasinghe et al. [41]
*D. hongkongensis*	CBS 115448^T^	KC343119	KC344087	KC343603	KC343845	KC343361	Gomes et al. [24]
*D. howardiae*	BRIP 59697a^T^	OM918695	OM960622	-	OM960604	-	Tan and Shivas [26]
*D. huangshanensis*	CNUCC 201903^T^	MN219730	MN227011	MN224558	MN224678	-	Zhou and Hou [25]
*D. hunanensis*	HNZZ 023^T^	MZ509550	MZ504713	MZ504691	MZ504702	MZ504680	Yang et al. [30]
*D. krabiensis*	MFLUCC 17-2481^T^	MN047101	MN431495	-	MN433215		Dayarathne et al. [42]
*D. limonicola*	CBS 142549^T^	MF418422	MF418582	MF418342	MF418501	MF418256	Guarnaccia and Crous [43]
*D. liquidambari*	SCHM 3621^T^	AY601919	-	-	-	-	Chang et al. [29]
*D. litchicola*	BRIP 54900^T^	JX862533	KF170925	-	JX862539	-	Tan et al. [39]
*D. lithocarpi*	CGMCC 3.15175^T^	KC153104	KF576311	-	KC153095	-	Gao et al. [44]
*D. meliae*	CFCC 53089^T^	MK432657	MK578057	ON081662	ON081654	-	Cao et al. [45]
*D. melitensis*	CBS 142551^T^	MF418424	MF418584	MF418344	MF418503	MF418258	Guarnaccia and Crous [43]
*D. millettiae*	GUCC 9167^T^	MK398674	MK502089	-	MK480609	MK502086	Long et al. [46]
*D. musigena*	CBS 129519^T^	KC343143	KC344111	KC343627	KC343869	KC343385	Gomes et al. [24]
*D. norfolkensis*	BRIP 59718a^T^	OM918699	OM960626	-	OM960608	-	Tan and Shivas [26]
*D. oculi*	HHUF 30565^T^	LC373515	LC373519	-	LC373517	-	Ozawa et al. [47]
*D. osmanthi*	GUCC 9165^T^	MK398675	MK502090	-	MK480610	MK502087	Long et al. [46]
*D. pandanicola*	MFLUCC 17-0607^T^	MG646974	MG646930	-	-	-	Tibpromma et al. [48]
*D. pascoei*	BRIP 54847^T^	JX862532	KF170924	-	JX862538	-	Tan et al. [39]
*D. perseae*	CBS 151.73	KC343173	KC344141	KC343657	KC343899	KC343415	Gomes et al. [24]
*D. pescicola*	MFLUCC 16-0105^T^	KU557555	KU557579	-	KU557623	KU557603	Dissanayake et al. [49]
*D. phyllanthicola*	SCHM 3680^T^	AY620819	-	-	-	-	Chang et al. [29]
*D. podocarpi-macrophylli*	CGMCC 3.18281^T^	KX986774	KX999207	KX999246	KX999167	KX999278	Gao et al. [50]
*D. pseudomangiferae*	CBS 101339^T^	KC343181	KC344149	KC343665	KC343907	KC343423	Gomes et al. [24]
*D. pseudooculi*	HHUF 30617^T^	LC373515	LC373519	-	LC373517	-	Ozawa et al. [47]
*D. pseudophoenicicola*	CBS 462.69^T^	KC343184	KC344152	KC343668	KC343910	KC343426	Gomes et al. [24]
*D. pterocarpicola*	MFLUCC 10-0580a	JQ619887	JX275441	-	JX275403	JX197433	Udayanga et al. [51]
*D. rhodomyrti*	CFCC 53101^T^	MK432643	MK578046	MK442990	MK578119	MK442965	Cao et al. [45]
*D. salinicola*	MFLU 18-0553^T^	MN047098	-	-	MN077073	-	Dayarathne et al. [42]
*D. schimae*	CFCC 53103^T^	MK432640	MK578043	MK442987	MK578116	MK442962	Yang et al. [52]
*D. searlei*	BRIP 66528^T^	MN708231	MN696540	-	-	-	Wrona et al. [28]
*D. sennae*	CFCC 51636^T^	KY203724	KY228891	-	KY228885	KY228875	Yang et al. [53]
*D. spinose*	PSCG 383^T^	MK626849	MK691234	MK726156	MK654811	MK691129	Guo et al. [23]
*D. taoicola*	MFLUCC 16-0117^T^	KU557567	KU557591	-	KU557635	-	Dissanayake et al. [49]
*D. tectonigena*	MFLUCC 12-0767^T^	KU712429	KU743976	-	KU749371	KU749358	Doilom et al. [54]
*D. viniferae*	JZB 320071^T^	MK341551	MK500112	-	MK500107	MK500119	Manawasinghe et al. [41]
*D. xishuangbanica*	CGMCC 3.18282^T^	KX986783	KX999216	KX999255	KX999175	-	Gao et al. [50]

^1^ BRIP: Queensland Plant Pathology Herbarium, Brisbane, Australia; CBS: Westerdijk Fungal Biodiversity Institute, Utrecht, the Netherlands; CFCC: China Forestry Culture Collection Center, Beijing, China; CGMCC: Chinese General Microbiological Culture Collection Center, Beijing, China; CNUCC: Capital Normal University Culture Collection Center, Beijing, China; GUCC: Culture Collection at the Department of Plant Pathology, Agriculture College, Guizhou University, China; HNZZ: Central South University of Forestry and Technology, Changsha, China; JZB: Culture collection of Institute of Plant and Environment Protection, Beijing, China; MFLU: Mae Fah Luang University herbarium, Thailand; MFLUCC: Mae Fah Luang University Culture Collection, Chiang Rai, Thailand; PSCG: Personal Culture Collection Y.S. Guo, China; SAUCC: Shandong Agricultural University Culture Collection, Shandong, China; SCHM: Mycological Herbarium of South China Agricultural University, Guangzhou, China; STMA: fungarium of the Helmholtz Centre for Infection Research, Braunschweig, Germany; ZHKUCC: Culture Collection of Zhongkai University of Agriculture and Engineering, Guangzhou, China. ^T^ indicates ex-type material. ^2^ ITS: internal transcribed spacers and intervening 5.8S nrDNA; *tub2*: partial β-tubulin gene; *his3*: partial histone H3 gene; *tef1*: partial elongation factor 1-alpha gene; *cal*: partial calmodulin gene.

**Table 2:** NMR spectroscopic data for compound **1** (^1^H 700 MHz, ^13^C 175 MHz, in DMSO-d_6_).

		1	
	No.	*δ*_C_, Type	*δ*_H_ (*J* in Hz)
Fragment A	1	169.3, C	-
	2	40.1, CH_2_	3.69, dd (17.7, 4.9) 3.94, dd (17.7, 7.3)
	3-NH	-	8.36, dd (6.9, 5.1)
	4	168.6, C	-
	5	41.6, CH_2_	3.77, d (4.9)
	6-NH	-	7.43, t (5.0)
Fragment B	7	170.4, C	-
	8	58.6, CH	3.98, dd (7.8, 2.9)
	9-NH	-	7.85, d (7.8)
	24	65.3, CH	4.18, qd (6.3, 2.8)
	24-OH	-	5.02, br s
	25	20.6, CH_3_	1.02, d (6.4)
Fragment C	10	175.3, C	-
	11	45.7, CH	2.37, qd (7.5, 3.2)
	12	70.6, CH	3.83, m
	13	41.2, CH_2_	1.65, t (6.9)
	14	68.9, CH	3.57, quin (6.1)
	14-OH	-	4.72, br s
	15	36.4, CH_2_	1.38, m 1.48, m
	16	27.5, CH_2_	1.88, m 1.99, m
	17	130.7, CH	5.45, m
	18	131.6, CH	5.09, dd (15.4, 8.9)
	19	39.0, CH	2.32, m
	20	78.5, CH	4.67, dd (9.6, 2.4)
	21	34.8, CH_2_	1.62, td (7.0, 2.4)
	22	26.4, CH_2_	1.15, m
	23	11.7, CH_3_	0.84, t (7.1)
	26	16.2, CH_3_	1.20, d (7.3)
	27	17.2, CH_3_	0.88, d (6.9)
	28	12.6, CH_3_	0.83, d (7.0)

**Table 3:** NMR data for compounds **2** (^1^H 700 MHz, ^13^C 175 MHz, in CDCl_3_ + 1% TFA) and **3** (^1^H 700 MHz, ^13^C 175 MHz, in DMSO-d_6_).

	2		3a		3b	
	*δ*_C_, Type	*δ*_H_ (*J* in Hz)	*δ*_C_, Type	*δ*_H_ (*J* in Hz)	*δ*_C_, Type	*δ*_H_ (*J* in Hz)
1	171.9, C	-	69.1, C	4.97, d (12.3) 4.86, d (12.3)	69.2, C	4.97, d (12.3) 4.86, d (12.3)
2	109.5, C *	-	125.3, C	-	125.3, C	-
3	157.5, C *	-	149.5, C	-	149.6, C	-
4	119.0, CH	7.06, d (8.6)	114.4, CH	6.63, d (8.1)	114.4, CH	6.59, d (8.1)
5	138.5, CH	7.46, d (8.6)	128.7, CH	6.75, d (8.1)	128.8, CH	6.74, d (8.1)
6	129.8, C	-	125.5, C	-	125.6, C	-
7	143.5, C *	-	142.6, C	-	142.6, C	-
8	-	-	105.8, C	-	106.0, C	-
9	131.0, C	-	130.6, C	-	131.1, C	-
10	140.9, C	-	139.3, C	-	139.6, C	-
11	143.3, C	-	142.6, C	-	142.5, C	-
12	122.1, CH	6.9, br s	117.1, CH	6.64, d (2.1)	117.0, CH	6.64, d (2.1)
13	131.0, C	-	127.7, C	-	128.1, C	-
14	122.9, CH	6.9, br s	120.2, CH	7.24, d (2.1)	120.1, CH	7.22, d (2.1)
15	20.6, CH_3_	2.24, s	20.6, CH_3_	2.2, s	20.7, CH_3_	2.2, s
1′	29.7, CH_2_	3.13, br d (6.7)	28.0, CH_2_	2.8, m (overlapped)	28.0, CH_2_	2.8, m (overlapped)
2′	121.4, CH	5.06, br t (6.7)	123.6, CH	4.8, t (7.1)	123.7, CH	4.8, t (7.1)
3′	133.9, C	-	130.2, C	-	130.2, C	-
4′	17.6, CH_3_	1.50, s	17.4, CH_3_	1.33, s	17.4, CH_3_	1.34, s
5′	25.6, CH_3_	1.64, s	25.5, CH_3_	1.51, s	25.6, CH_3_	1.52, s
1″	64.2, CH_2_	3.93, t (9.7) 4.37, br d (9.5)	63.7, CH_2_	4.27, dd (11.1, 2.1) 3.48, br dd (11.1, 9.0)	64.3, CH_2_	4.27, dd (11.1, 2.0) 3.73, dd (11.1, 9.2)
2″	79.6, CH	3.99, m	79.1, CH	3.60, dd (9.0, 2.3)	79.0, CH	3.23, dd (9.2, 1.9)
3″	72.4, C	-	68.8, C	-	69.1, C	-
4″	23.5, CH_3_	1.20, s	22.2, CH_3_	0.35, s	23.0, CH_3_	0.52, s
5″	25.6, CH_3_	1.30, s	27.4, CH_3_	0.85, s	27.2, CH_3_	0.84, s

* Chemical shifts assigned from the ^1^H-^13^C HMBC spectrum.

**Table 4:** NMR data for compound **4** (^1^H 700 MHz, ^13^C 175 MHz, in DMSO-d_6_).

No.	4a		4b	
	*δ*_C_, Type	*δ*_H_ (*J* in Hz)	*δ*_C_, Type	*δ*_H_ (*J* in Hz)
1	169.9, C	-	169.9, C	-
2	115.8, C	-	115.9, C	-
3	153.2, C	-	153.3, C	-
4	115.8, CH	6.73, d (8.2)	115.6, CH	6.70, d (8.2)
5	134.1, CH	7.02, d (8.2)	134.2, CH	7.02, d (8.2)
6	127.0, C	-	127.0, C	-
7	148.0, C	-	148.0, C	-
8	84.9, C	-	85.1, C	-
9	127.9, C	-	127.9, C	-
10	138.6, C	-	138.8, C	-
11	142.6, C	-	142.7, C	-
12	117.1, CH	6.67, d (2.1)	117.1, CH	6.67, d (2.1)
13	128.3, C	-	128.5, C	-
14	120.7, CH	7.33, br s	120.7, CH	7.33, br s
15	20.6, CH_3_	2.23, s	20.6, CH_3_	2.23, s
16-NH	-	8.79, s	-	9.04, br s
1′	27.8, CH_2_	2.92, m (overlapped)	27.8, CH_2_	2.92, m (overlapped)
2′	122.9, CH	4.79, br t (6.6)	123.0, CH	4.79, br t (6.6)
3′	130.7, C	-	130.8, C	-
4′	17.3, CH_3_	1.35, s	17.3, CH_3_	1.37, s
5′	25.4, CH_3_	1.50, s	25.5, CH_3_	1.52, s
1″	63.8, CH_2_	3.46, dd (11.2, 9.2) 4.31, m	64.1, CH_2_	3.68, dd (11.2, 9.2)4.31, m
2″	79.4, CH	3.56, dd (9.0, 1.8)	79.4, CH	3.29, m
3″	68.8, C	-	68.5, C	-
4″	21.9, CH_3_	0.33, s	22.7, CH_3_	0.60, s
5″	27.7, CH_3_	0.92, s	27.4, CH_3_	0.86, s

**Table 5:** NMR data for compound **7** (^1^H 500 MHz, ^13^C 125 MHz, in DMSO-d_6_).

No.	7a		7b	
	*δ*_C_, Type	*δ*_H_ (*J* in Hz)	*δ*_C_, Type	*δ*_H_ (*J* in Hz)
1	144.8, CH	7.78, d (1.7)	144.8, CH	7.83, d (1.7)
2	-	-	-	-
3	157.6, C	-	157.2, C	-
4	105.0, CH	6.82, s	105.8, CH	6.88, s
4a	142.6, C	-	142.5, C	-
5	107.4, C	-	107.7, C	-
6	185.5, C	-	185.6, C	-
7	81.9, C	-	81.9, C	-
8	43.3, CH	3.54, dd (11.9, 1.7)	43.1, CH	3.54, dd (11.9, 1.7)
8a	116.2, C	-	116.2, C	-
9	117.6, CH	6.51, d (15.9)	120.2, CH	6.61, d (15.7)
10	140.8, CH	7.07, d (15.9)	132.2, CH	7.41, d (15.7)
11	132.2, C	-	130.2, C	-
12	146.5, CH	5.73, d (9.8)	144.0, CH	5.52, d (9.9)
13	34.3, CH	2.46, m	33.4, CH	2.64, m
14	29.6, CH_2_	1.39, m 1.26, m	29.7, CH_2_	1.39, m 1.26, m
15	11.9, CH_3_	0.82, t (7.4)	11.9, CH_3_	0.82, t (7.4)
16	20.2, CH_3_	0.96, d (6.6)	21.0, CH_3_	0.96, d (6.6)
17	12.3, CH_3_	1.81, d (0.9)	19.9, CH_3_	1.88, d (1.1)
18	18.7, CH_3_	1.20, s	18.7, CH_3_	1.20, s
19	174.6, C	-	174.6, C	-
20	47.0, CH	3.41, br dd (11.9, 5.7)	47.0, CH	3.41, br dd (11.9, 5.7)
21	65.7, CH	4.04, m	65.6, CH	4.04, m
21-OH	-	5.22, br d (4.4)	-	5.22, br d (4.4)
22	22.7, CH_3_	1.35, d (6.3)	22.8, CH_3_	1.35, d (6.3)

**Table 6:** Antimicrobial activities of compounds **1**–**8**.

MIC (µg/mL)									
Test Organisms	1	2	3 (3a + 3b)	4 (4a + 4b)	5	6	7 (7a + 7b)	8	References
*Acinetobacter baumanii*	-	-	-	-	-	-	-	-	0.26 ^c^
*Bacillus subtilis*	-	-	33.3	66.6	66.6	16.6	16.6	8.3	8.3 ^o^
*Candida albicans*	-	-	-	-	-	-	-	-	16.6 ^n^
*Chromobacterium violaceum*	-	-	-	-	-	-	-	-	0.83 ^o^
*Escherichia coli*	-	-	-	-	-	-	-	-	1.7 ^o^
*Mucor hiemalis*	-	-	66.6	33.3	66.6	16.6	8.3	66.6	8.3 ^n^
*Mycobacterium smegmatis*	-	-	-	-	-	-	16.6	8.3	1.7 ^k^
*Pseudomonas aeruginosa*	-	-	-	-	-	-	-	-	0.21 ^g^
*Rhodoturula glutinis*	-	-	-	33.3	66.6	16.6	-	66.6	4.2 ^n^
*Schizosaccharomyces pombe*	-	-	-	33.3	33.3	66.6	-	33.3	8.3 ^n^
*Staphylococcus aureus*	-	-	33.3	66.6	66.6	16.6	66.6	8.3	0.83 ^o^
*Wickerhamomyces anomalus*	-	-	-	-	-	66.6	-	-	8.3 ^n^

(-): No inhibition, ^c^ Ciprobay 2.54 mg/mL, ^g^ Gentamicin 1 mg/mL, ^k^ Kanamycin 1 mg/mL, ^n^ Nystatin 1 mg/mL, ^o^ Oxytetracyclin 1 mg/mL. The starting concentration for antimicrobial assay was adjusted to 66.7 μg/mL.

**Table 7:** Cytotoxic activities of compounds **1**–**8**.

IC_50_ (µM)									
Cell Lines	1	2	3 (3a + 3b)	4 (4a + 4b)	5	6	7 (7a + 7b)	8	Epothilone B
KB3.1	-	SI	32.9	31.9	22.0	20.0	5.3	2.3	4.5 × 10^−5^
L929	-	-	51.6	59.2	54.4	49.5	12.9	2.8	4.3 × 10^−4^
A431	n.t	n.t	n.t	n.t	n.t	19.3	4.1	2.4	5.5 × 10^−5^
MCF-7	n.t	n.t	n.t	n.t	n.t	17.7	0.9	2.0	6.1 × 10^−5^
A549	n.t	n.t	n.t	n.t	n.t	20.8	2.3	2.6	6.9 × 10^−5^
SKOV-3	n.t	n.t	n.t	n.t	n.t	42.5	3.3	3.3	9.9 × 10^−5^
PC-3	n.t	n.t	n.t	n.t	n.t	17.9	1.1	1.9	7.3 × 10^−5^

SI: slight inhibition of cell proliferation, (-) not active, n.t: not tested. The starting concentration for the cytotoxic assay was adjusted to 37 µg/mL.

## Data Availability

The DNA sequences are deposited in GenBank (https://www.ncbi.nlm.nih.gov/genbank/) and all other relevant data are included in the Supplementary Information.

## Supplementary Materials

The following supporting information can be downloaded at: https://www.mdpi.com/article/10.3390/jof9070781/s1, Figure S1: HPLC-DAD chromatogram and ESI-MS data for eucalactam B (**1**), Figure S2: HPLC-DAD chromatogram and HR-ESI (+) MS data for eucalactam B (**1**), Figure S3: ^1^H NMR spectrum (DMSO-d_6_, 700 MHz) of eucalactam B (**1**), Figure S4: ^13^C NMR spectrum (DMSO-d_6_, 175 MHz) of eucalactam B (**1**), Figure S5: ^1^H-^1^H COSY NMR spectrum (DMSO-d_6_, 700 MHz) of eucalactam B (**1**), Figure S6: ^1^H-^13^C HSQC NMR spectrum (DMSO-d_6_, 700 MHz) of eucalactam B (**1**), Figure S7: ^1^H-^13^C HMBC NMR spectrum (DMSO-d_6_, 700 MHz) of eucalactam B (**1**), Figure S8: ^1^H-^1^H ROESY NMR spectrum (DMSO-d_6_, 700 MHz) of eucalactam B (**1**), Figure S9: ^1^H NMR spectrum (CDCl_3_, 500 MHz) of eucalactam B (**1**), Figure S10: UV-Vis spectrum (MeOH) of eucalactam B (**1**), Figure S11: ECD spectrum (MeOH) of eucalactam B (**1**), Figure S12: HPLC-DAD chromatogram and ESI-MS data for isoprenylisobenzofuran B (**2**), Figure S13: HPLC-DAD chromatogram and HR-ESI (+) MS data for isoprenylisobenzofuran B (**2**), Figure S14: ^1^H NMR spectrum (CDCl_3_ + 1%TFA, 700 MHz) of isoprenylisobenzofuran B (**2**), Figure S15: ^13^C NMR spectrum (CDCl_3_ + 1%TFA, 175 MHz) of isoprenylisobenzofuran B (**2**), Figure S16: ^1^H-^1^H COSY NMR spectrum (CDCl_3_ + 1%TFA, 700 MHz) of isoprenylisobenzofuran B (**2**), Figure S17: ^1^H-^13^C HSQC NMR spectrum (CDCl_3_ + 1%TFA, 700 MHz) of isoprenylisobenzofuran B (**2**), Figure S18: ^1^H-^13^C HMBC NMR spectrum (CDCl_3_ + 1%TFA, 700 MHz) of isoprenylisobenzofuran B (**2**), Figure S19: ^1^H-^1^H ROESY NMR spectrum (CDCl_3_ + 1%TFA, 700 MHz) of isoprenylisobenzofuran B (**2**), Figure S20: ^1^H NMR spectrum (MeOH-d_4_, 500 MHz) of compound **2**, Figure S21: ^13^C NMR spectrum (MeOH-d_4_, 125 MHz) of compound **2**, Figure S22: ^1^H-^13^C HMBC NMR spectrum (MeOH-d_4_, 500 MHz) of compound **2**, Figure S23: UV-Vis spectrum (MeOH) of isoprenylisobenzofuran B (**2**), Figure S24: ECD spectrum (MeOH) of isoprenylisobenzofuran B (**2**), Figure S25: HPLC-DAD chromatogram and ESI-MS data for isoprenylisobenzofuran C_1_/C_2_ (**3a** + **3b**), Figure S26: HPLC-DAD chromatogram and (+)/(−) HR-ESIMS data for isoprenylisobenzofuran C_1_/C_2_ (**3a** + **3b**), Figure S27: ^1^H NMR spectrum (DMSO-d_6_, 700 MHz) of isoprenylisobenzofuran C_1_/C_2_ (**3a** + **3b**), Figure S28: ^13^C NMR spectrum (DMSO-d_6_, 175 MHz) of isoprenylisobenzofuran C_1_/C_2_ (**3a** + **3b**), Figure S29: ^1^H-^1^H COSY NMR spectrum (DMSO-d_6_, 700 MHz) of isoprenylisobenzofuran C_1_/C_2_ (**3a** + **3b**), Figure S30: ^1^H-^13^C HSQC NMR spectrum (DMSO-d_6_, 700 MHz) of isoprenylisobenzofuran C_1_/C_2_ (**3a** + **3b**), Figure S31: ^1^H-^13^C HMBC NMR spectrum (DMSO-d_6_, 700 MHz) isoprenylisobenzofuran C_1_/C_2_ (**3a** + **3b**), Figure S32: ^1^H-^1^H ROESY NMR spectrum (DMSO-d_6_, 700 MHz) of isoprenylisobenzofuran C_1_/C_2_ (**3a** + **3b**), Figure S33: UV-Vis spectrum (MeOH) of isoprenylisobenzofuran C_1_/C_2_ (**3a** + **3b**), Figure S34: HPLC-DAD chromatogram and ESI-MS data for diaporisoindole F_1_/F_2_ (**4a** + **4b**), Figure S35: HPLC-DAD chromatogram and HR-ESI (+) MS data for diaporisoindole F_1_/F_2_ (**4a** + **4b**), Figure S36: ^1^H NMR spectrum (DMSO-d_6_, 700 MHz) of diaporisoindole F_1_/F_2_ (**4a** + **4b**), Figure S37: ^13^C NMR spectrum (DMSO-d_6_, 175 MHz) of diaporisoindole F_1_/F_2_ (**4a** + **4b**), Figure S38: ^1^H-^1^H COSY NMR spectrum (DMSO-d_6_, 700 MHz) of diaporisoindole F_1_/F_2_ (**4a** + **4b**), Figure S39: ^1^H-^13^C HSQC NMR spectrum (DMSO-d_6_, 700 MHz) of diaporisoindole F_1_/F_2_ (**4a** + **4b**), Figure S40: ^1^H-^13^C HMBC NMR spectrum (DMSO-d_6_, 700 MHz) of diaporisoindole F_1_/F_2_ (**4a** + **4b**), Figure S41: ^1^H-^1^H ROESY NMR spectrum (DMSO-d_6_, 700 MHz) of diaporisoindole F_1_/F_2_ (**4a** + **4b**), Figure S42: UV-Vis spectrum (MeOH) of diaporisoindole F_1_/F_2_ (**4a** + **4b**), Figure S43: HPLC-DAD chromatogram and ESI-MS data for isochromophilonol A_1_/A_2_ (**7a** + **7b**), Figure S44: HPLC-DAD chromatogram and HR-ESI (+) MS data for isochromophilonol A_1_/A_2_ (**7a** + **7b**), Figure S45: ^1^H NMR spectrum (DMSO-d_6_, 500 MHz) of isochromophilonol A_1_/A_2_ (**7a** + **7b**), Figure S46: ^13^C NMR spectrum (DMSO-d_6_, 125 MHz) of isochromophilonol A_1_/A_2_ (**7a** + **7b**), Figure S47: ^1^H-^1^H COSY NMR spectrum (DMSO-d_6_, 500 MHz) of isochromophilonol A_1_/A_2_ (**7a** + **7b**), Figure S48: ^1^H-^13^C HSQC NMR spectrum (DMSO-d_6_, 500 MHz) of isochromophilonol A_1_/A_2_ (**7a** + **7b**), Figure S49: ^1^H-^13^C HMBC NMR spectrum (DMSO-d_6_, 500 MHz) of isochromophilonol A_1_/A_2_ (**7a** + **7b**), Figure S50: ^1^H-^1^H ROESY NMR spectrum (DMSO-d_6_, 500 MHz) of isochromophilonol A_1_/A_2_ (**7a** + **7b**), Figure S51: ^1^H NMR spectrum (CDCl_3_, 500 MHz) of isochromophilonol A_1_/A_2_ (**7a** + **7b**), Figure S52: UV-Vis spectrum (MeOH) of isochromophilonol A_1_/A_2_ (**7a** + **7b**), Figure S53: ECD spectrum (MeOH) of isochromophilonol A_1_/A_2_ (**7**), Figure S54: HPLC-DAD chromatogram and ESI-MS data for diaporisoindole A/B (**5**), Figure S55: HPLC-DAD chromatogram and HR-ESI (+) MS data for diaporisoindole A/B (**5**), Figure S56: ^1^H NMR spectrum (CDCl_3_, 700 MHz) of diaporisoindole A/B (**5**), Figure S57: ^1^H-^1^H COSY NMR spectrum (CDCl_3_, 700 MHz) of diaporisoindole A/B (**5**), Figure S58: ^1^H-^13^C HSQC NMR spectrum (CDCl_3_, 700 MHz) of diaporisoindole A/B (**5**), Figure S59: ^1^H-^13^C HMBC NMR spectrum (CDCl_3_, 700 MHz) of diaporisoindole A/B (**5**), Figure S60: HPLC-DAD chromatogram and ESI-MS data for tenellone B (**6**), Figure S61: HPLC-DAD chromatogram and HR-ESI (+) MS data for tenellone B (**6**), Figure S62: ^1^H NMR spectrum (DMSO-d_6_, 700 MHz) of tenellone B (**6**), Figure S63: ^1^H-^1^H COSY NMR spectrum (DMSO-d_6_, 700 MHz) of tenellone B (**6**), Figure S64: ^1^H-^13^C HSQC NMR spectrum (DMSO-d_6_, 700 MHz) of tenellone B (**6**), Figure S65: ^1^H-^13^C HMBC NMR spectrum (DMSO-d_6_, 700 MHz) of tenellone B (**6**), Figure S66: HPLC-DAD chromatogram and ESI-MS data for beauvericin (**8**), Figure S67: HPLC-DAD chromatogram and HR-ESI (+) MS data for beauvericin (**8**), Figure S68: ^1^H NMR spectrum (DMSO-d_6_, 700 MHz) of beauvericin (**8**), Figure S69: ^1^H-^1^H COSY NMR spectrum (DMSO-d_6_, 700 MHz) of beauvericin (**8**), Figure S70: ^1^H-^13^C HSQC NMR spectrum (DMSO-d_6_, 700 MHz) of beauvericin (**8**), Figure S71: ^1^H-^13^C HMBC NMR spectrum (DMSO-d_6_, 700 MHz) of beauvericin (**8**), Figure S72: HPLC-DAD chromatograms of eucalactam B derived D-FDVA (**D**)/authentic amino acid derived D-FDVA. (**A**) _DL_-threonine, (**B**) _L_-Allothreonine, (**C**) _L_-threonine, Figure S73: HPLC-DAD chromatograms of eucalactam B derived _L_-FDVA (**D**)/authentic amino acid derived _L_-FDVA. (**A**) _DL_-threonine, (**B**) _L_-Allothreonine, (**C**) _L_-threonine, Figure S74: General Marfey’s reaction with threonine, Figure S75: RAxML phylogram including our strains and type and reference strains of *Diaporthe* spp., Figure S76: Flow chart of the purification procedure, Table S1: Comparison of ^1^H NMR data of compound **1** with that of eucalactam B in CDCl_3_, Table S2: Comparison of ^1^H NMR data of compound **7** with that of isochromophilonol in CDCl_3_, Table S3: Retention time of L or D authentic amino acid derived _D_-FDVA. Table S4: Retention time of L or D authentic amino acid derived _L_-FDVA. Table S5: GenBank accession numbers of the strains included in the broad phylogenetic study. Table S6: Selected edge-linked proportional partition substitution models subjected to IQTree2 calculated with ModelTest as implemented in IQTree using Bayesian information criterion (BIC). Table S7: Characteristics of the restricted MAFFT alignments following the first phylogenetic analysis using IQTree 2 for phylogenetic inference. Table S8: Selected unlinked partition substitution model subjected to MrBayes calculated with ModelFinder as implemented in the Phylosuite program package using Bayesian information criterion (BIC). Alignments of the ITS, *cal*, *his3*, *tef1,* and *tub2* sequences used in the phylogenetic study.
