# Supplementary material for: Unprecedented Antimicrobial and Cytotoxic Polyketides from Cultures of Diaporthe africana sp. nov
Source: J Fungi (Basel). 2023 Jul 24;9(7):781. doi: 10.3390/jof9070781 (PMC10381184; doi:10.3390/jof9070781)
Supplement: Supplementary file 1 [file jof-09-00781-s001.zip › jof-2454011-supplementary.pdf]

Supplementary information

Unprecedented antimicrobial and cytotoxic polyketides from cultures of *Diaporthe africana* sp. nov

Blondelle Matio Kemkuignou [1,2], Lambert Christopher [1,3], Marc Stadler [1,2], Simeon Kouam Fogue [4] and Yasmina Marin-Felix [1,2,*]

[1] Department of Microbial Drugs, Helmholtz Centre for Infection Research (HZI) and German Centre for Infection Research (DZIF), Partner Site Hannover/Braunschweig, Inhoffenstrasse 7, 38124 Braunschweig, Germany; blondelle.matiokemkuignou@helmholtz-hzi.de; marc.stadler@helmholtz-hzi.de

[2] Institute of Microbiology, Technische Universität Braunschweig, Spielmannstraße 7, 38106 Braunschweig, Germany

[3] Department of Cell Biology, Helmholtz Centre for Infection Research (HZI), Inhoffenstrasse 7, 38124 Braunschweig, Germany; christopher.lambert@helmholtz-hzi.de

[4] Department of Chemistry, Higher Teacher Training College, University of Yaoundé I, Yaoundé P.O. Box 47, Cameroon; kfogue@yahoo.com

* Correspondence: yasmina.marinfelix@helmholtz-hzi.de; Tel.: +49 53161814267

Contents

Figure S1: HPLC-DAD chromatogram and ESI-MS data for eucalactam B (**1**)... 5

Figure S2: HPLC-DAD chromatogram and HR-ESI (+) MS data for eucalactam B (**1**)................................ 6

Figure S3: ^{1}H NMR spectrum (DMSO-d_6, 700 MHz) of eucalactam B (**1**).. 6

Figure S4: ^{13}C NMR spectrum (DMSO-d_6, 175 MHz) of eucalactam B (**1**)... 7

Figure S5: ^{1}H-^{1}H COSY NMR spectrum (DMSO-d_6, 700 MHz) of eucalactam B (**1**) 7

Figure S6: ^{1}H-^{13}C HSQC NMR spectrum (DMSO-d_6, 700 MHz) of eucalactam B (**1**)...................................... 8

Figure S7: ^{1}H-^{13}C HMBC NMR spectrum (DMSO-d_6, 700 MHz) of eucalactam B (**1**)...................................... 8

Figure S8: ^{1}H-^{1}H ROESY NMR spectrum (DMSO-d_6, 700 MHz) of eucalactam B (**1**) 9

Figure S9: ^{1}H NMR spectrum ($CDCl_3$, 500 MHz) of eucalactam B (**1**)...9

Table S1: Comparison of ^{1}H NMR data of compound **1** with that of eucalactam B in $CDCl_3$..........................9

Figure S10: UV-Vis spectrum (MeOH) of eucalactam B (**1**).. 11

Figure S11: ECD spectrum (MeOH) of eucalactam B (**1**).. 11

Figure S12: HPLC-DAD chromatogram and ESI-MS data for isoprenylisobenzofuran B (**2**) 12

Figure S13: HPLC-DAD chromatogram and HR-ESI (+) MS data for isoprenylisobenzofuran B (**2**)............. 12

Figure S14: ^{1}H NMR spectrum ($CDCl_3$ + 1%TFA, 700 MHz) of isoprenylisobenzofuran B (**2**)..................... 13

Figure S15: ^{13}C NMR spectrum ($CDCl_3$ + 1%TFA, 175 MHz) of isoprenylisobenzofuran B (**2**).................... 13

Figure S16: 1H-1H COSY NMR spectrum ($CDCl_3$ + 1%TFA, 700 MHz) of isoprenylisobenzofuran B (**2**)..... 14

Figure S17: ^{1}H-^{13}C HSQC NMR spectrum ($CDCl_3$ + 1%TFA, 700 MHz) of isoprenylisobenzofuran B (**2**)...... 14

Figure S18: ^{1}H-^{13}C HMBC NMR spectrum ($CDCl_3$ + 1%TFA, 700 MHz) of isoprenylisobenzofuran B (**2**)..... 15

Figure S19: ^{1}H-^{1}H ROESY NMR spectrum ($CDCl_3$ + 1%TFA, 700 MHz) of isoprenylisobenzofuran B (**2**) 16

Figure S20: ^{1}H NMR spectrum (MeOH-d_4, 500 MHz) of compound **2**...16

Figure S21: ^{13}C NMR spectrum (MeOH-d_4, 125 MHz) of compound **2**..16

Figure S22: ^{1}H-^{13}C HMBC NMR spectrum (MeOH-d_4, 500 MHz) of compound **2**.......................................17

Figure S23: UV-Vis spectrum (MeOH) of isoprenylisobenzofuran B (**2**) 17

Figure S24: ECD spectrum (MeOH) of isoprenylisobenzofuran B (**2**) 18

Figure S25: HPLC-DAD chromatogram and ESI-MS data for isoprenylisobenzofuran C_1/C_2 (**3a** + **3b**) 19

Figure S26: HPLC-DAD chromatogram and (+)/(-) HR-ESIMS data for isoprenylisobenzofuran C_1/C_2 (**3a** + **3b**) 19

Figure S27: ^{1}H NMR spectrum (DMSO-d_6, 700 MHz) of isoprenylisobenzofuran C_1/C_2 (**3a** + **3b**) 20

Figure S28: ^{13}C NMR spectrum (DMSO-d_6, 175 MHz) of isoprenylisobenzofuran C_1/C_2 (**3a** + **3b**) 20

Figure S29: ^{1}H-^{1}H COSY NMR spectrum (DMSO-d_6, 700 MHz) of isoprenylisobenzofuran C_1/C_2 (**3a** + **3b**) 21

Figure S30: ^{1}H-^{13}C HSQC NMR spectrum (DMSO-d_6, 700 MHz) of d isoprenylisobenzofuran C_1/C_2 (**3a** + **3b**) 22

Figure S31: ^{1}H-^{13}C HMBC NMR spectrum (DMSO-d$_6$, 700 MHz) of isoprenylisobenzofuran C_1/C_2 (**3a** + **3b**) 23

Figure S32: ^{1}H-^{1}H ROESY NMR spectrum (DMSO-d$_6$, 700 MHz) of isoprenylisobenzofuran C_1/C_2 (**3a** + **3b**) 24

Figure S33: UV-Vis spectrum (MeOH) of isoprenylisobenzofuran C_1/C_2 (**3a** + **3b**) 24

Figure S34: HPLC-DAD chromatogram and ESI-MS data for diaporisoindole F_1/F_2 (**4a** + **4b**) 25

Figure S35: HPLC-DAD chromatogram and HR-ESI (+) MS data for diaporisoindole F1/F2 (**4a** + **4b**) 26

Figure S36: ^{1}H NMR spectrum (DMSO-d$_6$, 700 MHz) of diaporisoindole F_1/F_2 (**4a** + **4b**) 26

Figure S37: ^{13}C NMR spectrum (DMSO-d$_6$, 175 MHz) of diaporisoindole F_1/F_2 (**4a** + **4b**) 27

Figure S38: ^{1}H-^{1}H COSY NMR spectrum (DMSO-d$_6$, 700 MHz) of diaporisoindole F_1/F_2 (**4a** + **4b**) 27

Figure S39: ^{1}H-^{13}C HSQC NMR spectrum (DMSO-d$_6$, 700 MHz) of diaporisoindole F_1/F_2 (**4a** + **4b**) 28

Figure S40: ^{1}H-^{13}C HMBC NMR spectrum (DMSO-d$_6$, 700 MHz) of diaporisoindole F_1/F_2 (**4a** + **4b**) 28

Figure S41: ^{1}H-^{1}H ROESY NMR spectrum (DMSO-d$_6$, 700 MHz) of diaporisoindole F_1/F_2 (**4a** + **4b**) 29

Figure S42: UV-Vis spectrum (MeOH) of diaporisoindole F_1/F_2 (**4a** + **4b**) 30

Figure S43: HPLC-DAD chromatogram and ESI-MS data for isochromophilonol A_1/A_2 (**7a** + **7b**) 31

Figure S44: HPLC-DAD chromatogram and HR-ESI (+) MS data for isochromophilonol A_1/A_2 (**7a** + **7b**) 31

Figure S45: ^{1}H NMR spectrum (DMSO-d$_6$, 500 MHz) of isochromophilonol A_1/A_2 (**7a** + **7b**) 32

Figure S46: ^{13}C NMR spectrum (DMSO-d$_6$, 125 MHz) of isochromophilonol A_1/A_2 (**7a** + **7b**) 32

Figure S47: ^{1}H-^{1}H COSY NMR spectrum (DMSO-d$_6$, 500 MHz) of isochromophilonol A_1/A_2 (**7a** + **7b**) 33

Figure S48: ^{1}H-^{13}C HSQC NMR spectrum (DMSO-d$_6$, 500 MHz) of isochromophilonol A_1/A_2 (**7a** + **7b**) 33

Figure S49: ^{1}H-^{13}C HMBC NMR spectrum (DMSO-d$_6$, 500 MHz) of isochromophilonol A_1/A_2 (**7a** + **7b**) 34

Figure S50: ^{1}H-^{1}H ROESY NMR spectrum (DMSO-d$_6$, 500 MHz) of isochromophilonol A_1/A_2 (**7a** + **7b**) 35

Figure S51: ^{1}H NMR spectrum ($CDCl_3$, 500 MHz) of isochromophilonol A_1/A_2 (**7a** + **7b**) 36

Table S2: Comparison of ^{1}H NMR data of compound **7** with that of isochromophilonol in $CDCl_3$ 36

Figure S52: UV-Vis spectrum (MeOH) of isochromophilonol A_1/A_2 (**7a** + **7b**) 37

Figure S53: ECD spectrum (MeOH) of isochromophilonol A_1/A_2 (**7a+7b**) 37

Figure S54: HPLC-DAD chromatogram and ESI-MS data for diaporisoindole A/B (**5**) 38

Figure S55: HPLC-DAD chromatogram and HR-ESI (+) MS data for diaporisoindole A/B (**5**) 39

Figure S56: ^{1}H NMR spectrum ($CDCl_3$, 700 MHz) of diaporisoindole A/B (**5**) 39

Figure S57: ^{1}H-^{1}H COSY NMR spectrum ($CDCl_3$, 700 MHz) of diaporisoindole A/B (**5**) 40

Figure S58: ^{1}H-^{13}C HSQC NMR spectrum ($CDCl_3$, 700 MHz) of diaporisoindole A/B (**5**) 40

Figure S59: ^{1}H-^{13}C HMBC NMR spectrum ($CDCl_3$, 700 MHz) of diaporisoindole A/B (**5**) 41

Figure S60: HPLC-DAD chromatogram and ESI-MS data for tenellone B (**6**) 41

Figure S61: HPLC-DAD chromatogram and HR-ESI (+) MS data for tenellone B (**6**) 42

Figure S62: ^{1}H NMR spectrum (DMSO-d$_6$, 700 MHz) of tenellone B (**6**)......42
Figure S63: ^{1}H-^{1}H COSY NMR spectrum (DMSO-d$_6$, 700 MHz) of tenellone B (**6**)......43
Figure S64: ^{1}H-^{13}C HSQC NMR spectrum (DMSO-d$_6$, 700 MHz) of tenellone B (**6**)......43
Figure S65: ^{1}H-^{13}C HMBC NMR spectrum (DMSO-d$_6$, 700 MHz) of tenellone B (**6**)......44
Figure S66: HPLC-DAD chromatogram and ESI-MS data for beauvericin (**8**)......44
Figure S67: HPLC-DAD chromatogram and HR-ESI (+) MS data for beauvericin (**8**)......45
Figure S68: ^{1}H NMR spectrum (DMSO-d$_6$, 700 MHz) of beauvericin (**8**)......45
Figure S69: ^{1}H-^{1}H COSY NMR spectrum (DMSO-d$_6$, 700 MHz) of beauvericin (**8**)......46
Figure S70: ^{1}H-^{13}C HSQC NMR spectrum (DMSO-d$_6$, 700 MHz) of beauvericin (**8**)......46
Figure S71: ^{1}H-^{13}C HMBC NMR spectrum (DMSO-d$_6$, 700 MHz) of beauvericin (**8**)......47
Figure S72: HPLC-DAD chromatograms of diaporthamicin derived $_{D}$-FDVA (**D**)/authentic amino acid derived $_{D}$-FDVA. **A**) $_{DL}$-threonine, **B**) $_{L}$- Allothreonine, **C**) $_{L}$-threonine......48
Table S3: Retention time of L or D authentic amino acid derived $_{D}$-FDVA......48
Figure S73: HPLC-DAD chromatograms of diaporthamicin derived $_{L}$-FDVA (**D**)/authentic amino acid derived $_{L}$-FDVA. **A**) $_{DL}$-threonine, **B**) $_{L}$- Allothreonine, **C**) $_{L}$-threonine......49
Table S4: Retention time of L or D authentic amino acid derived $_{L}$-FDVA......49
Figure S74: General Marfey´s reaction with threonine......49
Figure S75: RAxML phylogram including our strains and type and reference strains of *Diaporthe* spp......51
Table S5: GenBank accession numbers of the strains included in the broad phylogenetic study......52
Table S6: Selected edge-linked proportional partition substitution models subjected to IQTree2 calculated with ModelTest as implemented in IQTree using Bayesian information criterion (BIC)......68
Table S7: Characteristics of the restricted MAFFT alignments following the first phylogenetic analysis using IQTree 2.1.3 for phylogenetic inference......68
Table S8: Selected unlinked partition substitution model subjected to MrBayes calculated with ModelFinder as implemented in the Phylosuite program package using Bayesian information criterion (BIC)......68
Figure S76: Flow chart of the purification procedure......146

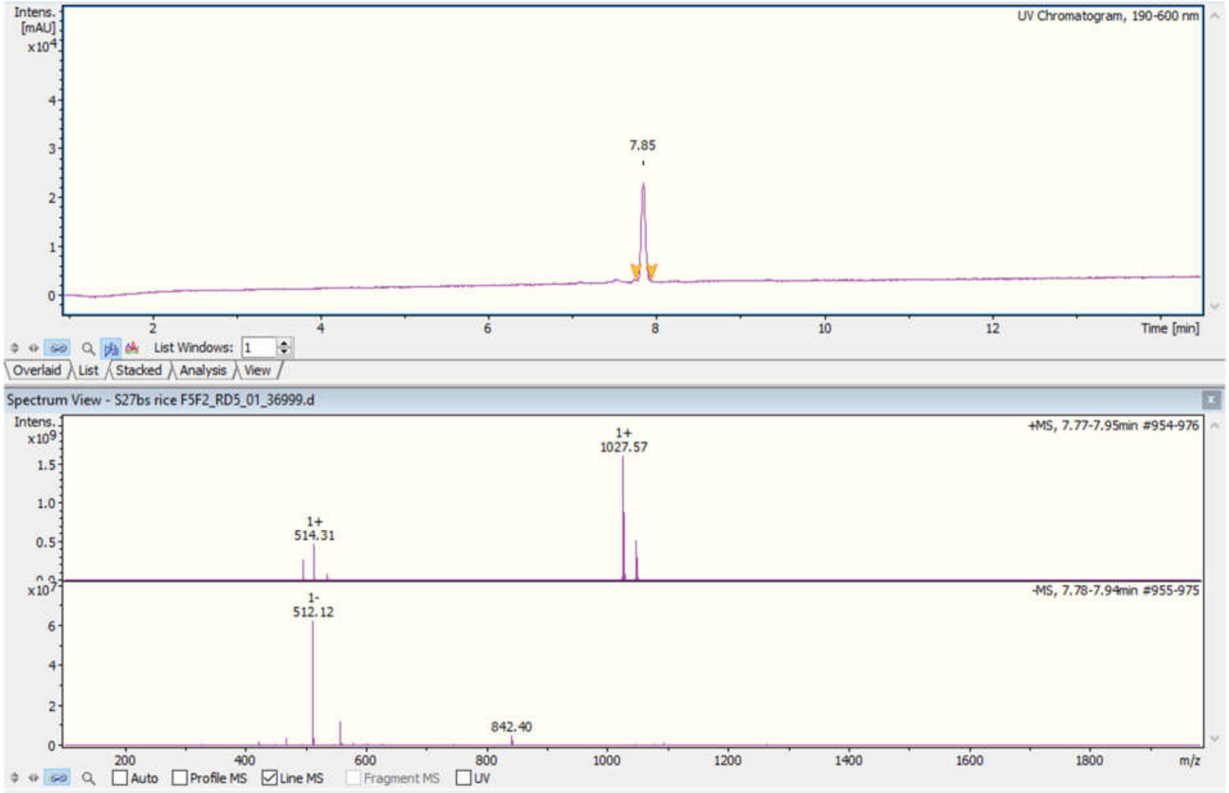

Figure S1: HPLC-DAD chromatogram and ESI-MS data for eucalactam B (**1**)

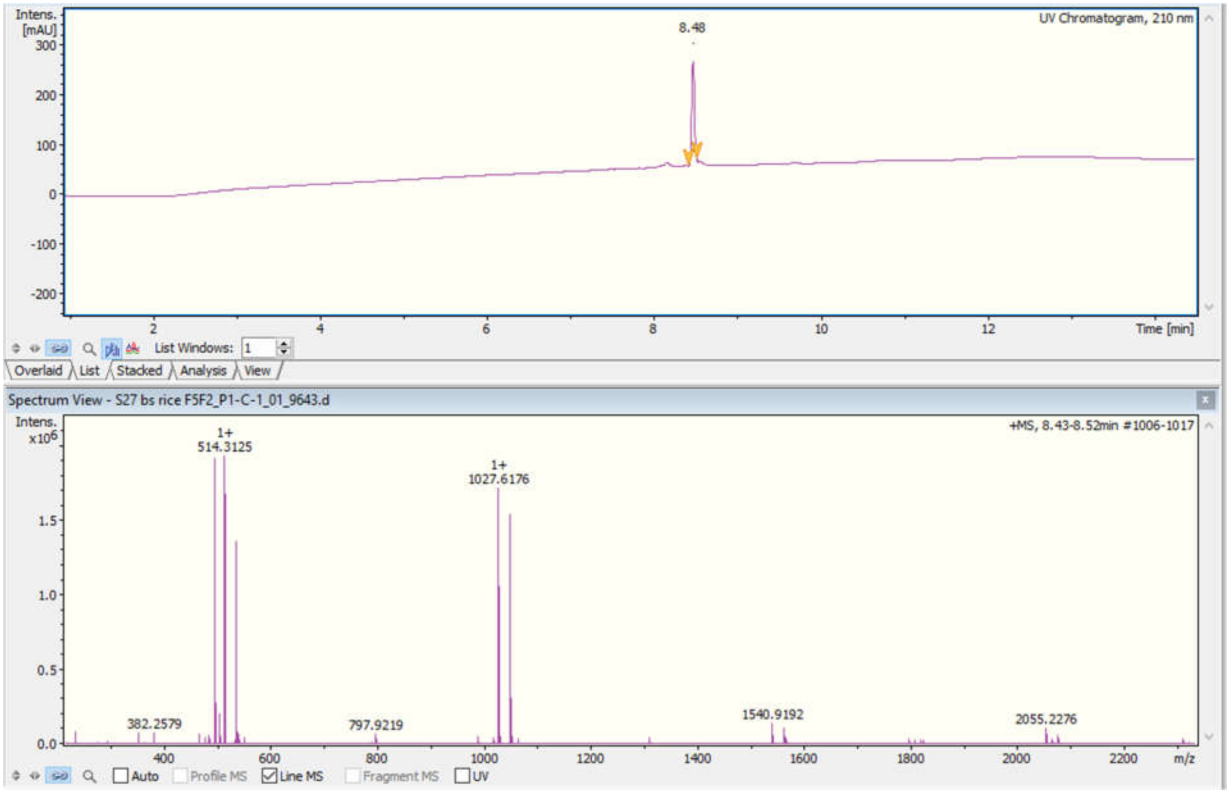

Figure S2: HPLC-DAD chromatogram and HR-ESI (+) MS data for eucalactam B (**1**)

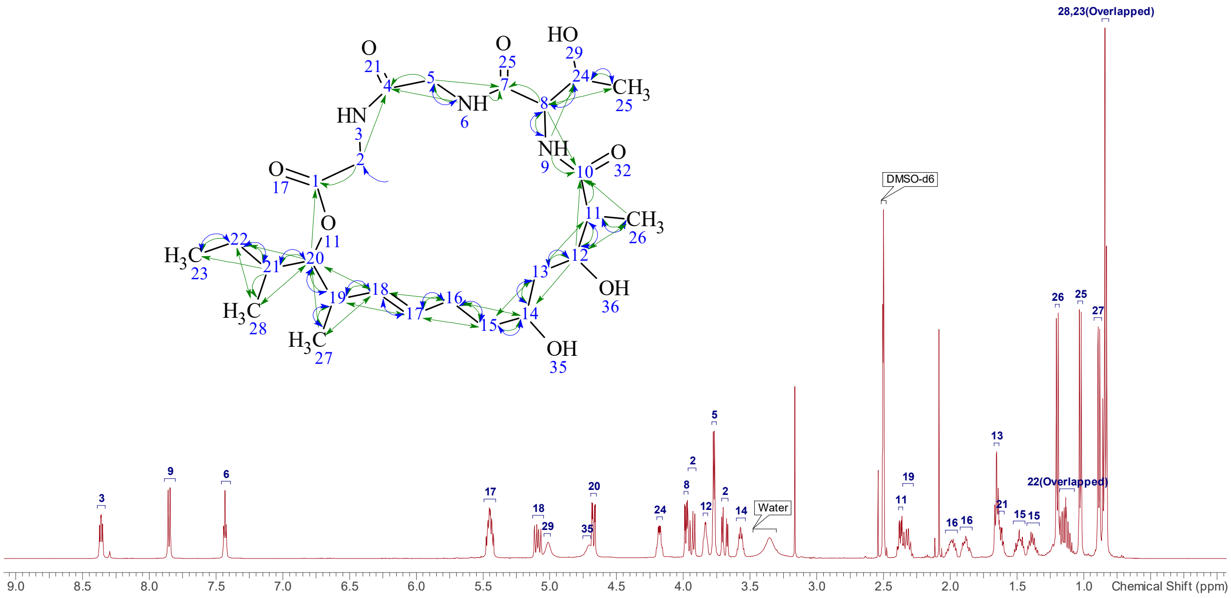

Figure S3: ^{1}H NMR spectrum (DMSO-d$_6$, 700 MHz) of eucalactam B (**1**)

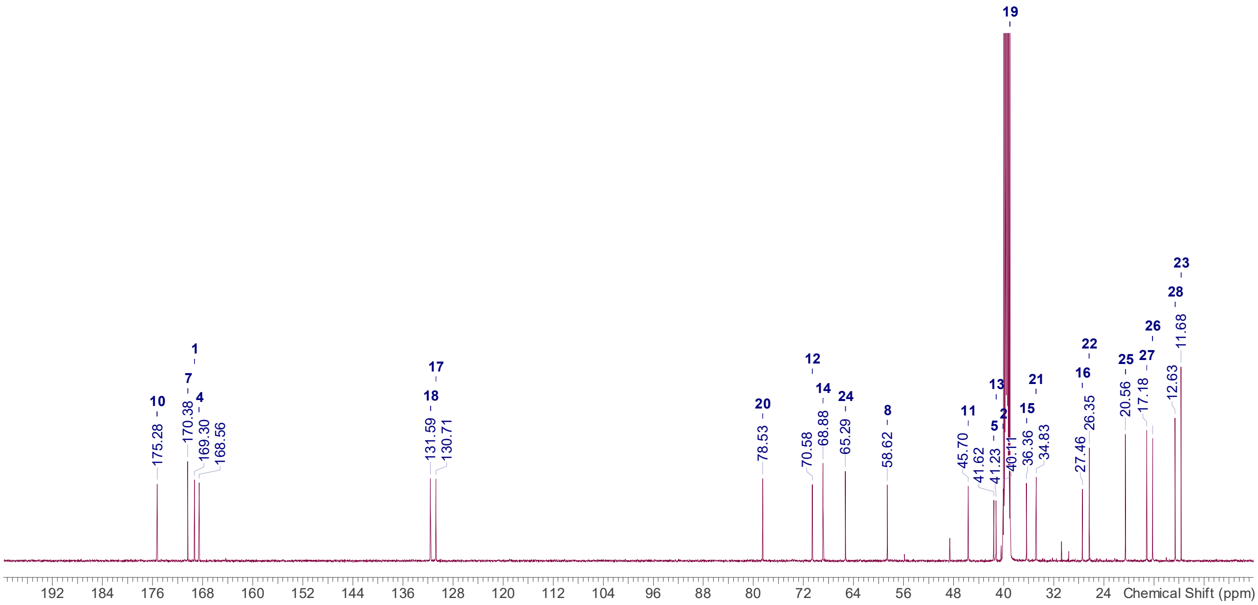

Figure S4: ^{13}C NMR spectrum (DMSO-d$_6$, 175 MHz) of eucalactam B (**1**)

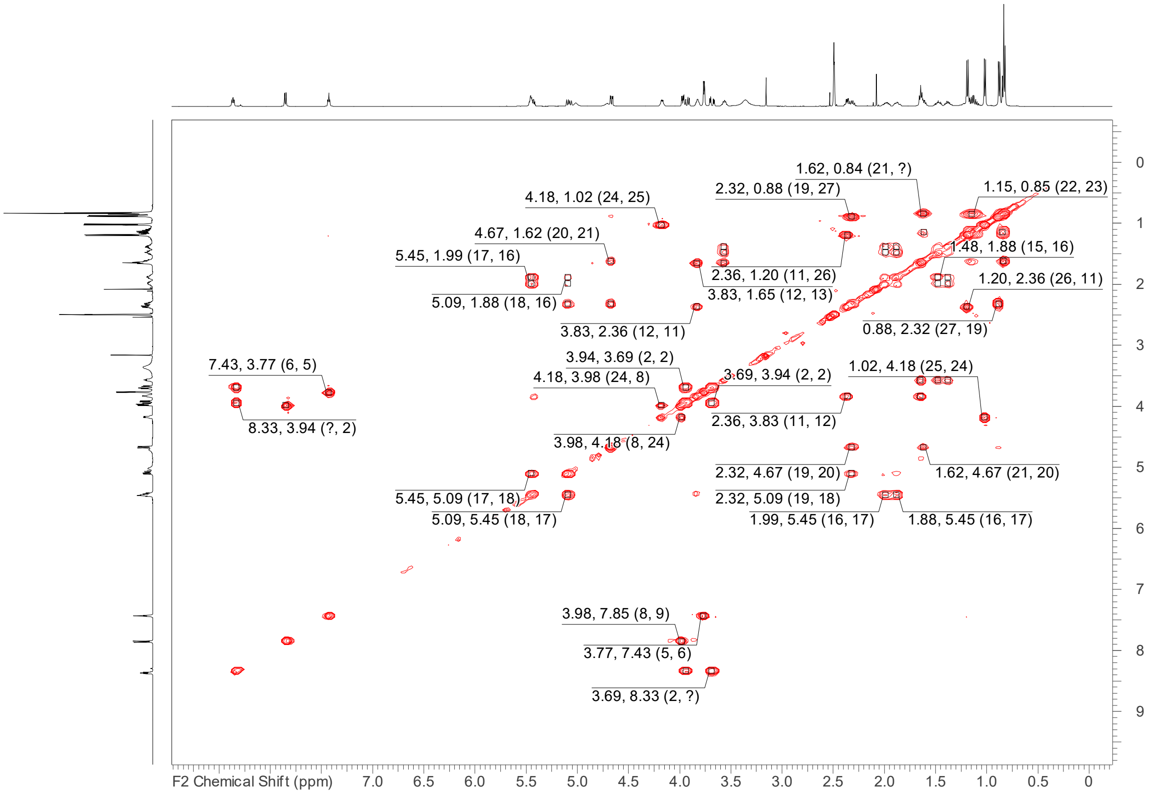

Figure S5: ^{1}H-^{1}H COSY NMR spectrum (DMSO-d$_6$, 700 MHz) of eucalactam B (**1**)

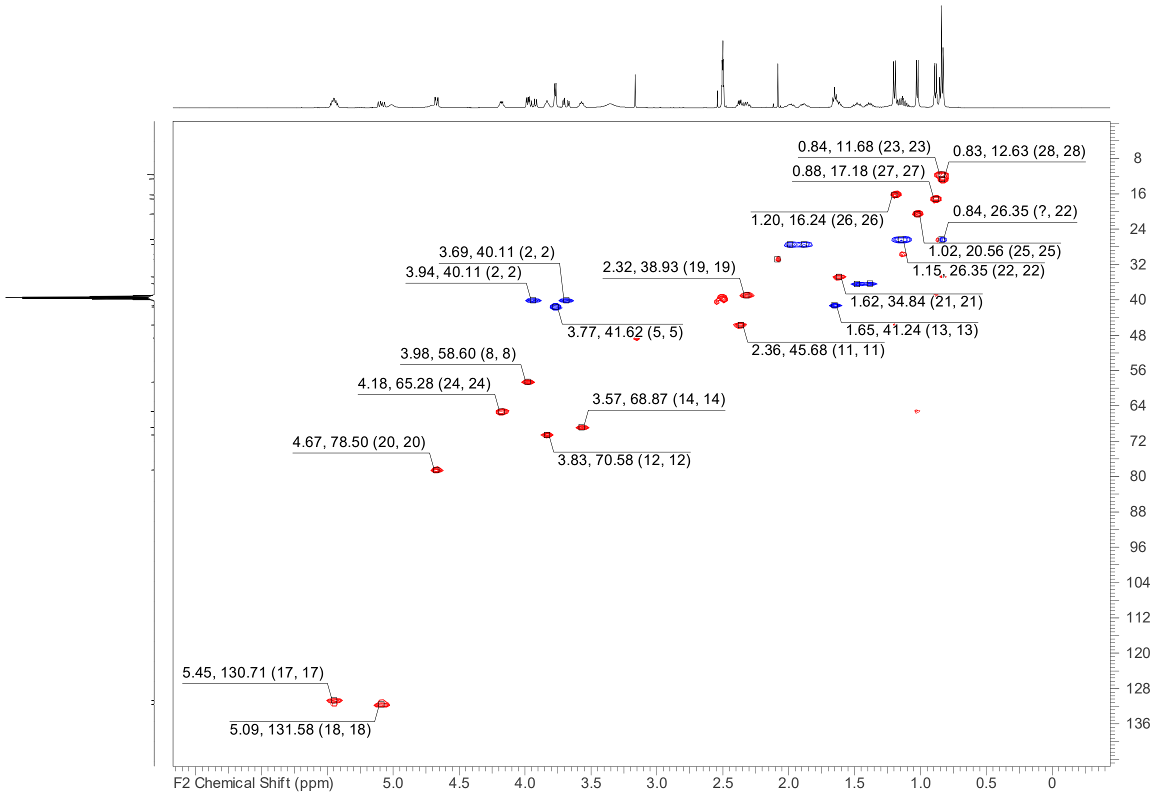

Figure S6: ^{1}H-^{13}C HSQC NMR spectrum (DMSO-d$_6$, 700 MHz) of eucalactam B (**1**)

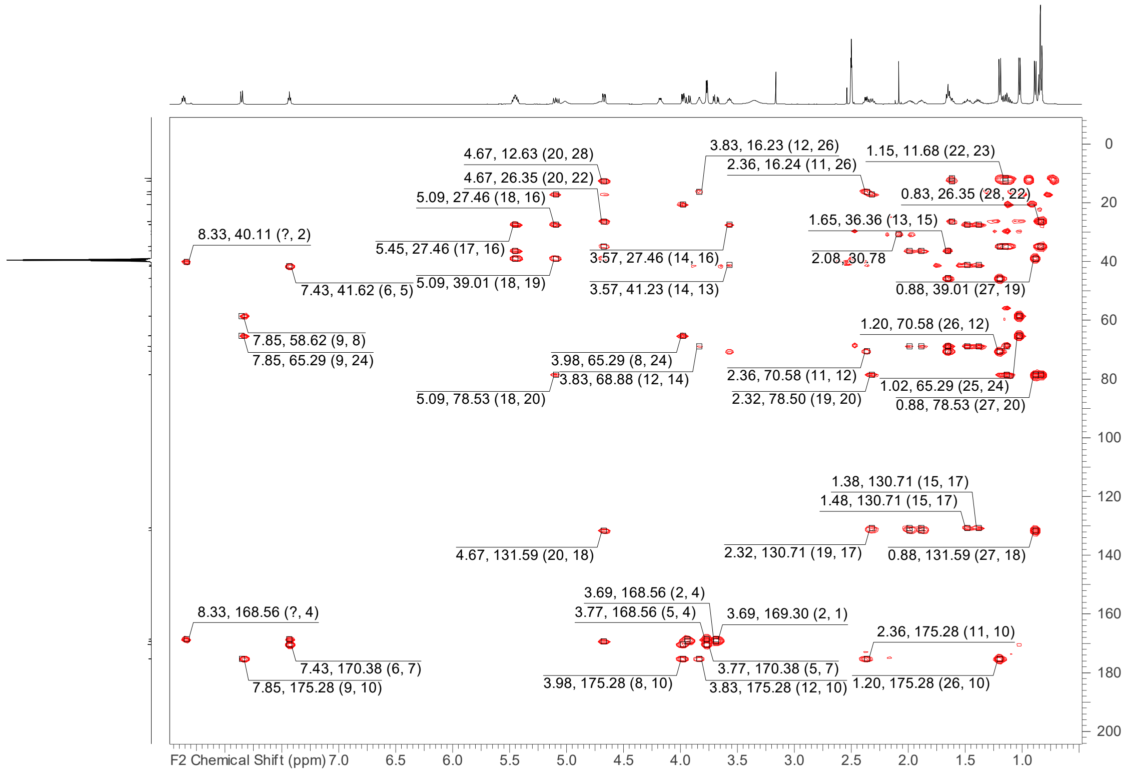

Figure S7: ^{1}H-^{13}C HMBC NMR spectrum (DMSO-d$_6$, 700 MHz) of eucalactam B (**1**)

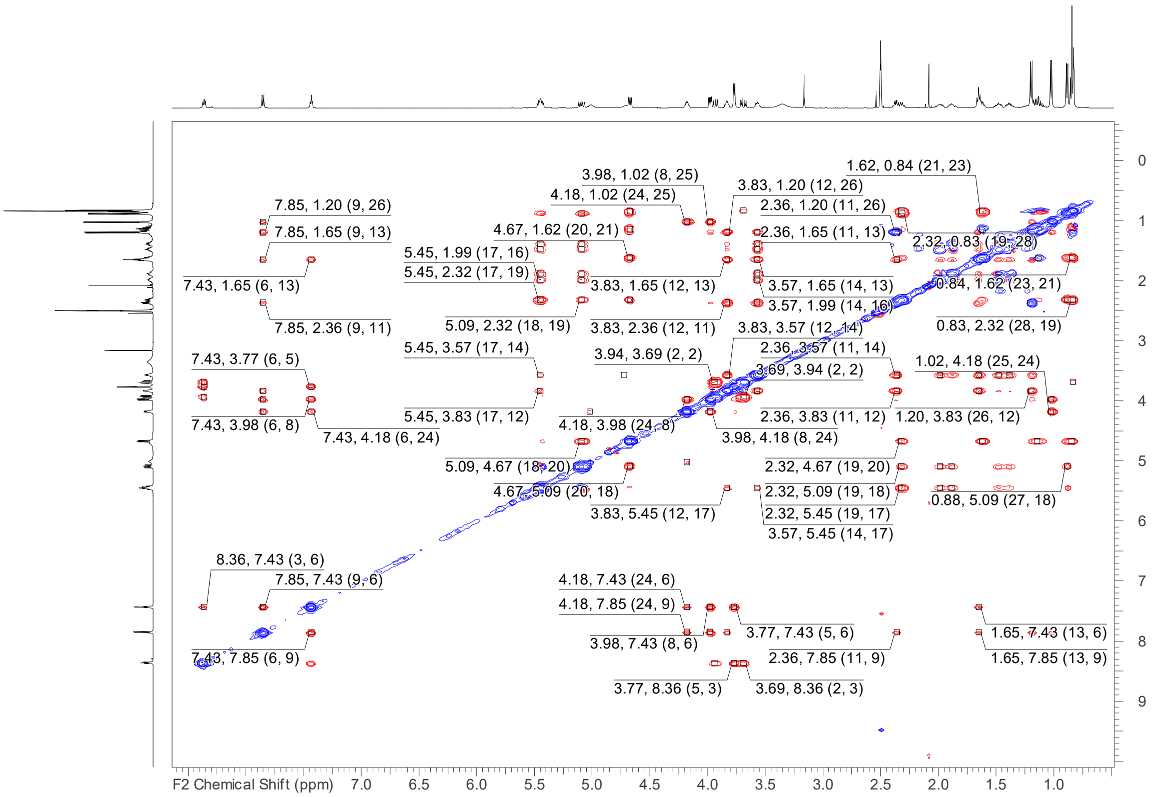

Figure S8: ^{1}H-^{1}H ROESY NMR spectrum (DMSO-d_6, 700 MHz) of eucalactam B (**1**)

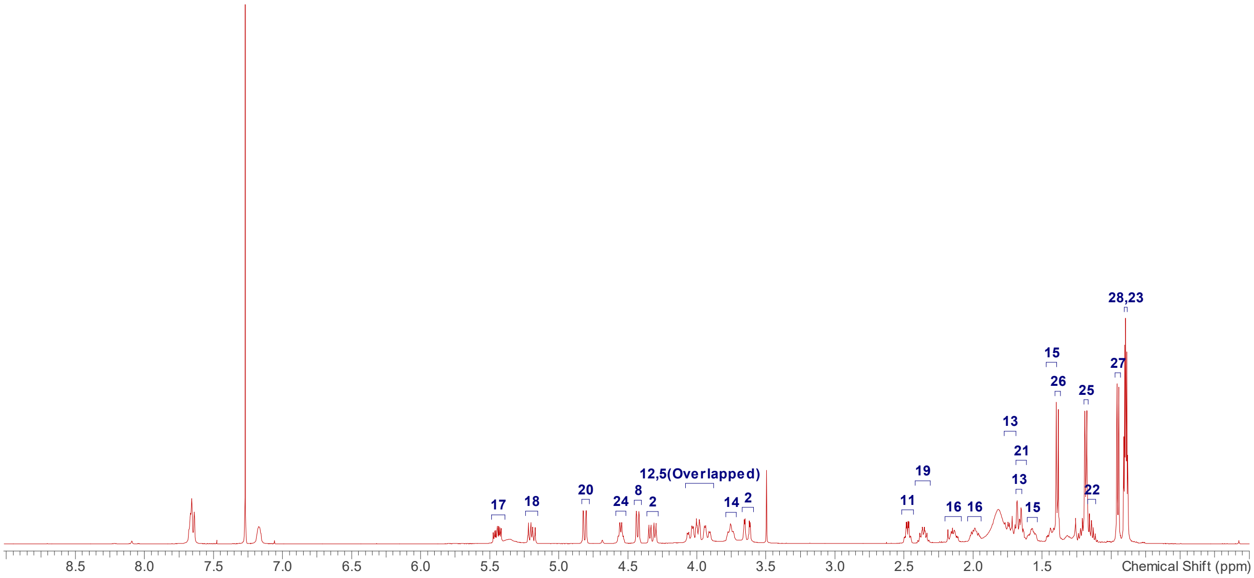

Figure S9: ^{1}H NMR spectrum ($CDCl_3$, 500 MHz) of eucalactam B (**1**)

Table S1: Comparison of ^{1}H NMR data of compound **1** with that of eucalactam B in $CDCl_3$

		Compound 1	Eucalactam B
	No.	δ_H (*J* in Hz)	δ_H (*J* in Hz)
Fragment A	1	-	

	2	3.64, dd (18.4, 3.4)	3.62, dd (18.3, 3.6)
		4.32, dd (18.4, 7.3)	4.33, dd (18.3, 7.2)
	3-NH	7.65*	7.45, dd (7.2, 3.6)
	4	-	-
	5	3.98, m (overlapped)	3.99, m (overlapped)
	6-NH	7.65*	7.64, t (4.7)
Fragment B	7	-	-
	8	4.43, br d (8.9)	4.42, br d (9.1)
	9-NH	7.17*	7.72, d (6.3)
	24	4.55, q (6.0)	4.54, qd (6.3, 0.9)
	25	1.18, d (6.0)	1.18, d (6.3)
Fragment C	10	-	-
	11	2.47, qd (7.3, 2.8)	2.46, dq (7.3, 2.7)
	12	3.99, m (overlapped)	3.99, m (overlapped)
	13	1.66, m (overlapped)	1.65, m (overlapped)
		1.75, m	1.77, dt (14.7, 10.6)
	14	3.76, m	3.73, m
	15	1.43, m	1.46, m
		1.57, m	1.56, m
	16	1.99, m	1.98, m
		2.15, m	2.14, m
	17	5.44, m	5.44, m
	18	5.2, dd (15.2, 9.1)	5.18, dd (15.3, 9.1)
	19	2.36, m	2.35, m
	20	4.81, dd (10.4, 1.8)	4.79, dd (10.4, 1.6)
	21	1.66, m (overlapped)	1.65, m (overlapped)
	22	1.14, m	1.13, m
	23	0.89, t (7.1)	0.87, t (6.9)
	26	1.39, d (7.3)	1.39 (d, 7.6)
	27	0.95, d (6.7)	0.94, d (6.6)
	28	0.89, d (7.1)	0.89, d (6.0)

* Broad signals (could not be assigned properly and might be interchangeable)

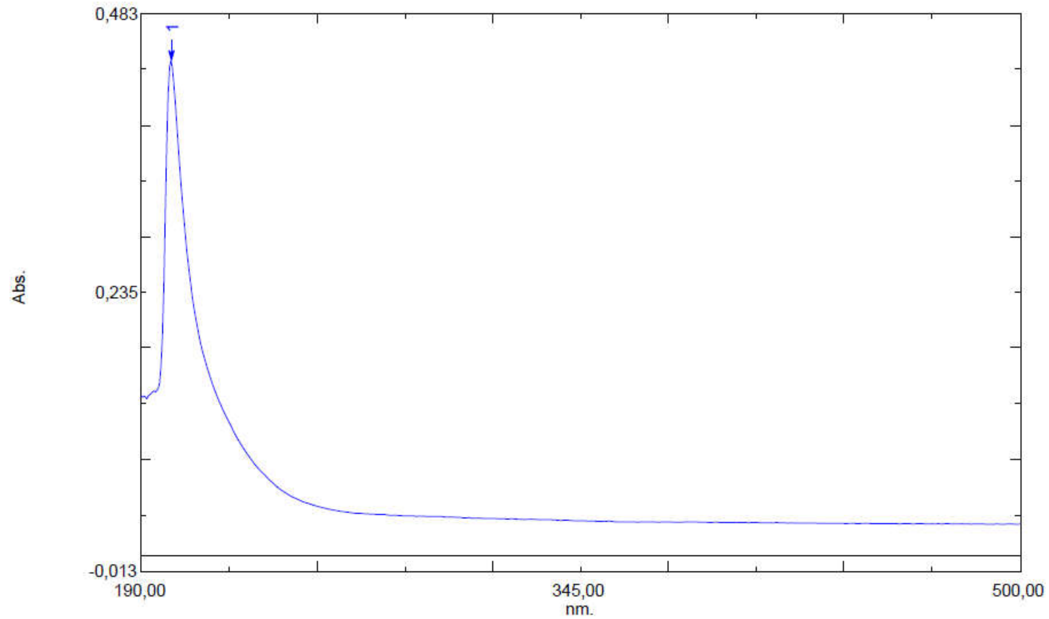

Measurement Properties

Wavelength Range (nm.):	190,00 to 500,00
Scan Speed:	Medium
Sampling Interval:	0,5
Auto Sampling Interval:	Disabled
Scan Mode:	Single

No.	P/V	Wavelength	Abs.	Description
1		200,50	0,442	

Figure S10: UV-Vis spectrum (MeOH) of eucalactam B (**1**)

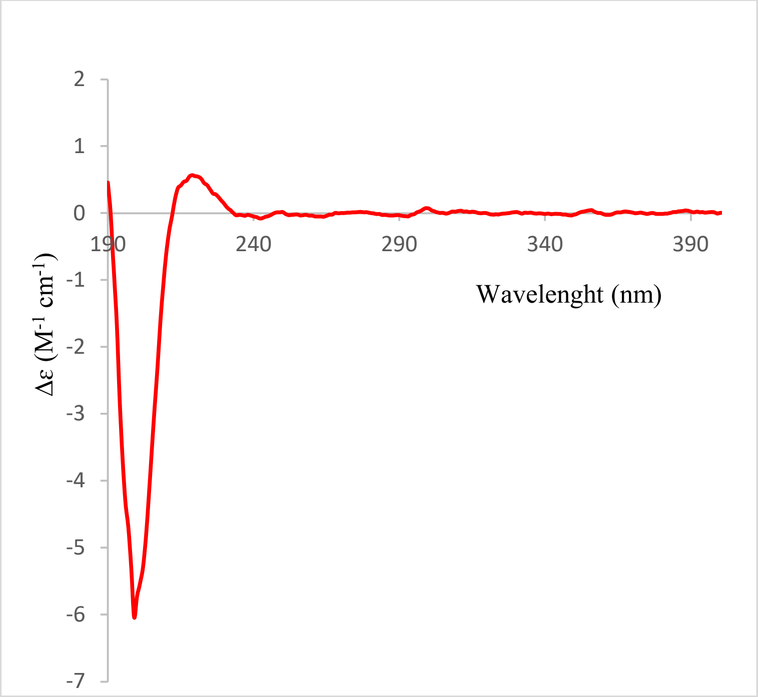

Figure S11: ECD spectrum (MeOH) of eucalactam B (**1**)

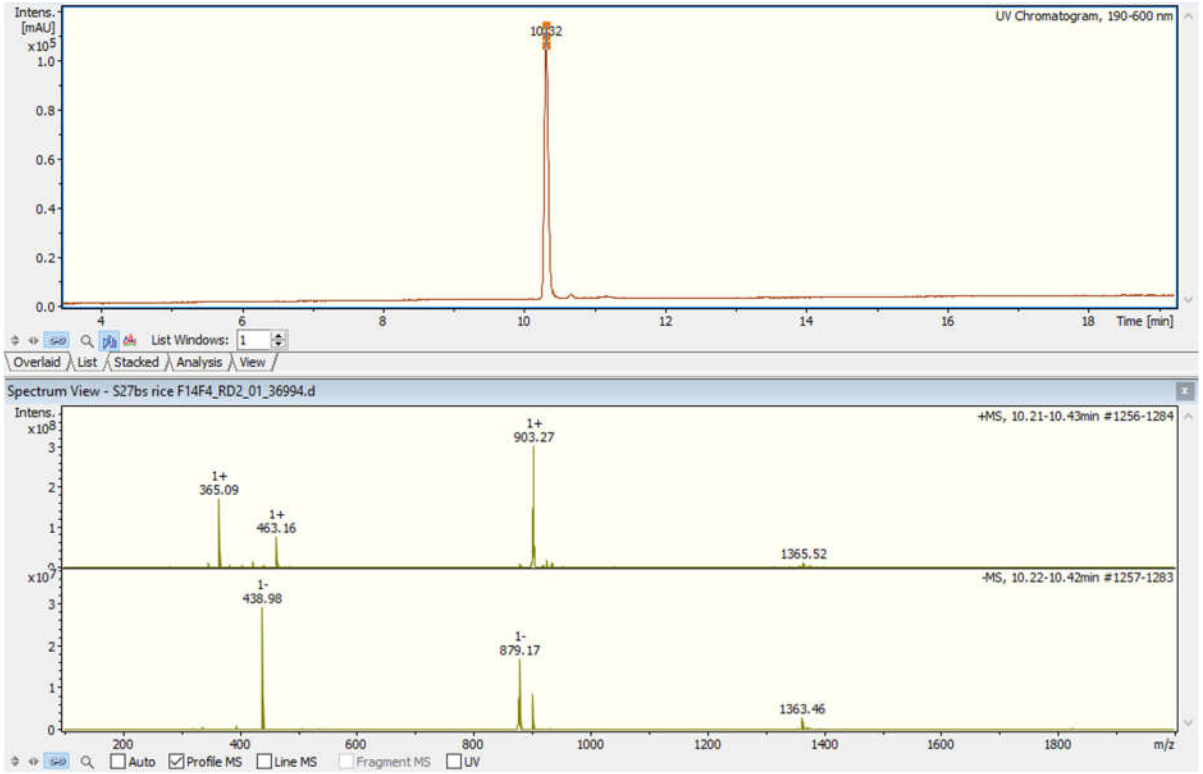

Figure S12: HPLC-DAD chromatogram and ESI-MS data for isoprenylisobenzofuran B (**2**)

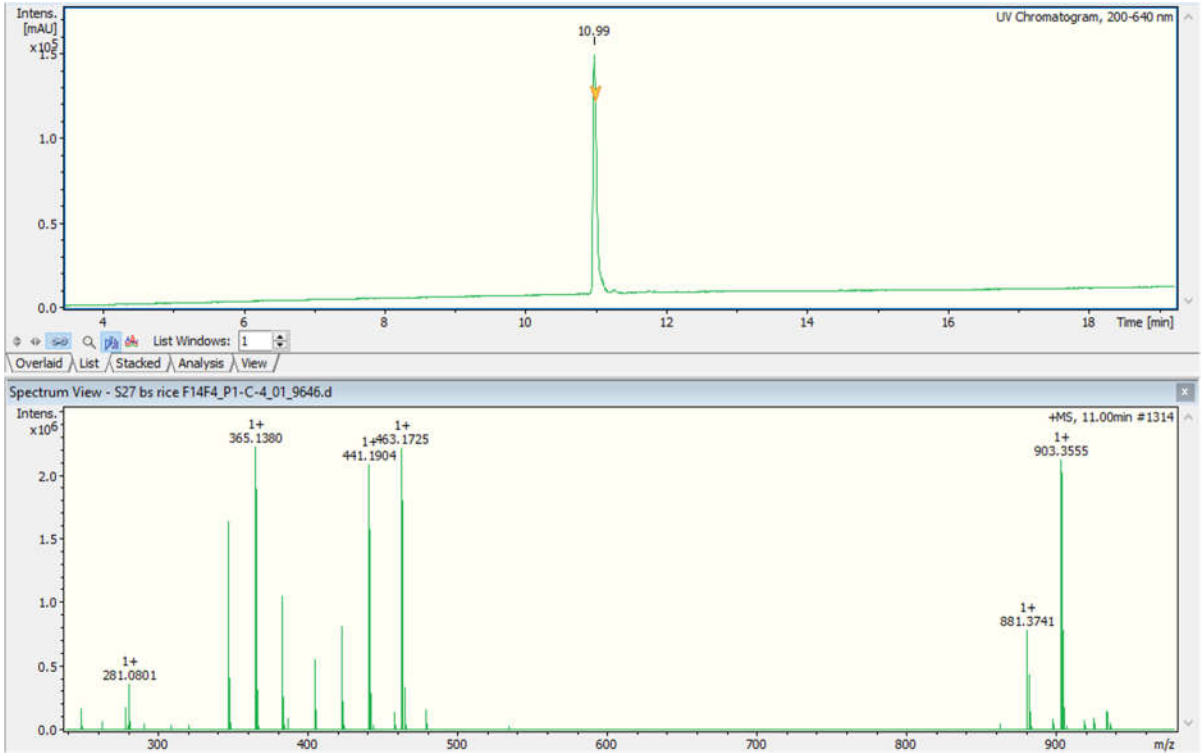

Figure S13: HPLC-DAD chromatogram and HR-ESI (+) MS data for isoprenylisobenzofuran B (**2**)

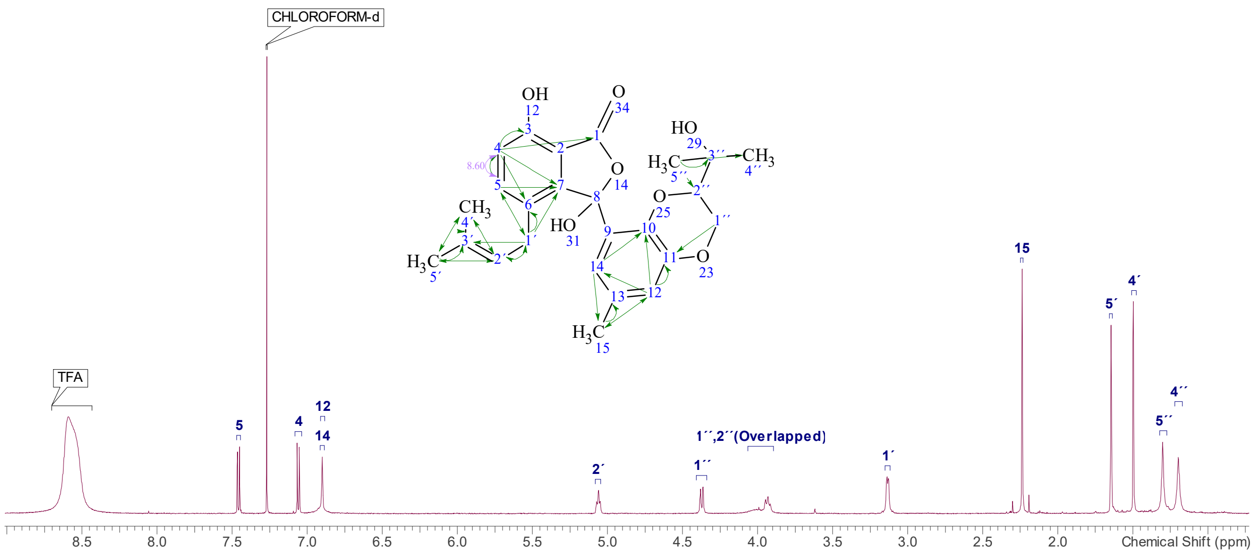

Figure S14: ^{1}H NMR spectrum ($CDCl_3$ + 1%TFA, 700 MHz) of isoprenylisobenzofuran B (**2**)

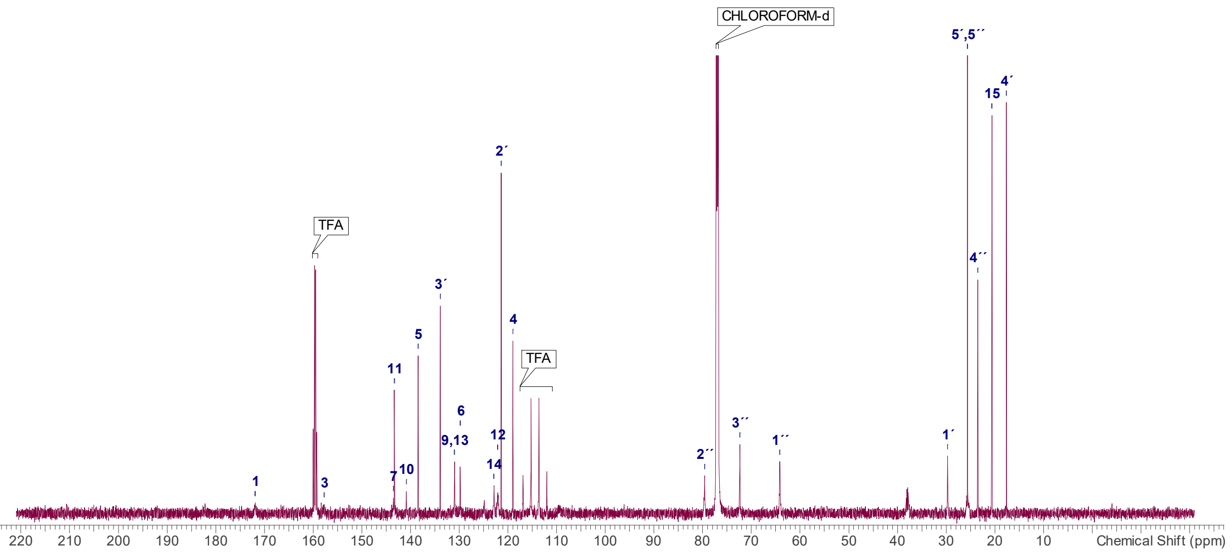

Figure S15: ^{13}C NMR spectrum ($CDCl_3$ + 1%TFA, 175 MHz) of isoprenylisobenzofuran B (**2**)

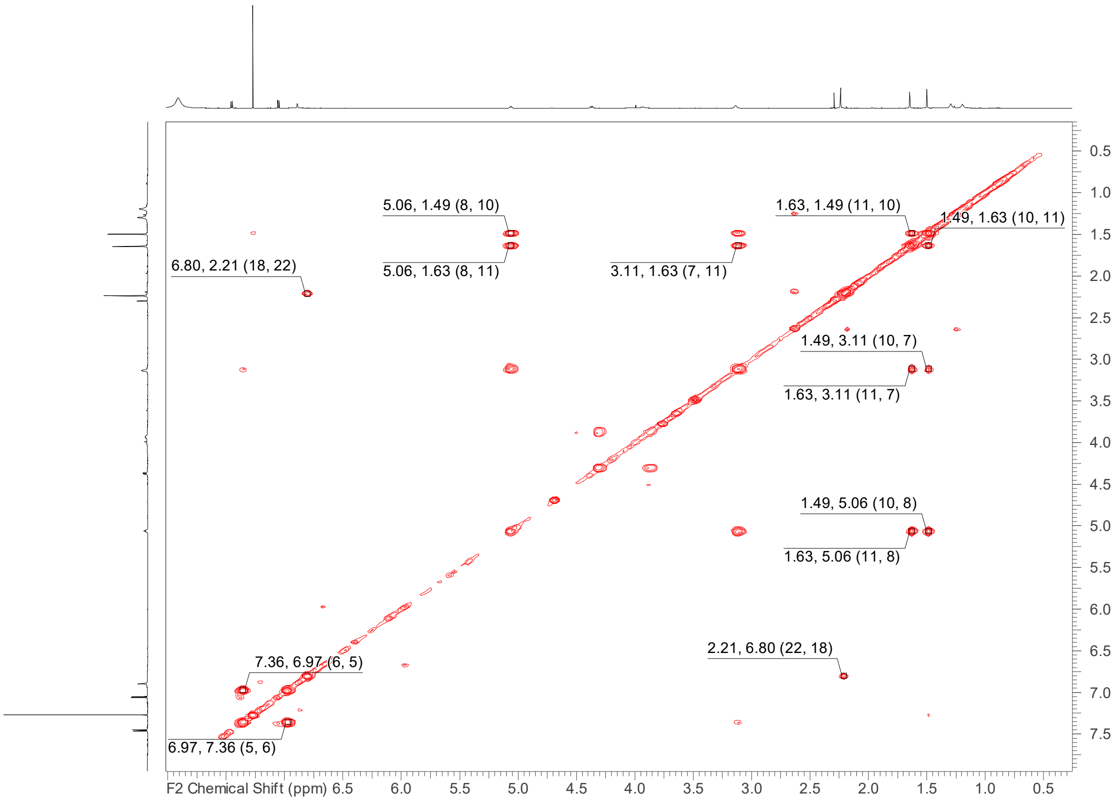

Figure S16: ^{1}H-^{1}H COSY NMR spectrum ($CDCl_3$ + 1%TFA, 700 MHz) of isoprenylisobenzofuran B (**2**)

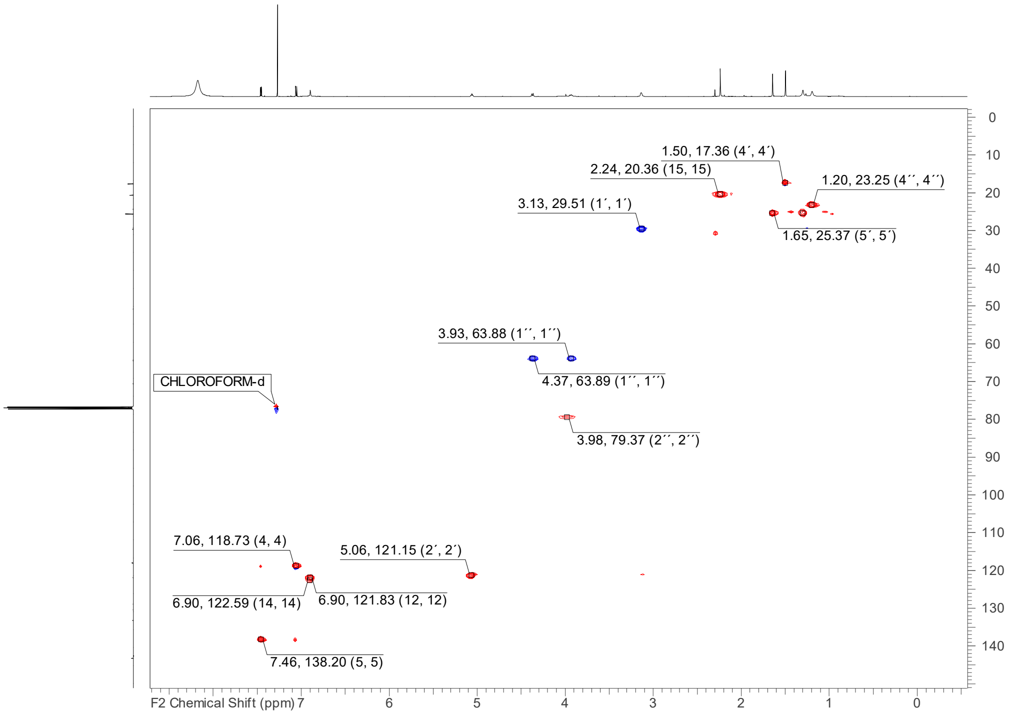

Figure S17: ^{1}H-^{13}C HSQC NMR spectrum ($CDCl_3$ + 1%TFA, 700 MHz) of isoprenylisobenzofuran B (**2**)

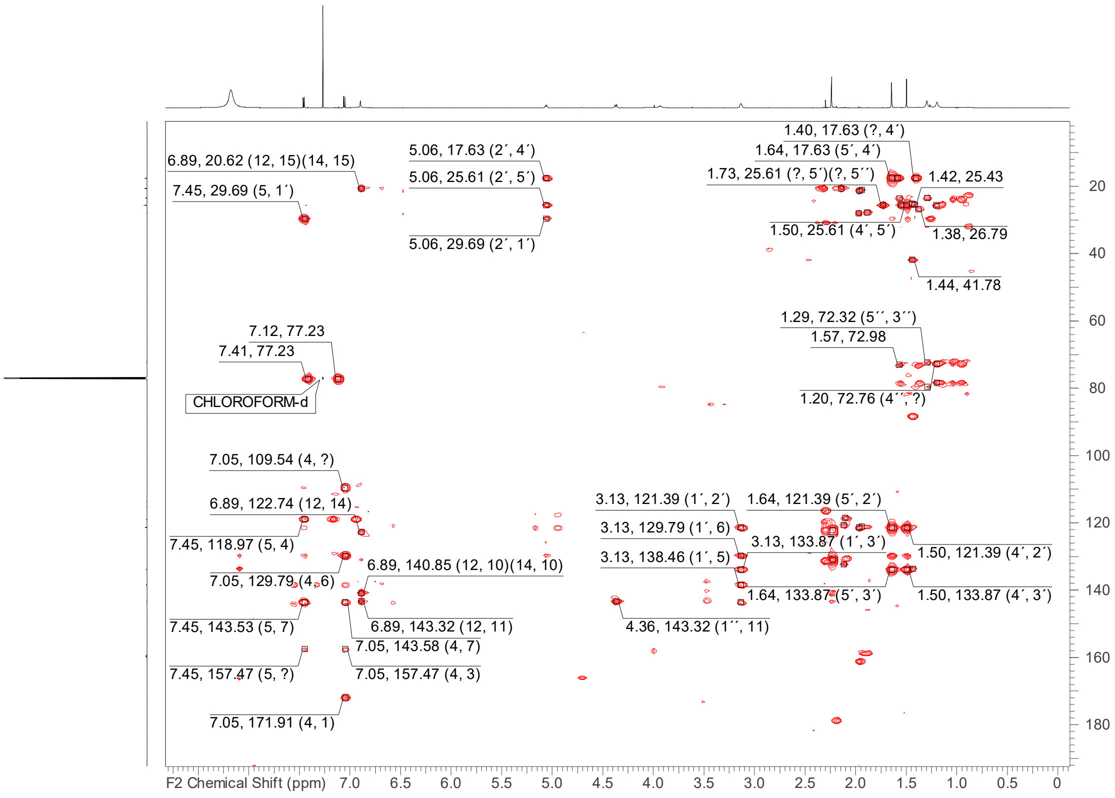

Figure S18: ^{1}H-^{13}C HMBC NMR spectrum ($CDCl_3$ + 1%TFA, 700 MHz) of isoprenylisobenzofuran B (**2**)

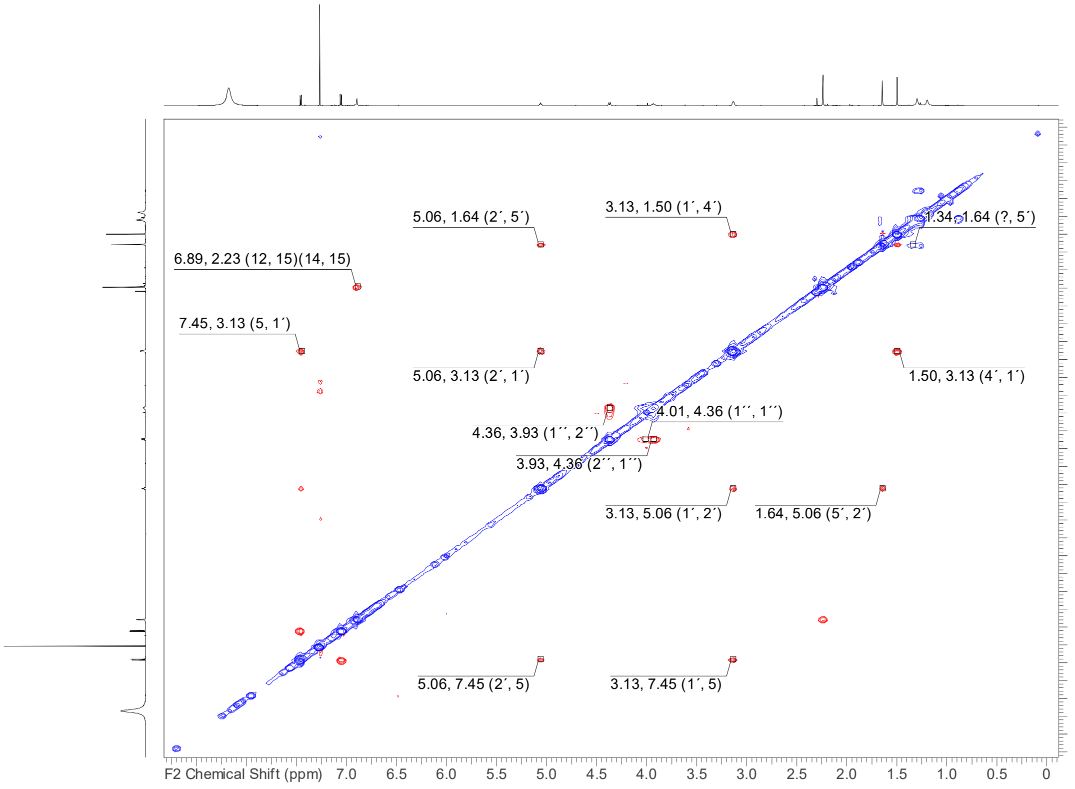

Figure S19: ^{1}H-^{1}H ROESY NMR spectrum ($CDCl_3$ + 1%TFA, 700 MHz) of isoprenylisobenzofuran B (**2**)

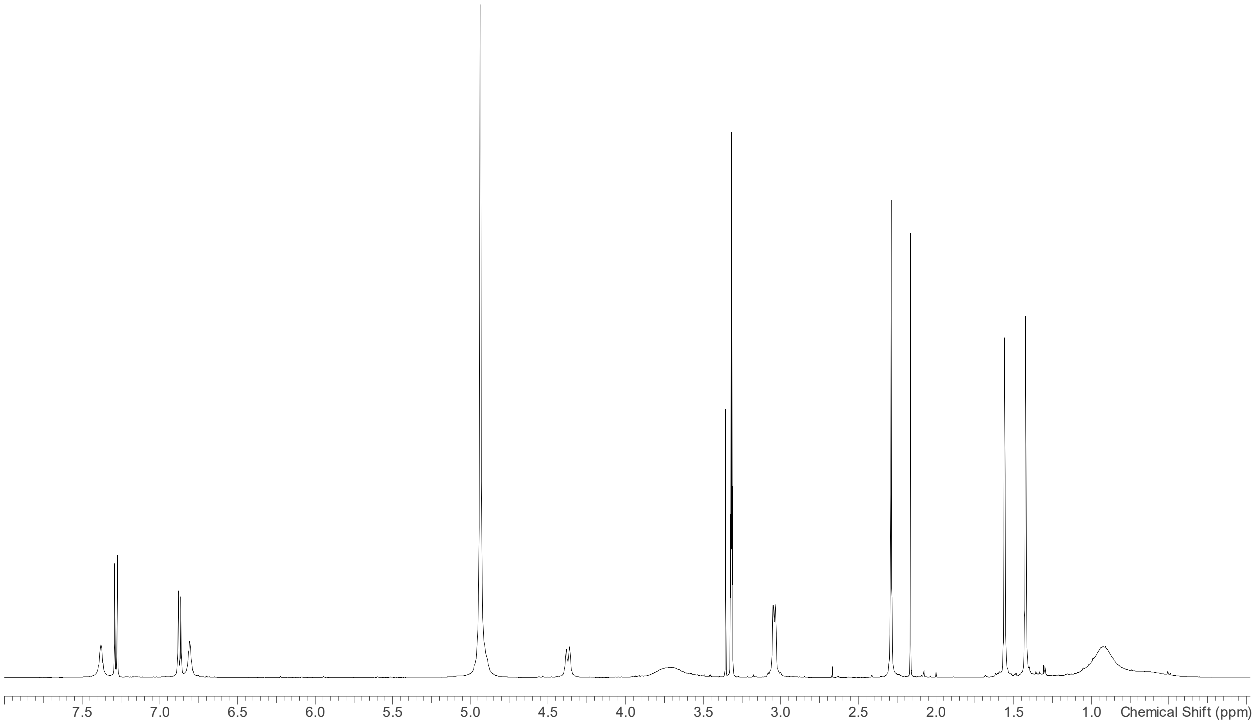

Figure S20: ^{1}H NMR spectrum (MeOH-d_4, 500 MHz) of compound **2**

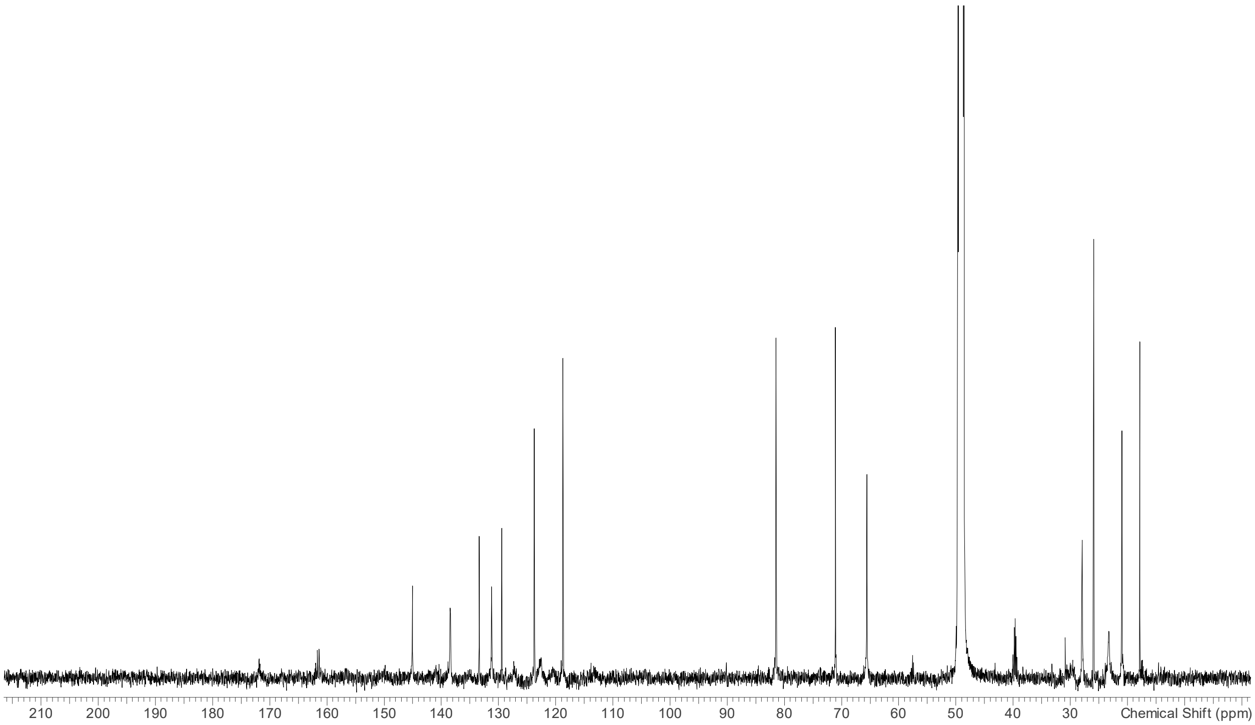

Figure S21: ^{13}C NMR spectrum (MeOH-d_4, 125 MHz) of compound **2**

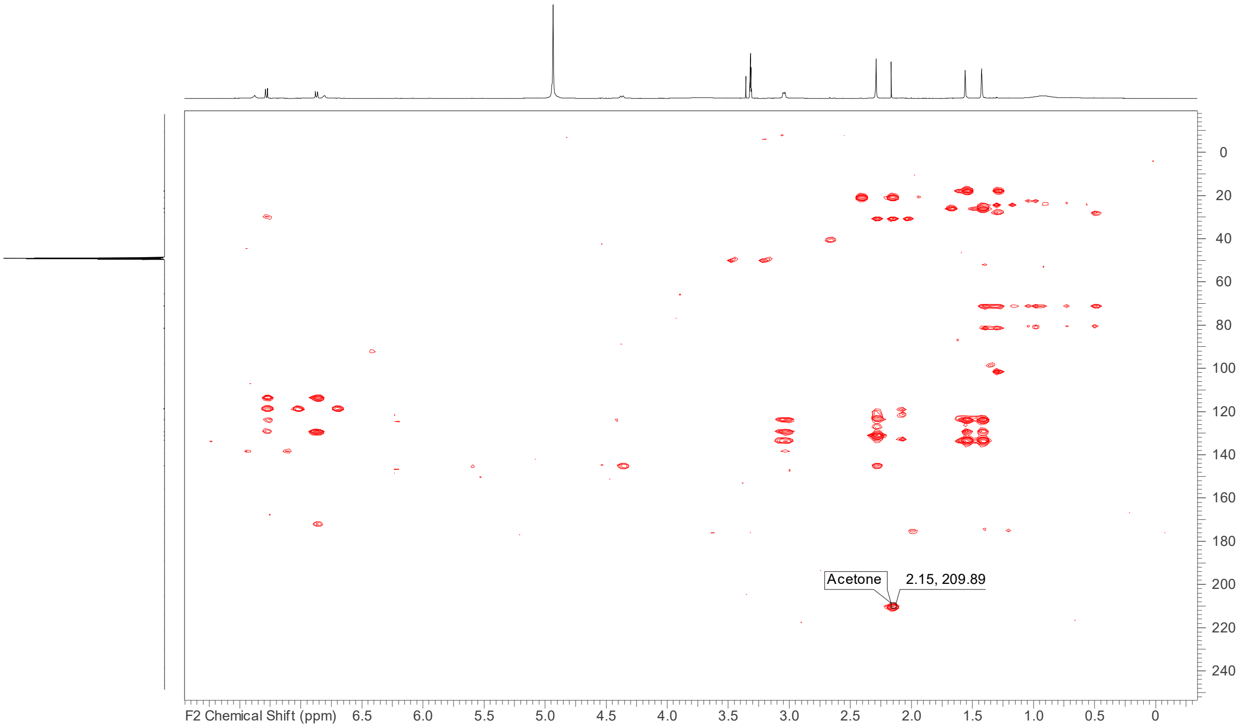

Figure S22: ^{1}H-^{13}C HMBC NMR spectrum (MeOH-d_4, 500 MHz) of compound **2**

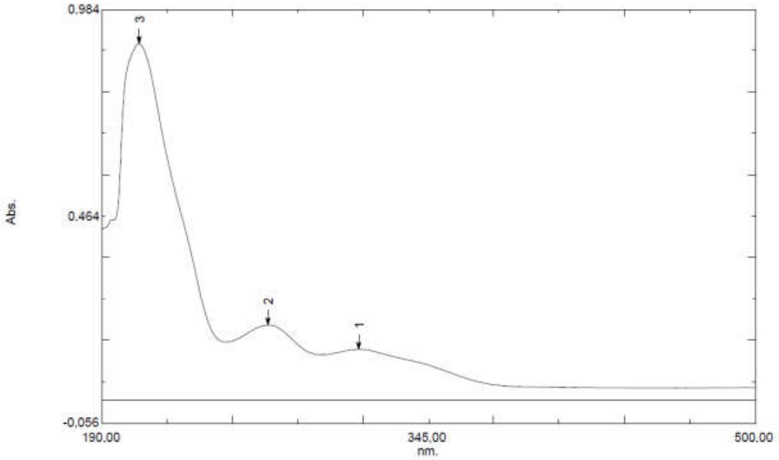

Measurement Properties
Wavelength Range (nm.): 190,00 to 500,00
Scan Speed: Medium
Sampling Interval: 0,5
Auto Sampling Interval: Disabled
Scan Mode: Single

Instrument Properties
Instrument Type: UV-2400PC Series

No.	P/V	Wavelength	Abs.	Description
1	↑	312,00	0,128	
2	↑	269,00	0,189	
3	↑	207,50	0,897	
4	↓	293,00	0,114	
5	↓	249,00	0,146	

Figure S23: UV-Vis spectrum (MeOH) of isoprenylisobenzofuran B (**2**)

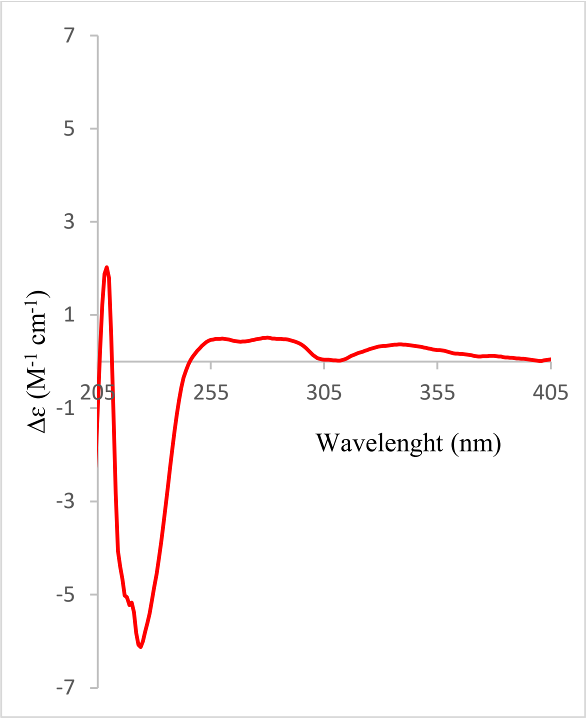

Figure S24: ECD spectrum (MeOH) of isoprenylisobenzofuran B (**2**)

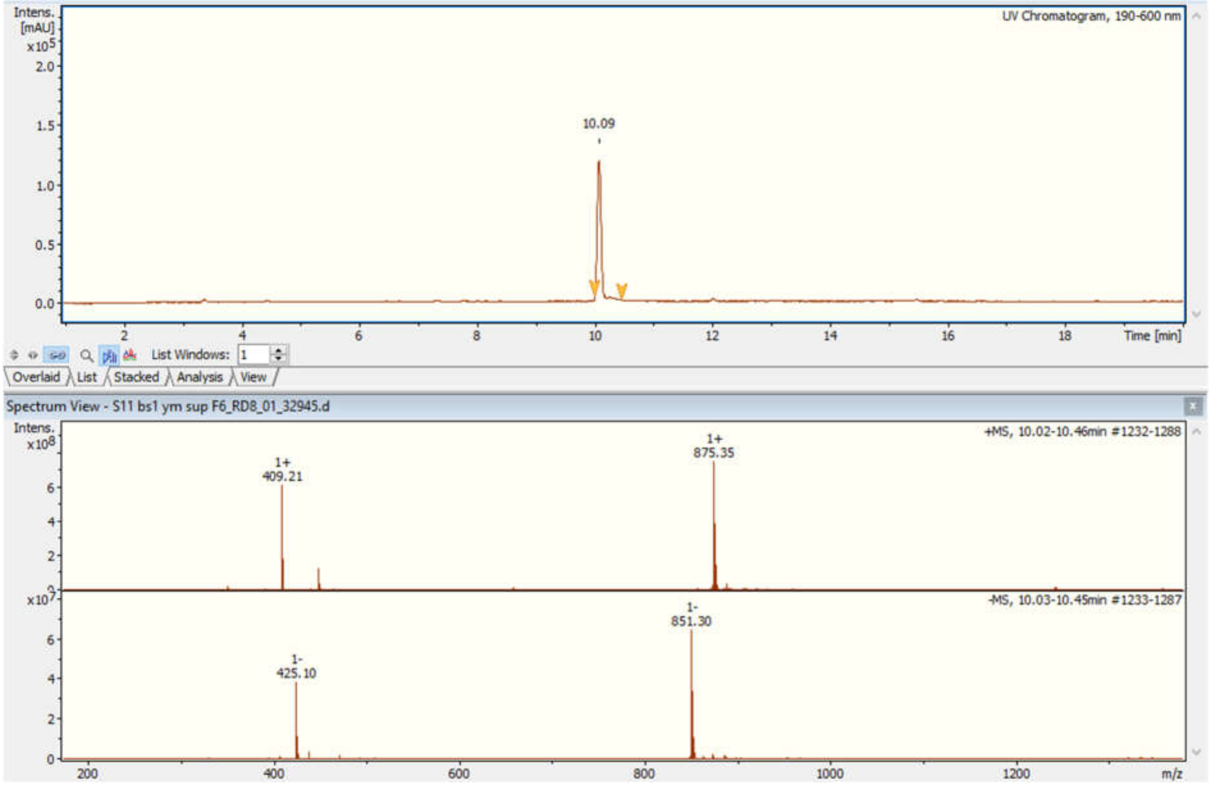

Figure S25: HPLC-DAD chromatogram and ESI-MS data for isoprenylisobenzofuran C 1/C2 (**3a** + **3b**)

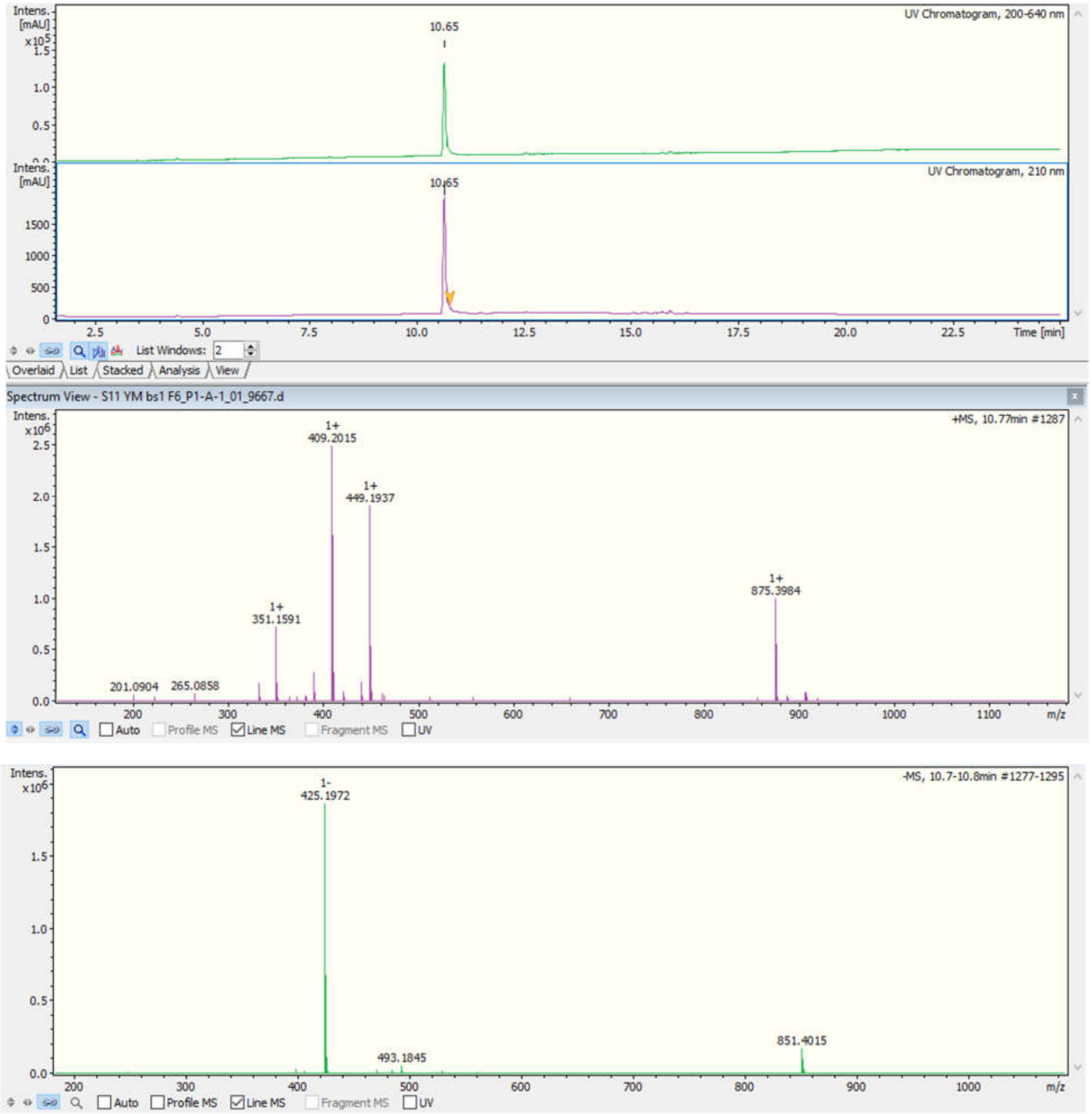

Figure S26: HPLC-DAD chromatogram and (+)/(-) HR-ESIMS data for isoprenylisobenzofuran C 1/C2 (**3a** + **3b**)

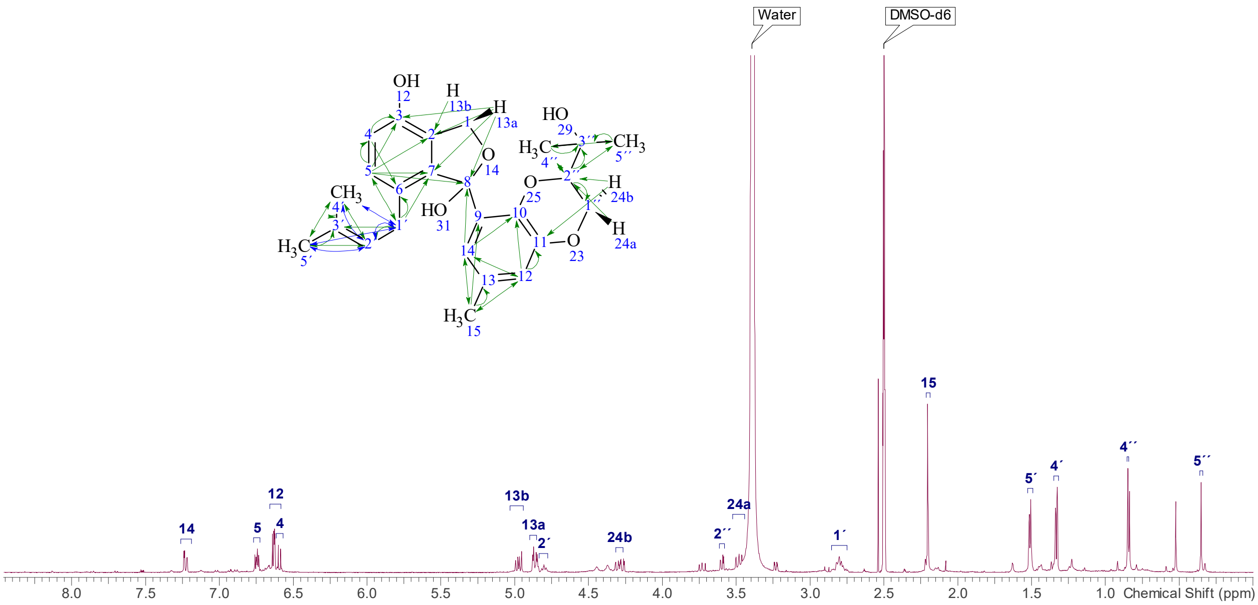

Figure S27: ^{1}H NMR spectrum (DMSO-d6, 700 MHz) of isoprenylisobenzofuran C 1/C2 (**3a** + **3b**)

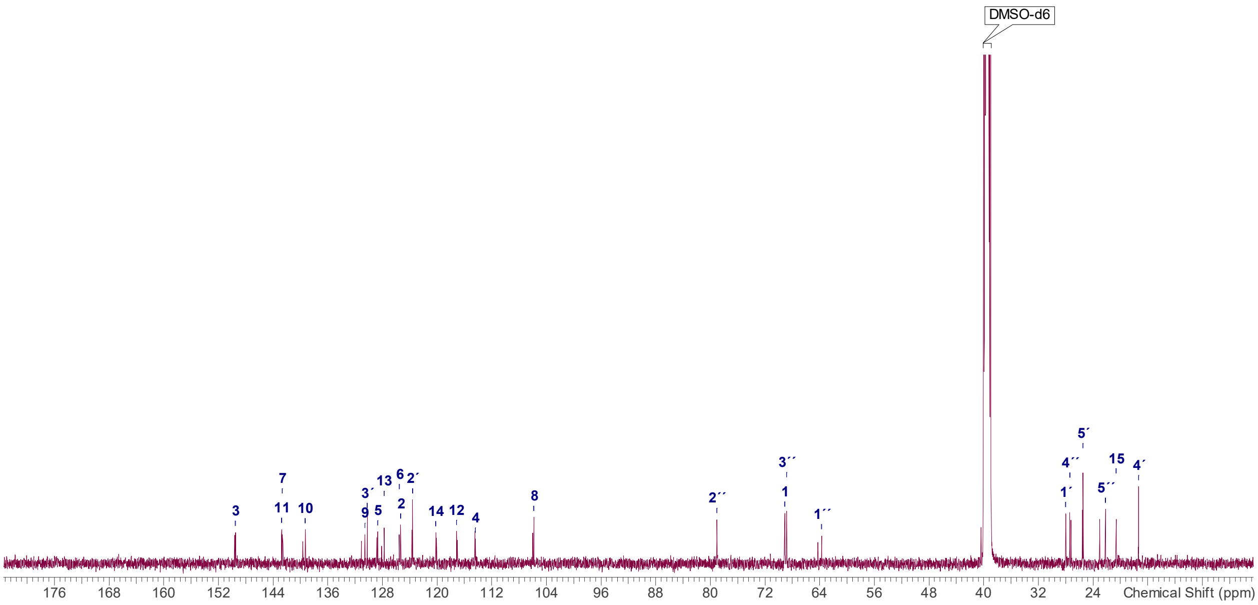

Figure S28: ^{13}C NMR spectrum (DMSO-d6, 175 MHz) of isoprenylisobenzofuran C1/C2 (**3a** + **3b**)

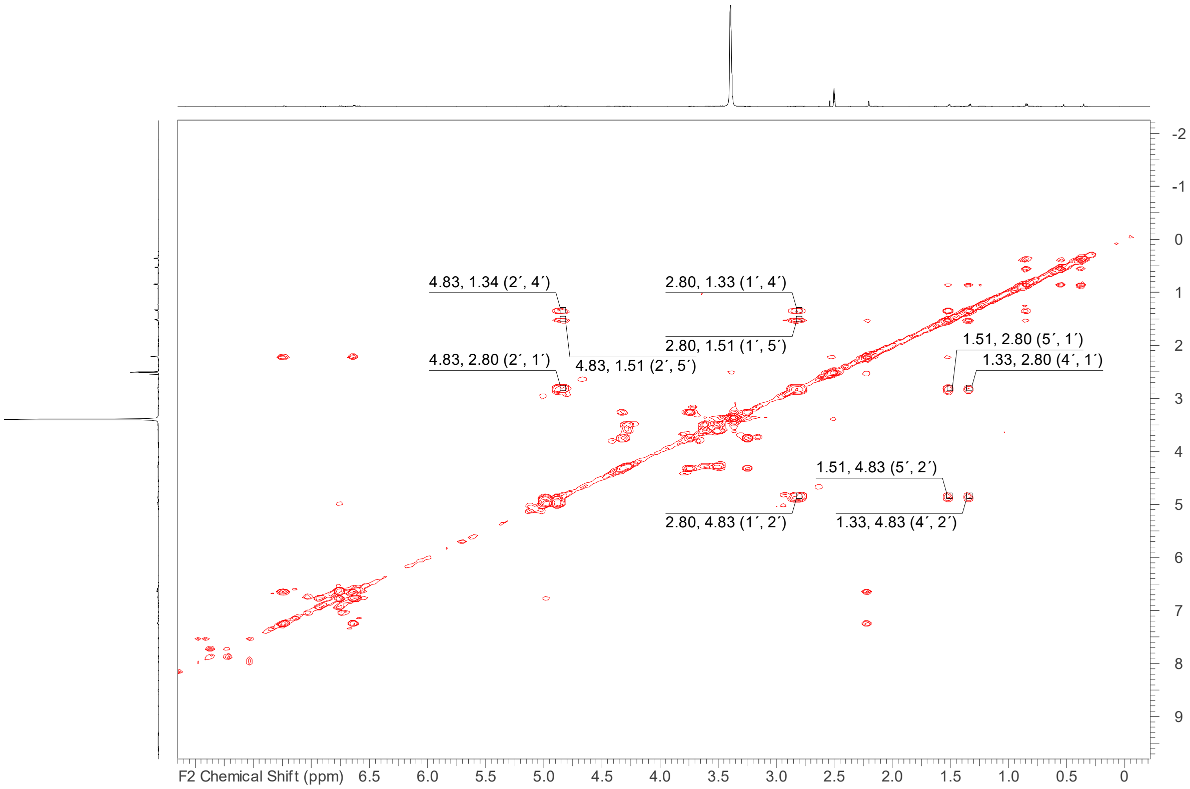

Figure S29: ^1H-^1H COSY NMR spectrum (DMSO-d_6, 700 MHz) of isoprenylisobenzofuran C_1/C_2 (**3a** + **3b**)

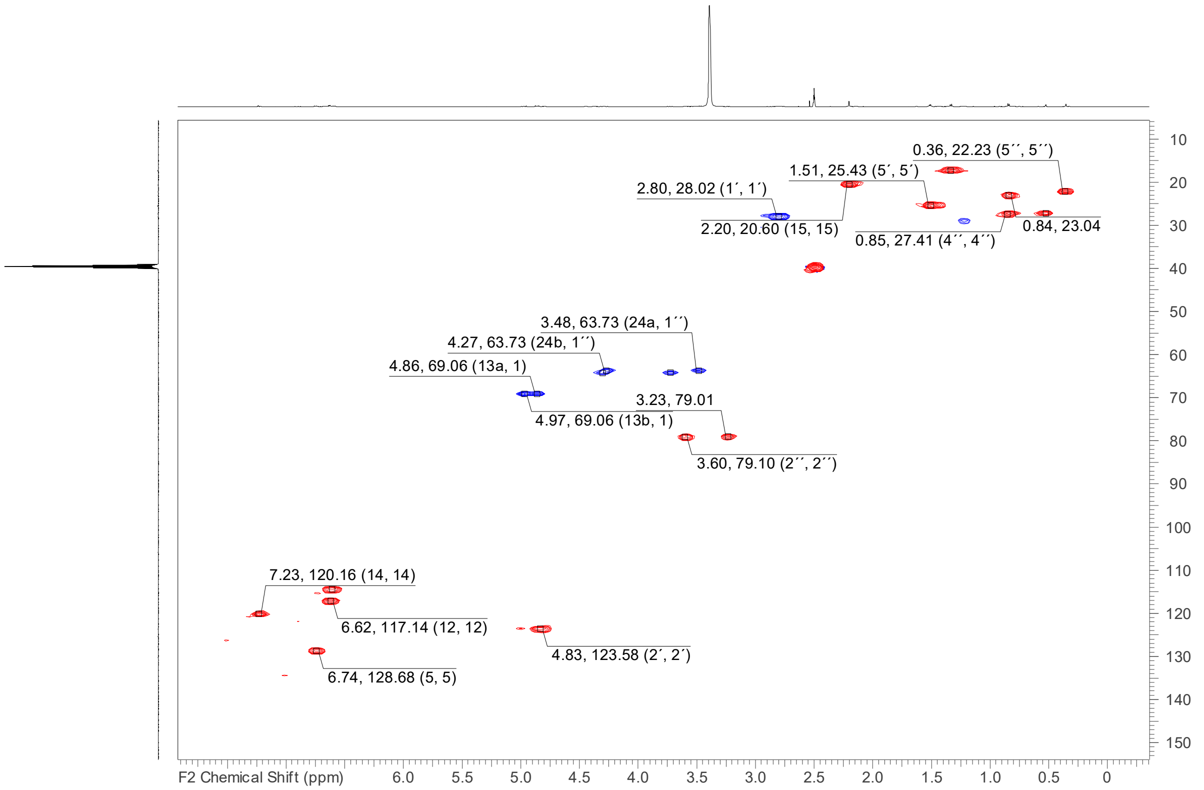

Figure S30: ^{1}H-^{13}C HSQC NMR spectrum (DMSO-d_6, 700 MHz) of isoprenylisobenzofuran C_1/C_2 (**3a** + **3b**)

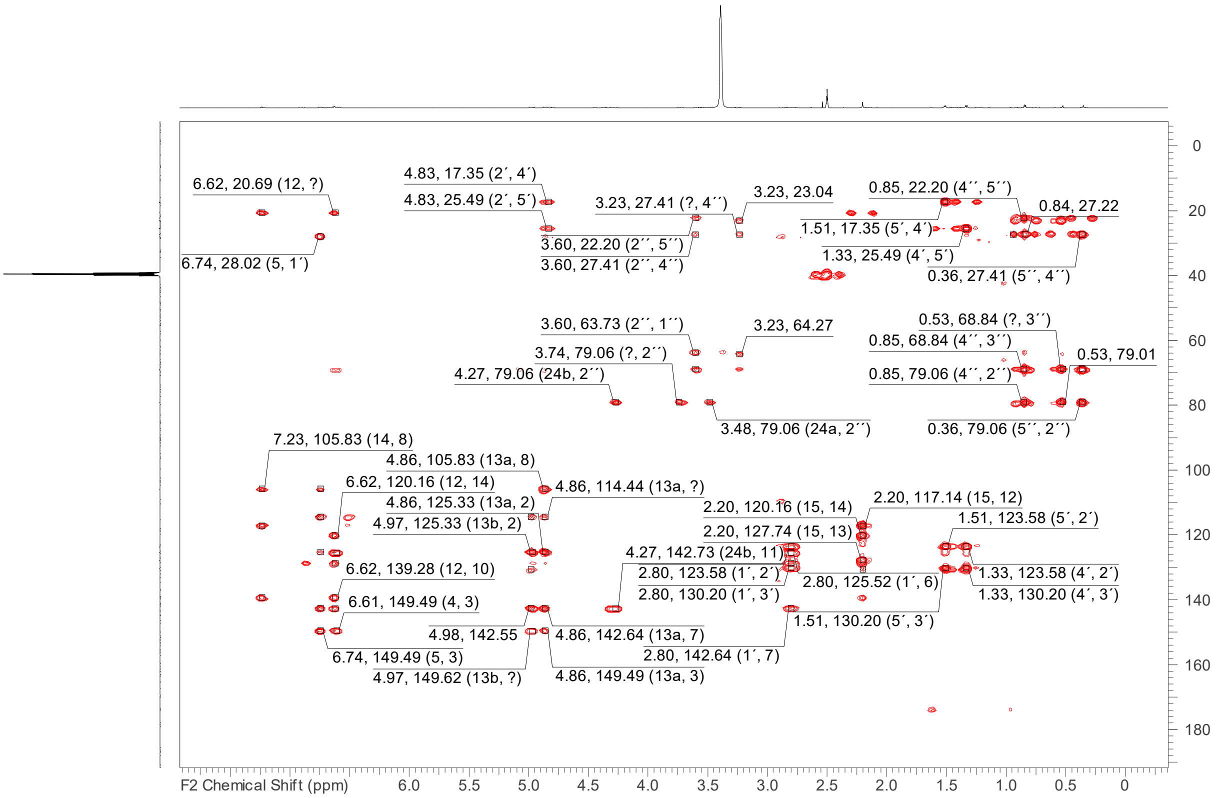

Figure S31: ^{1}H-^{13}C HMBC NMR spectrum (DMSO-d_6, 700 MHz) of isoprenylisobenzofuran C1/C2 (**3a** + **3b**)

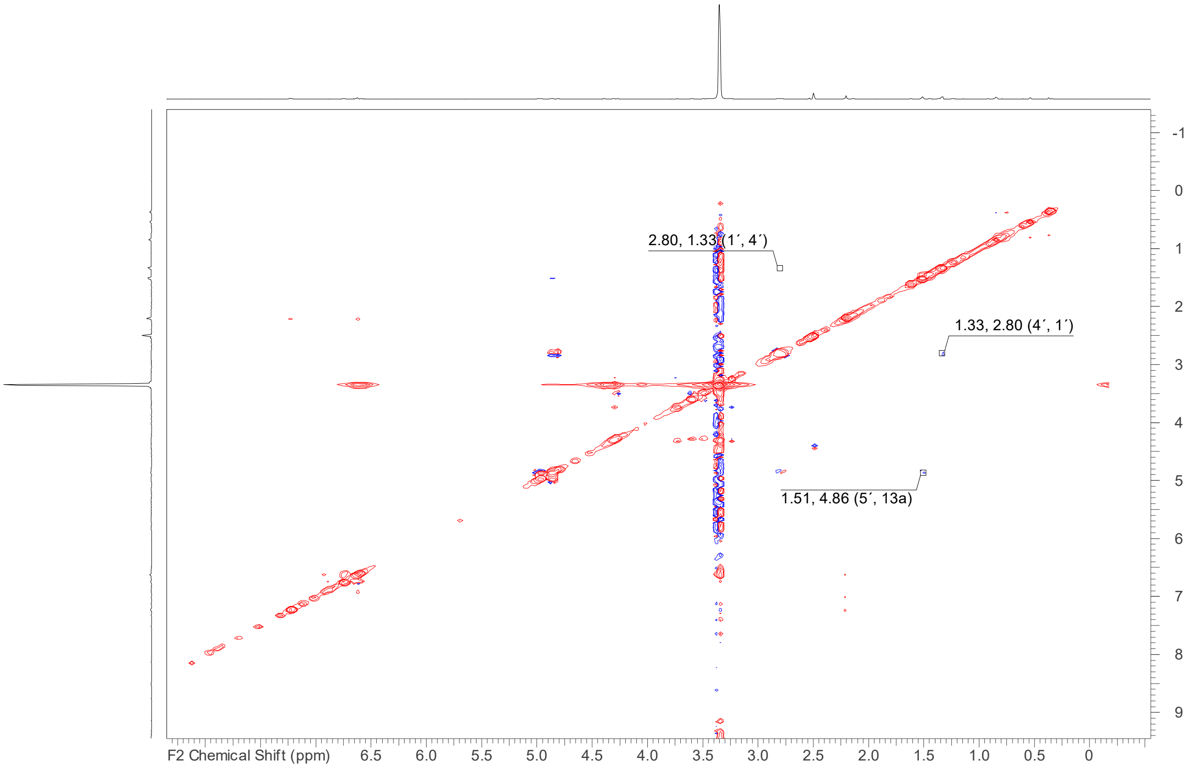

Figure S32: ^{1}H-^{1}H ROESY NMR spectrum (DMSO-d_6, 700 MHz) of isoprenylisobenzofuran C_1/C_2 (**3a** + **3b**)

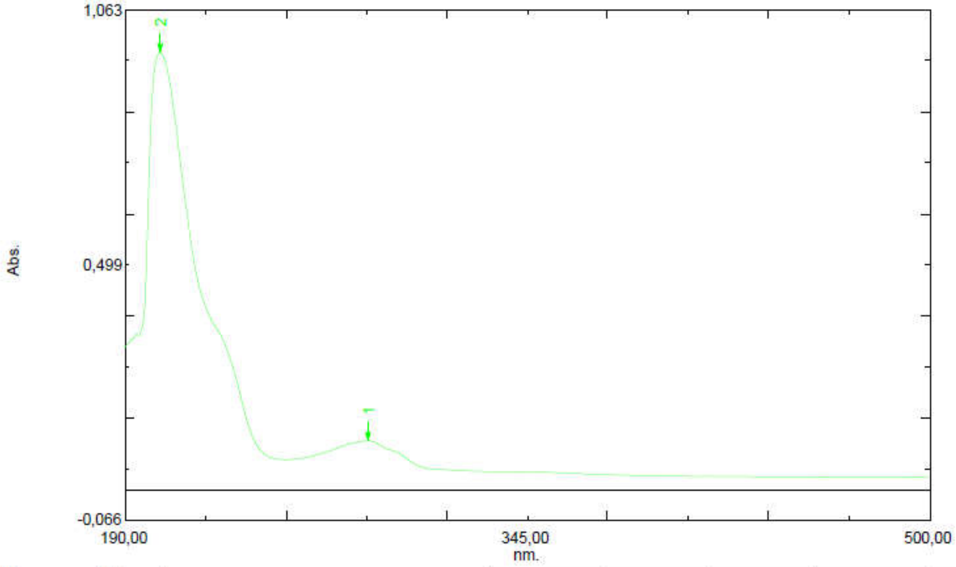

Measurement Properties
Wavelength Range (nm.): 190,00 to 500,00
Scan Speed: Medium
Sampling Interval: 0,5
Auto Sampling Interval: Disabled
Scan Mode: Single

No.	P/V	Wavelength	Abs.	Description
1	↑	283,50	0,110	
2	↑	203,50	0,969	
3	↓	252,00	0,067	

Figure S33: UV-Vis spectrum (MeOH) of isoprenylisobenzofuran C_1/C_2 (**3a** + **3b**)

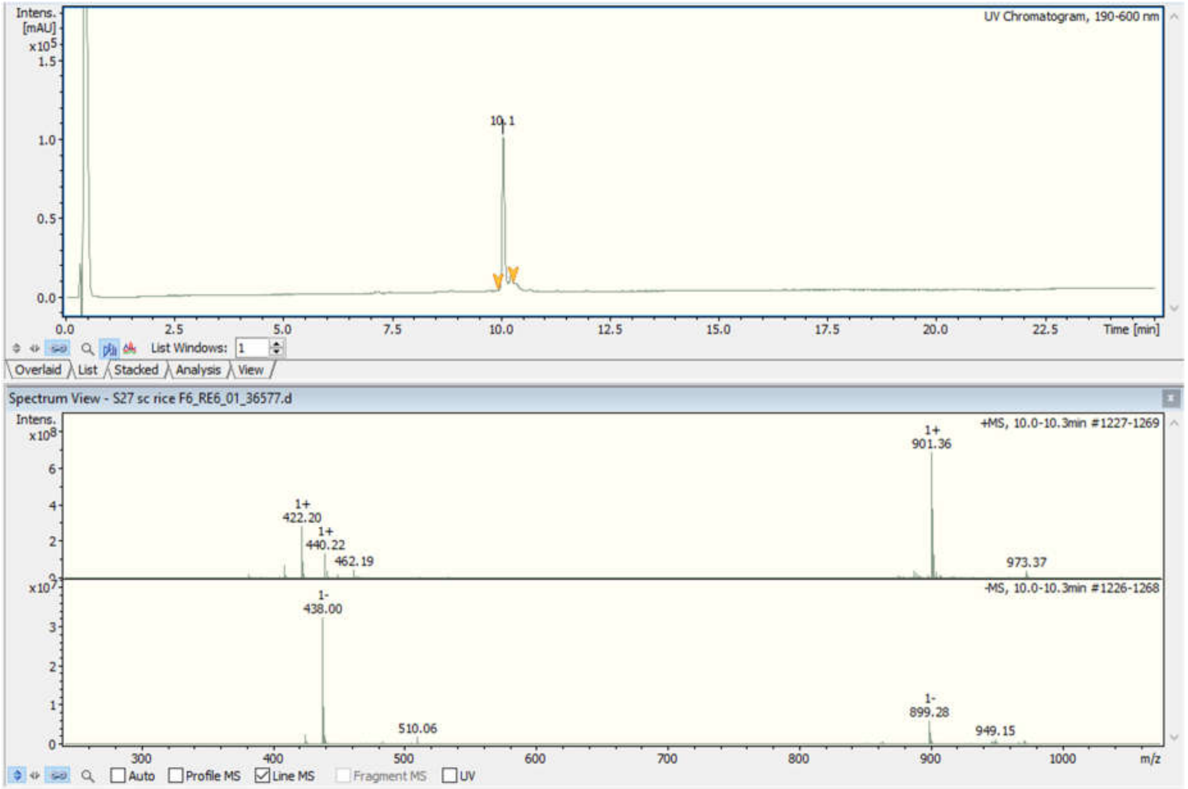

Figure S34: HPLC-DAD chromatogram and ESI-MS data for diaporisoindole F_1/F_2 (**4a** + **4b**)

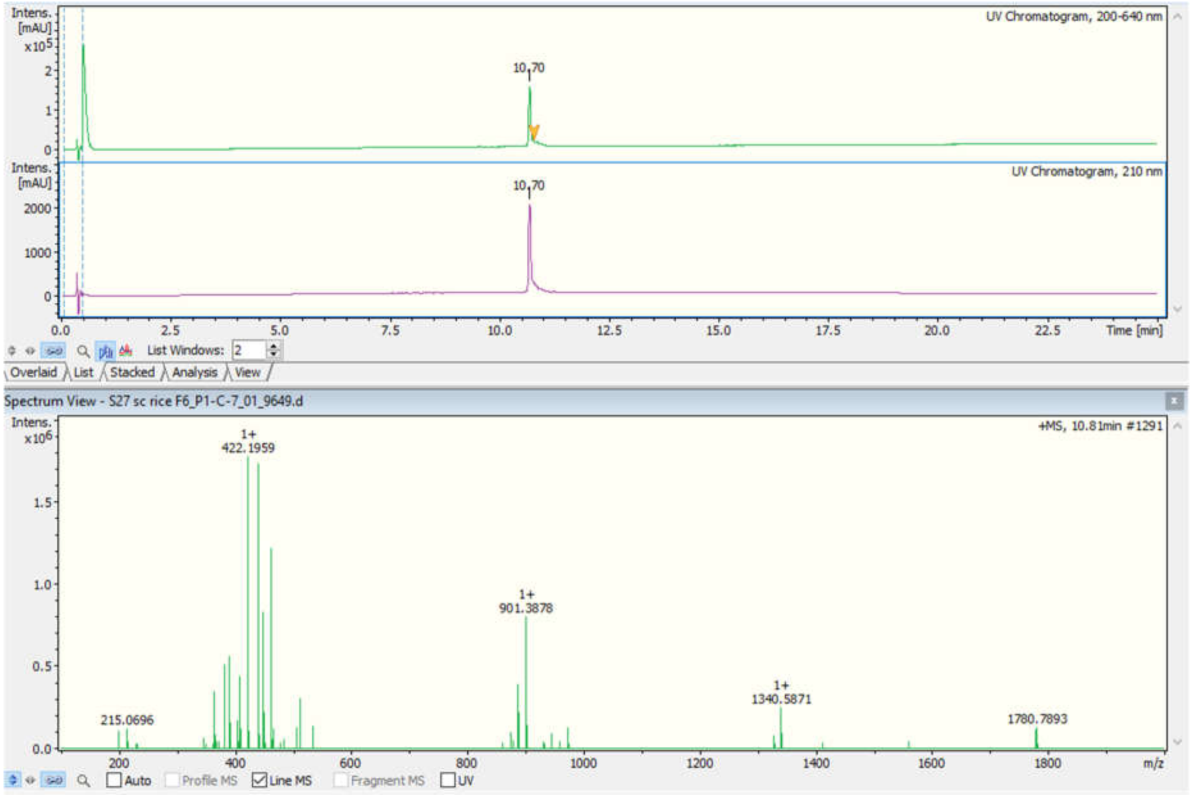

Figure S35: HPLC-DAD chromatogram and HR-ESI (+) MS data for diaporisoindole F1/F2 (**4a** + **4b**)

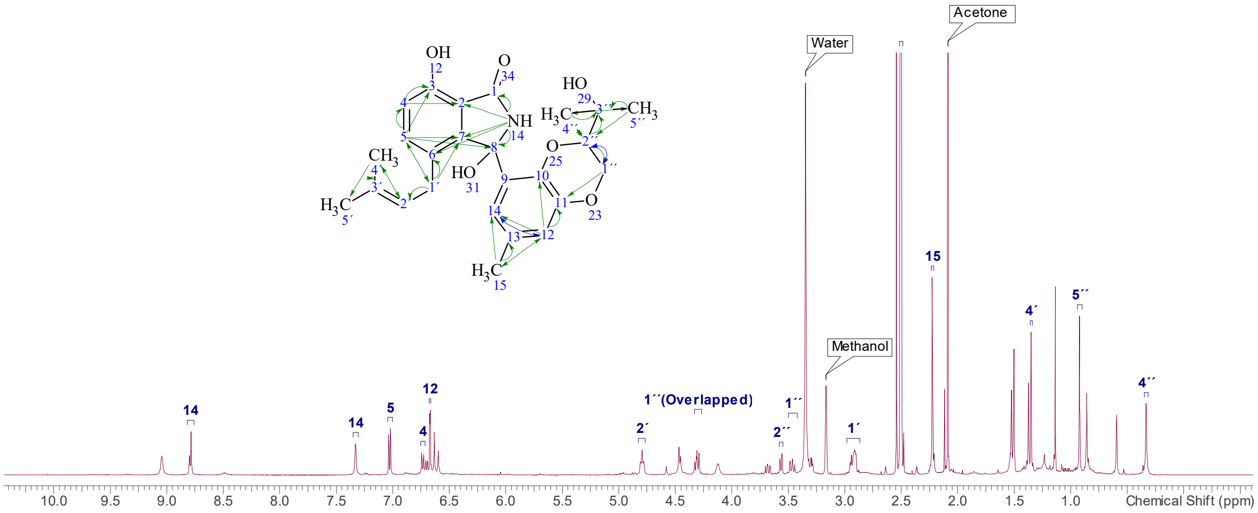

Figure S36: ^{1}H NMR spectrum (DMSO-d$_6$, 700 MHz) of diaporisoindole F1/F2 (**4a** + **4b**)

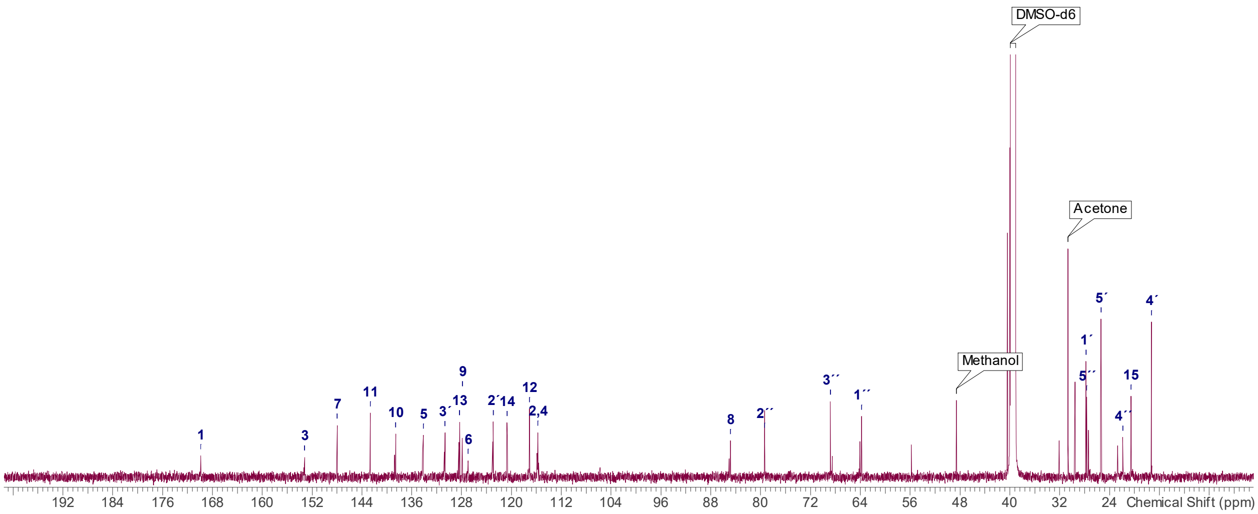

Figure S37: ^{13}C NMR spectrum (DMSO-d_6, 175 MHz) of diaporisoindole F_1/F_2 (**4a** + **4b**)

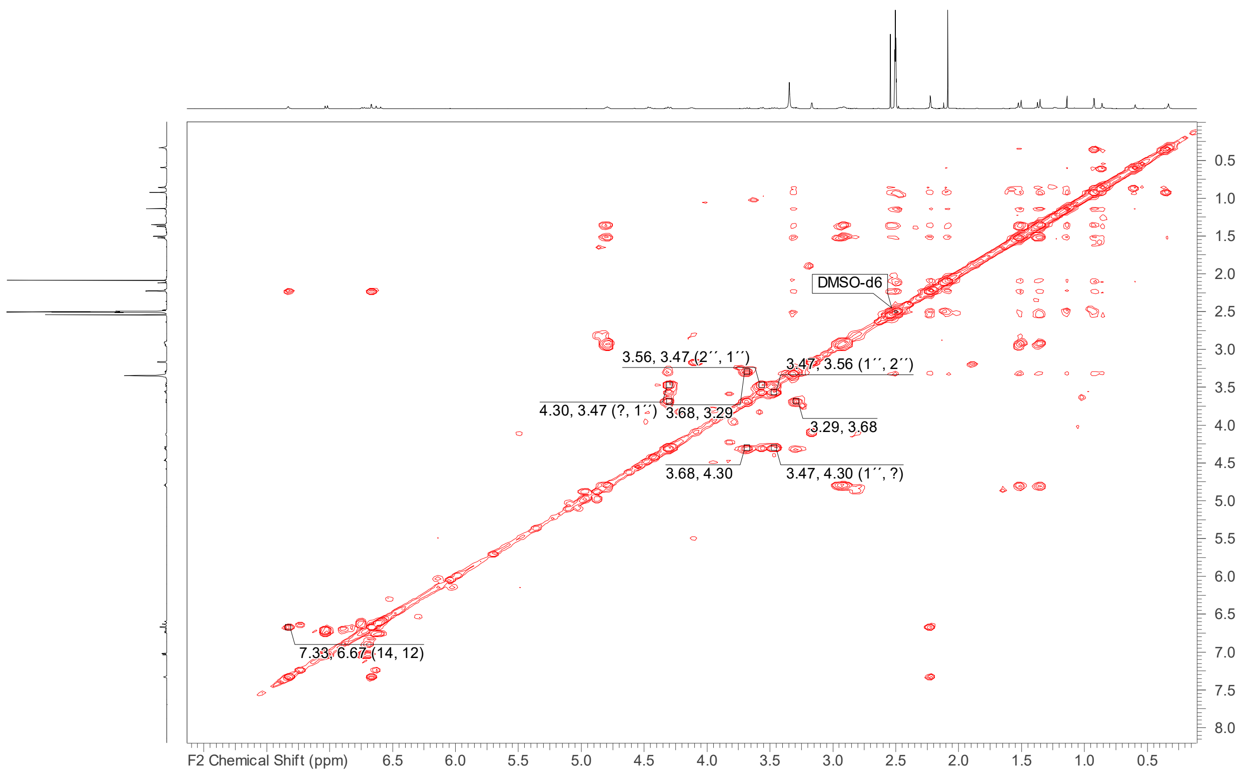

Figure S38: ^{1}H-^{1}H COSY NMR spectrum (DMSO-d_6, 700 MHz) of diaporisoindole F_1/F_2 (**4a** + **4b**)

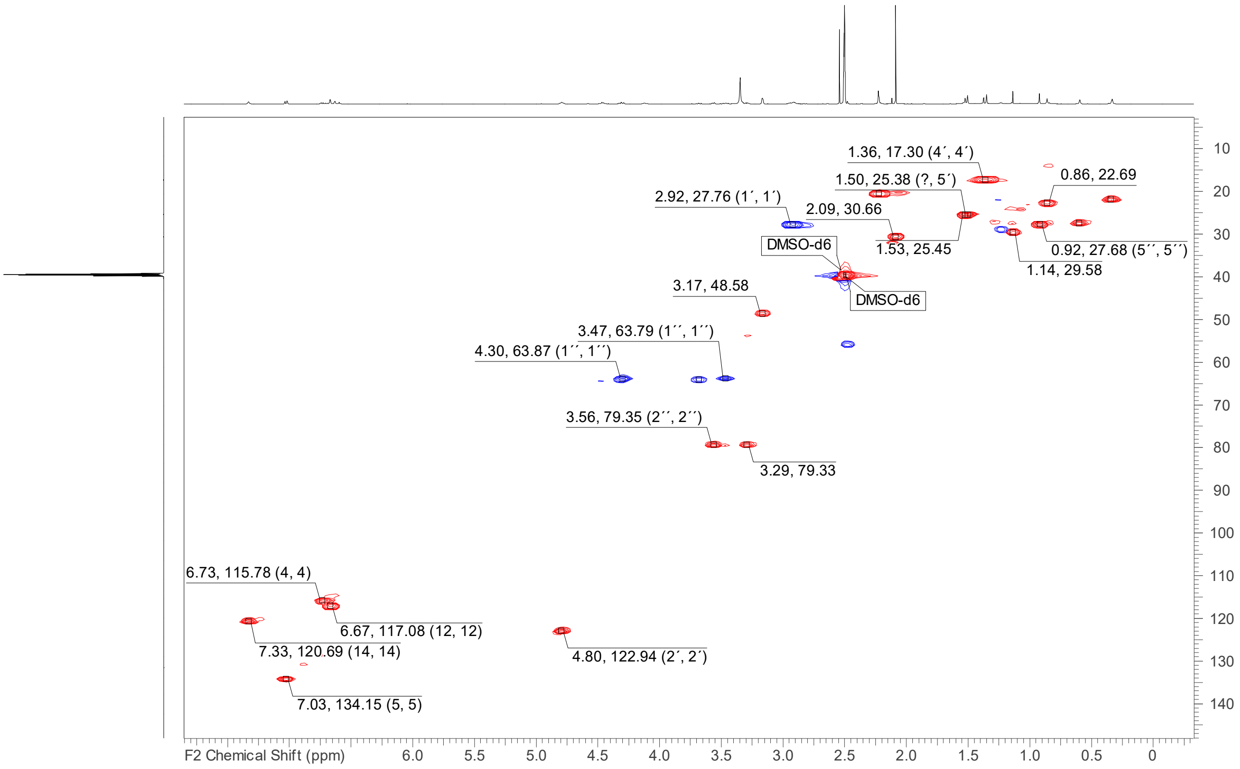

Figure S39: ^{1}H-^{13}C HSQC NMR spectrum (DMSO-d_6, 700 MHz) of diaporisoindole F_1/F_2 (**4a** + **4b**)

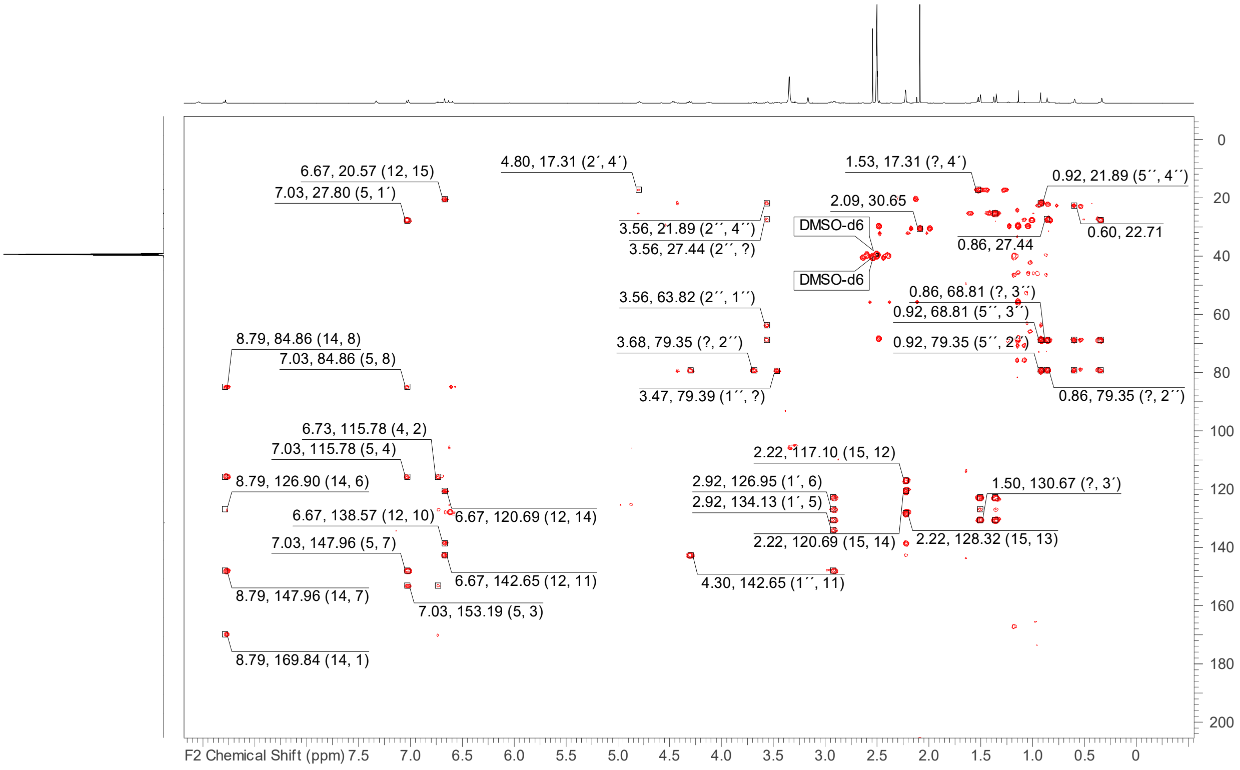

Figure S40: ^{1}H-^{13}C HMBC NMR spectrum (DMSO-d_6, 700 MHz) of diaporisoindole F_1/F_2 (**4a** + **4b**)

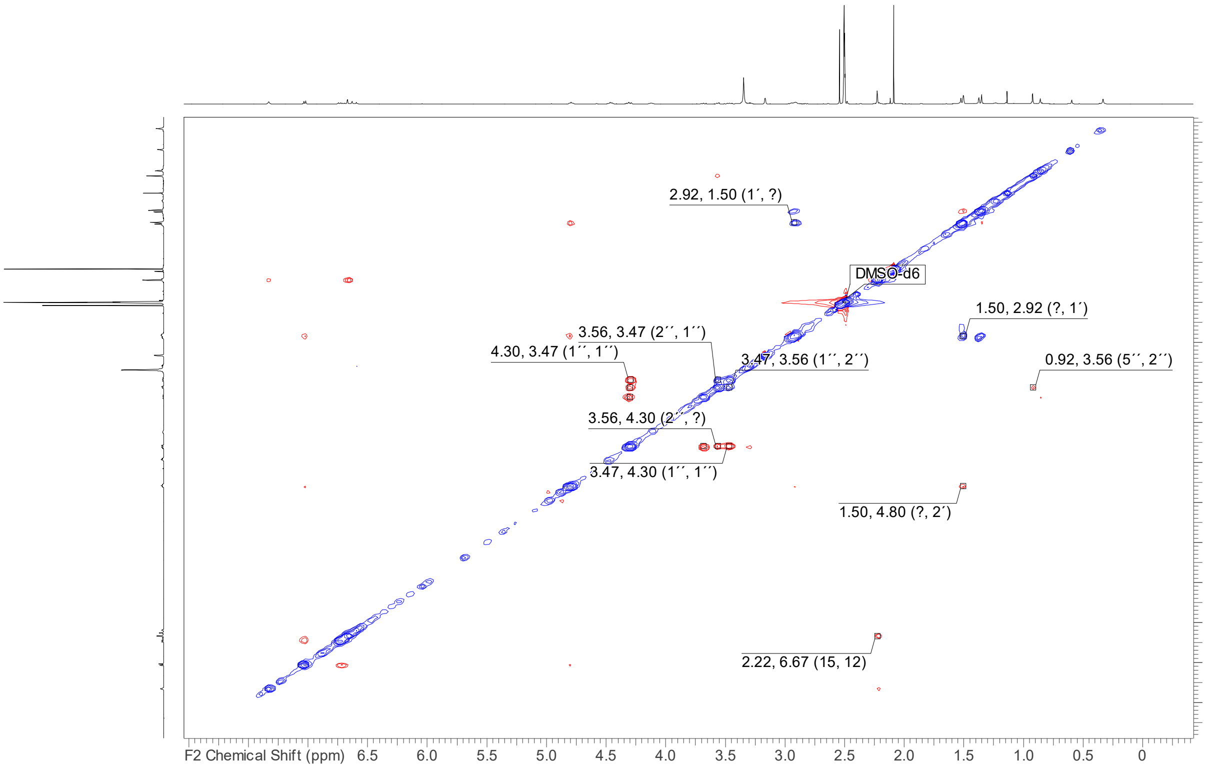

Figure S41: ^{1}H-^{1}H ROESY NMR spectrum (DMSO-d_6, 700 MHz) of diaporisoindole F_1/F_2 (**4a** + **4b**)

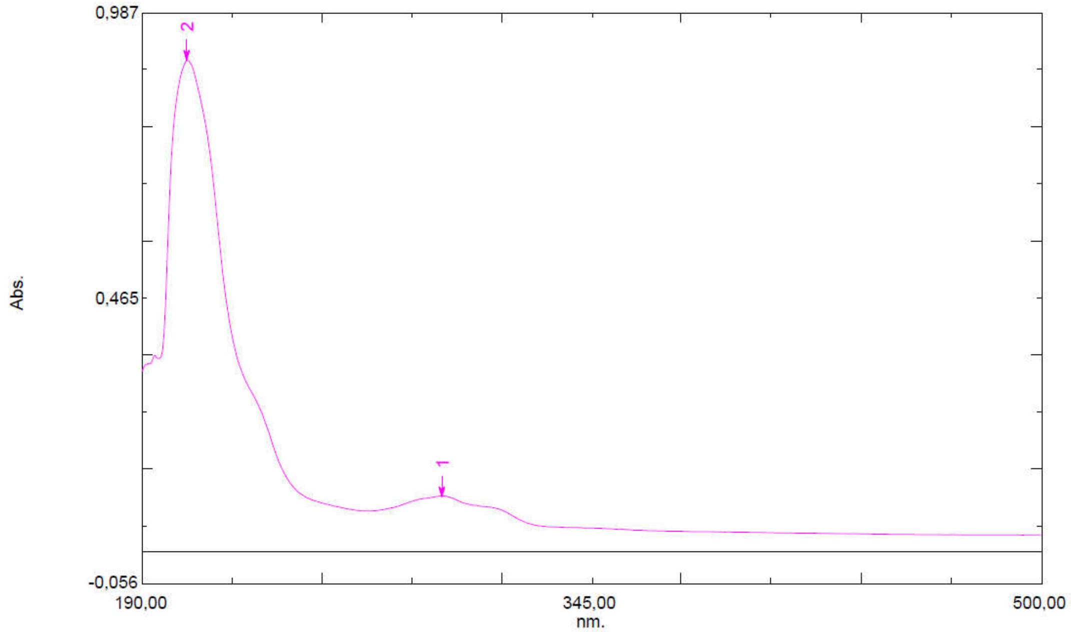

Measurement Properties
Wavelength Range (nm.): 190,00 to 500,00
Scan Speed: Medium
Sampling Interval: 0,5
Auto Sampling Interval: Disabled
Scan Mode: Single

No.	P/V	Wavelength	Abs.	Description
1	↑	293,50	0,102	
2	↑	205,50	0,900	
3	↓	268,00	0,075	

Figure S42: UV-Vis spectrum (MeOH) of diaporisoindole F_1/F_2 (**4a** + **4b**)

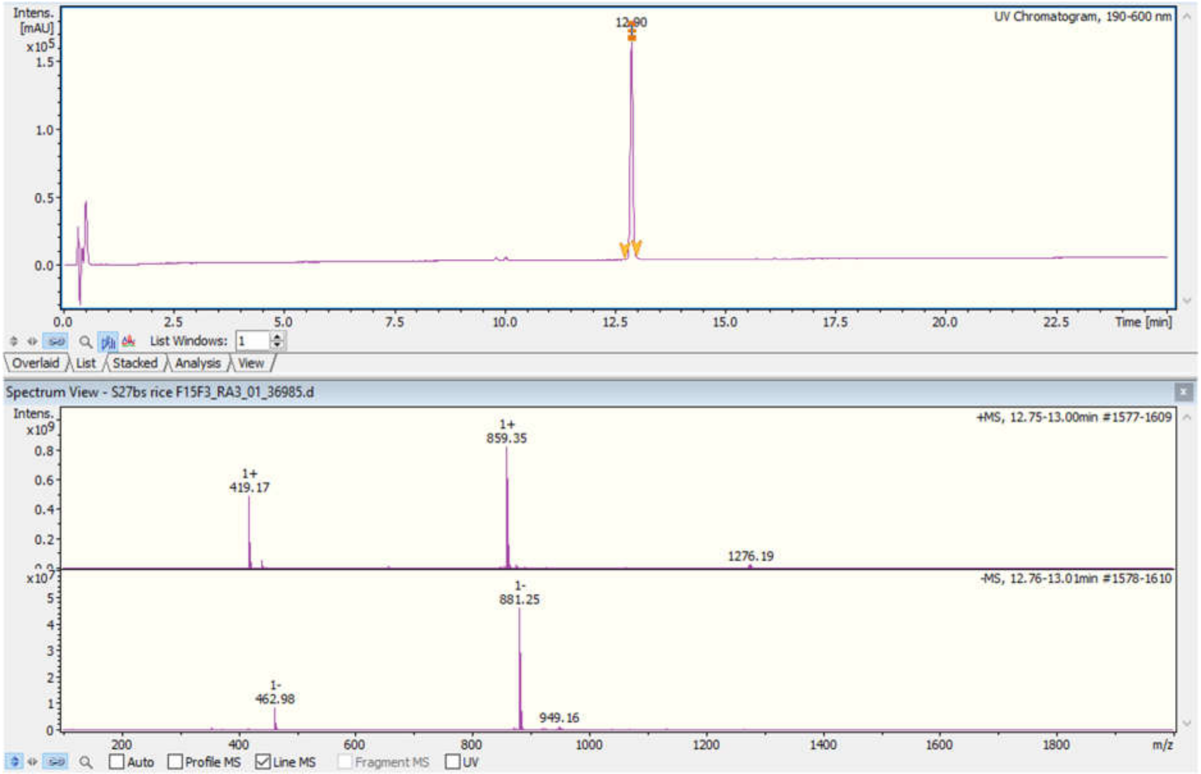

Figure S43: HPLC-DAD chromatogram and ESI-MS data for isochromophilonol A1/A2 (**7a** + **7b**)

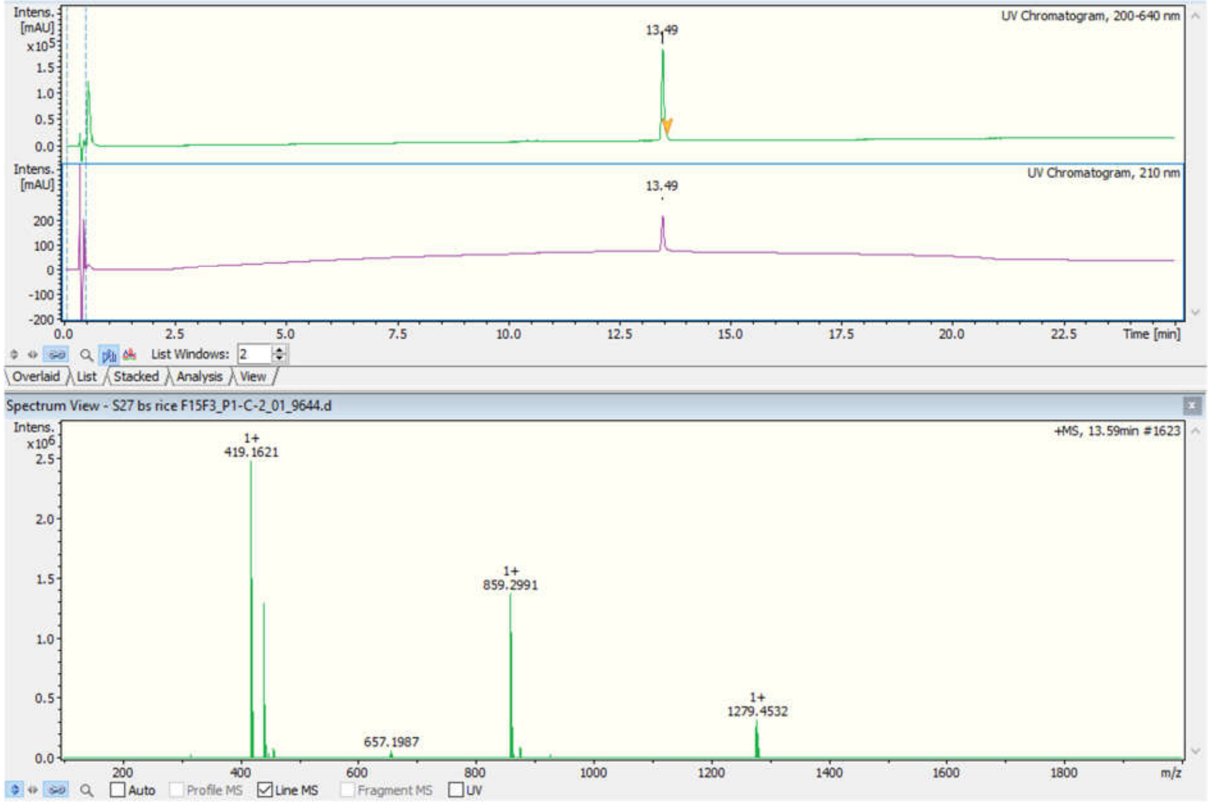

Figure S44: HPLC-DAD chromatogram and HR-ESI (+) MS data for isochromophilonol A1/A2 (**7a** + **7b**)

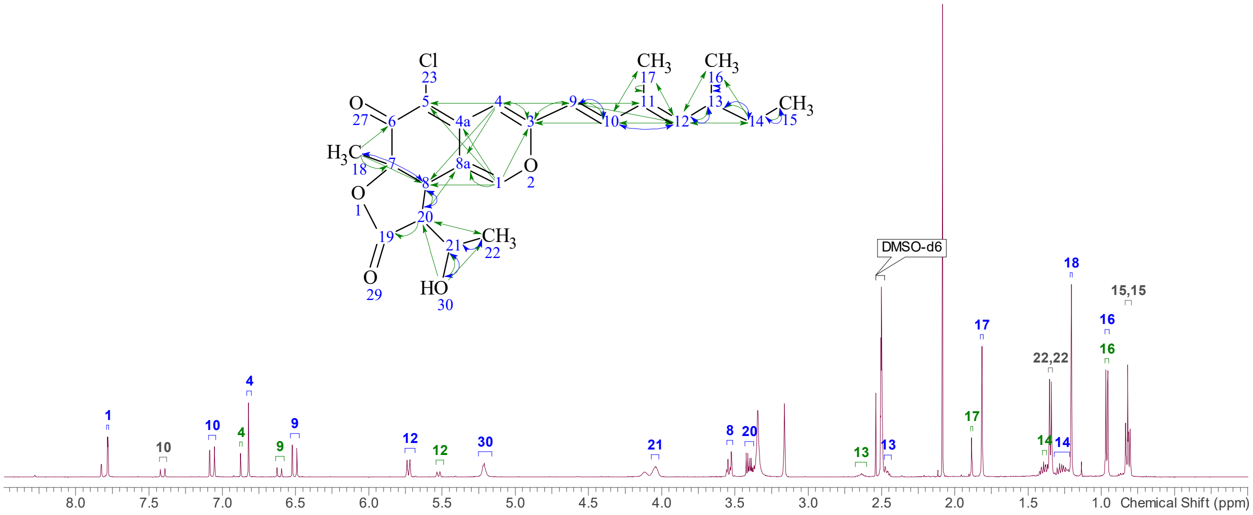

Figure S45: ^{1}H NMR spectrum (DMSO-d$_6$, 500 MHz) of isochromophilonol A$_1$/A$_2$ (**7a** + **7b**)

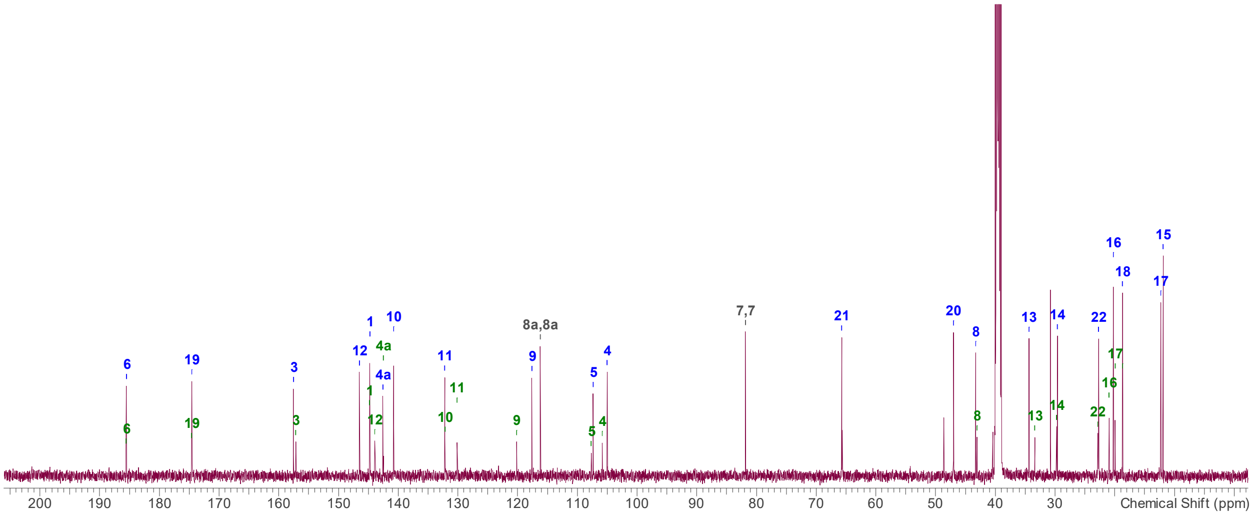

Figure S46: ^{13}C NMR spectrum (DMSO-d$_6$, 125 MHz) of isochromophilonol A$_1$/A$_2$ (**7a** + **7b**)

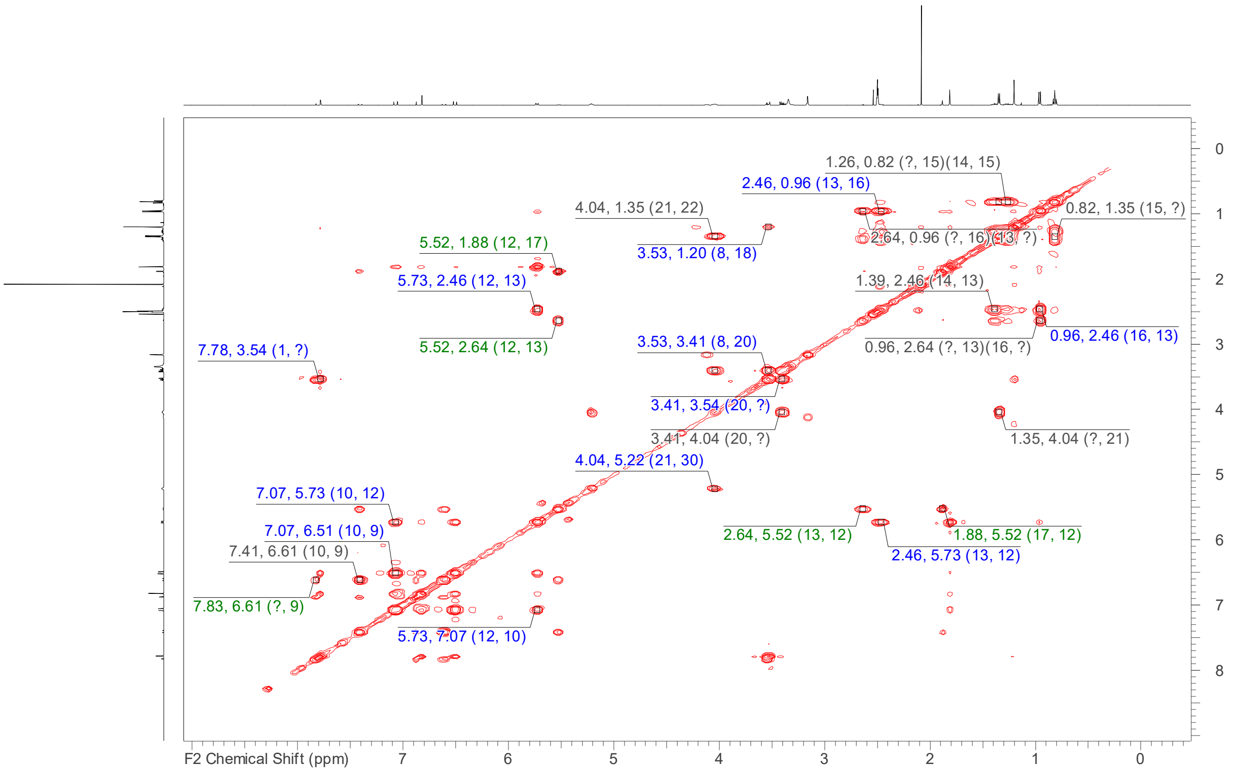

Figure S47: ^{1}H-^{1}H COSY NMR spectrum (DMSO-d_6, 500 MHz) of isochromophilonol A_1/A_2 (**7a** + **7b**)

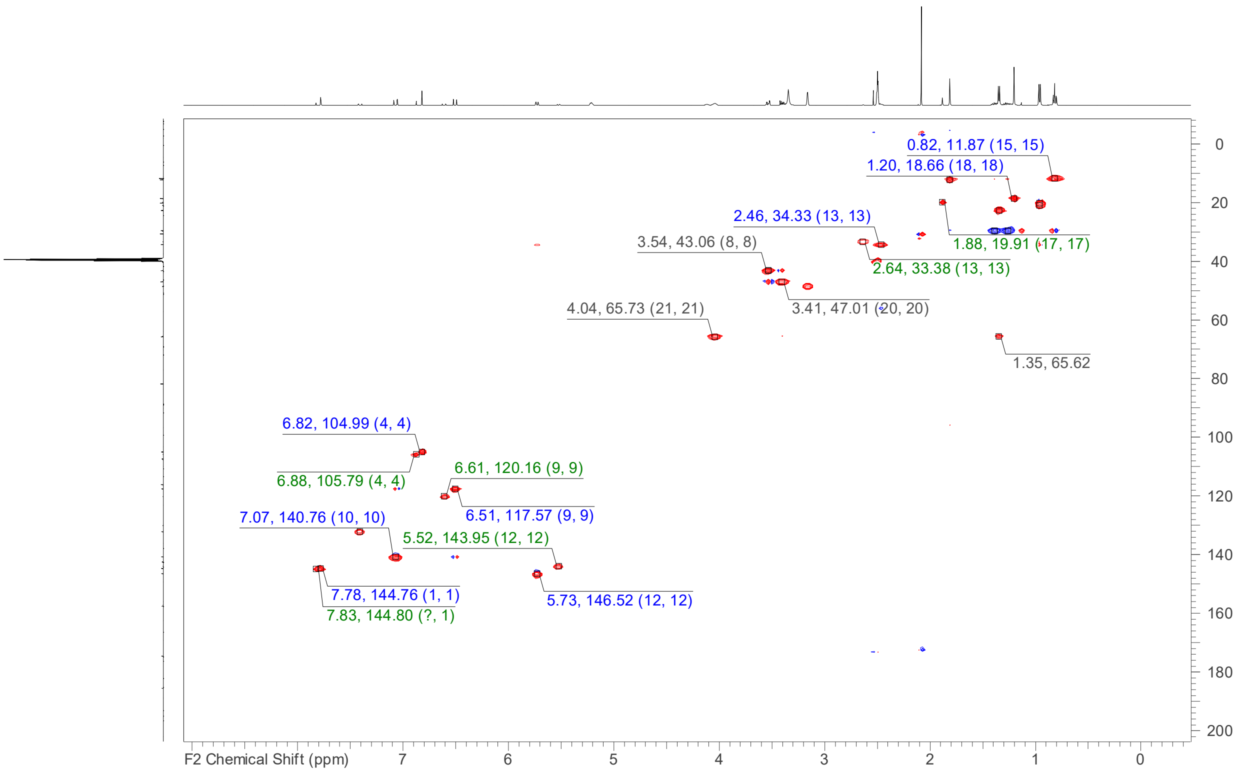

Figure S48: ^{1}H-^{13}C HSQC NMR spectrum (DMSO-d_6, 500 MHz) of isochromophilonol A_1/A_2 (**7a** + **7b**)

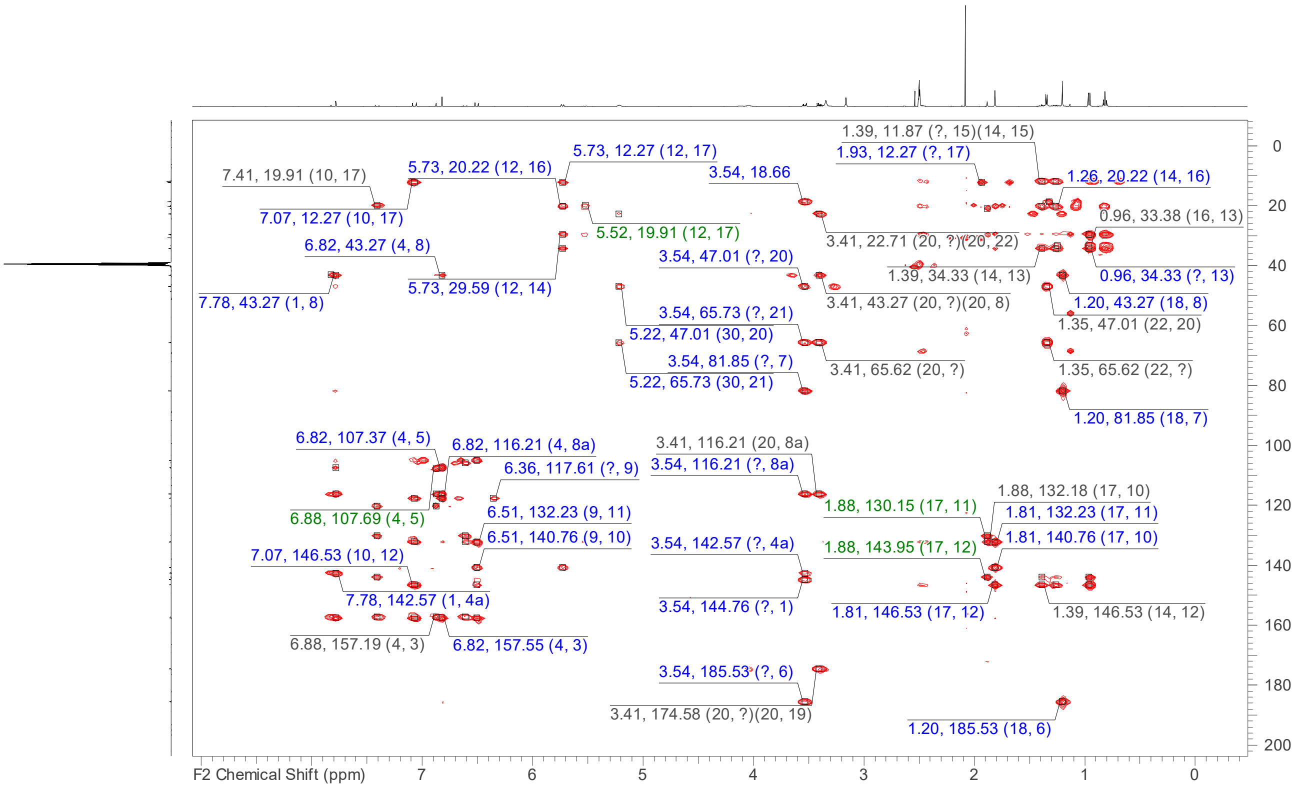

Figure S49: ^{1}H-^{13}C HMBC NMR spectrum (DMSO-d_6, 500 MHz) of isochromophilonol A$_1$/A$_2$ (**7a** + **7b**)

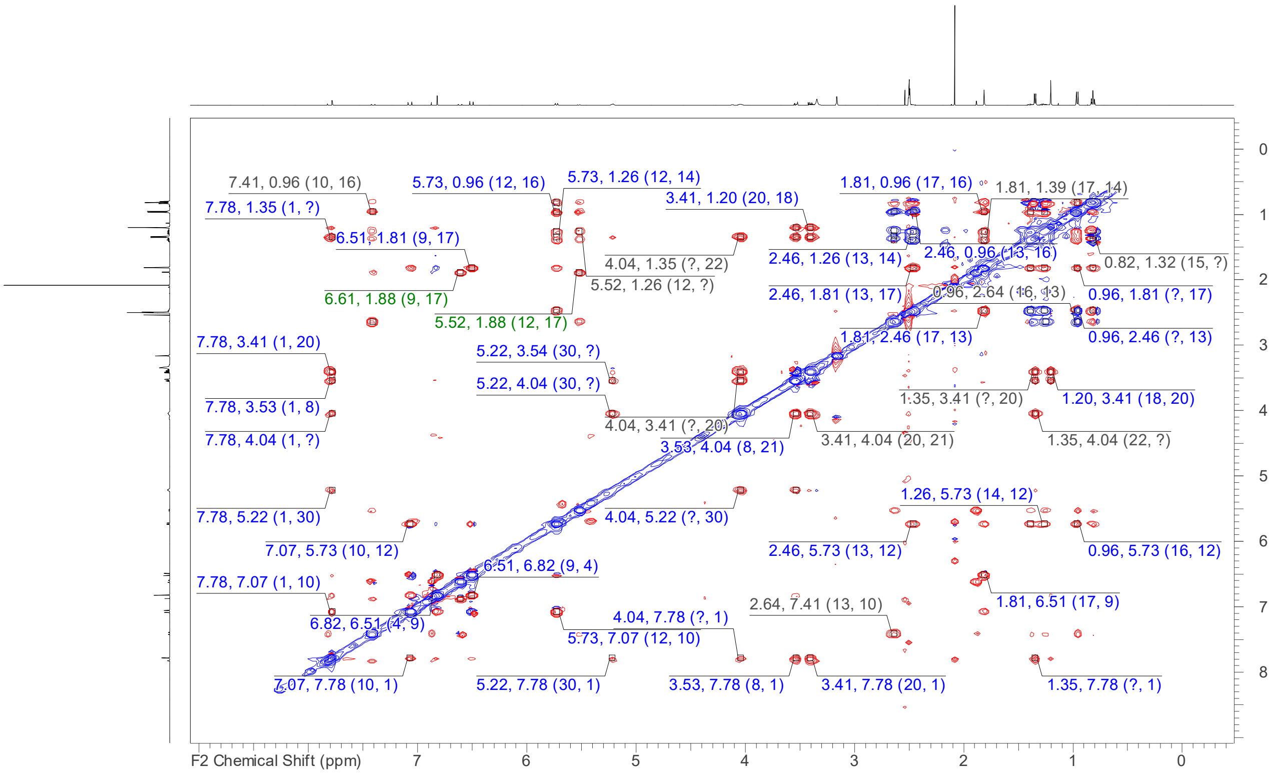

Figure S50: ^1H-^1H ROESY NMR spectrum (DMSO-d_6, 500 MHz) of isochromophilonol A_1/A_2 (**7a** + **7b**)

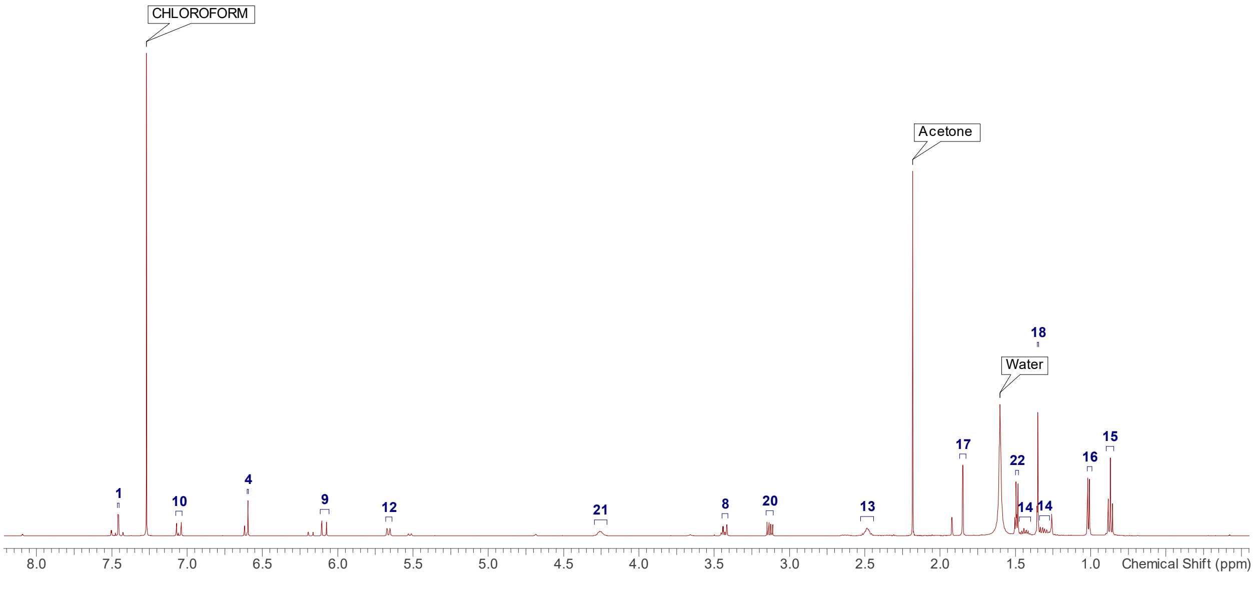

Figure S51: ^1H NMR spectrum ($CDCl_3$, 500 MHz) of isochromophilonol A_1/A_2 (**7a** + **7b**)

Table S2: Comparison of ^{1}H NMR data of compound **7** with that of isochromophilonol in $CDCl_3$.

	**7a**	**7b**	**Isochromophilonol**
No.	δ_H (J in Hz)	δ_H (J in Hz)	δ_H (J in Hz)
1	7.46, d (1.8)	7.5, d (1.8)	7.38, s
2	-	-	-
3	-	-	-
4	6.60, s	6.62, s	6.58, s
4a	-	-	-
5	-	-	-
6	-	-	-
7	-	-	-
8	3.43, dd (12.5, 1.8)	3.44, dd (12.5, 1.8)	3.45, d (12.0)
8a	-	-	-
9	6.09, d (15.7)	6.18, d (15.7)	6.08, d (15.6)
10	7.06, d (15.7)	7.06, d (15.7)	7.02, d (15.6)
11	-	-	-
12	5.66, d (9.8)	5.52, d (9.8)	5.65, d (9.6)
13	2.46, m	2.64, m	2.45, m
14	1.44, m<br>1.31, m	1.44, m<br>1.31, m	1.41, m<br>1.30, m
15	0.87, t (7.5)	0.87, t (7.5)	0.86, t (7.2)
16	1.01, d (6.6)	1.02, d (6.6)	1.0, d (6.6)
17	1.85, d (1.0)	1.92, d (1.2)	1.82, s
18	1.35, s	1.36, s	1.58, s
19	-	-	-
20	3.13, dd (12.5, 5.9)	3.13, dd (12.5, 5.9)	2.85, dd (3.6, 12.0)
21	4.25, m	4.25, m	4.30, m
22	1.49, d (6.4)	1.5, d (6.4)	1.35, d (6.6)

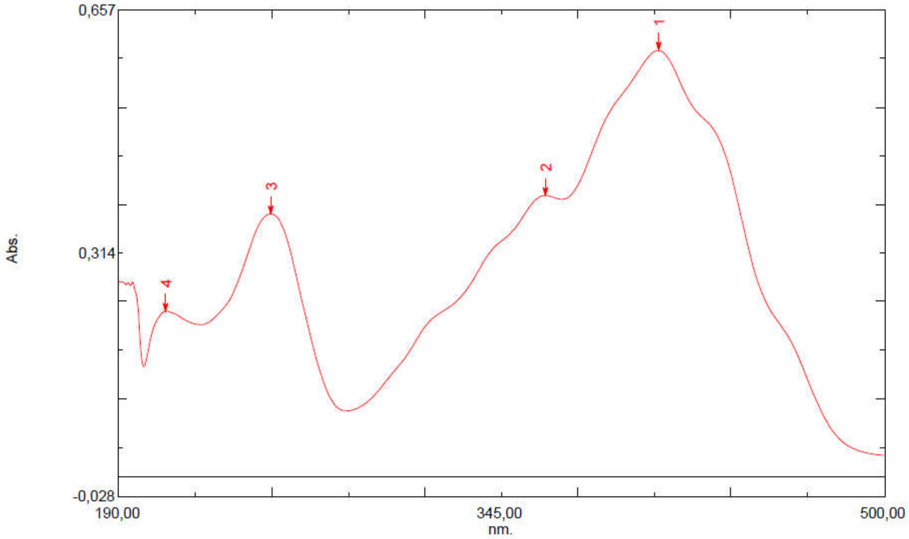

Measurement Properties	
Wavelength Range (nm.):	190,00 to 500,00
Scan Speed:	Medium
Sampling Interval:	0,5
Auto Sampling Interval:	Disabled
Scan Mode:	Single

Instrument Properties	
Instrument Type:	UV-2400PC Series
Measuring Mode:	Absorbance
Slit Width:	2,0 nm
Light Source Change Wavelength:	360,0 nm
S/R Exchange:	Normal

No.	P/V	Wavelength	Abs.	Description
1	↑	408,50	0,600	
2	↑	363,00	0,396	
3	↑	251,50	0,370	
4	↑	209,00	0,232	
5	↓	369,50	0,390	
6	↓	283,00	0,092	
7	↓	223,50	0,213	
8	↓	200,00	0,154	

Figure S52: UV-Vis spectrum (MeOH) of isochromophilonol A_1/A_2 (**7a** + **7b**)

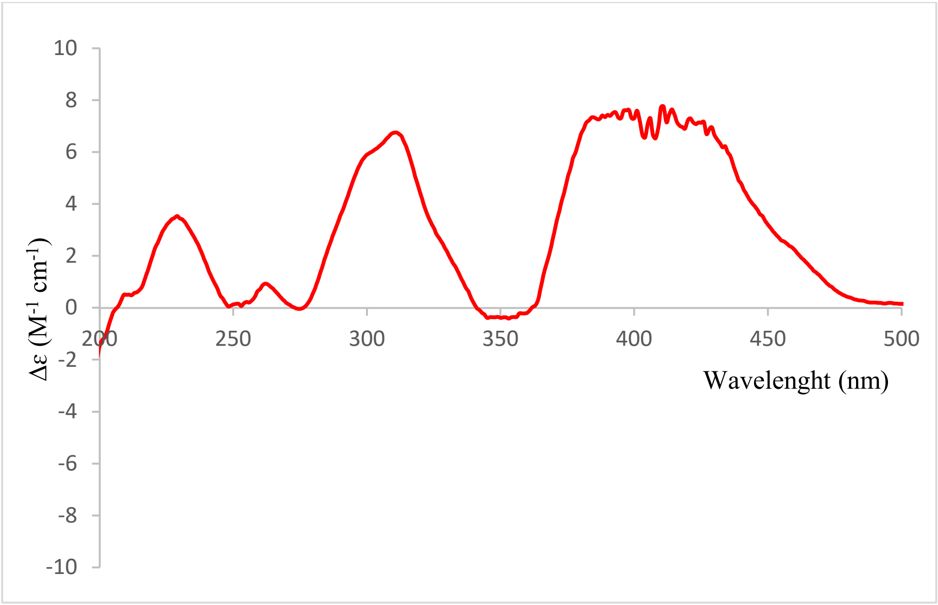

Figure S53: ECD spectrum (MeOH) of isochromophilonol A_1/A_2 (**7a+7b**)

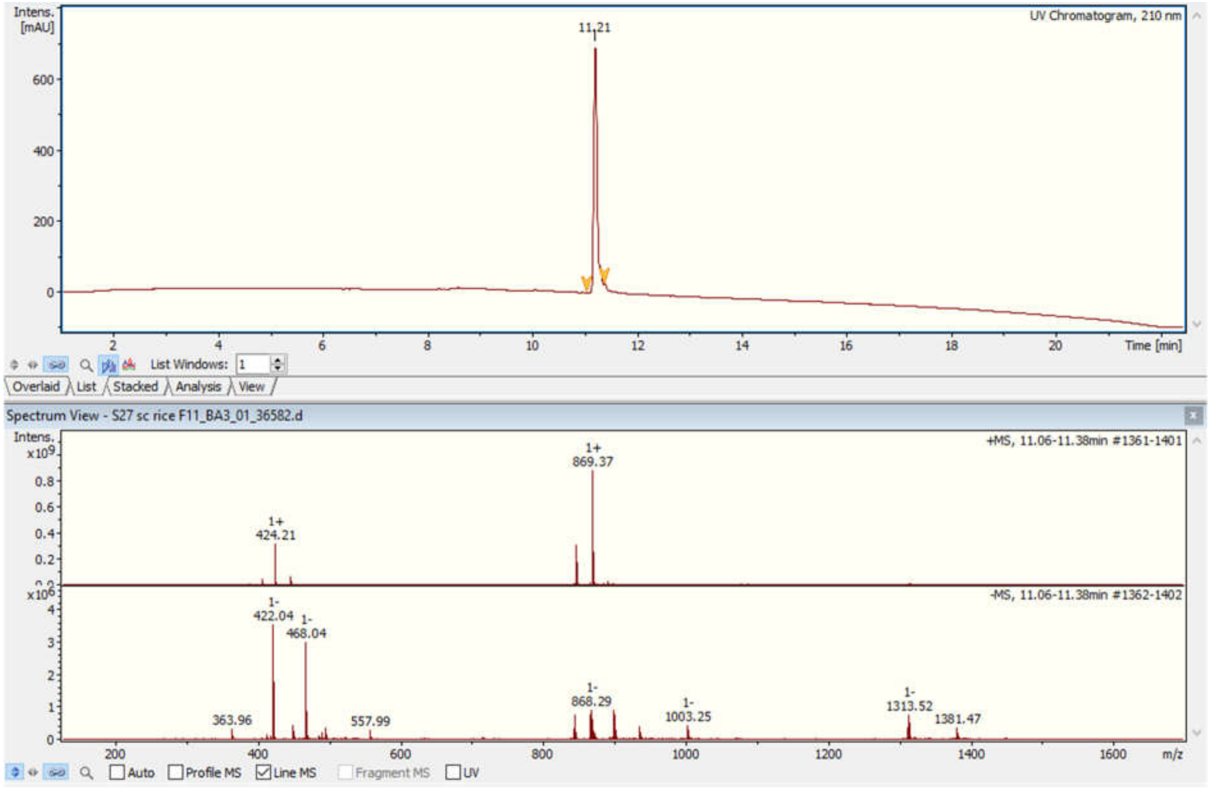

Figure S54: HPLC-DAD chromatogram and ESI-MS data for diaporisoindole A/B (**5**)

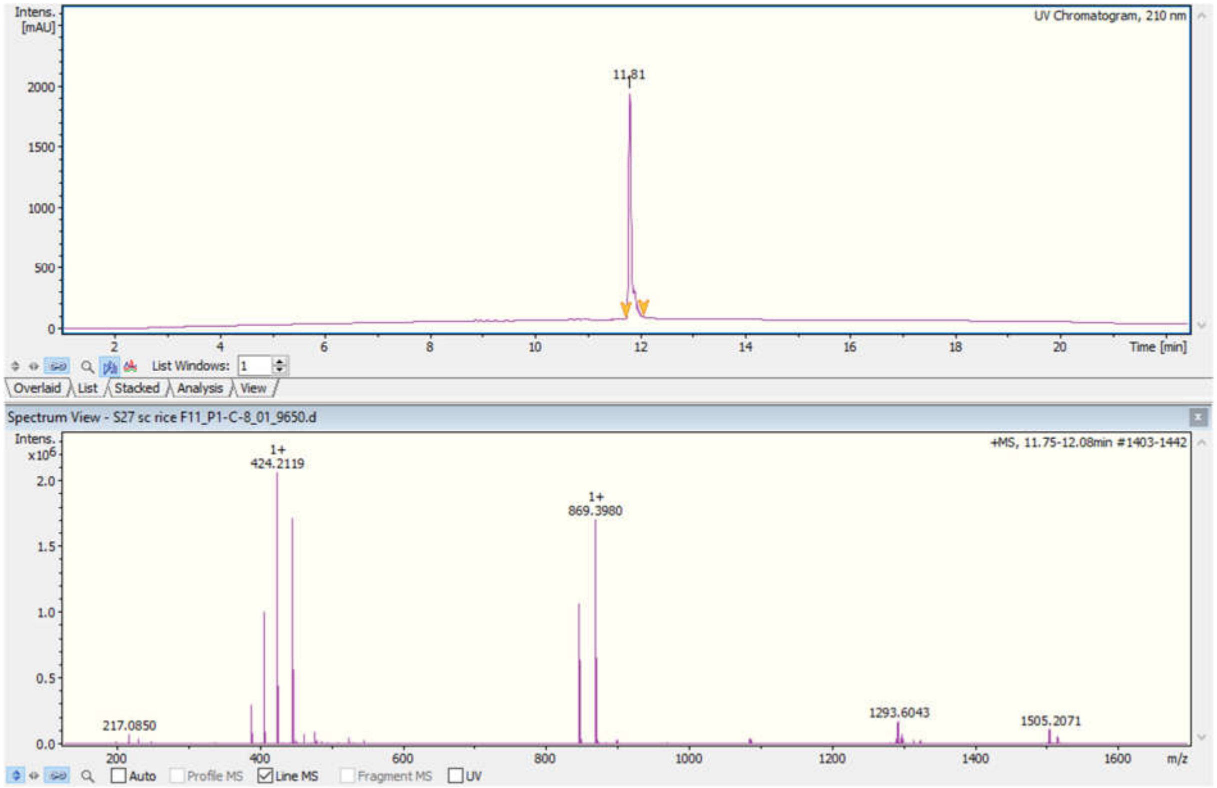

Figure S55: HPLC-DAD chromatogram and HR-ESI (+) MS data for diaporisoindole A/B (**5**)

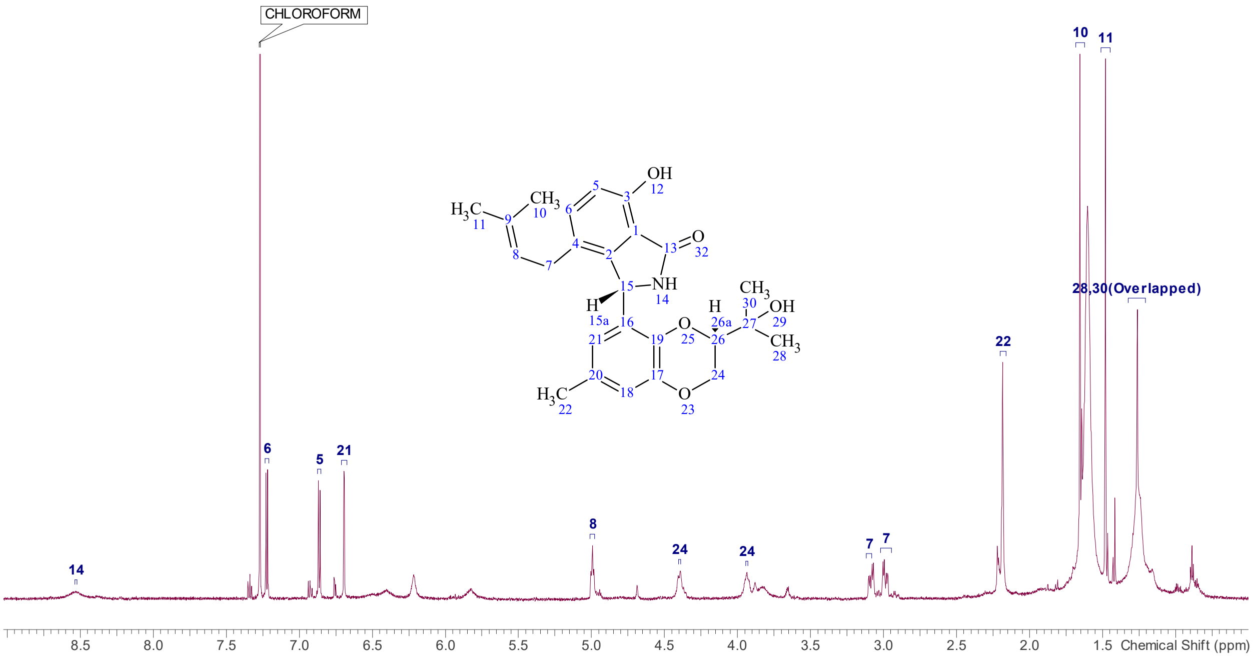

Figure S56: ^{1}H NMR spectrum ($CDCl_3$, 700 MHz) of diaporisoindole A/B (**5**)

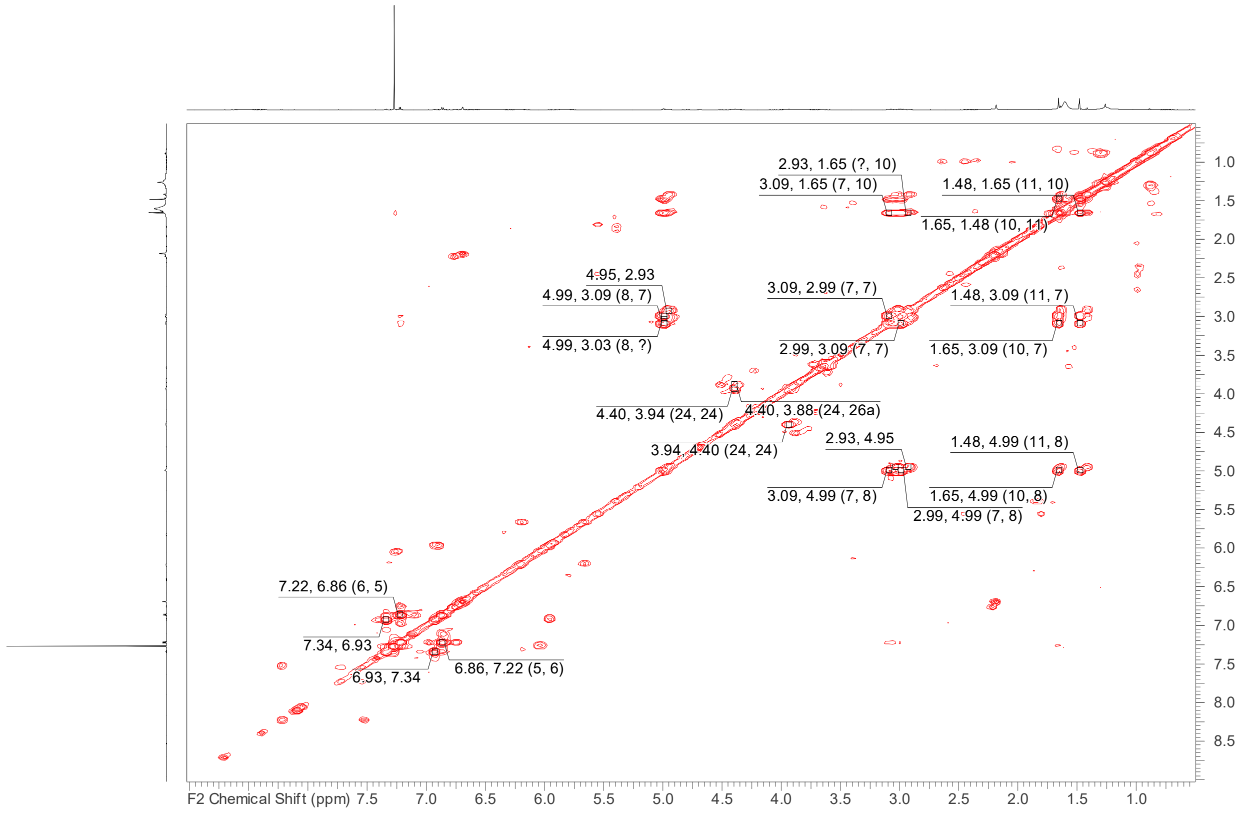

Figure S57: ^{1}H-^{1}H COSY NMR spectrum ($CDCl_3$, 700 MHz) of diaporisoindole A/B (**5**)

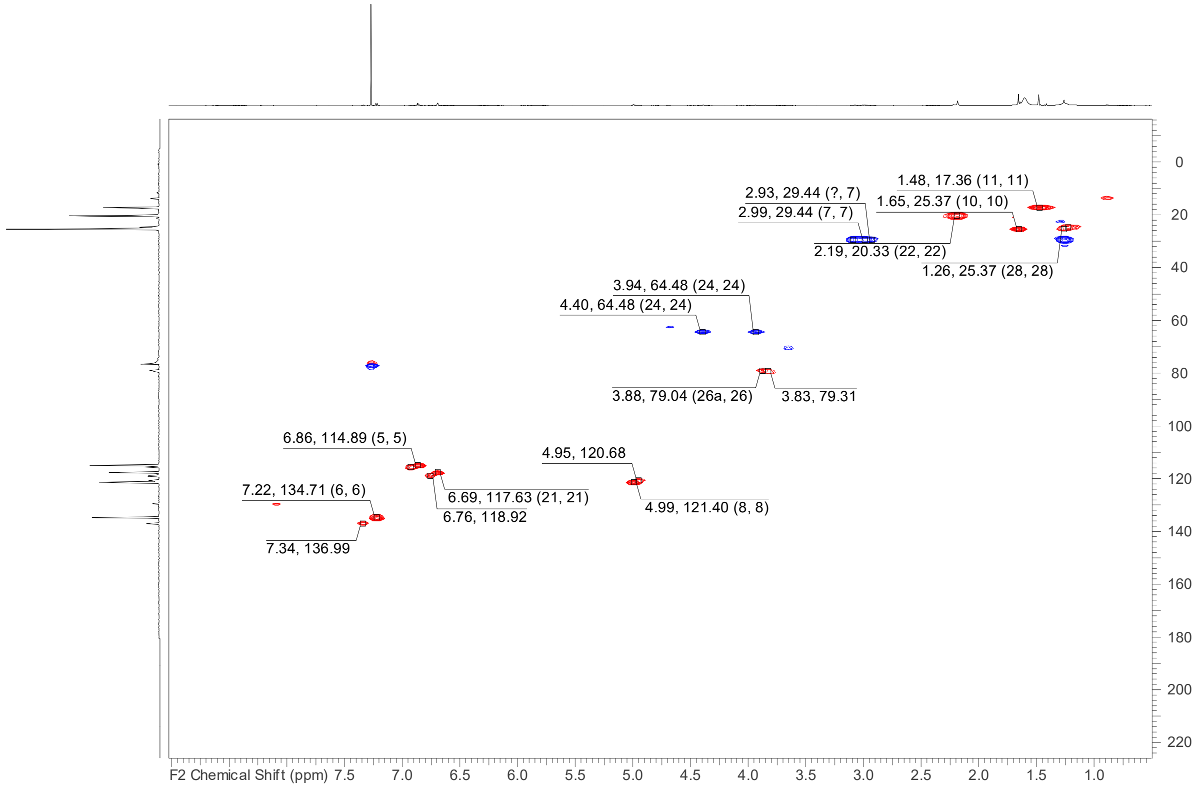

Figure S58: ^{1}H-^{13}C HSQC NMR spectrum ($CDCl_3$, 700 MHz) of diaporisoindole A/B (**5**)

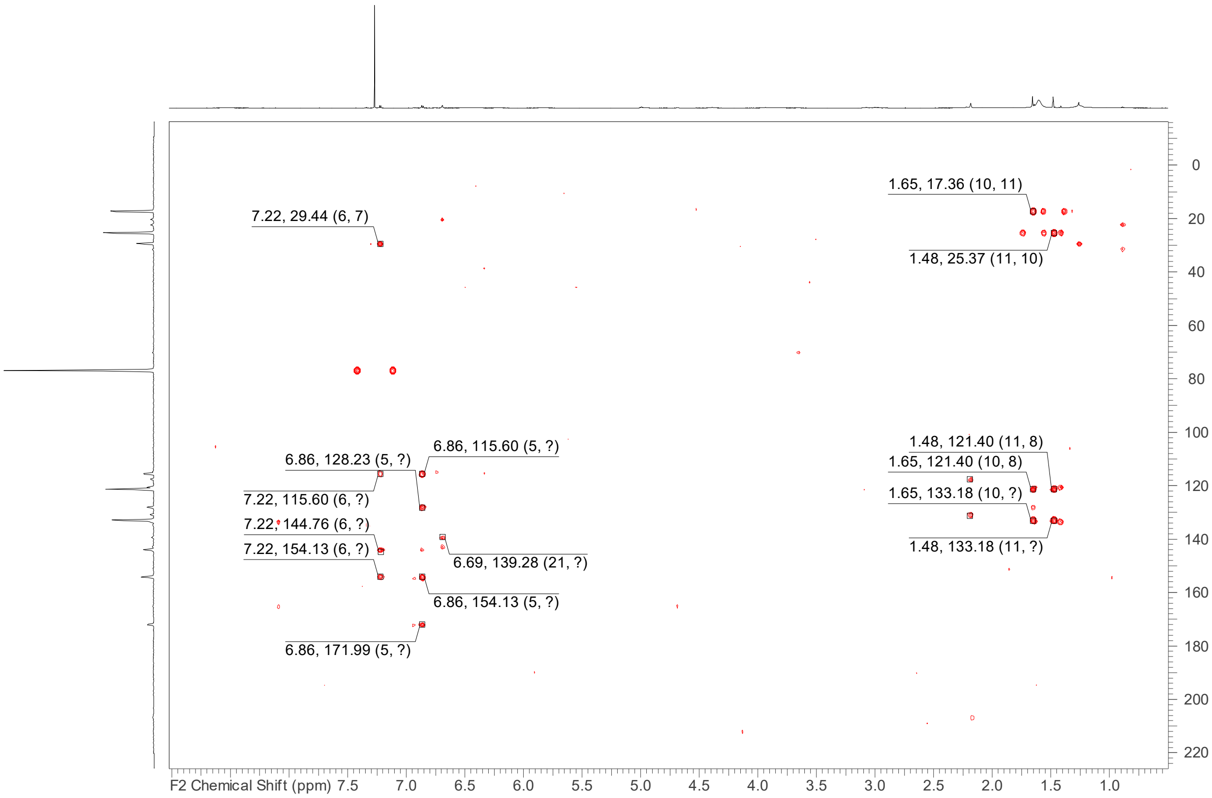

Figure S59: ^{1}H-^{13}C HMBC NMR spectrum ($CDCl_3$, 700 MHz) of diaporisoindole A/B (**5**)

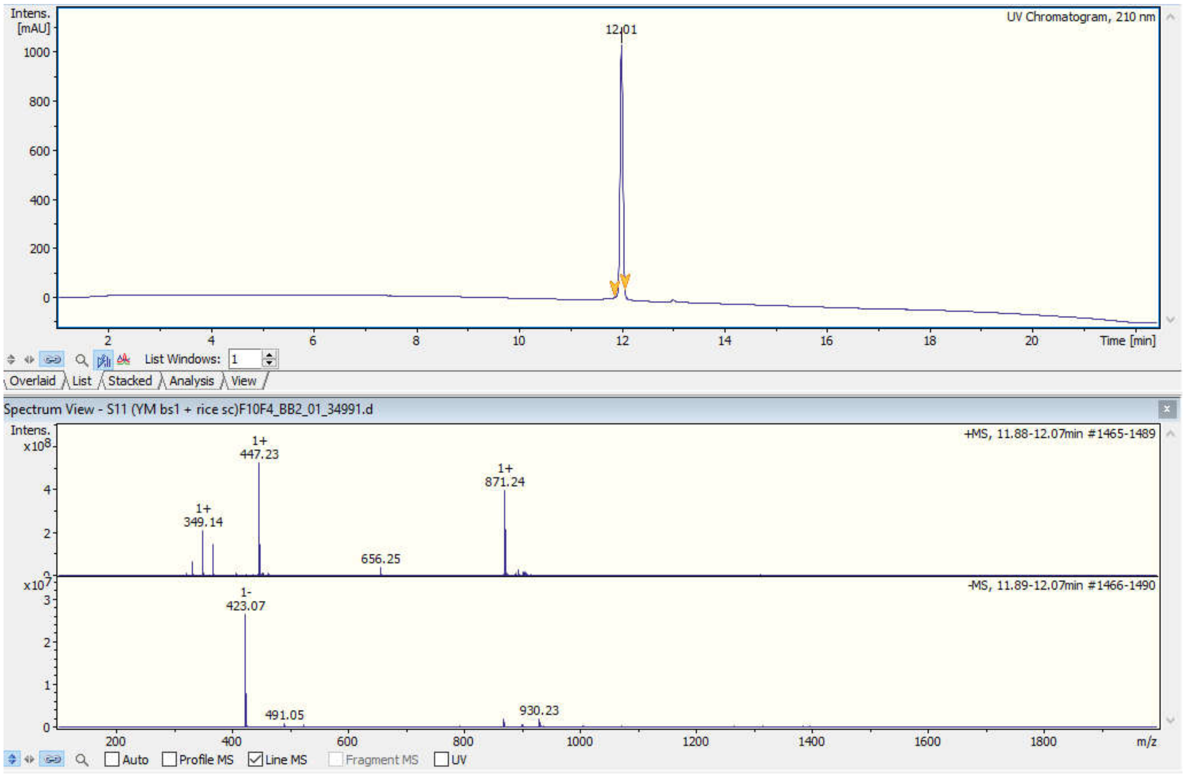

Figure S60: HPLC-DAD chromatogram and ESI-MS data for tenellone B (**6**)

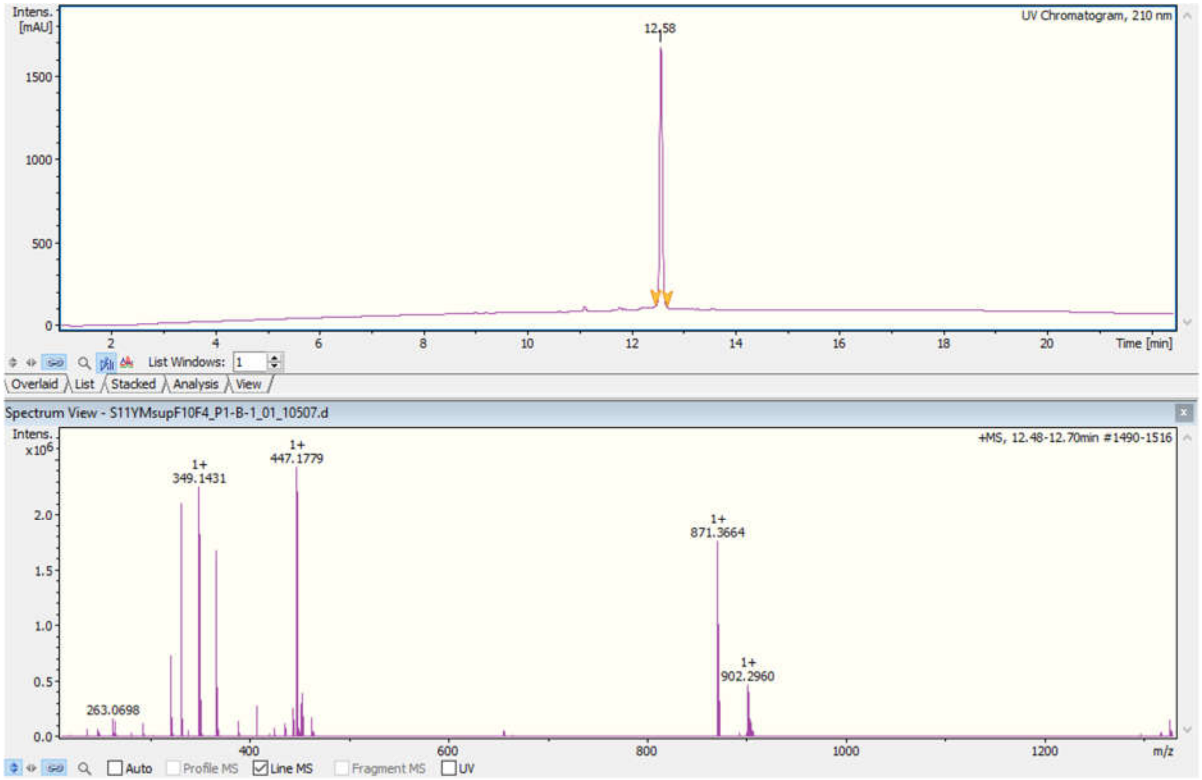

Figure S61: HPLC-DAD chromatogram and HR-ESI (+) MS data for tenellone B (**6**)

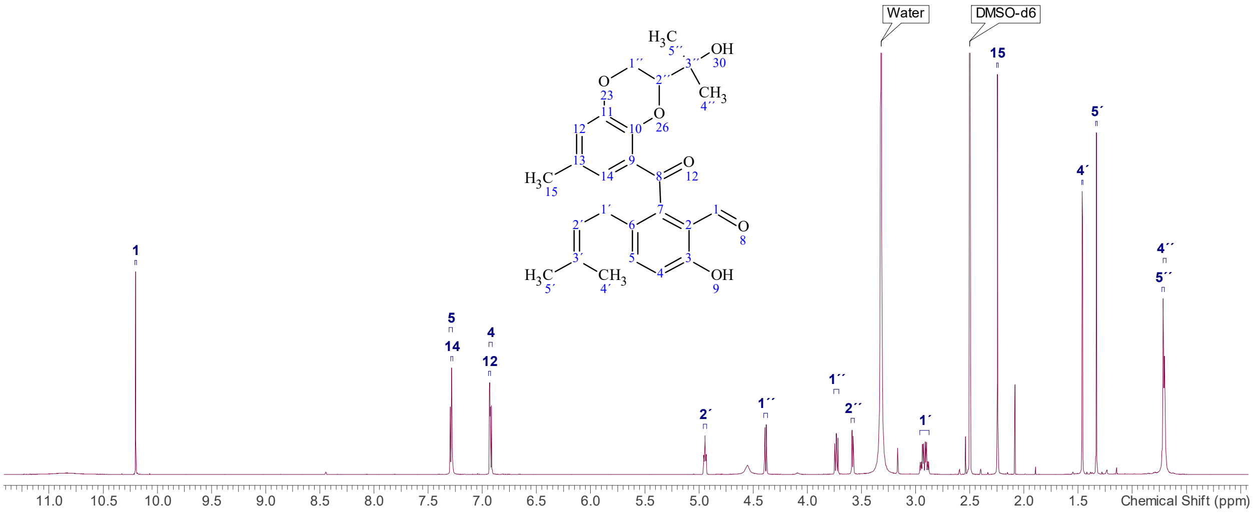

Figure S62: ^{1}H NMR spectrum (DMSO-d6, 700 MHz) of tenellone B (**6**)

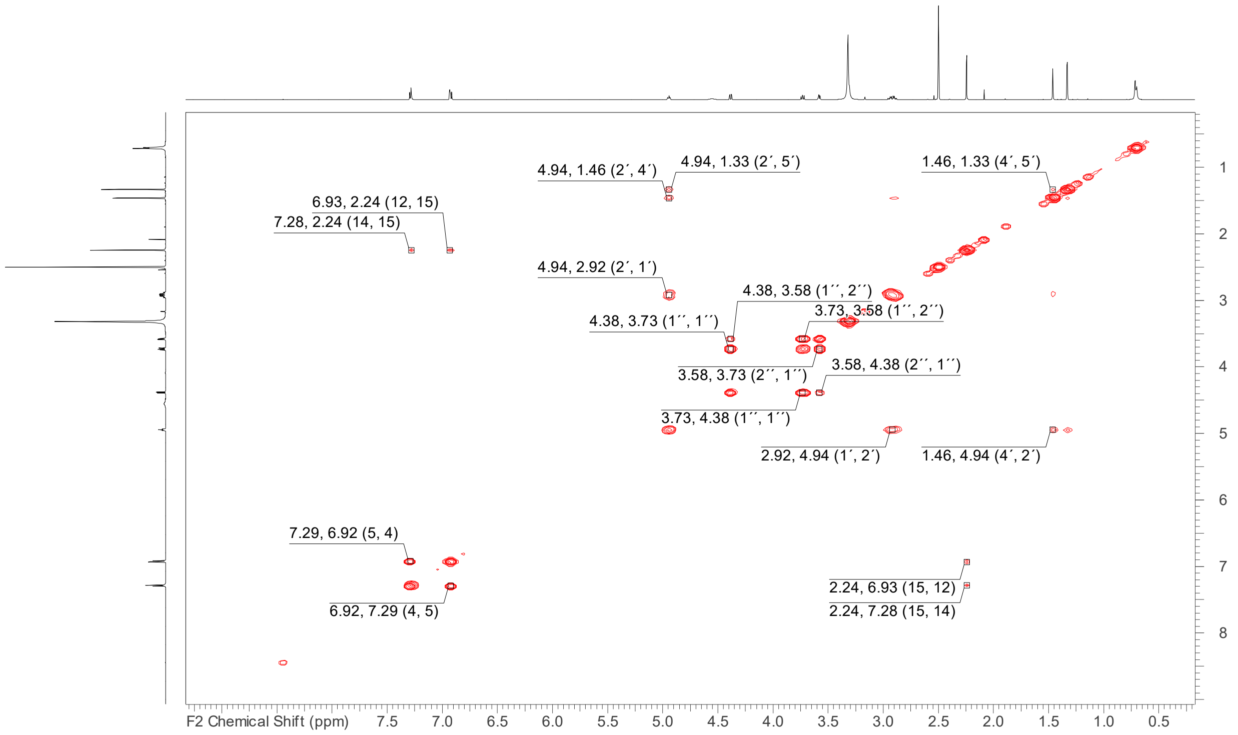

Figure S63: ^{1}H-^{1}H COSY NMR spectrum (DMSO-d_6, 700 MHz) of tenellone B (**6**)

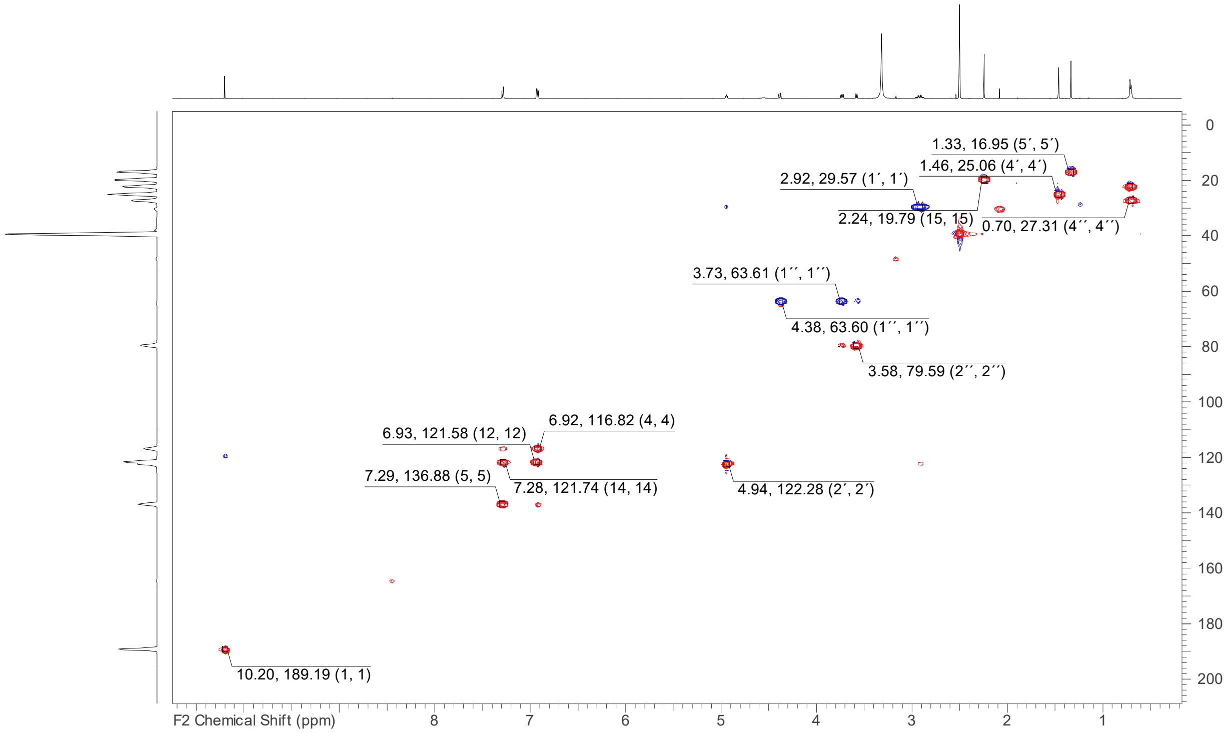

Figure S64: ^{1}H-^{13}C HSQC NMR spectrum (DMSO-d_6, 700 MHz) of tenellone B (**6**)

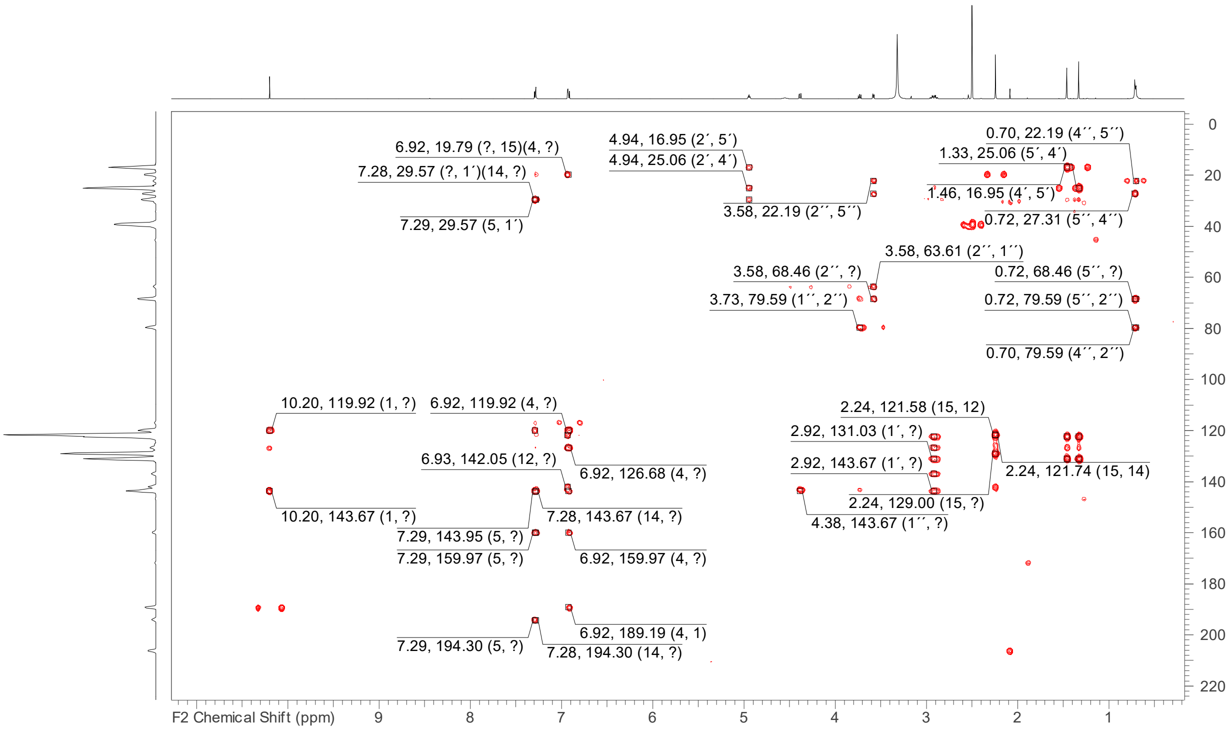

Figure S65: ^{1}H-^{13}C HMBC NMR spectrum (DMSO-d_6, 700 MHz) of tenellone B (**6**)

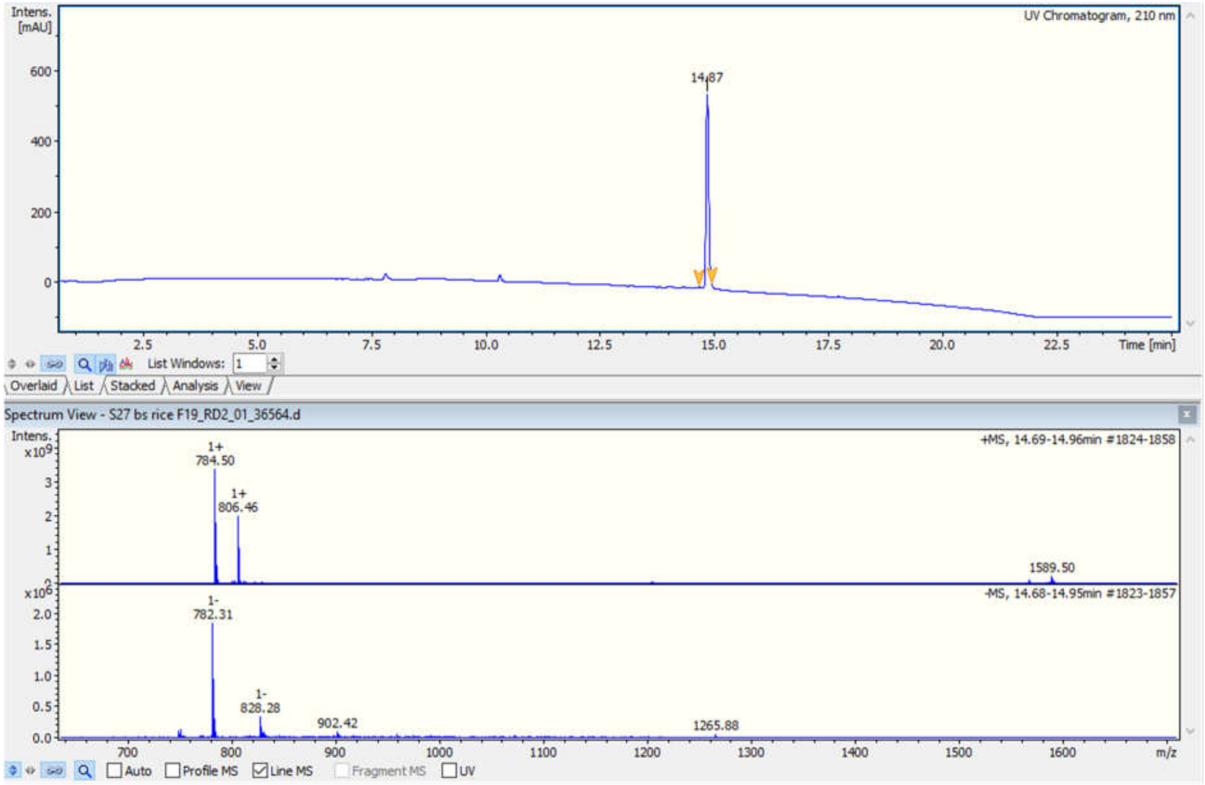

Figure S66: HPLC-DAD chromatogram and ESI-MS data for beauvericin (**8**)

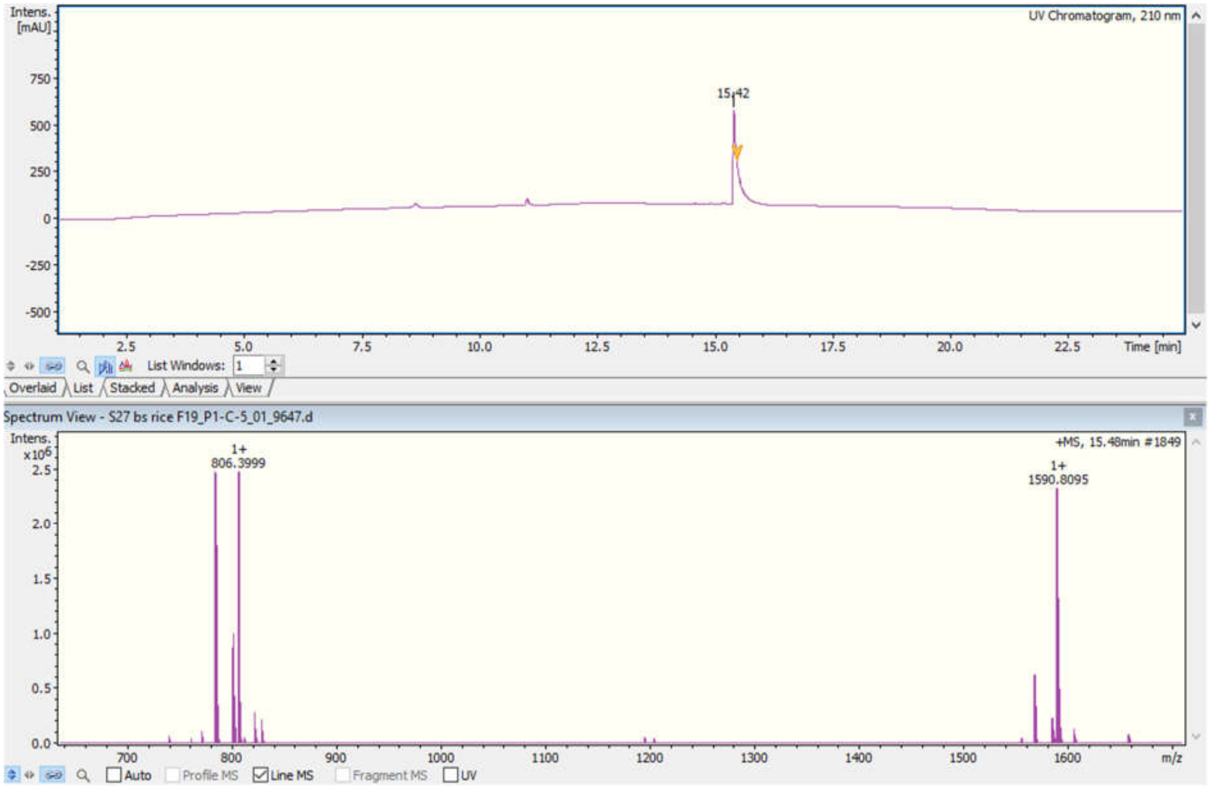

Figure S67: HPLC-DAD chromatogram and HR-ESI (+) MS data for beauvericin (**8**)

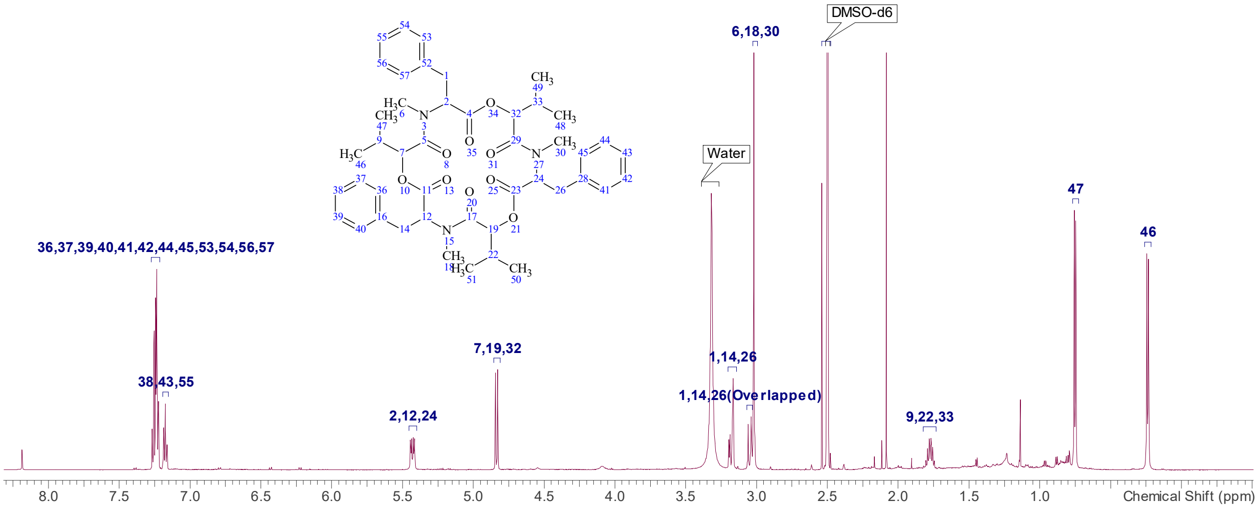

Figure S68: ¹H NMR spectrum (DMSO-d_6, 700 MHz) of beauvericin (**8**)

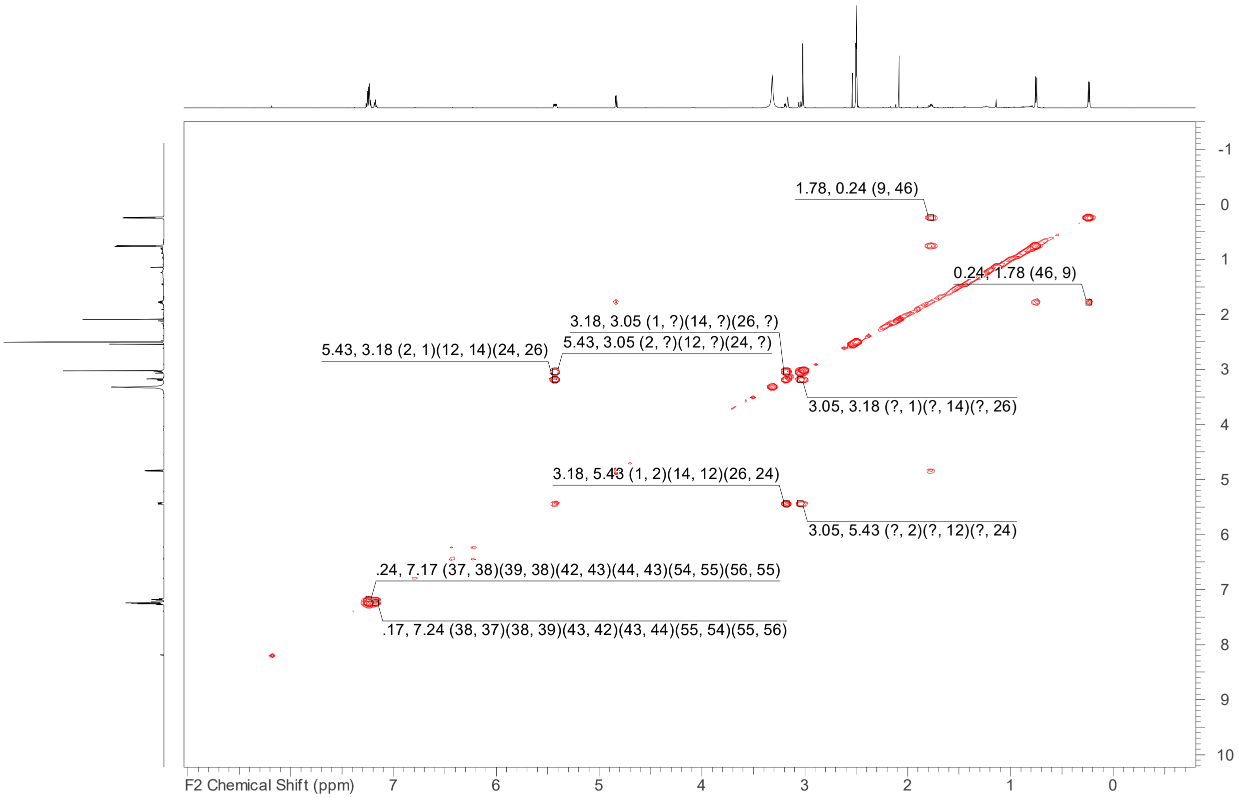

Figure S69: ^{1}H-^{1}H COSY NMR spectrum (DMSO-d_6, 700 MHz) of beauvericin (**8**)

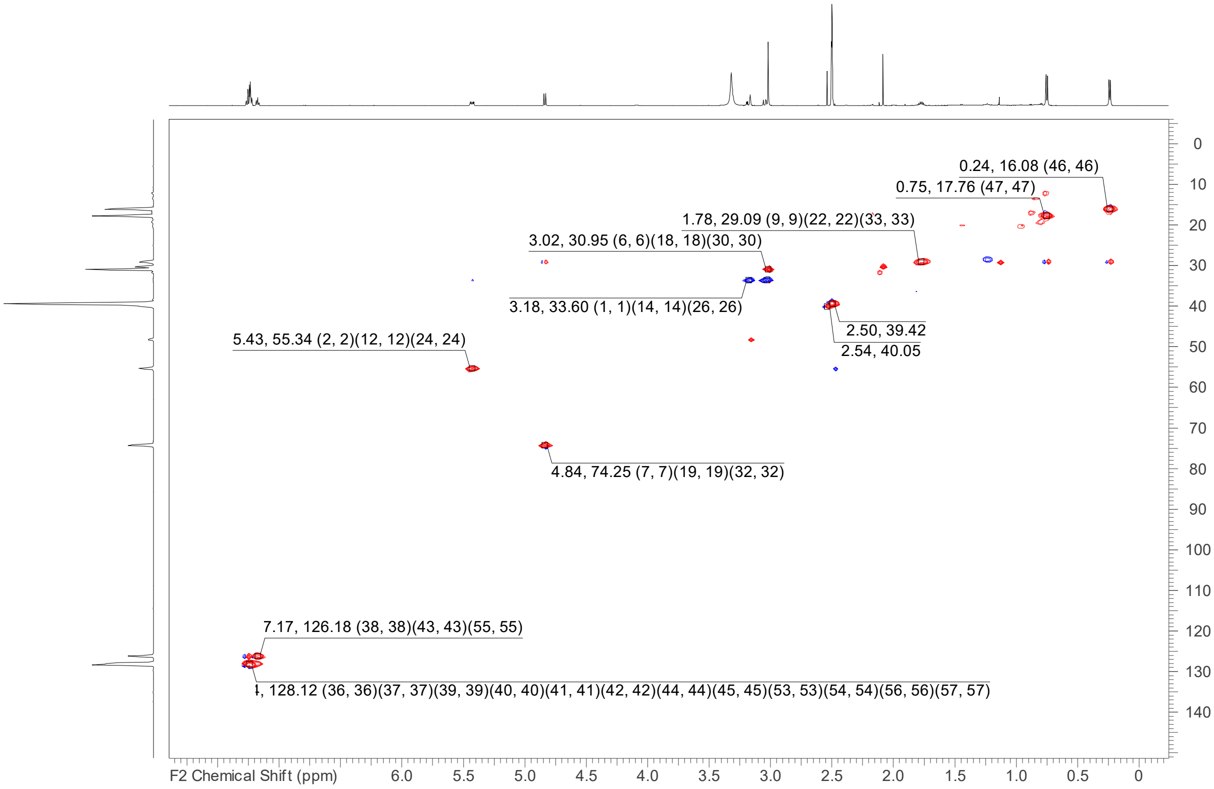

Figure S70: ^{1}H-^{13}C HSQC NMR spectrum (DMSO-d_6, 700 MHz) of beauvericin (**8**)

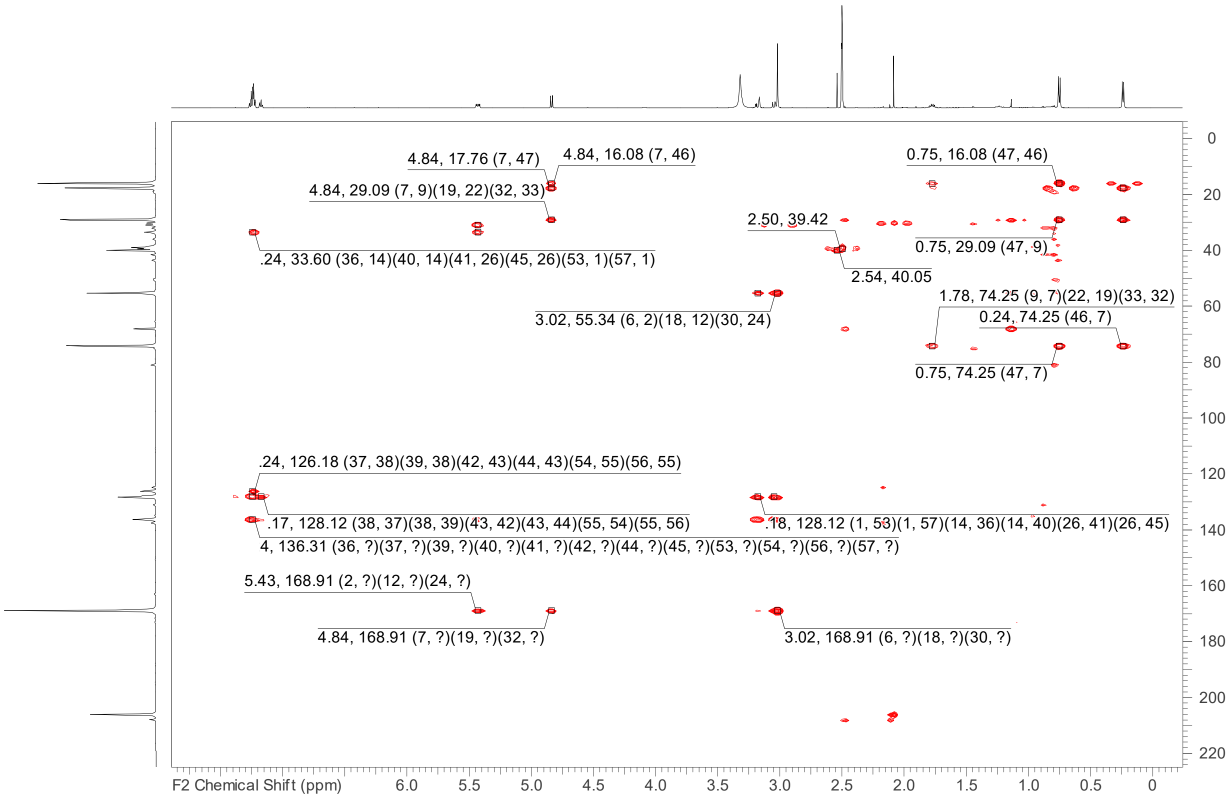

Figure S71: ^{1}H-^{13}C HMBC NMR spectrum (DMSO-d_6, 700 MHz) of beauvericin (**8**)

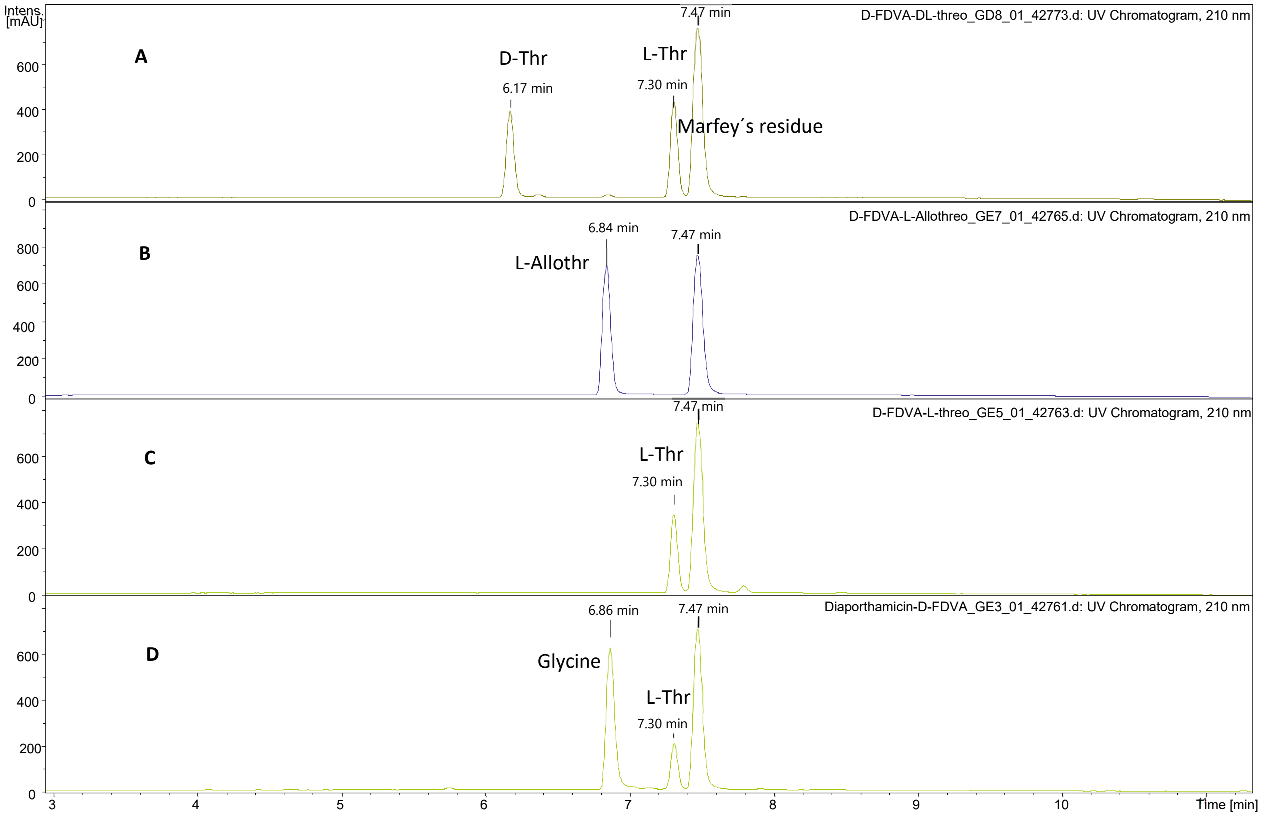

Figure S72: HPLC-DAD chromatograms of eucalactam B derived D-FDVA (**D**)/authentic amino acid derived D-FDVA. **A**) DL-threonine, **B**) L- Allothreonine, **C**) L-threonine.

Table S3: Retention time of L or D authentic amino acid derived D-FDVA

Nr.	Amino acid	Marfey's amino acid derivatives retention time (min)	Mass $[M+H]^+$
1.	L-Threonine	7.30	400.17
2.	D-Threonine	6.17	400.17
3.	L-AlloThreonine	6.84	400.17

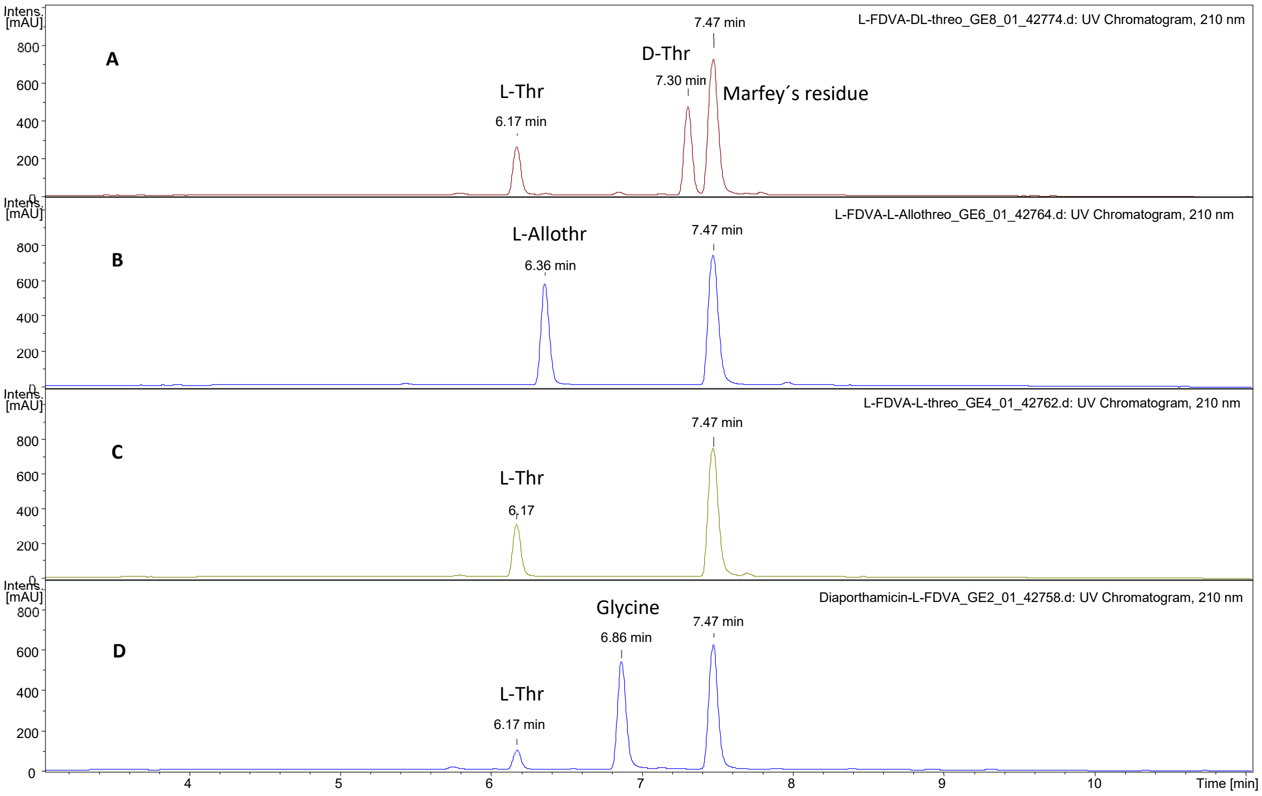

Figure S73: HPLC-DAD chromatograms of eucalactam B derived L-FDVA (**D**)/authentic amino acid derived L-FDVA. **A)** DL-threonine, **B)** L-Allothreonine, **C)** L-threonine.

Table S4: Retention time of L or D authentic amino acid derived L-FDVA

Nr.	Amino acid	Marfey's amino acid derivatives retention time (min)	Mass $[M+H]^+$
1.	L-Threonine	6.17	400.17
2.	D-Threonine	7.30	400.17
3.	L-AlloThreonine	6.36	400.17

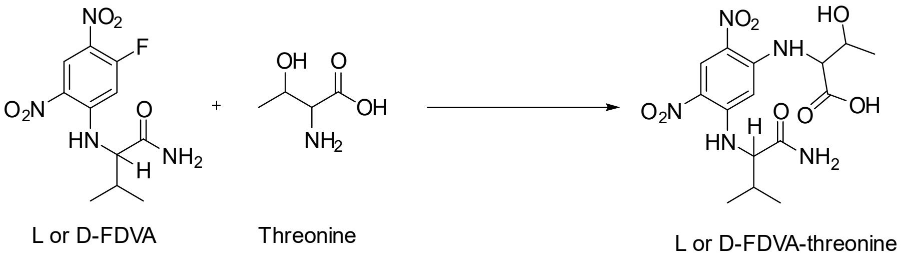

Figure S74: General Marfey´s reaction with threonine

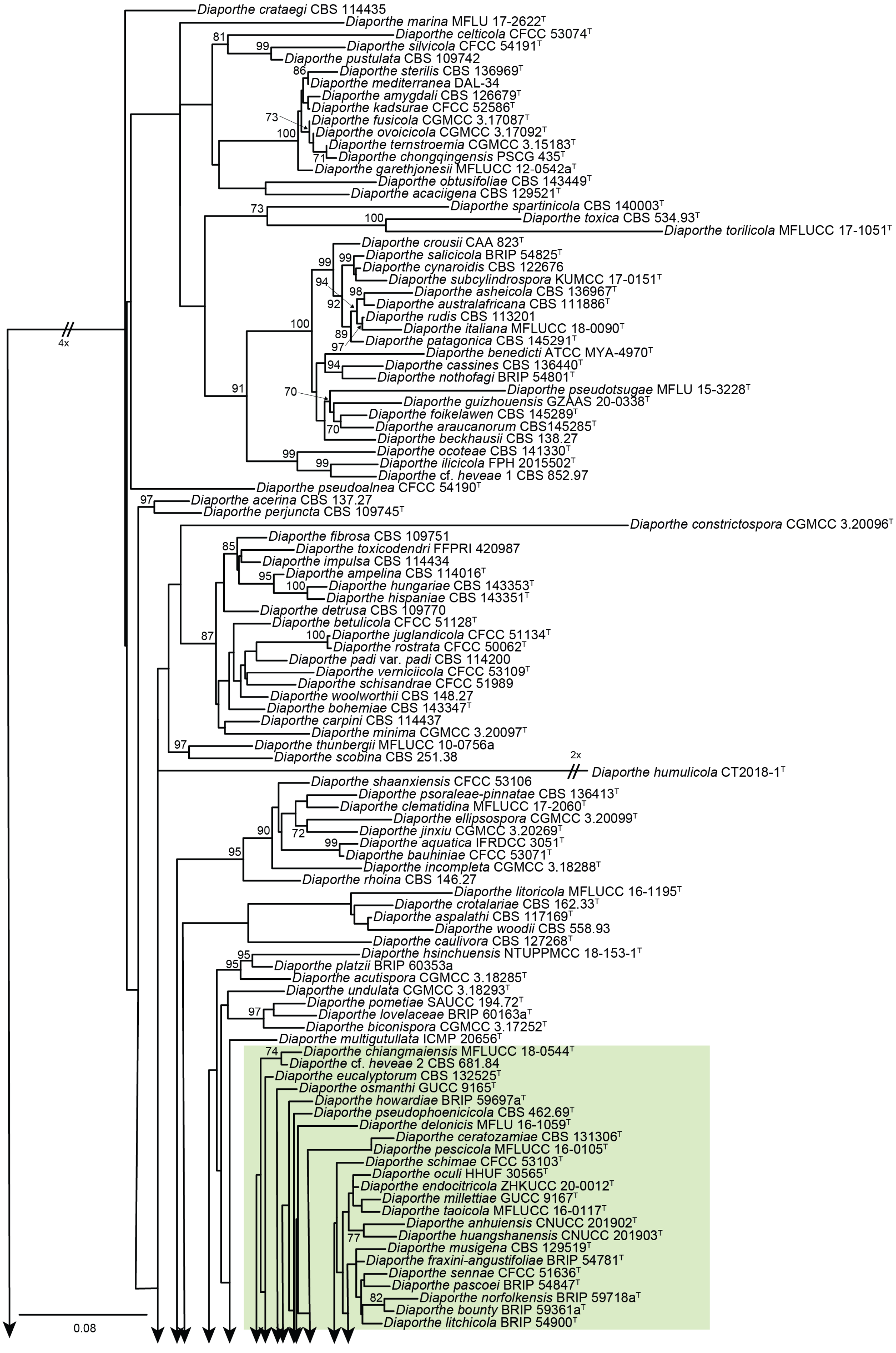
Diaporthe crataegi CBS 114435
Diaporthe marina MFLU 17-2622T
Diaporthe celticola CFCC 53074T
Diaporthe silvicola CFCC 54191T
Diaporthe pustulata CBS 109742
Diaporthe sterilis CBS 136969T
Diaporthe mediterranea DAL-34
Diaporthe amygdali CBS 126679T
Diaporthe kadsurae CFCC 52586T
Diaporthe fusicola CGMCC 3.17087T
Diaporthe ovoicicola CGMCC 3.17092T
Diaporthe ternstroemia CGMCC 3.15183T
Diaporthe chongqingensis PSCG 435T
Diaporthe garethjonesii MFLUCC 12-0542aT
Diaporthe obtusifoliae CBS 143449T
Diaporthe acaciigena CBS 129521T
Diaporthe spartinicola CBS 140003T
Diaporthe toxica CBS 534.93T
Diaporthe torilicola MFLUCC 17-1051T
Diaporthe crousii CAA 823T
Diaporthe salicicola BRIP 54825T
Diaporthe cynaroidis CBS 122676
Diaporthe subcylindrospora KUMCC 17-0151T
Diaporthe asheicola CBS 136967T
Diaporthe australafricana CBS 111886T
Diaporthe rudis CBS 113201
Diaporthe italiana MFLUCC 18-0090T
Diaporthe patagonica CBS 145291T
Diaporthe benedicti ATCC MYA-4970T
Diaporthe cassines CBS 136440T
Diaporthe nothofagi BRIP 54801T
Diaporthe pseudotsugae MFLU 15-3228T
Diaporthe guizhouensis GZAAS 20-0338T
Diaporthe foikelawen CBS 145289T
Diaporthe araucanorum CBS145285T
Diaporthe beckhausii CBS 138.27
Diaporthe ocoteae CBS 141330T
Diaporthe ilicicola FPH 2015502T
Diaporthe cf. heveae 1 CBS 852.97
Diaporthe pseudoalnea CFCC 54190T
Diaporthe acerina CBS 137.27
Diaporthe perjuncta CBS 109745T
Diaporthe constrictospora CGMCC 3.20096T
Diaporthe fibrosa CBS 109751
Diaporthe toxicodendri FFPRI 420987
Diaporthe impulsa CBS 114434
Diaporthe ampelina CBS 114016T
Diaporthe hungariae CBS 143353T
Diaporthe hispaniae CBS 143351T
Diaporthe detrusa CBS 109770
Diaporthe betulicola CFCC 51128T
Diaporthe juglandicola CFCC 51134T
Diaporthe rostrata CFCC 50062T
Diaporthe padi var. padi CBS 114200
Diaporthe verniciicola CFCC 53109T
Diaporthe schisandrae CFCC 51989
Diaporthe woolworthii CBS 148.27
Diaporthe bohemiae CBS 143347T
Diaporthe carpini CBS 114437
Diaporthe minima CGMCC 3.20097T
Diaporthe thunbergii MFLUCC 10-0756a
Diaporthe scobina CBS 251.38
Diaporthe humulicola CT2018-1T
Diaporthe shaanxiensis CFCC 53106
Diaporthe psoraleae-pinnatae CBS 136413T
Diaporthe clematidina MFLUCC 17-2060T
Diaporthe ellipsospora CGMCC 3.20099T
Diaporthe jinxiu CGMCC 3.20269T
Diaporthe aquatica IFRDCC 3051T
Diaporthe bauhiniae CFCC 53071T
Diaporthe incompleta CGMCC 3.18288T
Diaporthe rhoina CBS 146.27
Diaporthe litoricola MFLUCC 16-1195T
Diaporthe crotalariae CBS 162.33T
Diaporthe aspalathi CBS 117169T
Diaporthe woodii CBS 558.93
Diaporthe caulivora CBS 127268T
Diaporthe hsinchuensis NTUPPMCC 18-153-1T
Diaporthe platzii BRIP 60353a
Diaporthe acutispora CGMCC 3.18285T
Diaporthe undulata CGMCC 3.18293T
Diaporthe pometiae SAUCC 194.72T
Diaporthe lovelaceae BRIP 60163aT
Diaporthe biconispora CGMCC 3.17252T
Diaporthe multigutullata ICMP 20656T
Diaporthe chiangmaiensis MFLUCC 18-0544T
Diaporthe cf. heveae 2 CBS 681.84
Diaporthe eucalyptorum CBS 132525T
Diaporthe osmanthi GUCC 9165T
Diaporthe howardiae BRIP 59697aT
Diaporthe pseudophoenicicola CBS 462.69T
Diaporthe delonicis MFLU 16-1059T
Diaporthe ceratozamiae CBS 131306T
Diaporthe pescicola MFLUCC 16-0105T
Diaporthe schimae CFCC 53103T
Diaporthe oculi HHUF 30565T
Diaporthe endocitricola ZHKUCC 20-0012T
Diaporthe millettiae GUCC 9167T
Diaporthe taoicola MFLUCC 16-0117T
Diaporthe anhuiensis CNUCC 201902T
Diaporthe huangshanensis CNUCC 201903T
Diaporthe musigena CBS 129519T
Diaporthe fraxini-angustifoliae BRIP 54781T
Diaporthe sennae CFCC 51636T
Diaporthe pascoei BRIP 54847T
Diaporthe norfolkensis BRIP 59718aT
Diaporthe bounty BRIP 59361aT
Diaporthe litchicola BRIP 54900T
4x
2x
0.08

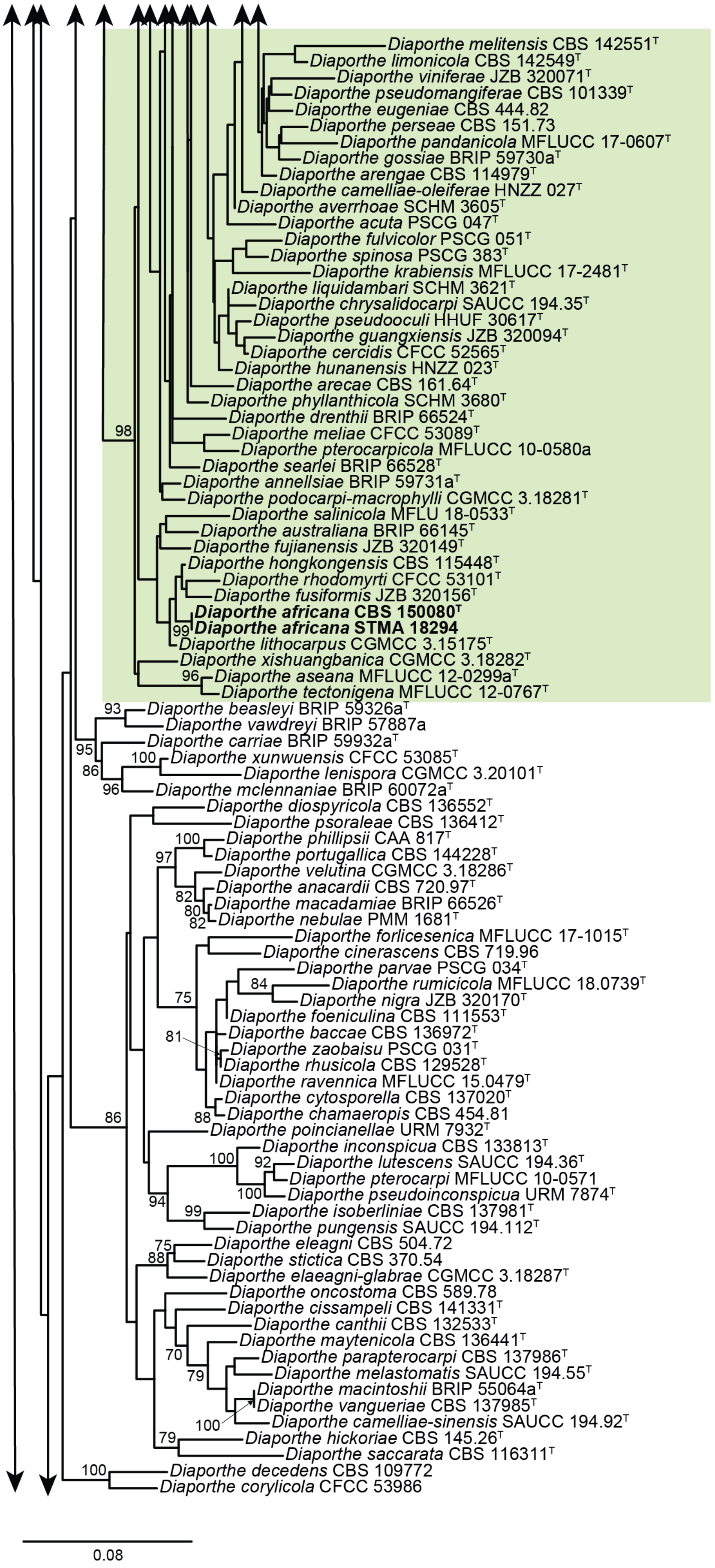
Diaporthe melitensis CBS 142551T
Diaporthe limonicola CBS 142549T
Diaporthe viniferae JZB 320071T
Diaporthe pseudomangiferae CBS 101339T
Diaporthe eugeniae CBS 444.82
Diaporthe perseae CBS 151.73
Diaporthe pandanicola MFLUCC 17-0607T
Diaporthe gossiae BRIP 59730aT
Diaporthe arengae CBS 114979T
Diaporthe camelliae-oleiferae HNZZ 027T
Diaporthe averrhoae SCHM 3605T
Diaporthe acuta PSCG 047T
Diaporthe fulvicolor PSCG 051T
Diaporthe spinosa PSCG 383T
Diaporthe krabiensis MFLUCC 17-2481T
Diaporthe liquidambari SCHM 3621T
Diaporthe chrysalidocarpi SAUCC 194.35T
Diaporthe pseudooculi HHUF 30617T
Diaporthe guangxiensis JZB 320094T
Diaporthe cercidis CFCC 52565T
Diaporthe hunanensis HNZZ 023T
Diaporthe arecae CBS 161.64T
Diaporthe phyllanthicola SCHM 3680T
Diaporthe drenthii BRIP 66524T
Diaporthe meliae CFCC 53089T
98
Diaporthe pterocarpicola MFLUCC 10-0580a
Diaporthe searlei BRIP 66528T
Diaporthe annellsiae BRIP 59731aT
Diaporthe podocarpi-macrophylli CGMCC 3.18281T
Diaporthe salinicola MFLU 18-0533T
Diaporthe australiana BRIP 66145T
Diaporthe fujianensis JZB 320149T
Diaporthe hongkongensis CBS 115448T
Diaporthe rhodomyrti CFCC 53101T
Diaporthe fusiformis JZB 320156T
Diaporthe africana CBS 150080T
99
Diaporthe africana STMA 18294
Diaporthe lithocarpus CGMCC 3.15175T
Diaporthe xishuangbanica CGMCC 3.18282T
96
Diaporthe aseana MFLUCC 12-0299aT
Diaporthe tectonigena MFLUCC 12-0767T
93
Diaporthe beasleyi BRIP 59326aT
Diaporthe vawdreyi BRIP 57887a
95
Diaporthe carriae BRIP 59932aT
100
Diaporthe xunwuensis CFCC 53085T
86
Diaporthe lenispora CGMCC 3.20101T
96
Diaporthe mclennaniae BRIP 60072aT
Diaporthe diospyricola CBS 136552T
Diaporthe psoraleae CBS 136412T
100
Diaporthe phillipsii CAA 817T
97
Diaporthe portugallica CBS 144228T
Diaporthe velutina CGMCC 3.18286T
82
Diaporthe anacardii CBS 720.97T
80
Diaporthe macadamiae BRIP 66526T
82
Diaporthe nebulae PMM 1681T
Diaporthe forlicesenica MFLUCC 17-1015T
Diaporthe cinerascens CBS 719.96
Diaporthe parvae PSCG 034T
84
Diaporthe rumicicola MFLUCC 18.0739T
75
Diaporthe nigra JZB 320170T
Diaporthe foeniculina CBS 111553T
81
Diaporthe baccae CBS 136972T
Diaporthe zaobaisu PSCG 031T
Diaporthe rhusicola CBS 129528T
Diaporthe ravennica MFLUCC 15.0479T
Diaporthe cytosporella CBS 137020T
88
Diaporthe chamaeropis CBS 454.81
86
Diaporthe poincianellae URM 7932T
100
Diaporthe inconspicua CBS 133813T
92
Diaporthe lutescens SAUCC 194.36T
Diaporthe pterocarpi MFLUCC 10-0571
100
Diaporthe pseudoinconspicua URM 7874T
94
99
Diaporthe isoberliniae CBS 137981T
Diaporthe pungensis SAUCC 194.112T
75
Diaporthe eleagni CBS 504.72
88
Diaporthe stictica CBS 370.54
Diaporthe elaeagni-glabrae CGMCC 3.18287T
Diaporthe oncostoma CBS 589.78
Diaporthe cissampeli CBS 141331T
Diaporthe canthii CBS 132533T
Diaporthe maytenicola CBS 136441T
70
Diaporthe parapterocarpi CBS 137986T
79
Diaporthe melastomatis SAUCC 194.55T
Diaporthe macintoshii BRIP 55064aT
Diaporthe vangueriae CBS 137985T
100
Diaporthe camelliae-sinensis SAUCC 194.92T
79
Diaporthe hickoriae CBS 145.26T
Diaporthe saccarata CBS 116311T
100
Diaporthe decedens CBS 109772
Diaporthe corylicola CFCC 53986
0.08

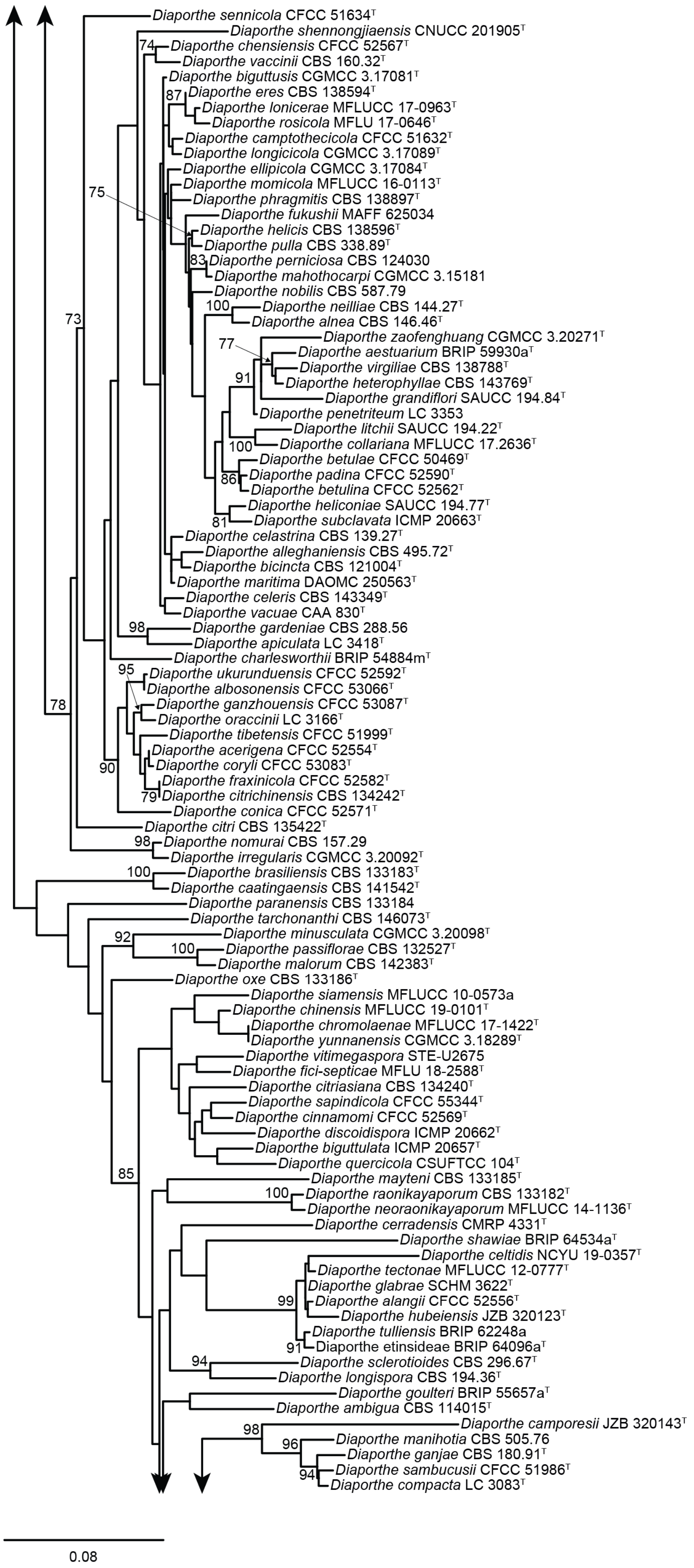

Diaporthe sennicola CFCC 51634T
Diaporthe shennongjiaensis CNUCC 201905T
Diaporthe chensiensis CFCC 52567T
Diaporthe vaccinii CBS 160.32T
Diaporthe biguttusis CGMCC 3.17081T
Diaporthe eres CBS 138594T
Diaporthe lonicerae MFLUCC 17-0963T
Diaporthe rosicola MFLU 17-0646T
Diaporthe camptothecicola CFCC 51632T
Diaporthe longicicola CGMCC 3.17089T
Diaporthe ellipicola CGMCC 3.17084T
Diaporthe momicola MFLUCC 16-0113T
Diaporthe phragmitis CBS 138897T
Diaporthe fukushii MAFF 625034
Diaporthe helicis CBS 138596T
Diaporthe pulla CBS 338.89T
Diaporthe perniciosa CBS 124030
Diaporthe mahothocarpi CGMCC 3.15181
Diaporthe nobilis CBS 587.79
Diaporthe neilliae CBS 144.27T
Diaporthe alnea CBS 146.46T
Diaporthe zaofenghuang CGMCC 3.20271T
Diaporthe aestuarium BRIP 59930aT
Diaporthe virgiliae CBS 138788T
Diaporthe heterophyllae CBS 143769T
Diaporthe grandiflori SAUCC 194.84T
Diaporthe penetriteum LC 3353
Diaporthe litchii SAUCC 194.22T
Diaporthe collariana MFLUCC 17.2636T
Diaporthe betulae CFCC 50469T
Diaporthe padina CFCC 52590T
Diaporthe betulina CFCC 52562T
Diaporthe heliconiae SAUCC 194.77T
Diaporthe subclavata ICMP 20663T
Diaporthe celastrina CBS 139.27T
Diaporthe alleghaniensis CBS 495.72T
Diaporthe bicincta CBS 121004T
Diaporthe maritima DAOMC 250563T
Diaporthe celeris CBS 143349T
Diaporthe vacuae CAA 830T
Diaporthe gardeniae CBS 288.56
Diaporthe apiculata LC 3418T
Diaporthe charlesworthii BRIP 54884mT
Diaporthe ukurunduensis CFCC 52592T
Diaporthe albosonensis CFCC 53066T
Diaporthe ganzhouensis CFCC 53087T
Diaporthe oraccinii LC 3166T
Diaporthe tibetensis CFCC 51999T
Diaporthe acerigena CFCC 52554T
Diaporthe coryli CFCC 53083T
Diaporthe fraxinicola CFCC 52582T
Diaporthe citrichinensis CBS 134242T
Diaporthe conica CFCC 52571T
Diaporthe citri CBS 135422T
Diaporthe nomurai CBS 157.29
Diaporthe irregularis CGMCC 3.20092T
Diaporthe brasiliensis CBS 133183T
Diaporthe caatingaensis CBS 141542T
Diaporthe paranensis CBS 133184
Diaporthe tarchonanthi CBS 146073T
Diaporthe minusculata CGMCC 3.20098T
Diaporthe passiflorae CBS 132527T
Diaporthe malorum CBS 142383T
Diaporthe oxe CBS 133186T
Diaporthe siamensis MFLUCC 10-0573a
Diaporthe chinensis MFLUCC 19-0101T
Diaporthe chromolaenae MFLUCC 17-1422T
Diaporthe yunnanensis CGMCC 3.18289T
Diaporthe vitimegaspora STE-U2675
Diaporthe fici-septicae MFLU 18-2588T
Diaporthe citriasiana CBS 134240T
Diaporthe sapindicola CFCC 55344T
Diaporthe cinnamomi CFCC 52569T
Diaporthe discoidispora ICMP 20662T
Diaporthe biguttulata ICMP 20657T
Diaporthe quercicola CSUFTCC 104T
Diaporthe mayteni CBS 133185T
Diaporthe raonikayaporum CBS 133182T
Diaporthe neoraonikayaporum MFLUCC 14-1136T
Diaporthe cerradensis CMRP 4331T
Diaporthe shawiae BRIP 64534aT
Diaporthe celtidis NCYU 19-0357T
Diaporthe tectonae MFLUCC 12-0777T
Diaporthe glabrae SCHM 3622T
Diaporthe alangii CFCC 52556T
Diaporthe hubeiensis JZB 320123T
Diaporthe tulliensis BRIP 62248a
Diaporthe etinsideae BRIP 64096aT
Diaporthe sclerotioides CBS 296.67T
Diaporthe longispora CBS 194.36T
Diaporthe goulteri BRIP 55657aT
Diaporthe ambigua CBS 114015T
Diaporthe camporesii JZB 320143T
Diaporthe manihotia CBS 505.76
Diaporthe ganjae CBS 180.91T
Diaporthe sambucusii CFCC 51986T
Diaporthe compacta LC 3083T
74
87
75
83
100
77
91
100
86
81
73
98
95
78
90
79
98
100
92
100
85
99
91
94
98
96
94
0.08

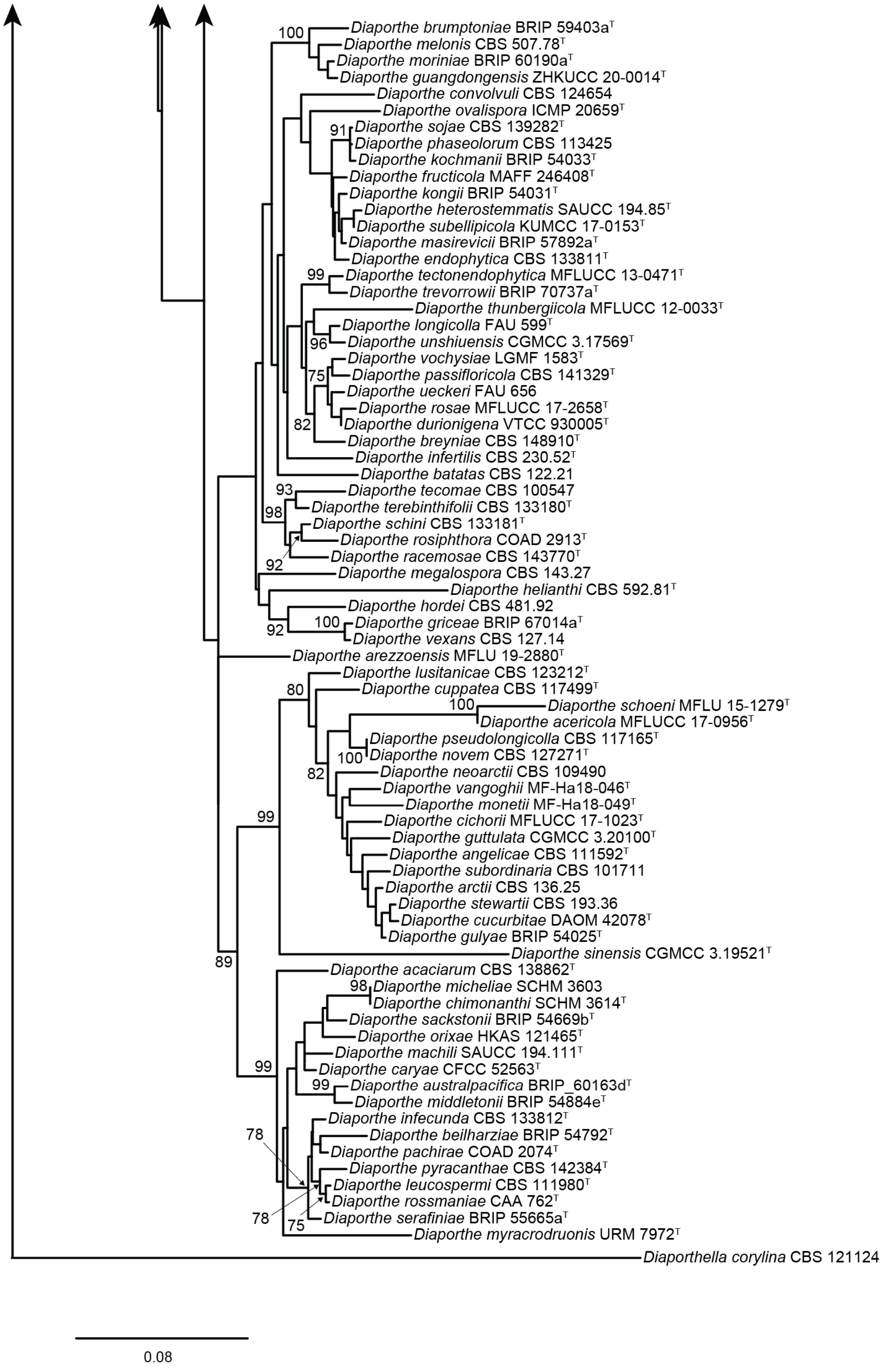

Figure S75: RAxML phylogram including our strains and type and reference strains of *Diaporthe* spp.

Table S5: GenBank accession numbers of the strains included in the broad phylogenetic study.

Species	Isolates[1]	GenBank accession numbers[2]				
		ITS	*tub2*	*his3*	*tef1*	*cal*
Diaporthe acaciarum	CBS 138862[T]	KP004460	KP004509	KP004504	-	-
D. acaciigena	CBS 129521[T]	KC343005	KC343973	KC343489	KC343731	KC343247
D. acericola	MFLUCC 17-0956[T]	KY964224	KY964074	-	KY964180	KY964137
D. acerigena	CFCC 52554[T]	MH121489	-	MH121449	MH121531	MH121413
D. acerina	CBS 137.27	KC343006	KC343974	KC343490	KC343732	KC343248
D. acuta	PSCG 047[T]	MK626957	MK691225	MK726161	MK654802	MK691125
D. acutispora	CGMCC 3.18285[T]	KX986764	KX999195	KX999235	KX999155	KX999274
D. aestuarium	BRIP 59930a[T]	OM918686	OM960613	-	OM960595	-
***D. africana* sp. nov.**	**CBS 150080[T]**	**OR198681**	**OR225229**	**OR225231**	**OR225227**	**OR225233**
	**STMA 18294**	**OR198680**	**OR225230**	**OR225232**	**OR225228**	**OR225234**
D. alangii	CFCC 52556[T]	MH121491	MH121573	MH121451	MH121533	MH121415
D. albosonensis	CFCC 53066[T]	MK432659	MK578059	MK443004	MK578133	MK442979
D. alleghaniensis	CBS 495.72[T]	FJ889444	KC843228	KC343491	GQ250298	KC343249
D. alnea	CBS 146.46[T]	KC343008	KC343976	KC343492	KC343734	KC343250
D. ambigua	CBS 114015[T]	KC343010	KC343978	KC343494	KC343736	KC343252
D. ampelina	CBS 114016[T]	AF230751	JX275452	-	GQ250351	JX197443
D. amygdali	CBS 126679[T]	KC343022	KC343990	KC343506	KC343748	KC343264
D. anacardii	CBS 720.97[T]	KC343024	KC343992	KC343508	KC343750	KC343266
D. angelicae	CBS 111592[T]	KC343026	KC343994	KC343511	KC343752	KC343268
D. anhuiensis	CNUCC 201902[T]	MN219727	MN227009	MN224550	MN224669	MN224556
D. annellsiae	BRIP 59731a[T]	OM918687	OM960614	-	OM960596	-
D. apiculata	LC 3418[T]	KP267896	KP293476	KP293550	KP267970	-

D. aquatica	IFRDCC 3051[T]	JQ797437	-	-	-	-
D. araucanorum	CBS 145285[T]	MN509711	MN509722	-	MN509733	MN974277
D. arctii	CBS 136.25	KC343031	KC343999	KC343515	KC343757	KC343273
D. arecae	CBS 161.64[T]	KC343032	KC344000	KC343516	KC343758	KC343274
D. arengae	CBS 114979[T]	KC343034	KC344002	KC343518	KC343760	KC343276
D. arezzoensis	MFLU 19-2880[T]	MT185503	MT454055	-	-	-
D. aseana	MFLUCC 12-0299a[T]	KT459414	KT459432	-	KT459448	KT459464
D. asheicola	CBS 136967[T]	KJ160562	KJ160518	-	KJ160594	KJ160542
D. aspalathi	CBS 117169[T]	KC343036	KC344004	KC343520	KC343762	KC343278
D. australafricana	CBS 111886[T]	KC343038	KC344006	KC343522	KC343764	KC343280
D. australiana	BRIP 66145[T]	MN708222	MN696530	-	MN696522	-
D. australpacifica	BRIP 60163d[T]	OM918688	OM960615	-	OM960597	-
D. averrhoae	SCHM 3605[T]	AY618930	-	-	-	-
D. baccae	CBS 136972[T]	KJ160565	MF418509	MF418264	KJ160597	-
D. batatas	CBS 122.21	KC343040	KC344008	KC343524	KC343766	KC343282
D. bauhiniae	CFCC 53071[T]	MK432648	MK578051	MK442995	MK578124	MK442970
D. beasleyi	BRIP 59326a[T]	OM918689	OM960616	-	OM960598	-
D. beckhausii	CBS 138.27	KC343041	KC344009	KC343525	KC343767	KC343283
D. beilharziae	BRIP 54792[T]	JX862529	KF170921	-	JX862535	-
D. benedicti	ATCC MYA-4970[T]	KM669929	-	-	KM669785,	KM669862
D. betulae	CFCC 50469[T]	KT732950	KT733020	KT732999	KT733016	KT732997
D. betulicola	CFCC 51128[T]	KX024653	KX024657	KX024661	KX024655	KX024659
D. betulina	CFCC 52562[T]	MH121497	MH121579	MH121457	MH121539	MH121421
D. bicincta	CBS 121004[T]	KC343134	KC344102	KC343618	KC343860	KC343376
D. biconispora	CGMCC 3.17252[T]	KJ490597	KJ490418	KJ490539	KJ490476	-
D. biguttulata	ICMP 20657[T]	KJ490582	KJ490403	KJ490524	KJ490461	-

D. biguttusis	CGMCC 3.17081[T]	KF576282	KF576306	-	KF576257	-
D. bohemiae	CBS 143347[T]	MG281015	MG281188	MG281361	MG281536	MG281710
D. bounty	BRIP 59361a[T]	OM918690	OM960617	-	OM960599	-
D. brasiliensis	CBS 133183[T]	KC343042	KC344010	KC343526	KC343768	KC343284
D. breyniae	CBS 148910[T]	ON400846	ON409186	ON409187	ON409188	ON409189
D. brumptoniae	BRIP 59403a[T]	OM918702	OM960629	-	OM960611	-
D. caatingaensis	CBS 141542[T]	KY085927	KY115600	KY115605	KY115603	KY115597
D. camelliae-oleiferae	HNZZ 027[T]	MZ509555	MZ504718	MZ504696	MZ504707	MZ504685
D. camelliae-sinensis	SAUCC 194.92[T]	MT822620	MT855817	MT855588	MT855932	MT855699
D. camporesii	JZB 320143[T]	MN533805	MN561316	-	-	-
D. camptothecicola	CFCC 51632[T]	KY203726	KY228893	KY228881	KY228887	KY228877
D. canthii	CBS 132533[T]	JX069864	KC843230	-	KC843120	KC843174
D. carpini	CBS 114437	KC343044	KC344012	KC343528	KC343770	KC343286
D. carriae	BRIP 59932a[T]	OM918691	OM960618	-	OM960600	-
D. caryae	CFCC 52563[T]	MH121498	MH121580	MH121458	MH121540	MH121422
D. cassines	CBS 136440[T]	KF777155	-	-	KF777244	-
D. caulivora	CBS 127268[T]	KC343045	KC344013	KC343529	KC343771	KC343287
D. celastrina	CBS 139.27[T]	KC343047	KC344015	KC343531	KC343773	KC343289
D. celeris	CBS 143349[T]	MG281017	MG281190	MG281363	MG281538	MG281712
D. celticola	CFCC 53074[T]	MK573948	MK574643	MK574603	MK574623	MK574587
D. celtidis	NCYU 19-0357[T]	MW114346	MW148266	-	MW192209	-
D. ceratozamiae	CBS 131306[T]	JQ044420	-	-	-	-
D. cercidis	CFCC 52565[T]	MH121500	MH121582	MH121460	MH121542	MH121424
D. cerradensis	CMRP 4331[T]	MN173198	MW751671	MW751663	MT311685	MW751655

D. cf. *heveae* 1	CBS 852.97	KC343116	KC344084	KC343600	KC343842	KC343358
D. cf. *heveae* 2	CBS 681.84	KC343117	KC344085	KC343601	KC343843	KC343359
D. chamaeropis	CBS 454.81	KC343048	KC344016	KC343532	KC343774	KC343290
D. charlesworthii	BRIP 54884m^{T}	KJ197288	KJ197268	-	KJ197250	-
D. chensiensis	CFCC 52567^{T}	MH121502	MH121584	MH121462	MH121544	MH121426
D. chiangmaiensis	MFLUCC 18-0544^{T}	OK393703	-	-	OL439483	-
D. chimonanthi	SCHM 3614^{T}	AY622993				
D. chinensis	MFLUCC 19-0101^{T}	MW187324	MW245013	-	MW205017	MW294199
D. chongqingensis	PSCG 435^{T}	MK626916	MK691321	MK726257	MK654866	MK691209
D. chromolaenae	MFLUCC 17-1422^{T}	MH094275	-	-	-	-
D. chrysalidocarpi	SAUCC 194.35^{T}	MT822563	MT855760	MT855532	MT855876	MT855646
D. cichorii	MFLUCC 17-1023^{T}	KY964220	KY964104	-	KY964176	KY964133
D. cinnamomi	CFCC 52569^{T}	MH121504	MH121586	MH121464	MH121546	-
D. cinerascens	CBS 719.96	KC343050	KC344018	KC343534	KC343776	KC343292
D. cissampeli	CBS 141331^{T}	KX228273	KX228384	KX228366	-	-
D. citri	CBS 135422^{T}	KC843311	KC843187	MF418281	KC843071	KC843157
D. citriasiana	CBS 134240^{T}	JQ954645	KC357459	MF418282	JQ954663	KC357491
D. citrichinensis	CBS 134242^{T}	JQ954648	MF418524	KJ420880	JQ954666	KC357494
D. clematidina	MFLUCC 17-2060^{T}	MT310657	MT394623	-	MT394669	MT394624
D. collariana	MFLUCC 17-2636^{T}	MG806115	MG783041	-	MG783040	MG783042
D. compacta	LC3083^{T}	KP267854	KP293434	KP293508	KP267928	-
D. conica	CFCC 52571^{T}	MH121506	MH121588	MH121466	MH121548	MH121428
D. constrictospora	CGMCC 3.20096^{T}	MT385947	MT424702	MW022487	-	MT424718
D. convolvuli	CBS 124654	KC343054	KC344022	KC343538	KC343780	KC343296
D. coryli	CFCC 53083^{T}	MK432661	MK578061	MK443006	MK578135	MK442981
D. corylicola	CFCC 53986	MW839880	MW883977	MW836717	MW815894	MW836684
D. crataegi	CBS 114435	KC343055	KC344023	KC343539	KC343781	KC343297

D. crotalariae	CBS 162.33[T]	KC343056	KC344024	KC343540	KC343782	KC343298
D. crousii	CAA823[T]	MK792311	MK837932	MK871450	MK828081	MK883835
D. cucurbitae	DAOM 42078[T]	KM453210	KP118848	KM453212	KM453211	-
D. cuppatea	CBS 117499[T]	AY339322	JX275420	KC343541	AY339354	JX197414
D. cynaroidis	CBS 122676	KC343058	KC344026	KC343542	KC343784	KC343300
D. cytosporella	CBS 137020[T]	KC843307	KC843221	MF418283	KC843116	KC843141
D. decedens	CBS 109772	KC343059	KC344027	KC343543	KC343785	KC343301
D. delonicis	MFLU 16-1059[T]	MT215490	MT212209	-	-	-
D. detrusa	CBS 109770	KC343061	KC344029	KC343545	KC343787	KC343303
D. diospyricola	CBS 136552[T]	KF777156	-	-	-	-
D. discoidispora	ICMP 20662[T]	KJ490624	KJ490445	KJ490566	KJ490503	-
D. drenthii	BRIP 66524[T]	MN708229	MN696537	-	MN696526	-
D. durionigena	VTCC 930005[T]	MN453530	MT276159	-	MT276157	-
D. elaeagni-glabrae	CGMCC 3.18287[T]	KX986779	KX999212	KX999251	KX999171	KX999281
D. eleagni	CBS 504.72	KC343064	KC344032	KC343548	KC343790	KC343306
D. ellipicola	CGMCC 3.17084[T]	KF576270	KF576291	-	KF576245	-
D. ellipsospora	CGMCC 3.20099[T]	MT385949	MT424704	MW022488	MT424684	MT424720
D. endocitricola	ZHKUCC 20-0012 [T]	MT355682	MT409290	-	MT409336	MT409312
D. endophytica	CBS 133811[T]	KC343065	KC344033	KC343549	KC343791	KC343307
D. eres	CBS 138594[T]	KJ210529	KJ420799	KJ420850	KJ210550	KJ434999
D. etinsideae	BRIP 64096a[T]	OM918692	OM960619	-	OM960601	-
D. eucalyptorum	CBS 132525[T]	JX069862	-	-	-	-
D. eugeniae	CBS 444.82	KC343098	KC344066	KC343582	KC343824	KC343340
D. fibrosa	CBS 109751	KC343099	KC344067	KC343583	KC343825	KC343341
D. fici-septicae	MFLU 18-2588[T]	MW114348	MW148268	-	MW192211	-
D. foeniculina	CBS 111553[T]	KC343101	KC344069	KC343585	KC343827	KC343343
D. foikelawen	CBS 145289[T]	MN509713	MN509724	-	MN509735	MN974278

D. forlicesenica	MFLUCC 17-1015^{T}	KY964215	KY964099	-	KY964171	-
D. fraxini-angustifoliae	BRIP 54781^{T}	JX862528	KF170920	-	JX852534	-
D. fraxinicola	CFCC 52582^{T}	MH121517	-	-	MH121559	MH121435
D. fructicola	MAFF 246408^{T}	LC342734	LC342736	LC342737	LC342735	LC342738
D. fujianensis	JZB 320149^{T}	MW010212	MW056008	-	MW20523	MW205212
D. fukushii	MAFF 625034	JQ807469	-	-	JQ807418	-
D. fulvicolor	PSCG 051^{T}	MK626859	MK691236	MK726163	MK654806	MK691132
D. fusicola	CGMCC 3.17087^{T}	KF576281	KF576305	-	KF576256	KF576233
D. fusiformis	JZB 320156^{T}	MW010218	MW056014	-	MW205234	MW205218
D. ganjae	CBS 180.91^{T}	KC343112	KC344080	KC343596	KC343838	KC343354
D. ganzhouensis	CFCC 53087^{T}	MK432665	MK578065	MK443010	MK578139	MK442985
D. gardeniae	CBS 288.56	KC343113	KC344081	KC343597	KC343839	KC343355
D. garethjonesii	MFLUCC 12-0542a^{T}	KT459423	KT459441	-	KT459457	KT459470
D. glabrae	SCHM 3622^{T}	AY601918	-	-	-	-
D. gossiae	BRIP 59730a^{T}	OM918693	OM960620	-	OM960602	-
D. goulteri	BRIP 55657a^{T}	KJ197290	KJ197270	-	KJ197252	-
D. grandiflori	SAUCC194.84^{T}	MT822612	MT855809	MT85558	MT855924	MT855691
D. griceae	BRIP 67014a^{T}	OM918694	-	OM960621	OM960603	-
D. guangdongensis	ZHKUCC 20-0014^{T}	MT355684	MT409292	-	MT409338	MT409314
D. guangxiensis	JZB 320094^{T}	MK335772	MK500168	-	MK523566	MK736727
D. guizhouensis	GZAAS 20-0338^{T}	OM060254	OL961762	-	OL961761	OL961763
D. gulyae	BRIP 54025^{T}	JF431299	KJ197271	-	JN645803	-
D. guttulata	CGMCC 3.20100^{T}	MT385950	MT424705	MW022491	MT424685	MW022470
D. helianthi	CBS 592.81^{T}	KC343115	KC344083	KC343599	KC343841	JX197454
D. helicis	CBS 138596^{T}	KJ210538	KJ420828	KJ420875	KJ210559	KJ435043
D. heliconiae	SAUCC 194.77^{T}	MT822605	MT855802	MT855573	MT855917	MT855684

D. heterophyllae	CBS 143769^{T}	MG600222	MG600226	MG600220	MG600224	MG600218
D. heterostemmatis	SAUCC 194.85^{T}	MT822613	MT855810	MT855581	MT855925	MT855692
D. hickoriae	CBS 145.26^{T}	KC343118	KC344086	KC343602	KC343844	KC343360
D. hispaniae	CBS 143351^{T}	MG281123	MG281296	MG281471	MG281644	MG281820
D. hongkongensis	CBS 115448^{T}	KC343119	KC344087	KC343603	KC343845	KC343361
D. hordei	CBS 481.92	KC343120	KC344088	KC343604	KC343846	KC343362
D. howardiae	BRIP 59697a^{T}	OM918695	OM960622	-	OM960604	-
D. hsinchuensis	NTUPPMCC 18-153-1^{T}	MZ268409	MZ268430	MZ268493	MZ268472	MZ268451
D. huangshanensis	CNUCC 201903^{T}	MN219730	MN227011	MN224558	MN224678	-
D. hubeiensis	JZB 320123^{T}	MK335809	MK500148	-	MK523570	MK500235
D. humulicola	CT2018-1^{T}	MN152927	-	MN180213	MN180207	MN180204
D. hunanensis	HNZZ 023^{T}	MZ509550	MZ504713	MZ504691	MZ504702	MZ504680
D. hungariae	CBS 143353^{T}	MG281126	MG281299	MG281474	MG281647	MG281823
D. ilicicola	FPH 2015502^{T}	MH171064	MH171074	MH171084	-	-
D. impulsa	CBS 114434	KC343121	KC344089	KC343605	KC343847	KC343363
D. incompleta	CGMCC 3.18288^{T}	KX986794	KX999226	KX999265	KX999186	KX999289
D. inconspicua	CBS 133813^{T}	KC343123	KC344091	KC343607	KC343849	KC343365
D. infecunda	CBS 133812^{T}	KC343126	KC344094	KC343610	KC343852	KC343368
D. infertilis	CBS 230.52^{T}	KC343052	KC344020	KC343536	KC343778	KC343294
D. irregularis	CGMCC 3.20092^{T}	MT385951	MT424706	-	MT424686	MT424721
D. isoberliniae	CBS 137981^{T}	KJ869133	KJ869245	-	-	-
D. italiana	MFLUCC 18-0090^{T}	MH846237	MH853688	-	MH853686	MH853690
D. jinxiu	CGMCC3.20269^{T}	MW477881	MW480877	MW480865	MW480873	MW480869
D. juglandicola	CFCC 51134^{T}	MW477881	KX024634	-	KX024628	KX024616
D. kadsurae	CFCC 52586^{T}	MH121521	MH121600	MH121479	MH121563	MH121439
D. kochmanii	BRIP 54033^{T}	JF431295	-	-	JN645809	-

D. kongii	BRIP 54031^{T}	JF431301	KJ197272	-	JN645797	-
D. krabiensis	MFLUCC 17-2481^{T}	MN047101	MN431495	-	MN433215	
D. lenispora	CGMCC 3.20101^{T}	MT385952	MT424707	MW022493	MT424687	MW022472
D. leucospermi	CBS 111980^{T}	JN712460	KY435673	KY435653	KY435632	KY435663
D. limonicola	CBS 142549^{T}	MF418422	MF418582	MF418342	MF418501	MF418256
D. liquidambari	SCHM 3621^{T}	AY601919	-	-	-	-
D. litchicola	BRIP 54900^{T}	JX862533	KF170925	-	JX862539	-
D. litchii	SAUCC 194.22^{T}	MT822550	MT855747	MT855519	MT855863	MT855635
D. lithocarpi	CGMCC 3.15175^{T}	KC153104	KF576311	-	KC153095	-
D. litoricola	MFLUCC 16-1195^{T}	MF190139	-	-	-	-
D. longicicola	CGMCC 3.17089^{T}	KF576267	KF576291	-	KF576242	-
D. longicolla	FAU 599^{T}	KJ590728	KJ610883	KJ659188	KJ590767	KJ612124
D. longispora	CBS 194.36^{T}	KC343135	KC344103	KC343619	KC343861	KC343377
D. lonicerae	MFLUCC 17-0963^{T}	KY964190	KY964073	-	KY964146	KY964116
D. lovelaceae	BRIP 60163a^{T}	OM918696	OM960623	-	OM960605	-
D. lusitanicae	CBS 123212^{T}	KC343136	KC344104	KC343620	KC343862	KC343378
D. lutescens	SAUCC 194.36^{T}	MT822564	MT855761	MT855533	MT855877	MT855647
D. macadamiae	BRIP 66526^{T}	MN708230	MN696539	-	MN696528	-
D. machili	SAUCC 194.111^{T}	MT822639	MT855836	MT855606	MT855951	MT855718
D. macintoshii	BRIP 55064a^{T}	KJ197289	KJ197269	-	KJ197251	-
D. mahothocarpi	CGMCC 3.15181	KC153096	-	-	KC153087	-
D. malorum	CBS142383^{T}	KY435638	KY435668	KY435648	KY435627	KY435658
D. manihotia	CBS 505.76	KC343138	KC344106	KC343622	KC343864	KC343380
D. marina	MFLU 17-2622^{T}	MN047102	-	-	-	-
D. maritima	DAOMC 250563^{T}	KU552025	KU574615	-	KU552023	-
D. masirevicii	BRIP 57892a^{T}	KJ197277	KJ197257	-	KJ197239	-

D. mayteni	CBS 133185^{T}	KC343139	KC344107	KC343623	KC343865	KC343381
D. maytenicola	CBS 136441^{T}	KF777157	KF777250	-	-	-
D. mclennaniae	BRIP 60072a^{T}	OM918697	OM960624	-	OM960606	-
D. mediterranea	DAL-34	MT007489	MT006686	MT007095	MT006989	MT006761
D. megalospora	CBS 143.27	KC343140	KC344108	KC343624	KC343866	KC343382
D. melastomatis	SAUCC 194.55^{T}	MT822583	MT855780	MT855551	MT855896	MT855664
D. meliae	CFCC 53089^{T}	MK432657	MK578057	ON081662	ON081654	-
D. melitensis	CBS 142551^{T}	MF418424	MF418584	MF418344	MF418503	MF418258
D. melonis	CBS 507.78^{T}	KC343142	KC344110	KC343626	KC343868	KC343384
D. micheliae	SCHM 3603	AY620820	-	-	-	-
D. middletonii	BRIP 54884e^{T}	KJ197286	KJ197266	-	KJ197248	-
D. millettiae	GUCC 9167^{T}	MK398674	MK502089	-	MK480609	MK502086
D. minima	CGMCC 3.20097^{T}	MT385953	MT424708	MW022496	MT424688	MT424722
D. minusculata	CGMCC 3.20098^{T}	MT385957	MT424712	MW022499	MT424692	MW022475
D. miriciae	BRIP 54736j^{T}	KJ197283	KJ197263	-	KJ197245	-
D. momicola	MFLUCC 16-0113^{T}	KU557563	KU557587	-	KU557631	KU557611
D. monetii	MF-Ha18-049^{T}	MW008494	MW008505	MZ671965	MW008516	MZ671939
D. moriniae	BRIP 60190a^{T}	OM918698	OM960625	-	OM960607	-
D. multigutullata	ICMP 20656^{T}	KJ490633	KJ490454	KJ490575	KJ490512	-
D. musigena	CBS 129519^{T}	KC343143	KC344111	KC343627	KC343869	KC343385
D. myracrodruonis	URM 7972^{T}	MK205289	MK205291	-	MK213408	MK205290
D. nebulae	PMM 1681^{T}	KY511337	KY511369	-	MH708552	-
D. neilliae	CBS 144.27^{T}	KC343144	KC344112	KC343628	KC343870	KC343386
D. neoarctii	CBS 109490	KC343145	KC344113	KC343629	KC343871	KC343387
D. neoraonikayaporum	MFLUCC 14-1136^{T}	KU712449	KU743988	-	KU749369	KU749356
D. nigra	JZB 320170^{T}	MN653009	MN887113	-	MN892277	-

D. nobilis	CBS 587.79	KC343153	KC344121	KC343637	KC343879	KC343395
D. nomurai	CBS 157.29	KC343154	KC344122	KC343638	KC343880	KC343396
D. norfolkensis	BRIP 59718a[T]	OM918699	OM960626	-	OM960608	-
D. nothofagi	BRIP 54801[T]	JX862530	KF170922	-	JX862536	-
D. novem	CBS 127271[T]	KC343157	KC344125	KC343641	KC343883	KC343399
D. obtusifoliae	CBS 143449[T]	MG386072	-	MG386137	-	-
D. ocoteae	CBS 141330[T]	KX228293	KX228388	-	-	-
D. oculi	HHUF 30565[T]	LC373515	LC373519	-	LC373517	-
D. oncostoma	CBS 589.78	KC343162	KC344130	KC343646	KC343888	KC343404
D. oraccinii	LC 3166[T]	KP267863	KP293443	KP293517	KP267937	-
D. orixae	HKAS 121465[T]	OK283041	OK432278	OK484486	OK432279	OK484485
D. osmanthi	GUCC 9165[T]	MK398675	MK502090	-	MK480610	MK502087
D. ovalispora	ICMP 20659[T]	KJ490628	KJ490449	KJ490570	KJ490507	-
D. ovoicicola	CGMCC 3.17092[T]	KF576264	KF576288	-	KF576239	KF576222
D. oxe	CBS 133186[T]	KC343164	KC344132	KC343648	KC343890	KC343406
D. pachirae	COAD 2074[T]	MG559537	MG559541	-	MG559539	MG559535
D. padi var. *padi*	CBS 114200	KC343169	KC344137	KC343653	KC343895	KC343411
D. padina	CFCC 52590[T]	MH121525	MH121604	MH121483	MH121567	MH121443
D. pandanicola	MFLUCC 17-0607[T]	MG646974	MG646930	-	-	-
D. paranensis	CBS 133184	KC343171	KC344139	KC343655	KC343897	KC343413
D. parapterocarpi	CBS 137986[T]	KJ869138	KJ869248	-	-	-
D. parvae	PSCG 034[T]	MK626919	MK691248	MK726210	MK654858	-
D. pascoei	BRIP 54847[T]	JX862532	KF170924	-	JX862538	-
D. passiflorae	CBS 132527[T]	JX069860	KY435674	KY435654	KY435633	KY435664
D. passifloricola	CBS 141329[T]	KX228292	KX228387	KX228367	-	-
D. patagonica	CBS 145291[T]	MN509717	MN509728	-	MN509739	MN974279

D. penetriteum	LC 3353	KP714505	KP714529	KP714493	KP714517	-
D. perjuncta	CBS 109745^{T}	KC343172	KC344140	KC343656	KC343898	KC343414
D. perniciosa	CBS 124030	KC343149	KC344117	KC343633	KC343875	KC343391
D. perseae	CBS 151.73	KC343173	KC344141	KC343657	KC343899	KC343415
D. pescicola	MFLUCC 16-0105^{T}	KU557555	KU557579	-	KU557623	KU557603
D. phaseolorum	CBS 113425	KC343174	KC344142	KC343658	KC343900	KC343416
D. phragmitis	CBS 138897^{T}	KP004445	KP004507	KP004503	-	-
D. phillipsii	CAA 817^{T}	MK792305	MN000351	MK871445	MK828076	MK883831
D. phyllanthicola	SCHM 3680^{T}	AY620819	-	-	-	-
D. platzii	BRIP 60353a	OM918700	OM960627	-	OM960609	-
D. podocarpi-macrophylli	CGMCC 3.18281^{T}	KX986774	KX999207	KX999246	KX999167	KX999278
D. poincianellae	URM 7932^{T}	MH989509	MH989537	MH989539	MH989538	MH989540
D. pometiae	SAUCC 194.72^{T}	MT822600	MT855797	MT855568	MT855912	MT855679
D. portugallica	CBS 144228^{T}	MH063905	MH063917	MH063899	MH063911	MH063893
D. pseudoalnea	CFCC 54190^{T}	MZ727037	MZ753487	MZ781302	MZ816343	MZ753468
D. pseudoinconspicua	URM 7874^{T}	MH122538	MH122524	MH122517	MH122533	MH122528
D. pseudolongicolla	CBS 117165^{T}	DQ286285	-	-	DQ286259	-
D. pseudomangiferae	CBS 101339^{T}	KC343181	KC344149	KC343665	KC343907	KC343423
D. pseudooculi	HHUF 30617^{T}	LC373515	LC373519	-	LC373517	-
D. pseudophoenicicola	CBS 462.69^{T}	KC343184	KC344152	KC343668	KC343910	KC343426
D. pseudotsugae	MFLU 15-3228^{T}	KY964225	KY964108	-	KY964181	KY964138
D. psoraleae	CBS 136412^{T}	KF777158	KF777251	-	KF777245	-
D. psoraleae-pinnatae	CBS 136413^{T}	KF777159	KF777252	-	-	-
D. pterocarpi	MFLUCC 10-0571	JQ619899	JX275460	-	JX275416	JX197451
D. pterocarpicola	MFLUCC 10-0580a	JQ619887	JX275441	-	JX275403	JX197433
D. pulla	CBS 338.89^{T}	KC343152	KC344120	KC343636	KC343878	KC343394

D. pungensis	SAUCC 194.112^T	MT822640	MT855837	MT855607	MT855952	MT855719
D. pustulata	CBS 109742	KC343185	KC344153	KC343669	KC343911	KC343427
D. pyracanthae	CBS142384^T	KY435635	KY435666	KY435645	KY435625	KY435656
D. quercicola	CSUFTCC 104^T	ON076567	-	ON081667	ON081659	ON081670
D. racemosae	CBS 143770^T	MG600223	MG600227	MG600221	MG600225	MG600219
D. raonikayaporum	CBS 133182^T	KC343188	KC344156	KC343672	KC343914	KC343430
D. ravennica	MFLUCC 15–0479^T	KU900335	KX432254	-	KX365197	-
D. rhodomyrti	CFCC 53101^T	MK432643	MK578046	MK442990	MK578119	MK442965
D. rhoina	CBS 146.27	KC343189	KC344157	KC343673	KC343915	KC343431
D. rhusicola	CBS 129528^T	JF951146	KC843205	-	KC843100	KC843124
D. rosae	MFLUCC 17-2658^T	MG828894	MG843878	-	-	MG829273
D. rosicola	MFLU 17-0646^T	MG828895	MG843877	-	MG829270	MG829274
D. rosiphthora	COAD 2913^T	MT311196	-	-	MT313692	MT313690
D. rossmaniae	CAA 762^T	MK792290	MK837914	MK871432	MK828063	MK883822
D. rostrata	CFCC 50062^T	KP208847	KP208855	KP208851	KP208853	KP208849
D. rudis	CBS 113201	KC343234	KC344202	KC343718	KC343960	KC343476
D. rumicicola	MFLUCC 18-0739^T	MH84623	MK049555	-	MK049554	-
D. saccarata	CBS 116311^T	KC343190	KC344158	KC343674	KC343916	KC343432
D. sackstonii	BRIP 54669b^T	KJ197287	KJ197267	-	KJ197249	-
D. salicicola	BRIP 54825^T	JX862531	KF170923	-	JX862537	-
D. salinicola	MFLU 18-0553^T	MN047098	-	-	MN077073	-
D. sambucusii	CFCC 51986^T	KY852495	KY852511	KY852503	KY852507	KY852499
D. sapindicola	CFCC 55344^T	MW881507	MW898937	MW898940	MW898934	MW898943
D. schimae	CFCC 53103^T	MK432640	MK578043	MK442987	MK578116	MK442962
D. schini	CBS 133181^T	KC343191	KC344159	KC343675	KC343917	KC343433

D. schisandrae	CFCC 51988[T]	KY852497	KY852513	KY852505	KY852509	KY852501
D. schoeni	MFLU 15-1279[T]	KY964226	KY964109	-	KY964182	KY964139
D. sclerotioides	CBS 296.67[T]	KC343193	KC344161	KC343677	KC343919	KC343435
D. scobina	CBS 251.38	KC343195	KC344163	KC343679	KC343921	KC343437
D. searlei	BRIP 66528[T]	MN708231	MN696540	-	-	-
D. sennae	CFCC 51636[T]	KY203724	KY228891	-	KY228885	KY228875
D. sennicola	CFCC 51634[T]	KY203722	KY228889	-	KY228883	KY228873
D. serafiniae	BRIP 55665a[T]	KJ197274	KJ197254	-	KJ197236	-
D. shaanxiensis	CFCC 53106	MK432654	-	MK443001	MK578130	MK442976
D. shawiae	BRIP 64534a[T]	OM918701	OM960628	-	OM960610	-
D. shennongjiaensis	CNUCC201905[T]	MN216229	MN227012	MN224559	MN224672	MN224551
D. siamensis	MFLUCC 10-0573a	JQ619879	JX275429	-	JX275393	-
D. silvicola	CFCC 54191[T]	MZ727041	MZ753491	MZ753481	MZ816347	MZ753472
D. sinensis	CGMCC 3.19521[T]	MK637451	MK660447	-	MK660449	-
D. sojae	CBS 139282[T]	KJ590719	KJ610875	KJ659208	KJ590762	KJ612116
D. spartinicola	CBS 140003[T]	KR611879	KR857695	KR857696	-	-
D. spinosa	PSCG 383[T]	MK626849	MK691234	MK726156	MK654811	MK691129
D. sterilis	CBS 136969[T]	KJ160579	KJ160528	MF418350	KJ160611	KJ160548
D. stewartii	CBS 193.36	FJ889448	-	-	GQ250324	-
D. stictica	CBS 370.54	KC343212	KC344180	KC343696	KC343938	KC343454
D. subclavata	ICMP 20663[T]	KJ490630	KJ490451	KJ490572	KJ490509	-
D. subcylindrospora	KUMCC 17-0151[T]	MG746629	MG746631	-	MG746630	-
D. subellipicola	KUMCC 17-0153[T]	MG746632	MG746634	-	MG746633	-
D. subordinaria	CBS 101711	KC343213	KC344181	KC343697	KC343939	KC343455
D. taoicola	MFLUCC 16-0117[T]	KU557567	KU557591	-	KU557635	-
D. tarchonanthi	CBS 146073[T]	MT223794	MT223733	MT223759	-	-
D. tecomae	CBS 100547	KC343215	KC344183	KC343699	KC343941	KC343457

D. tectonae	MFLUCC 12-0777^{T}	KU712430	KU743977	-	KU749359	KU749345
D. tectonendophytica	MFLUCC 13-0471^{T}	KU712439	KU743986	-	KU749367	KU749354
D. tectonigena	MFLUCC 12-0767^{T}	KU712429	KU743976	-	KU749371	KU749358
D. terebinthifolii	CBS 133180^{T}	KC343216	KC344184	KC343700	KC343942	KC343458
D. ternstroemia	CGMCC 3.15183^{T}	KC153098	-	-	KC153089	-
D. trevorrowii	BRIP 70737a^{T}	OM918703	OM960630	-	OM960612	-
D. thunbergii	MFLUCC 10-0756a	JQ619893	JX275449	-	JX275409	JX197440
D. thunbergiicola	MFLUCC 12-0033^{T}	KP715097	-	-	KP715098	-
D. tibetensis	CFCC 51999^{T}	MF279843	MF279873	MF279828	MF279858	MF279888
D. torilicola	MFLUCC 17-1051^{T}	KY964212	KY964096	-	KY964168	KY964127
D. toxica	CBS 534.93^{T}	KC343220	KC344188	KC343704	KC343946	KC343462
D. toxicodendri	FFPRI 420987	LC275192	LC275224	LC275216	LC275216	LC275200
D. tulliensis	BRIP 62248a	KR936130	KR936132	-	KR936133	-
D. ueckeri	FAU 656	KJ590726	KJ610881	KJ659215	KJ590747	KJ612122
D. ukurunduensis	CFCC 52592^{T}	MH121527	-	MH121485	MH121569	MH121445
D. undulata	CGMCC 3.18293^{T}	KX986798	KX999230	KX999269	KX999190	-
D. unshiuensis	CGMCC3.17569^{T}	KJ490587	KJ490408	KJ490529	KJ490466	-
D. vaccinii	CBS 160.32^{T}	AF317578	KC344196	KC343712	GQ250326	KC343470
D. vacuae	CAA 830^{T}	MK792309	MK837931	MK871449	MK828080	MK883834
D. vangoghii	MF-Ha18-046^{T}	MW008492	MW008503	MZ671963	MW008514	MZ671937
D. vangueriae	CBS 137985^{T}	KJ869137	KJ869247	-	-	-
D. vawdreyi	BRIP 57887a	KR936126	KR936128	-	KR936129	-
D. velutina	CGMCC 3.18286^{T}	KX986790	KX999223	KX999261	KX999182	-
D. verniciicola	CFCC 53109^{T}	MK573944	MK574639	MK574599	MK574619	MK574583
D. vexans	CBS 127.14	KC343229	KC344197	KC343713	KC343955	KC343471
D. viniferae	JZB 320071^{T}	MK341551	MK500112	-	MK500107	MK500119
D. virgiliae	CBS 138788^{T}	KP247573	KP247582	-	-	-

D. vitimegaspora	STE-U 2675	AF230749	-	-	-	-
D. vochysiae	LGMF 1583[T]	MG976391	MK007527	MK033323	MK007526	MK007528
D. woodii	CBS 558.93	KC343244	KC344212	KC343728	KC343970	KC343486
D. woolworthii	CBS 148.27	KC343245	KC344213	KC343729	KC343971	KC343487
D. xishuangbanica	CGMCC 3.18282[T]	KX986783	KX999216	KX999255	KX999175	-
D. xunwuensis	CFCC 53085[T]	MK432663	MK578063	MK443008	MK578137	MK442983
D. yunnanensis	CGMCC 3.18289[T]	KX986796	KX999228	KX999267	KX999188	KX999290
D. zaobaisu	PSCG 031[T]	MK626922	MK691245	MK726207	MK654855	-
D. zaofenghuang	CGMCC3.20271[T]	MW477883	MW480875	-	MW480871	MW480867
Diaporthella corylina	CBS 121124	KC343004	KC343972	KC343488	KC343730	KC343246

[1]ATCC: American Type Culture Collection, Virginia, USA; BRIP: Queensland Plant Pathology Herbarium, Brisbane, Australia; CAA: Collection of Artur Alves housed at Department of Biology, University of Aveiro, Portugal; CBS: Westerdijk Fungal Biodiversity Institute, Utrecht, the Netherlands; CFCC: China Forestry Culture Collection Center, Beijing, China; CGMCC: Chinese General Microbiological Culture Collection Center, Beijing, China; CMRP: Taxonline Microbiological Collections of Paraná Network, at the Federal University of Paraná, Brazil; CNUCC: Capital Normal University Culture Collection Center, Beijing, China; COAD: Culture Collection of Octávio de Almeida Drumond. Universidade Federal de Viçosa, Viçosa, Brasil; CPC: Culture collection of Pedro Crous, housed at Westerdijk Fungal Biodiversity Institute; CSUFTCC: Central South University of Forestry and Technology Culture Collection, Hunan, China; DAOM: Plant Research Institute, Department of Agriculture (Mycology), Ottawa, Canada; DAL: strains deposited in fungal collection of the Instituto Agroforestal Mediterráneo–Universitat Politècnica de València, Valencia, Spain; DAOMC: Canadian Collection of Fungal Cultures, Ottawa, Canada; FPH: personal collection of Francesca Peduto Hand, Department of Plant Pathology, The Ohio State University, Columbus; GUCC: Culture Collection at the Department of Plant Pathology, Agriculture College, Guizhou University, China; GZAAS: Herbarium of Guizhou Academy of Agricultural Sciences, Guiyang, China; FAU: Isolates in culture collection of Systematic Mycology and Microbiology Laboratory; FFPRI: the Forestry and Forest Products Research Institute culture collection, Tsukuba, Japan; HKAS: Chinese Academy of Sciences, Kunming, China; HNZZ: Central South University of Forestry and Technology, Changsha, China; ICMP: International Collection of Micro-organisms from Plants, Landcare Research, Private Bag 92170, Auckland, New Zealand; IFRDCC: International Fungal Research and Development Culture Collection; KUMCC: Kumming Institute of Botany, Kumming, China; JZB: Culture collection of Institute of Plant and Environment Protection, Beijing, China; LC: Working collection of Lei Cai, housed at Institute of Microbiology, Chinese Academy of Sciences, Beijing, China; LGMF, Laboratório de Genética de Microrganismos (LabGeM) culture collection, at the Federal University of Paraná, Brazil; MAFF: Ministry of Agriculture, Forestry and Fisheries, Tokyo, Japan; MF: Laboratory of Mycology and Phytopathology, Institute of Plant Protection (VIZR), Saint Petersburg, Russia; MFLU: Mae Fah Luang University herbarium, Thailand; MFLUCC: Mae Fah Luang University Culture Collection, Chiang Rai, Thailand; NCYU: Department of Plant Medicine, National Chiayi University, Chiayi, Taiwan;

NTUPPMCC: Department of Plant Pathology and Microbiology, National Taiwan University Culture Collection, PMM: collection of Providence Moyo at the University of Stellenbosch, Stellenbosch, South Africa; PSCG: Personal Culture Collection Y.S. Guo, China; SAUCC: Shandong Agricultural University Culture Collection, Shandong, China; SCHM: Mycological Herbarium of South China Agricultural University, Guangzhou, China; STMA: fungarium of the Helmholtz Centre for Infection Research, Braunschweig, Germany; URM: Culture Collection at the Universidade Federal de Pernambuco, Recife, Brazil; VTCC: Vietnam Type Culture Collection, Center of Biotechnology, Vietnam National University, Hanoi, Vietnam; ZHKUCC: Culture Collection of Zhongkai University of Agriculture and Engineering, Guangzhou, China. ^{T} indicates ex-type material.

[2]ITS: internal transcribed spacers and intervening 5.8S nrDNA; *tub2*: partial β-tubulin gene; *his3*: partial histone H3 gene; *tef1*: partial elongation factor 1-alpha gene; *cal*: partial calmodulin gene

Table S6: Selected edge-linked proportional partition substitution models subjected to IQTree2 calculated with ModelTest as implemented in IQTree using Bayesian information criterion (BIC).

DNA Locus	Model	Speed	Parameters
**ITS**	TNe+I+G4	0.9587	TNe{2.82221,7.01859}+FQ+I{0.636222}+G4{0.422006}
cal	TNe+R2	0.9204	TNe{2.57916,4.98792}+FQ+R2{0.82344,0.325372,0.17656,4.14633}
his3	TN+F+I	0.3385	TN{1.22781,9.29183}+F{0.1749,0.376724,0.237885,0.210491}+I{0.867199}
tef1	TIM2+F+G4	1.6094	TIM2{1.84916,4.52005,7.72974}+F{0.230302,0.304076,0.204932,0.26069}+G4{0.454737}
tub2	K3Pu+F+R3	1.0764	K3Pu{3.90115,0.626085}+F{0.208225,0.35591,0.225394,0.21047}+R3{0.792328,0.235654,0.172231,2.05872,0.0354402,12.9432}

Table S7: Characteristics of the restricted MAFFT alignments following the first phylogenetic analysis using IQTree 2.1.3 for phylogenetic inference.

DNA Locus	Sequences	Sites	Unique	Informative	Invariant	Constant
**ITS**	66	554	189	69	428	428
cal	38	423	162	83	252	252
his3	29	359	45	24	321	321
tef1	57	392	248	119	191	191
tub2	59	721	340	123	427	426

Table S8: Selected unlinked partition substitution model subjected to MrBayes calculated with ModelFinder as implemented in the Phylosuite program package using Bayesian information criterion (BIC).

DNA Locus	Model	BIC	lnL
**ITS + *cal* + *his2* + *tef1* + *tub2***	GTR+I+G	30886.3962229	-14900.859375

- Alignment of the ITS sequences used in the phylogenetic study

>Diaporthe_amygdali_CBS_126679

AACAAGGTCTCCGTTGGTGAACCAGCGGAGGGATCATTGCTG-GAACGCGCCTC-
GGCGCACCCAGAAACCC-TTTGTGAACTTATACC-
TTACTGTTGCCTCGGCGCAGGCCGGCTCCCATCTGGGGGCCCCTCGTTTC-----

TGACGAGGAGCAGGCTCGCCGGCGGCCAAGTTAACTCTTGTTTTTAATTTGAAACTCTG
AG-
AATAAAACATAAATGAATCAAAACTTTCAACAACGGATCTCTTGGTTCTGGCATCGATG
AAGAACGCAGCGAAATGCGATAAGTAATGTGAATTGCAGAATTCAGTGAATCATCGAA
TCTTTGAACGCACATTGCGCCCTCTGGTATTCCGGAGGGCATGCCTGTTCGAGCGTCATT
TCAACCCTCAAGCCTGGCTTGGTGATGGGGCACTGCCTTTGTGTAAAAGCGAAGGCAGG
CCCTGAAATTCAGTGGCGAGCTCGCCAGGACTCCGAGCGCAGTAGTTAAACCCTCGCTT
TGGAAGG-ACTGG--CGGTGCCCTGCCGTTAAACCCCCAACTCTTGAAAATT

>Diaporthe_eres_CBS_138594

NNNNNNNNNNNNNNNNNNNNNNNNGCGGAGGGATCATTGCTG-
GAACGCGCCCCAGGCGCACCCAGAAACCC-TTTGTGAACTTATACC-
TTACTGTTGCCTCGGCGCTAGCTGGTCCC--TC--GGGGCCCCTCACCCT----
CGGGTGTTGAGACAGCCCGTCGGCGGCCAACCTAACTCTTGTTTTTACACTGAAACTCT
GAG-
CACAAAACATAAATGAATCAAAACTTTCAACAACGGATCTCTTGGTTCTGGCATCGATG
AAGAACGCAGCGAAATGCGATAAGTAATGTGAATTGCAGAATTCAGTGAATCATCGAA
TCTTTGAACGCACATTGCGCCCTCTGGTATTCCGGAGGGCATGCCTGTTCGAGCGTCATT
TCAACCCTCAAGCCTGGCTTGGTGATGGGGCACTGCTTCT----
TACCCAAGAAGCAGGCCCTGAAATTCAGTGGCGAGCTCGCCAGGACCCCGAGCGCAGT
AGTTAAACCCTCGCTCTGGAAGGCCCTGG--
CGGTGCCCTGCCGTTAAACCCCCAACTTCTGAAAATT

>Diaporthe_acuta_PSCG_047

NNNNNNNNNNNNNNNNNNNNNNNNNNNNNNNNNNNNNNNNNNNNNNNNNNNNNNNN
NNNNNNNNNNNNNNNNNNNCCC-TATGTGAAC-TATACC-T--CTGTTGCCTCGGCGC-
TGCTGGTCTT--TA--
TAGGCCCTTTACTGCTTTAGTGGTAAAGAGAAGGCACGCCGGCGGCCAAAACAACTC-
TGTTTTTATACTGAAACTCTGAG---CAATACA-
AAATGAATCAAAACTTTCAACAACGGATCTCTTGGTTCTGGCATCGATGAAGAACGCA
GCGAAATGCGATAAGTAATGTGAATTGCAGAATTCAGTGAATCATCGAATCTTTGAACG
CACATTGCGCCCTCTGGTATTCCGGAGGGCATGCCTGTTCGAGCGTCATTTCAACCCTCA
AGCATTGCTTGGTGTTGGGGCACTGCTTTT--------
AACGAAGCAGGCCCTGAAATCTAGTGGCGAGCGCGCCAGGACCCCGAGCGTAGTAGTT
AAACCCTCGCTTTGGAAGGCCCTGG--
CGGTGCCCTGCCGTTAAACCCCCAACTTTTGAAAATT

>Diaporthe_anhuiensis_CNUCC_201902

NNNNNNNNNNNNNNNNNNNNNNNNNNNNNNGTGGCGTCGGCTT-
GGCCGCGCCCAGGGCGCACCCAGAAACCCTTTTGTGAACTTATACC-TT-

TTGTTGCCTCGGCGCATGCTGGTCTC--TAG-TAGGCCCCTCACCCC------
GGTGAGGAGAAGGCACGCCGGCGGCCAAGTTAACTCTTGTTTTTACACTGAAACTCTGA
GAAAAAAAACACAAATGAATCAAAACTTTCAACAACGGATCTCTTGGTTCTGGCATCG
ATGAAGAACGCAGCGAAATGCGATAAGTAATGTGAATTGCAGAATTCAGTGAATCATC
GAATCTTTGAACGCACATTGCGCCCTCTGGTATTCCGGAGGGCATGCCTGTTCGAGCGT
CATTTCAACCCTCAAGCATTGCTTGGTGTTGGGGCACTGCTTTT--------
TACCAAGCAGGCCCTGAAATCTAGTGGCGAGCTCGCCAGGACCCCGAGCGCAGTAGTT
AAACCCTCGCTCTGGAAGGCCCTGG--
CGGTGCCCTGCCGTTAAACCCCCAACTTCTGAAAATT

>Diaporthe_annellsiae_BRIP_59731a

AACAAGGTCTCCGTTGGTGAACCAGCGGAGGGATCATTGCTG-
GAACGCGCCCCAGGCGCACCCAGAAACCC-TTTGTGAACTTATACC-
TTACTGTTGCCTCGGCGAATGCTGGCCCC--CTC-GGGGTCCCCTG--GA------
GACAGGGAGCAGGCACGCCGGCGGCCAAGTTAACTCTTGTTTTTACACTGAAACTCTGA
G--
AAAAAACACAAATGAATCAAAACTTTCAACAACGGATCTCTTGGTTCTGGCATCGATG
AAGAACGCAGCGAAATGCGATAAGTAATGTGAATTGCAGAATTCAGTGAATCATCGAA
TCTTTGAACGCACATTGCGCCCTCTGGTATTCCGGAGGGCATGCCTGTTCGAGCGTCATT
TCAACCCTCAAGCATTGCTTGGTGTTGGGGCACTGCCTGT--------
AAAAGGGCAGGCCCTGAAATCTAGTGGCGAGCTCGCCAGGACCCCGAGCGTAGTAGTT
AAACCCTCGCTTTGGAAGGCCCTGG--
CGGTGCCCTGCCGTTAAACCCCCNNNNNNNNNNNNNN

>Diaporthe_arecae_CBS_161_64

AACAAGGTCTCCGTTGGTGAACCAGCGGAGGGATCATTGCTG-
GAACGCGCCCTAGGCGCACCCAGAAACCC-TTTGTGAACTTATACC-
TTACTGTTGCCTCGGCGCATGCTGGCCCC--CTC-GGGGCCCCCTG--GA------
GACAGGGAGCAGGCACGCCGGCGGCCAAGTTAACTCTTGTTTTTACACTGAAACTCTGA
G-
AAAAAAACACAAATGAATCAAAACTTTCAACAACGGATCTCTTGGTTCTGGCATCGAT
GAAGAACGCAGCGAAATGCGATAAGTAATGTGAATTGCAGAATTCAGTGAATCATCGA
ATCTTTGAACGCACATTGCGCCCTCTGGTATTCCGGAGGGCATGCCTGTTCGAGCGTCAT
TTCAACCCTCAAGCATTGCTTGGTGTTGGGGCACTGCTTTT--------
TACCGAGCAGGCCCTGAAATCTAGTGGCGAGCTCGCCAGGACCCCGAGCGCAGTAGTT
AAACCCTCGCTCTGGAAGGCCCTGG--
CGGTGCCCTGCCGTTAAACCCCCAACTTCTGAAAATT

>Diaporthe_arengae_CBS_114979

AACAAGGTCTCCGTTGGTGAACCAGCGGAGGGATCATTGCTG-
GAACGCGCCCCAGGCGCACCCAGAAACCC-TTTGTGAACTTATACC-TT-
TTGTTGCCTCGGCGCATGCTGGCCTC--TAG-TAGGCCCCTCACCCC------
GGTGAGGAGAAGGCACGCCGGCGGCCAAGTTAACTCTTGTTTTTACACTGAAACTCTGA
G--
AAAAAACACAAATGAATCAAAACTTTCAACAACGGATCTCTTGGTTCTGGCATCGATG
AAGAACGCAGCGAAATGCGATAAGTAATGTGAATTGCAGAATTCAGTGAATCATCGAA
TCTTTGAACGCACATTGCGCCCTCTGGTATTCCGGAGGGCATGCCTGTTCGAGCGTCATT
TCAACCCTCAAGCATTGCTTGGTGTTGGGGCACTGCTTCT--------
AACGAAGCAGGCCCTGAAATCTAGTGGCGAGCTCGCCAGGACCCCGAGCGCAGTAGTT
AAACCCTCGCTCTGGAAGGCCCTGG--
CGGTGCCCTGCCGTTAAACCCCCAACTTCTGAAAATT

>Diaporthe_aseana_MFLUCC_12_0299a

NNNNNNNNNNNNNNNNNNNNNNNNNCGGAGGGATCATTGCTG-
GAACGCGCCCCAGGCGCACCCAGAAACCC-TTTGTGAACTTATACC-
TTACTGTTGCCTCGGCGCATGCTGGCCCC--CCTGGGGGTCCCTCG--GA------
GACGAGGAGCAGGCACGCCGGCGGCCAAGTTAACTCTTGTTTTTACACTGAAACTCTGA
G-
AAAAAAACACAAATGAATCAAAACTTTCAACAACGGATCTCTTGGTTCTGGCATCGAT
GAAGAACGCAGCGAAATGCGATAAGTAATGTGAATTGCAGAATTCAGTGAATCATCGA
ATCTTTGAACGCACATTGCGCCCTCTGGTATTCCGGAGGGCATGCCTGTTCGAGCGTCAT
TTCAACCCTCAAGCATTGCTTGGTGTTGGGGCACTGCCTTT-------
TAAACGGGCAGGCCCTGAAATCTAGTGGCGAGCTCGCCAGGACCCCGAGCGCAGTAGT
TAAACCCTCGCTCTGGAAGGCCCTGG--
CGGTGCCCTGCCGTTAAACCCCCAACTCTTGAAAATT

>Diaporthe_australiana_BRIP_66145

AACAAGGTCTCCGTTGGTGAACCAGCGGAGGGATCATTGCTG-
GAACGCGCCCCAGGCGCACCCAGAAACCC-TTTGTGAACTTATACC-
TTACTGTTGCCTCGGCGCATGCTGGCCCC--CTG-GGGGTCCCTCACTCC------
GATGAGGAGCAGGCACGCCGGCGGCCAAGTTAACTCTTGTTTTTACACTGAAACTCTGA
GAAAAAAAACACAAATGAATCAAAACTTTCAACAACGGATCTCTTGGTTCTGGCATCG
ATGAAGAACGCAGCGAAATGCGATAAGTAATGTGAATTGCAGAATTCAGTGAATCATC
GAATCTTTGAACGCACATTGCGCCCTCTGGTATTCCGGAGGGCATGCCTGTTCGAGCGT
CATTTCAACCCTCAAGCACTGCTTGGTGTTGGGGCACTGCCTTT-----
TACCCAAGGGCAGGCCCTGAAATCTAGTGGCGAGCTCGCCAGGACCCCGAGCGCAGTA
GTTAAACCCTCGCTCTGGAAGGCCCTGG--
CGGTGCCCTGCCGTTAAACCCCCAACTCTTGAAAATT

>Phomopsis_averrhoae_SCHM_3605

NNNNNNNNNNNNNNNNNNNNNNNCCAGCGGAGGGATCATTGCTG-
GAACGCGCCCTAGGCGCACCCAGAAACCC-TTTGTGAACTTATACC-
TTACTGTTGCCTCGGCGCATGCTGGTCTC--TAG-TAGGCCCCTCACTCC------
GGTGAGGAGAAGGCACGCCGGCGGCCAAGTTAACTCTTGTTTTTACACTGAAACTCTGA
G--
AAAAAACACAAATGAATCAAAACTTTCAACAACGGATCTCTTGGTTCTGGCATCGATG
AAGAACGCAGCGAAATGCGATAAGTAATGTGAATTGCAGAATTCAGTGAATCATCGAA
TCTTTGAACGCACATTGCGCCCTCTGGTATTCCGGAGGGCATGCCTGTTCGAGCGTCATT
TCAACCCTCAAGCATTGCTTGGTGTTGGGGCACTGCTTTT--------
AACGAAGCAGGCCCTGAAATCTAGTGGCGAGCTCGCCAGGACCCCGAGCGTAGTAGTT
AAACCCTCGCTTTGGAAGGCCCTGG--
CGGTGCCCTGCCGTTAAACCCCCAACTTTTGAAAATT

>Diaporthe_bounty_BRIP_59361a

AACAAGGTCTCCGTTGGTGAACCAGCGGAGGGATCATTGCTG-
GAACGCGCCCCAGGCGCACCCAGAAACCC-TTTGTGAACTTATACC-
TTACTGTTGCCTCGGCGCATGCTGGTCTC--TAG-TAGGCCCCTCACCCC------
GGTGAGGAGAAGGCACGCCGGCGGCCAAGTTAACTCTTGTTTTTACACTGAAACTCTGA
G--
AAAAAACACAAATGAATCAAAACTTTCAACAACGGATCTCTTGGTTCTGGCATCGATG
AAGAACGCAGCGAAATGCGATAAGTAATGTGAATTGCAGAATTCAGTGAATCATCGAA
TCTTTGAACGCACATTGCGCCCTCTGGTATTCCGGAGGGCATGCCTGTTCGAGCGTCATT
TCAACCCTCAAGCACTGCTTGGTGTTGGGGCACTGCTTCT--------
CACGAAGCAGGCCCTGAAATCTAGTGGCGAGCTCGCCAGGACCCCGAGCGCAGTAGTT
AAACCCTCGCTCTGGAAGGCCCTGG--
CGGTGCCCTGCCGTTAAACCCCCAACTTCNNNNNNNN

>Diaporthe_camelliaeoleiferae_HNZZ027

NNNNNNNNNNNNNNNNNNNNNNNNNNNNNNNNNNNNNNNNNNNNNNNNNNNNNNNNN
NNNNNNNNNNNNNNNNNNNCCC-TTTGTGAACTTATACC-TT-
TTGTTGCCTCGGCGCATGCTGGTCTC--TAG-TAGGCCCCTCACCCC------
GGTGAGGAGACGGCACGCCGGCGGCCAAGTTAACTCTTGTTTTTACACTGAAACTCTGA
G--
AAAAAACACAAATGAATCAAAACTTTCAACAACGGATCTCTTGGTTCTGGCATCGATG
AAGAACGCAGCGAAATGCGATAAGTAATGTGAATTGCAGAATTCAGTGAATCATCGAA
TCTTTGAACGCACATTGCGCCCTCTGGTATTCCGGAGGGCATGCCTGTTCGAGCGTCATT
TCAACCCTCAAGCATTGCTTGGTGTTGGGGCACTGCTTTT--------
TACCAAGCAGGCCCTGAAATCTAGTGGCGAGCTCGCCAGGACCCCGAGCGCAGTAGTT
AAACCCTCGCTCTGGAAGGCCCTGG--
CGGTGCCCTGCCGTTAAACCCCCAACTTCTGAAANNN

>Diaporthe_ceratozamiae_CBS_131306

AACAAGGTCTCCGTTGGTGAACCAGCGGAGGGATCATTGCTG-
GAACGCGCCCCAGGCGCACCCAGAAACCC-TTTGTGAACTTATACC-TT-
TTGTTGCCTCGGCGCATGCTGGCCCT--TTT-GGGGTCCCCTG--GA------
GACAGGGAGCAGGCACGCCGGCGGCCAAGTTAACTCTTGTTTTTACATTGAAACTCTGA
G--
AAAAAACACAAATGAATCAAAACTTTCAACAACGGATCTCTTGGTTCTGGCATCGATG
AAGAACGCAGCGAAATGCGATAAGTAATGTGAATTGCAGAATTCAGTGAATCATCGAA
TCTTTGAACGCACATTGCGCCCTCTGGTATTCCGGAGGGCATGCCTGTTCGAGCGTCATT
TCAACCCTCAAGCACTGCTTGGTGTTGGGGCACTGCTTTT--------
AACGAAGCAGGCCCTGAAATCTAGTGGCGAGCTCGCCAGGACCCCGAGCGTAGTAGTT
AAACCCTCGCTTTGGAAGGCCCTGG--
CGGTGCCCTGCCGTTAAACCCCCAACTTCTGAAAATT

>Diaporthe_cercidis_CFCC_52565

NNNNNNNNNNNNNNNNNNNNNNNNNNNNNNNNNNNNNNNNNNNNNNNNNNNNNNNN
NNNNNNNNNNNNNNNNNNNNNCCC-TTTGTGAACTTATACC-
TTACTGTTGCCTCGGCGCATGCTGGCCCC--CTC-GGGGTCCCCTG--GA------
GACAGGGAGCAGGCACGCCGGCGGCCAAGTTAACTCTTGTTTTTACACTGAAACTCTGA
G-
AAAAAAACACAAATGAATCAAAACTTTCAACAACGGATCTCTTGGTTCTGGCATCGAT
GAAGAACGCAGCGAAATGCGATAAGTAATGTGAATTGCAGAATTCAGTGAATCATCGA
ATCTTTGAACGCACATTGCGCCCTCTGGTATTCCGGAGGGCATGCCTGTTCGAGCGTCAT
TTCAACCCTCAAGCATTGCTTGGTGTTGGGGCACTGCCTGT--------
AAAAGGGCAGGCCCTGAAATCTAGTGGCGAGCTCGCTAGGACCCCGAGCGTAGTAGTT
AAACCCTCGCTTTGGAAGGCCCTGG--
CGGTGCCCTGCCGTTAAACCCCCAACTTTTGAAANNN

>Diaporthe_cf_heveae_2_CBS_681_84

AACAAGGTCTCCGTTGGTGAACCAGCGGAGGGATCATTGCTG-
GAACGCGCCCCAGGCGCACCCAGAAACCC-TTTGTGAACTTATACC-
TTACTGTTGCCTCGGCGCATGCTGGCCCC--CCT-GGGGTCCCTTG--GA------
GACGAGGAGCAGGCACGCCGGCGGCCAAGTTAACTCTTGTTTTTACACTGAAACTCTGA
G-
AAAAAAACACAAATGAATCAAAACTTTCAACAACGGATCTCTTGGTTCTGGCATCGAT
GAAGAACGCAGCGAAATGCGATAAGTAATGTGAATTGCAGAATTCAGTGAATCATCGA
ATCTTTGAACGCACATTGCGCCCTCTGGTATTCCGGAGGGCATGCCTGTTCGAGCGTCAT
TTCAACCCTCAAGCATTGCTTGGTGTTGGGGCACTGCTTTT------
ACCCAAAAGCAGGCCCTGAAATCTAGTGGCGAGCTCGCCAGGACCCCGAGCGTAGTAG

TTAAACCCTCGCTCTGGAAGGCCCTGG--
CGGTGCCCTGCCGTTAAACCCCCAACTCTTGAAAATT

>Diaporthe_chiangmaiensis_NI207

NNNNNNNNNTCCGTTGGTGAACCAGCGGAGGGATCATTGCTG-
GAACGCGCCCCAGGCGCACCCAGAAACCC-
TTTGTGAACTTATACCTTTACTGTTGCCTCGGCGCATGCTGGCCCC--CTC-
GGGGTCCCTCG--GA------
GACGAGGAGCAGGCACGCCGGCGGCCAAGTTAACTCTTGTTTTTACACTGAAACTCTGA
G-
AAAAAAACMCAAATGAATCAAAACTTTCAACAACGGATCTCTTGGTTCTGGCATCGAT
GAAGAACGCAGCGAAATGCGATAAGTAATGTGAATTGCAGAATTCAGTGAATCATCGA
ATCTTTGAACGCACATTGCGCCCTCTGGTATTCCGGAGGGCATGCCTGTTCGAGCGTCAT
TTCAACCCTCAAGCATTGCTTGGTGTTGGGGCACTGCTTTT------
ACCCAAAAGCAGGCCCTGAAATCTAGTGGCGAGCTCGCCAGGACCCCGAGCGTAGTAG
TTAAACCCTCGCTTTGGAAGGCCCTGG--
CGGTGCCCTGCCGTTAAACCCCCNNNNNNNNNNNNNN

>Diaporthe_chrysalidocarpi_SAUCC194_35

NNNNNNNTCTCCGTTGGTGAACCAGCGGAGGGATCATTGCTG-
GAACGCGCCCTAGGCGCACCCAGAAACCC-TTTGTGAACTTATACC-
TTACTGTTGCCTCGGCGCATGCTGGCCCC--CTC-GGGGCCCCCTG--GA------
GACAGGGAGCAGGCACGCCGGCGGCCAAGTTAACTCTTGTTTTTACACTGAAACTCTGA
G--
AAAAAACACAAATGAATCAAAACTTTCAACAACGGATCTCTTGGTTCTGGCATCGATG
AAGAACGCAGCGAAATGCGATAAGTAATGTGAATTGCAGAATTCAGTGAATCATCGAA
TCTTTGAACGCACATTGCGCCCTCTGGTATTCCGGAGGGCATGCCTGTTCGAGCGTCATT
TCAACCCTCAAGCATTGCTTGGTGTTGGGGCACTGCCTGT--------
AAAAGGGCAGGCCCTGAAATCTAGTGGCGAGCTCGCCAGGACCCCGAGCGTAGTAGTT
AAACCCTCGCTTTGGAAGGCCCTGG--
CGGTGCCCTGCCGTTAAACCCCCAACTTCTGAAAATT

>Diaporthe_delonicis_MFLU_16_1059

NNNNNNNNNNNNNNNNNNNNNNNNNNCCGGAGGATCATTGCTGAGAACGCGCCCTA
GGCGCACCCAGAAACCC-TTTGTGAACTTATACC-
TTACTGTTGCCTCGGCGCATGCTGGCCCC--CTT-GGGGTCCCCTG--GA------
TACAGGGAGCAGGCACGCCGGCGGCCAAGTTAACTCTTGTTTTTTCACTGAAACTCTGA
A--
AAAAAACACAAATGAATCAAAACTTTCAACAACGGATCTCTTGGTTCTGGCATCGATTA
AAAACGCAACGAAATGCGATAAGTAATGTGAATTGCATAATTCAGTGAATCATCGAAT

CTTTGAACGCACATTGCGCCCTCTGGTATTCCGGAGGGCATGCCTGTTCGAGCGTCATTT
CAACCCTCAAGCATTGCTTGGTGTTGGGGCACTGCTTTT--------
AACCAAGCAGGCCCTGAAATCTAGTGGCGAGCTCGCCAGGACCCCGAGCGCAGTAGTT
AAACCCTCGCTTTGGAAGGCCCTGG--
CGGTGCCCTGCCGTTAAACCCCCAACTTCTGAAAATT

>Diaporthe_drenthii_BRIP_66524

AACAAGGTCTCCGTTGGTGAACCAGCGGAGGGATCATTGCTG-
GAACGCGCCCTAGGCGCACCCAGAAACCC-TTTGTGAACTTATACC-
TTACTGTTGCCTCGGCGCATGCTGGCCCC--CTC-GGGGTCCCCTG--GA------
GACAGGGAGCAGGCACGCCGGCGGCCAAGTTAACTCTTGTTTTTACACTGAAACTCTGA
G-
AAAAAAACACAAATGAATCAAAACTTTCAACAACGGATCTCTTGGTTCTGGCATCGAT
GAAGAACGCAGCGAAATGCGATAAGTAATGTGAATTGCAGAATTCAGTGAATCATCGA
ATCTTTGAACGCACATTGCGCCCTCTGGTATTCCGGAGGGCATGCCTGTTCGAGCGTCAT
TTCAACCCTCAAGCATTGCTTGGTGTTGGGGCACTGCCTGT--------
AAAAGGGCAGGCCCTGAAATCTAGTGGCGAGCTCGCCAGGACCCCGAGCGTAGTAGTT
AAACCCTCGCTTTGGAAGGCCCTGG--
CGGTGCCCTGCCGTTAAACCCCCAACTTTTGAAAATT

>Diaporthe_endocitricola_ZHKUCC20_0012

NNNNNNNNNTCCGTAGGTGAACCTGCGGAGGGATCATTGCTG-
GAACGCGCCCCAGGCGCACCCAGAAACCC-TTTGTGAACTTATACC-TT-
TTGTTGCCTCGGCGCATGCTGGTCTC--TAG-TAGGCCCCTCACCCC------
GGTGAGGAGACGGCACGCCGGCGGCCAAGTTAACTCTTGTTTTTACACTGAAACTCTGA
G--
AAAAAACACAAATGAATCAAAACTTTCAACAACGGATCTCTTGGTTCTGGCATCGATG
AAGAACGCAGCGAAATGCGATAAGTAATGTGAATTGCAGAATTCAGTGAATCATCGAA
TCTTTGAACGCACATTGCGCCCTCTGGTATTCCGGAGGGCATGCCTGTTCGAGCGTCATT
TCAACCCTCAAGCATTGCTTGGTGTTGGGGCACTGCTTTT--------
AACGAAGCAGGCCCTGAAATCTAGTGGCGAGCTCGCCAGGACCCCGAGCGCAGTAGTT
AAACCCTCGCTTTGGAAGGCCCTGG--
CGGTGCCCTGCCGTTAAACCCCCAACTTCTGAAAATT

>Diaporthe_eucalyptorum_CBS_132525

AACAAGGTCTCCGTTGGTGAACCAGCGGAGGGATCATTGCTG-
GAACGCGCCCCAGGCGCACCCAGAAACCC-TTTGTGAACTTATACC-
TTACTGTTGCCTCGGCGCATGCTGGCCCC--CTC-GGGGTCCCTTG--GA------
GACAAGGAGCAGGCACGCCGGCGGCCAAGTTAACTCTTGTTTTTACACTGAAACTCTGA
G-

AAAAAAACACAAATGAATCAAAACTTTCAACAACGGATCTCTTGGTTCTGGCATCGAT
GAAGAACGCAGCGAAATGCGATAAGTAATGTGAATTGCAGAATTCAGTGAATCATCGA
ATCTTTGAACGCACATTGCGCCCTCTGGTATTCCGGAGGGCATGCCTGTTCGAGCGTCAT
TTCAACCCTCAAGCACTGCTTGGTGTTGGGGCACTGCCTGT--------
AAAAGGGCAGGCCCTGAAATCTAGTGGCGAGCTCGCCAGGACCCCGAGCGCAGTAGTT
AAACCCTCGCTCTGGAAGGCCCTGG--
CGGTGCCCTGCCGTTAAACCCCCAACTTTTGAAAATT

>Diaporthe_eugeniae_CBS_444_82

AACAAGGTCTCCGTTGGTGAACCAGCGGAGGGATCATTGCTG-
GAACGCGCCCCAGGCGCACCCAGAAACCC-TTTGTGAACTTATACC-TT-
TTGTTGCCTCGGCGCATGCTGGTCTC--TAG-TAGGCCCCTCACCCC------
GGTGAGGAGACGGCACGCCGGCGGCCAAGTTAACTCTTGTTTTTACACTGAAACTCTGA
G--
AAAAAACACAAATGAATCAAAACTTTCAACAACGGATCTCTTGGTTCTGGCATCGATG
AAGAACGCAGCGAAATGCGATAAGTAATGTGAATTGCAGAATTCAGTGAATCATCGAA
TCTTTGAACGCACATTGCGCCCTCTGGTATTCCGGAGGGCATGCCTGTTCGAGCGTCATT
TCAACCCTCAAGCATTGCTTGGTGTTGGGGCACTGCTTCT--------
AACGAAGCAGGCCCTGAAATCTAGTGGCGAGCTCGCCAGGACCCCGAGCGTAGTAGTT
AAACCCTCGCTCTGGAAGGCCCTGG--
CGGTGCCCTGCCGTTAAACCCCCAACTTTTGAAAATT

>Diaporthe_fujianensis_JZB320149

AACAAGGTCTCCGTTGGTGAACCAGCGGAGGGATCATTGCTG-
GAACGCGCCCCAGGCGCACCCAGAAACCC-TTTGTGAACTTATACC-
TTACTGTTGCCTCGGCGCATGCCGGCCCC--CCCGGGGGCCCCTCG--GA------
GACGAGGAGCAGGCACGCCGGCGGCCAAGTTAACTCTTGTTTTTACACTGAAACTCTGA
G-
AAAAAAACACAAATGAATCAAAACTTTCAACAACGGATCTCTTGGTTCTGGCATCGAT
GAAGAACGCAGCGAAATGCGATAAGTAATGTGAATTGCAGAATTCAGTGAATCATCGA
ATCTTTGAACGCACATTGCGCCCTCTGGTATTCCGGAGGGCATGCCTGTTCGAGCGTCAT
TTCAACCCTCAAGCATTGCTTGGTGTTGGGGCACTGCCTGT--------
AAAAGGGCAGGCCCTGAAATCTAGTGGCGAGCTCGCCAGGACCCCGAGCGCAGTAGTT
AAACCCTCGCTTTGGAAGGCCCTGG--CGGTGCCCTGCCGTTAA-
CCCCCAANNNNNNNNNNNNN

>Diaporthe_fulvicolor_PSCG_051

NNNNNNNNNNNNNNNNNNNNNNNNNNNNNNNNNNNNNNNNNNNNNNNNNNNNNNNN
NNNNNNNNNNNNNNNNNNNNCCC-TTTGTGAACTTATACC-
TTACTGTTGCCTCGGCGCATGCTGGCCCC--CTC-GGGGCCCCTG--GA------

GACAGGGAGCAGGCACGCCGGCGGCCAAGTTAACTCTTGTTTTTACACTGAAACTCTGA
A--
AAAAAACACAAATGAATCAAAACTTTCAACAACGGATCTCTTGGTTCTGGCATCCATG
AAAAACGCAGCGAAATGCGATAAGTAATGTGAATTGCAAAATTCAGTGAATCATCCAA
TCTTTGAACGCACATTGCGCCCTCTGGTATTCCGGAAGGCATGCCTGTTCGAGCGTCATT
TCAACCCTCAAGCATTGCTTGGTGTTGGGGCACTGCTTTT--------
AACGAAGCAGGCCCTGAAATCTAGTGGCGAGCTCGCTAGGACCCCGAGCGTAATAATT
AAACCCTCCCTTTGGAAGGCCCTGG--
CGGTGCCCTGCCGTTAAACCCCCAACTTCTGAAAATT

>Diaporthe_fusiformis_JZB320156

AACAAGGTCTCCGTTGGTGAACCAGCGGAGGGATCATTGCTG-
GAACGCGCCCCAGGCGCACCCAGAAACCC-TTTGTGAACTTATACC-
TTACTGTTGCCTCGGCGCATGCTGGCCCC--CTG-GGGGTCCCTCACTCC------
GGTGAGGAGCAGGCACGCCGGCGGCCAAGTTAACTCTTGTTTTTACACTGAAACTCTGA
G--
AAAAAACACAAATGAATCAAAACTTTCAACAACGGATCTCTTGGTTCTGGCATCGATG
AAGAACGCAGCGAAATGCGATAAGTAATGTGAATTGCAGAATTCAGTGAATCATCGAA
TCTTTGAACGCACATTGCGCCCTCTGGTATTCCGGAGGGCATGCCTGTTCGAGCGTCATT
TCAACCCTCAAGCATTGCTTGGTGTTGGGGCACTGCTTCT----
TACCCAAGAAGCAGGCCCTGAAATCTAGTGGCGAGCTCGCCAGGACCCCGAGCGCAGT
AGTTAAACCCTCGCTTTGGAAGGCCCTGGGCGGTGCCCTGCCGTTAAACCCCCAANN
NNNNNNNNNN

>Diaporthe_gossiae_BRIP_59730a

AACAAGGTCTCCGTTGGTGAACCAGCGGAGGGATCATTGCTG-
GAACGCGCCCCAGGCGCACCCAGAAACCC-TTTGTGAACTTATACC-TT-
TTGTTGCCTCGGCGCA-GCTGGTCTC--TAG-TAGACCCTTTACTGC---
AATGGTAAAGAGACGGCACGCCGGCGGCCAAGTTAACTCTTGTTTTTACACTGAAACTC
TGAG-
AAAAAAACACAAATGAATCAAAACTTTCAACAACGGATCTCTTGGTTCTGGCATCGAT
GAAGAACGCAGCGAAATGCGATAAGTAATGTGAATTGCAGAATTCAGTGAATCATCGA
ATCTTTGAACGCACATTGCGCCCTCTGGTATTCCGGAGGGCATGCCTGTTCGAGCGTCAT
TTCAACCCTCAAGCATTGCTTGGTGTTGGGGCACTGCTTTT--------
TACCAAGCAGGCCCTGAAATCTAGTGGCGAGCTCGCCAGGACCCCGAGCGCAGTAGTT
AAACCCTCGCTTTGGAAGGCCCTGG--
CGGTGCCCTGCCGTTAAACCCCCNNNNNNNNNNNNNN

>Diaporthe_guangxiensis_JZB320094

NNNNNNNNNNNNNNNNNNNNNNNNNNNNNNNNNNNAATTGCTG-GGACGCG-
CCTACGCGCACCCAG-AACCC-TTTGTGAACTTATACC-
TTACTGTTGCCTCGGCGCATGCTGGCCCC--TAT-GGGGTCCCCTG--GA------
GACAGGGAGCAGGCACGCCGGCGGCCAAGTTAACTCTTGTTTTTACACTGAAACTCTGA
G--
AAAAAACACAAATGAATCAAAACTTTCAACAACGGATCTCTTGGTTCTGGCATCGATG
AAGAACGCAGCGAAATGCGATAAGTAATGTGAATTGCAGAATTCAGTGAATCATCGAA
TCTTTGAACGCACATTGCGCCCTCTGGTATTCCGGAGGGCATGCCTGTTCGAGCGTCATT
TCAACCCTCAAGCATTGCTTGGTGTTGGGGCACTGCTTTT--------
AACGAAGCAGGCCCTGAAATCTAGTGGCGAGCTCGCCAGGACCCCGAGCGCAGTAGTT
AAACCCTCGCTCTGGAAGGCCCTGG--
CGGCGCCCTGCCGTTAAACCCCCAACTTTTGAAAATT

>Diaporthe_hongkongensis_CBS_115448

AACAAGGTCTCCGTTGGTGAACCAGCGGAGGGATCATTGCTG-
GAACGCGCCCCAGGCGCACCCAGAAACCC-TTTGTGAACTTATACC-
TTACTGTTGCCTCGGCGCATGCTGGCCCC--CCCGGGGGCCCCTCG--GA------
GACGAGGAGCAGGCACGCCGGCGGCCAAGTTAACTCTTGTTTTTACACTGAAACTCTGA
G--
AAAAAACACAAATGAATCAAAACTTTCAACAACGGATCTCTTGGTTCTGGCATCGATG
AAGAACGCAGCGAAATGCGATAAGTAATGTGAATTGCAGAATTCAGTGAATCATCGAA
TCTTTGAACGCACATTGCGCCCTCTGGTATTCCGGAGGGCATGCCTGTTCGAGCGTCATT
TCAACCCTCAAGCATTGCTTGGTGTTGGGGCACTGCTTTT------
ACCCAAAAGCAGGCCCTGAAATCTAGTGGCGAGCTCGCCAGGACCCCGAGCGCAGTA
GTTAAACCCTCGCTCTGGAAGGCCCTGG--
CGGTGCCCTGCCGTTAAACCCCCAACTTTTGAAAATT

>Diaporthe_howardiae_BRIP_59697a

AACAAGGTCTCCGTTGGTGAACCAGCGGAGGGATCATTGCTG-
GAACGCGCCCCAGGCGCACCCAGAAACCC-TTTGTGAACTTATACC-
TTACTGTTGCCTCGGCGCATGCTGGCCCC--CTC-GGGGCCCCCTG--GA------
GACAGGGAGCAGGCACGCCGGCGGCCAAGTTAACTCTTGTTTTTACACTGAAACTCTGA
G--
AAAAAACACAAATGAATCAAAACTTTCAACAACGGATCTCTTGGTTCTGGCATCGATG
AAGAACGCAGCGAAATGCGATAAGTAATGTGAATTGCAGAATTCAGTGAATCATCGAA
TCTTTGAACGCACATTGCGCCCTCTGGTATTCCGGAGGGCATGCCTGTTCGAGCGTCATT
TCAACCCTCAAGCATTGCTTGGTGTTGGGGCACTGCTTTT--------
TACCAAGCAGGCCCTGAAATCTAGTGGCGAGCTCGCCAGGACCCCGAGCGCAGTAGTT
AAACCCTCGCTTTGGAAGGCCCTGG--
CGGTGCCCTGCCGTTAAACCCCCAACTTTTGAAAANN

>Diaporthe_huangshanensis_CNUCC_201903

NNNNNNNNNNNNNNNNNNNNNNNNNNNNNGGGGGCTTGGCT--GGACGCG-
ACTAGGCGCACCCAGAAACCC-TTTGTGAACTTATACC-TT-
TTGTTGCCTCGGCGCATGCTGGCCTC--TAG-TAGGCCCCTCACCCC------
GGTGAGGAGACGGCACGCCGGCGGCCAAGTTAACTC-
TGTTTTTACACTGAAACTCTGAG--
AAAAAACACAAATGAATCAAAACTTTCAACAACGGATCTCTTGGTTCTGGCATCGATG
AAGAACGCAGCGAAATGCGATAAGTAATGTGAATTGCAGAATTCAGTGAATCATCGAA
TCTTTGAACGCACATTGCGCCCTCTGGTATTCCGGAGGGCATGCCTGTTCGAGCGTCATT
TCAACCCTCAAGCATTGCTTGGTGTTGGGGCACTGCTTTT--------
AACGAAGCAGGCCCTGAAATCTAGTGGCGAGCTCGCCAGGACCCCGAGCGTAGTAGTT
AAACCCTCGCTCTGGAAGGCCCTGG--
CGGTGCCCTGCCGTTAAACCCCCAACTTCTGAAAATT

>Diaporthe_hunanensis_HNZZ023

NNNNNNNNNNNNNNNNNNNNNNNNNNNNNNNNNNNNNNNNNNNNNNNNNNNNNNN
NNNNNNNNNNNNNNNNNNNNCCC-TTTGTGAACTTATACC-
TTACTGTTGCCTCGGCGCATGCTGGCCCC--CTC-GGGGCCCCCTG--GA------
GACAGGGAGCAGGCACGCCGGCGGCCAAGTTAACTCTTGTTTTTACACTGAAACTCTGA
G--
AAAAAACACAAATGAATCAAAACTTTCAACAACGGATCTCTTGGTTCTGGCATCGATG
AAGAACGCAGCGAAATGCGATAAGTAATGTGAATTGCAGAATTCAGTGAATCATCGAA
TCTTTGAACGCACATTGCGCCCTCTGGTATTCCGGAGGGCATGCCTGTTCGAGCGTCATT
TCAACCCTCAAGCATTGCTTGGTGTTGGGGCACTGCTTTT--------
AACGAAGCAGGCCCTGAAATCTAGTGGCGAGCTCGCTAGGACCCCGAGCGTAGTAGTT
AAACCCTCGCTTTGGAAGGCCCTGG--
CGGTGCCCTGCCGTTAAACCCCCAACTTTTGAAANNN

>Diaporthe_krabiensis_MFLUCC_17_2481

NNNNNNNNNNCCGTTGGTGAACCAGCGGAGGGATCATTGCTG-
GAACGCGCCCCAGGCGCACCCAGAAACCC-TTTGTGAACTTATACC-
TTACTGTTGCCTCGGCGCATGCTGGCCCC--CTC-GGGGCCCCCTG--GA------
GACAGGGAGCAGGCACGCCGGCGGCCAAGTTAACTCTTGTTTTTACACTGAAACTCTGA
G--
AAAAAACACAAATGAATCAAAACTTTCAACAACGGATCTCTTGGTTCTGGCATCGATG
AAGAACGCAGCGAAATGCGATAAGTAATGTGAATTGCAGAATTCAGTGAATCATCGAA
TCTTTGAACGCACATTGCGCCCTCTGGTATTCCGGAGGGCATGCCTGTTCGAGCGTCATT
TCAACCCTCAAGCATTGCTTGGTGTTGGGGCACTGCTTTT--------
AACGAAGCAGGCCCTGAAATCTAGTGGCGAGCTCGCCAGGACCCCGAGCGCAGTAGTT

AAACCCTCGCTCTGGAAGGCCCTGG--
CGGTGCCCTGCCGTTAAACCCCCAACTTCTGAAAATT

>Diaporthe_limonicola_CBS_142549

NNNNNNNNNNNNNNNNNNNNNNNNNNNNNNNNNTCATTGCTG-
GAACGCGCCCCAGGCGCACCCAGAAACCC-TTTGTGAACTTATACC-
TTACTGTTGCCTCGGCGCATGCTGGCCTC--TAG-TAGGCCCCTCACCCC------
GGTGAGGAGACGGCACGCCGGCGGCCAAGTTAACTCTTGTTTTTACACTGAAACTCTGA
G--
AAAAAACACAAATGAATCAAAACTTTCAACAACGGATCTCTTGGTTCTGGCATCGATG
AAGAACGCAGCGAAATGCGATAAGTAATGTGAATTGCAGAATTCAGTGAATCATCGAA
TCTTTGAACGCACATTGCGCCCTCTGGTATTCCGGAGGGCATGCCTGTTCGAGCGTCATT
TCAACCCTCAAGCACTGCTTGGTGTTGGGGCACTGCTTTT--------
AACGAAGCAGGCCCTGAAATCTAGTGGCGAGCTCGCCAGGACCCCGAGCGCAGTAGTT
AAACCCTCGCTCTGGAAGGCCCTGG--
CGGTGCCCTGCCGTTAAACCCCCAACTTCTGAAAATT

>Phomopsis_liquidambari_SCHM_3621

NNNNNNNNNNNNNNNNNNNNNNCCAGCGGAGGGATCATTGCTG-
GAACGCGCCCTAGGCGCACCCAGAAACCC-TTTGTGAACTTATACC-
TTACTGTTGCCTCGGCGCATGCTGGCCCC--CTC-GGGGCCCCCTG--GA------
GACAGGGAGCAGGCACGCCGGCGGCCAAGTTAACTCTTGTTTTTACACTGAAACTCTGA
G-
AAAAAAACACAAATGAATCAAAACTTTCAACAACGGATCTCTTGGTTCTGGCATCGAT
GAAGAACGCAGCGAAATGCGATAAGTAATGTGAATTGCAGAATTCAGTGAATCATCGA
ATCTTTGAACGCACATTGCGCCCTCTGGTATTCCGGAGGGCATGCCTGTTCGAGCGTCAT
TTCAACCCTCAAGCATTGCTTGGTGTTGGGGCACTGCCTGT--------
AAAAGGGCAGGCCCTGAAATCTAGTGGCGAGCTCGCTAGGACCCCGAGCGTAGTAGTT
AAACCCTCGCTTTGGAAGGCCCTGG--
CGGTGCCCTGCCGTTAAACCCCCAACTTTTGAAAATT

>Diaporthe_litchicola_BRIP_54900

AACAAGGTCTCCGTTGGTGAACCAGCGGAGGGATCATTGCTG-
GAACGCGCCCCAGGCGCACCCAGAAACCC-TTTGTGAACTTATACC-TT-
TTGTTGCCTCGGCGCATGCTGGCCTC--TAG-TAGGCCCCTCACCCC------
GGTGAGGAGACGGCACGCCGGCGGCCAAAACAACTC-
TGTTTTTACACTGAAACTCTGAG--
AAAAAACACAAATGAATCAAAACTTTCAACAACGGATCTCTTGGTTCTGGCATCGATG
AAGAACGCAGCGAAATGCGATAAGTAATGTGAATTGCAGAATTCAGTGAATCATCGAA
TCTTTGAACGCACATTGCGCCCTCTGGTATTCCGGAGGGCATGCCTGTTCGAGCGTCATT

TCAACCCTCAAGCATTGCTTGGTGTTGGGGCACTGCTTTT--------
AACGAAGCAGGCCCTGAAATCTAGTGGCGAGCTCGCCAGGACCCCGAGCGCAGTAGTT
AAACCCTCGCTCTGGAAGGCCCTGG--
CGGTGCCCTGCCGTTAAACCCCCAACTTTTGAAAATT

>Diaporthe_lithocarpi_CGMCC_3_15175

NNNNNNNNNNNNNNNNNNNNNNNNNNNNNAGGGATCATTGCTG-
GAACGCGCCCCAGGCGCACCCAGAAACCC-TTTGTGAACTTATACC-
TTACTGTTGCCTCGGCGCATGCTGGCCCC--CTCGGGGGCCCCTCG--GA------
GACGAGGAGCAGGCACGCCGGCGGCCAAGTTAACTCTTGTTTTTACACTGAAACTCTGA
G-
AAAAAAACACAAATGAATCAAAACTTTCAACAACGGATCTCTTGGTTCTGGCATCGAT
GAAGAACGCAGCGAAATGCGATAAGTAATGTGAATTGCAGAATTCAGTGAATCATCGA
ATCTTTGAACGCACATTGCGCCCTCTGGTATTCCGGAGGGCATGCCTGTTCGAGCGTCAT
TTCAACCCTCAAGCATTGCTTGGTGTTGGGGCACTGCCTGT--------
AAAAGGGCAGGCCCTGAAATCTAGTGGCGAGCTCGCCAGGACCCCGAGCGCAGTAGTT
AAACCCTCGCTTTGGAAGGCCCTGG--
CGGTGCCCTGCCGTTAAACCCCCAACTTCTGAAAATT

>Diaporthe_meliae_CFCC_53089

NNNNNNNNNNNNNNNNNNNNNNNNNNNNNNNNNNNNNNNNNNNNNNNNNNNNNNNNN
NNNNNNNNNNNNNNNNNNNCCC-TTTGTGAACTTATACC-
TTACTGTTGCCTCGGCGCATGCCGGCCCC--CTC-GGGGCCCCCTG--GA------
GACAGGGAGCAGGCACGCCGGCGGCCAAGTTAACTCTTGTTTTTACACTGAAACTCTGA
G--
AAAAAACACAAATGAATCAAAACTTTCAACAACGGATCTCTTGGTTCTGGCATCGATG
AAGAACGCAGCGAAATGCGATAAGTAATGTGAATTGCAGAATTCAGTGAATCATCGAA
TCTTTGAACGCACATTGCGCCCTCTGGTATTCCGGAGGGCATGCCTGTTCGAGCGTCATT
TCAACCCTCAAGCATTGCTTGGTGTTGGGGCACTGCCTGT--------
AAAAGGGCAGGCCCTGAAATCTAGTGGCGAGCTCGCCAGGACCCCGAGCGCAGTAGTT
AAACCCTCGCTTTGGAAGGCCCTGG--
CGGTGNNNNNNNNNNNNNNNNNNNNNNNNNNNNNNNNN

>Diaporthe_melitensis_CBS_142551

NNNNNNNNNNNNNNNNNNNNNNNNNNNNNNNNNNNNNNNNNNNNNNNNNNNNNNNNN
NNNNNGCGCACCCAGAAACCC-TTTGTGAACTTATACC-TT-
TTGTTGCCTCGGCGCATGCTGGCCCC--CTC-GGGGCCCCCTG--GA------
GACAGGGAGCAGGCACGCCGGCGGCCAAGTTAACTCTTGTTTTTACACTGAAACTCTGA
G--
AAAAAACACAAATGAATCAAAACTTTCAACAACGGATCTCTTGGTTCTGGCATCGATG

AAGAACGCAGCGAAATGCGATAAGTAATGTGAATTGCAGAATTCAGTGAATCATCGAA
TCTTTGAACGCACATTGCGCCCTCTGGTATTCCGGAGGGCATGCCTGTTCGAGCGTCATT
TCAACCCTCAAGCACTGCTTGGTGTTGGGGCACTGCTTCT--------
AACGAAGCAGGCCCTGAAATCTAGTGGCGAGCTCGCCAGGACCCCGAGCGCAGTAGTT
AAACCCTCGCTCTGGAAGGCCCTGG--
CGGTGCCCTGCCGTTAAACCCCCAACTTCTGAAAATT

>Diaporthe_millettiae_GUCC9167

AACAAGGTCTCCGTTGGTGAACCAGCGGAGGGATCATTGCTG-
GAACGCGCCCCAGGCGCACCCAGAAACCC-TTTGTGAACTTATACC-TT-
TTGTTGCCTCGGCGCATGCTGGTCTC--TAG-TAGGCCCCTCACCCC------
GGTGAGGAGACGGCACGCCGGCGGCCAAGTTAACTCTTGTTTTTACACTGAAACTCTGA
G--
AAAAAACACAAATGAATCAAAACTTTCAACAACGGATCTCTTGGTTCTGGCATCGATG
AAGAACGCAGCGAAATGCGATAAGTAATGTGAATTGCAGAATTCAGTGAATCATCGAA
TCTTTGAACGCACATTGCGCCCTCTGGTATTCCGGAGGGCATGCCTGTTCGAGCGTCATT
TCAACCCTCAAGCATTGCTTGGTGTTGGGGCACTGCTTTT--------
AACGAAGCAGGCCCTGAAATCTAGTGGCGAGCTCGCCAGGACCCCGAGCGCAGTAGTT
AAACCCTCGCTTTGGAAGGCCCTGG--
CGGTGCCCTGCCGTTAAACCCCCAACTTCTGAAAATT

>Diaporthe_musigena_CBS_129519

AACAAGGTCTCCGTTGGTGAACCAGCGGAGGGATCATTGCTG-
GAACGCGCCCCAGGCGCACCCAGAAACCC-TTTGTGAACTTATACC-TT-
TTGTTGCCTCGGCGCATGCTGGCCTC--TAG-TAGGCCCCTCACCCC------
GGTGAGGAGACGGCACGCCGGCGGCCAAAACAACTC-
TGTTTTTACACTGAAACTCTGAG--
AAAAAACACAAATGAATCAAAACTTTCAACAACGGATCTCTTGGTTCTGGCATCGATG
AAGAACGCAGCGAAATGCGATAAGTAATGTGAATTGCAGAATTCAGTGAATCATCGAA
TCTTTGAACGCACATTGCGCCCTCTGGTATTCCGGAGGGCATGCCTGTTCGAGCGTCATT
TCAACCCTCAAGCATTGCTTGGTGTTGGGGCACTGCTTTT--------
TACCAAGCAGGCCCTGAAATCTAGTGGCGAGCTCGCCAGGACCCCGAGCGCAGTAGTT
AAACCCTCGCTCTGGAAGGCCCTGG--
CGGTGCCCTGCCGTTAAACCCCCAACTTCTGAAAATT

>Diaporthe_norfolkensis_BRIP_59718a

NNNNNNNNNNNNNNNNNNNNNNNNNNNNNNNNNNNNNNNNNNNNNNNNNNNNNNNN
NNNNNNNNNNCCCAGAAACCC-TTTGTGAACTTATACC-TT-TTGTTGCTTGGGCGC-
GGCTGTTTTT--TTA-CAGGCCCTTTGCTAT---
ACTAGCAAAGAGACGGCACGCCGGCGGCCAAATCAACTC-

TGTTTTTACACTGAAACTCTGAG--
AAAAAACACAAATGAATCAAAACTTTCAACAACGGATCTCTTGGTTCTGGCATCGATG
AAGAACGCAGCGAAATGCGATAAGTAATGTGAATTGCAGAATTCAGTGAATCATCGAA
TCTTTGAACGCACATTGCGCCCTCTGGTATTCCGGAGGGCATGCCTGTTCGAGCGTCATT
TCAACCCTCAAGCACTGCTTGGTGTTGGGGCACTGCTTTT--------
CACGAAGCAGGCCCTGAAATCTAGTGGCGAGCTCGCCAGGACCCCGAGCGCAGTAGTT
AAACCCTCGCTCTGGAAGGCCCTGG--CGGTGCCCTGCCGTTAAACCCCCAACTT-
TGAAAATT

>Diaporthe_oculi_HHUF_30565

NNNNNNNNNNNNNNNNNNNNNNNNNNNNNNNATCATTGCTG-
GAACGCGCCCCAGGCGCACCCAGAAACCC-TTTGTGAACTTATACC-TT-
TTGTTGCCTCGGCGCATGCTGGTCTC--TAG-TAGGCCCCTCACCCC------
GGTGAGGAGACGGCACGCCGGCGGCCAAGTTAACTCTTGTTTTTACACTGAAACTCTGA
G-
AAAAAAACACAAATGAATCAAAACTTTCAACAACGGATCTCTTGGTTCTGGCATCGAT
GAAGAACGCAGCGAAATGCGATAAGTAATGTGAATTGCAGAATTCAGTGAATCATCGA
ATCTTTGAACGCACATTGCGCCCTCTGGTATTCCGGAGGGCATGCCTGTTCGAGCGTCAT
TTCAACCCTCAAGCATTGCTTGGTGTTGGGGCACTGCTTTT--------
TACCAAGCAGGCCCTGAAATCTAGTGGCGAGCTCGCCAGGACCCCGAGCGCAGTAGTT
AAACCCTCGCTCTGGAAGGCCCTGG--
CGGTGCCCTGCCGTTAAACCCCCAACTTCTGAAAATT

>Diaporthe_osmanthi_GUCC9165

AACAAGGTCTCCGTTGGTGAACCAGCGGAGGGATCATTGCTG-
GAACGCGCCCTAGGCGCACCCAGAAACCC-TTTGTGAACTTATACC-
TTACTGTTGCCTCGGCGCATGCTGGCCCC--CTT-GGGGTCCCCTG--GA------
GACAGGGAGCAGGCACGCCGGCGGCCAAGTTAACTCTTGTTTTTACACTGAAACTCTGA
G--
AAAAAACACAAATGAATCAAAACTTTCAACAACGGATCTCTTGGTTCTGGCATCGATG
AAGAACGCAGCGAAATGCGATAAGTAATGTGAATTGCAGAATTCAGTGAATCATCGAA
TCTTTGAACGCACATTGCGCCCTCTGGTATTCCGGAGGGCATGCCTGTTCGAGCGTCATT
TCAACCCTCAAGCATTGCTTGGTGTTGGGGCACTGCCTGT--------
GAAAGGGCAGGCCCTGAAATCTAGTGGCGAGCTCGCCAGGACCCCGAGCGCAGTAGTT
AAACCCTCGCTTTGGAAGGCCCTGG--CGGTGCCCTGCCGTTAAACCCCCAACTT-
TGAAAATT

>Diaporthe_pandanicola_MFLUCC_17_0607

AACAAGGTCTCCGTTGGTGAACCAGCGGAGGGATCATTGCTG-
GAACGCGCCCCAGGCGCACCCAGAAACCC-TTTGTGAACTTATACC-

TTACTGTTGCCTCGGCGCTTGCTGGTCTC--TAG-TAGGCCCTTTACCGC---
AATGGTAAAGAGACGGCACGCCGGCGGCCAAAACAACTC-
TGTTTTTACACTGAAACTCTGAG--
AAAAAACACAAATGAATCAAAACTTTCAACAACGGATCTCTTGGTTCTGGCATCGATG
AAGAACGCAGCGAAATGCGATAAGTAATGTGAATTGCAGAATTCAGTGAATCATCGAA
TCTTTGAACGCACATTGCGCCCTCTGGTATTCCGGAGGGCATGCCTGTTCGAGCGTCATT
TCAACCCTCAAGCATTGCTTGGTGTTGGGGCACTGCCTGT--------
AAAAGGGCAGGCCCTGAAATCTAGTGGCGAGCTCGCCAGGACCCCGAGCGCAGTAGTT
AAACCCTCGCTTTGGAAGGCCCTGG--
CGGTGCCCTGCCGTTAAACCCCCAACTTTTGAAAATT

>Diaporthe_pascoei_BRIP_54847

AACAAGGTCTCCGTTGGTGAACCAGCGGAGGGATCATTGCTG-
GAACGCGCCCCAGGCGCACCCAGAAACCC-TTTGTGAACTTATACC-TT-
TTGTTGCCTCGGCGCATGCTGGTCTC--TAG-TAGGCCCCTCACCCC------
GGTGAGGAGACGGCACGCCGGCGGCCAAAACAACTCTTGTTTTTACACTGAAACTCTG
AG--
AAAAAACACAAATGAATCAAAACTTTCAACAACGGATCTCTTGGTTCTGGCATCGATG
AAGAACGCAGCGAAATGCGATAAGTAATGTGAATTGCAGAATTCAGTGAATCATCGAA
TCTTTGAACGCACATTGCGCCCTCTGGTATTCCGGAGGGCATGCCTGTTCGAGCGTCATT
TCAACCCTCAAGCATTGCTTGGTGTTGGGGCACTGCTTTT--------
TACCAAGCAGGCCCTGAAATCTAGTGGCGAGCTCGCCAGGACCCCGAGCGCAGTAGTT
AAACCCTCGCTCTGGAAGGCCCTGG--
CGGTGCCCTGCCGTTAAACCCCCAACTTTTGAAAATT

>Diaporthe_perseae_CBS_151_73

AACAAGGTCTCCGTTGGTGAACCAGCGGAGGGATCATTGCTG-
GAACGCGCCCCAGGCGCACCCAGAAACCC-TTTGTGAACTTATACC-TT-
TTGTTGCCTCGGCGC-TGCTGGTCTT--CA--CAGGCCCTTTGCTTC----
ACAGCAAAGAGACGGCACGCCGGCGGCCAAGTCAACTCTTGTTTTTACACTGAAACTC
TGAG--
AAAAAACACAAATGAATCAAAACTTTCAACAACGGATCTCTTGGTTCTGGCATCGATG
AAGAACGCAGCGAAATGCGATAAGTAATGTGAATTGCAGAATTCAGTGAATCATCGAA
TCTTTGAACGCACATTGCGCCCTCTGGTATTCCGGAGGGCATGCCTGTTCGAGCGTCATT
TCAACCCTCAAGCATTGCTTGGTGTTGGGGCACTGCTTTT--------
AACGAAGCAGGCCCTGAAATCTAGTGGCGAGCTCGCCAGGACCCCGAGCGCAGTAGTT
AAACCCTCGCTCTGGAAGGCCCTGG--
CGGTGCCCTGCCGTTAAACCCCCAACTTCTGAAAATT

>Diaporthe_pescicola_MFLUCC_16_0105

AACAAGGTCTCCGTTGGTGAACCAGCGGAGGGATCATTGCTG-
GAACGCGCCCTAGGCGCACCCAGAAACCC-TTTGTGAACTTATACC-TT-
TTGTTGCCTCGGCGCATGCTGGCCCC--TAT-GGGGTCCCCTG--GA------
GACAGGGAGCAGGCACGCCGGCGGCCAAGTTAACTCTTGTTTTTACACTGAAACTCTGA
G--
AAAAAACACAAATGAATCAAAACTTTCAACAACGGATCTCTTGGTTCTGGCATCGATG
AAGAACGCAGCGAAATGCGATAAGTAATGTGAATTGCAGAATTCAGTGAATCATCGAA
TCTTTGAACGCACATTGCGCCCTCTGGTATTCCGGAGGGCATGCCTGTTCGAGCGTCATT
TCAACCCTCAAGCATTGCTTGGTGTTGGGGCACTGCTTTT--------
AACGAAGCAGGCCCTGAAATCTAGTGGCGAGCTCGCCAGGACCCCGAGCGTAGTAGTT
AAACCCTCGCTTTGGAAGGCCCTGG--
CGGTGCCCTGCCGTTAAACCCCCAACTTCTGAAAATT

>Phomopsis_phyllanthicola

NNNNNNNNNNNNNNNNNNNTGACCAGCGGAGGGATCATTGCTG-
GAACGCGCCCTAGGCGCACCCAGAAACCC-TTTGTGAACTTATACC-
TTACTGTTGCCTCGGCGCATGCTGGCCCC--CTT-GGGGTCCCCTG--GA------
GACAGGGAGCAGGCACGCCGGCGGCCAAGTTAACTCTTGTTTTTACACTGAAACTCTGA
G--
AAAAAACACAAATGAATCAAAACTTTCAACAACGGATCTCTTGGTTCTGGCATCGATG
AAGAACGCAGCGAAATGCGATAAGTAATGTGAATTGCAGAATTCAGTGAATCATCGAA
TCTTTGAACGCACATTGCGCCCTCTGGTATTCCGGAGGGCATGCCTGTTCGAGCGTCATT
TCAACCCTCAAGCATTGCTTGGTGTTGGGGCACTGCTTTT--------
AACCAAGCAGGCCCTGAAATCTAGTGGCGAGCTCGCCAGGACCCCGAGCGCAGTAGTT
AAACCCTCGCTTTGGAAGGCCCTGG--
CGGTGCCCTGCCGTTAAACCCCCAACTTCTGAAAATT

>Diaporthe_podocarpi-macrophylli_CGMCC3_18281

NNNNNNNNNNNNNNNNNNNNNNNNNCGGAGGGATCATTGCTG-
GAACGCGCCCCAGGCGCACCCAGAAACCC-TTTGTGAACTTATACC-
TTACTGTTGCCTCGGCGCATGCTGGCCCC--CC--GGGGTCCCCTG--GA------
GACAGGGAGCAGGCACGCCGGCGGCCAAGTTAACTCTTGTTTTTACACTGAAACTCTGA
GAAAAAAAACACAAATGAATCAAAACTTTCAACAACGGATCTCTTGGTTCTGGCATCG
ATGAAGAACGCAGCGAAATGCGATAAGTAATGTGAATTGCAGAATTCAGTGAATCATC
GAATCTTTGAACGCACATTGCGCCCTCTGGTATTCCGGAGGGCATGCCTGTTCGAGCGT
CATTTCAACCCTCAAGCATTGCTTGGTGCTGGGGCACTGCCTGT--------
GAAAGGGCAGGCCCTGAAATCTAGTGGCGAGCTCGCCAGGACCCCGAGCGCAGTAGTT
AAACCCTCGCTTTGGAAGGCCCTGG--
CGGTGCCCTGCCGTTAAACCCCCAACTTCTGAAAATT

>Diaporthe_pseudomangiferae_CBS_101339

AACAAGGTCTCCGTTGGTGAACCAGCGGAGGGATCATTGCTG-
GAACGCGCCCCAGGCGCACCCAGAAACCC-TTTGTGAACTTATACC-TT-
TTGTTGCCTCGGCGCATGCTGGCCTC--TAG-TAGGCCCCTCACCCC------
GGTGAGGAGAAGGCACGCCGGCGGCCAAGTTAACTCTTGTTTTTACACTGAAACTCTGA
G--
AAAAAACACAAATGAATCAAAACTTTCAACAACGGATCTCTTGGTTCTGGCATCGATG
AAGAACGCAGCGAAATGCGATAAGTAATGTGAATTGCAGAATTCAGTGAATCATCGAA
TCTTTGAACGCACATTGCGCCCTCTGGTATTCCGGAGGGCATGCCTGTTCGAGCGTCATT
TCAACCCTCAAGCATTGCTTGGTGTTGGGGCACTGCTTCT--------
AACGAAGCAGGCCCTGAAATCTAGTGGCGAGCTCGCCAGGACCCCGAGCGTAGTAGTT
AAACCCTCGCTCTGGAAGGCCCTGG--
CGGTGCCCTGCCGTTAAACCCCCAACTTTTGAAAATT

>Diaporthe_pseudooculi_HHUF_30617

NNNNNNNNNNNNNNNNGGTGAACCTGCGGAGGGATCATTGCTG-
GAACGCGCCCTAGGCGCACCCAGAAACCC-TTTGTGAACTTATACC-
TTACTGTTGCCTCGGCGCATGCTGGCCCC--CTC-GGGGTCCCCTG--GA------
GACAGGGAGCAGGCACGCCGGCGGCCAAGTTAACTCTTGTTTTTACACTGAAACTCTGA
G-
AAAAAAACACAAATGAATCAAAACTTTCAACAACGGATCTCTTGGTTCTGGCATCGAT
GAAGAACGCAGCGAAATGCGATAAGTAATGTGAATTGCAGAATTCAGTGAATCATCGA
ATCTTTGAACGCACATTGCGCCCTCTGGTATTCCGGAGGGCATGCCTGTTCGAGCGTCAT
TTCAACCCTCAAGCATTGCTTGGTGTTGGGGCACTGCCTGT--------
AAAAGGGCAGGCCCTGAAATCTAGTGGCGAGCTCGCTAGGACCCCGAGCGTAGTAGTT
AAACCCTCGCTTTGGAAGGCCCTGG--
CGGTGCCCTGCCGTTAAACCCCCAACTTTTGAAAATT

>Diaporthe_pseudophoenicicola_CBS_462_69

AACAAGGTCTCCGTTGGTGAACCAGCGGAGGGATCATTGCTG-
GAACGCGCCCTAGGCGCACCCAGAAACCC-
TTTGTGAACTTATACCTTTACTGTTGCCTCGGCGCATGCTGGCCCC--CTC-
GGGGTCCCCTG--GA------
GACAGGGAGCAGGCACGCCGGCGGCCAAGTTAACTCTTGTTTTTACACTGAAACTCTGA
G--
AAAAAACACAAATGAATCAAAACTTTCAACAACGGATCTCTTGGTTCTGGCATCGATG
AAGAACGCAGCGAAATGCGATAAGTAATGTGAATTGCAGAATTCAGTGAATCATCGAA
TCTTTGAACGCACATTGCGCCCTCTGGTATTCCGGAGGGCATGCCTGTTCGAGCGTCATT
TCAACCCTCAAGCATTGCTTGGTGTTGGGGCACTGCTTTT--------
CACCAAGCAGGCCCTGAAATCTAGTGGCGAGCTCGCCAGGACCCCGAGCGTAGTAGTT

AAACCCTCGCTTTGGAAGGCCCTGG--
CGGTGCCCTGCCGTTAAACCCCCAACTTCTGAAAATT

>Diaporthe_pterocarpicola_MFLUCC_10_0580a

NNNNNNNNNNNNNNNNNNNNNNNNNNNNNNNNNNNTTATCGA-
GAGGACAGCGCGAGGGCACC------CCC-TTTGTGAACTTATACC-
TTACTGTTGCCTCGGCGCATGCCGGCCCC--CTC-GGGGCCCCCTG--GA------
GACAGGGAGCAGGCACGCCGGCGGCCAAGTTAACTCTTGTTTTTACACTGAAACTCTGA
G--
AAAAAACACAAATGAATCAAAACTTTCAACAACGGATCTCTTGGTTCTGGCATCGATG
AAGAACGCAGCGAAATGCGATAAGTAATGTGAATTGCAGAATTCAGTGAATCATCGAA
TCTTTGAACGCACATTGCGCCCTCTGGTATTCCGGAGGGCATGCCTGTTCGAGCGTCATT
TCAACCCTCAAGCATTGCTTGGTGTTGGGGCACTGCCTGT--------
AAAAGGGCAGGCCCTGAAATCTAGTGGCGAGCTCGCCAGGACCCCGAGCGCAGTAGTT
AAACCCTCGCTTTGGAAGGCCCTGG--
CGGTGCCCTGCCGTTAAACCCCCAACTTTTGAAAATT

>Diaporthe_rhodomyrti_CFCC_53101

NNNNNNNNNNNNNNNNNNNNNNNNNNNNNNNNNNNNNNNNNNNNNNNNNNNNN
NNNNNNNNNNNNNNNNNNNNCCC-TTTGTGAACTTATACC-
TTACTGTTGCCTCGGCGCATGCCGGCCCC--CCCGGGGGCCCCTCG--GA------
GACGAGGAGCAGGCACGCCGGCGGCCAAGTTAACTCTTGTTTTTACACTGAAACTCTGA
G-
AAAAAAACACAAATGAATCAAAACTTTCAACAACGGATCTCTTGGTTCTGGCATCGAT
GAAGAACGCAGCGAAATGCGATAAGTAATGTGAATTGCAGAATTCAGTGAATCATCGA
ATCTTTGAACGCACATTGCGCCCTCTGGTATTCCGGAGGGCATGCCTGTTCGAGCGTCAT
TTCAACCCTCAAGCATTGCTTGGTGTTGGGGCACTGCTTCT----
TACCCAAGAAGCAGGCCCTGAAATCTAGTGGCGAGCTCGCCAGGACCCCGAGCGCAGT
AGTTAAACCCTCGCTCTGGAAGGCCCTGG--
CGGTGNNNNNNNNNNNNNNNNNNNNNNNNNNNNNNNN

>Diaporthe_salinicola_MFLU18_0533

NNNNNNNNNNCCGTTGGTGCACCAGTGGAGGAATCATTGCTG-
GAACGCACCCCAGGCGCACCCAGAAACCC-TTTGTGAACTTATACC-
TTACTGTTGCCTCGGCGCATACTGGCTCC--CTGGGGGGTCCCTTG--GA------
GCCGAGGAGCAGGCACGCCGGCGGCCAAGTTAAATCTTGTTTTT--
ACTGAAACTCTGAG-
AAAAAAACACAAATGAATCAAAACTTTCAACAACGGATCTCTTGGTTCTGGCATCGAT
GAAGAACGCAGCGAAATGCGATAAGTAATGTGAATTGCAGAATTCAGTGAATCATCGA
ATCTTTGAACGCACATTGCGCCCTCTGGTATTCCGGAGGGCATGCCTGTTCGAGCGTCAT

TTCAACCCTCAAGCCCTGCTTGGTGTTGGGGCACTGCCTGT--------
ANNNNNNNNNNNNNNNNNNNNNNNNNNNNNNNNNNNNNNNNNNNNNNNNNNNNNNN
NNNNNNNNNNNNNNNNNNNNNNNNNNNNNNNNNNNNNNNNNNNNNNNNNNNNNNNN
NNNNNNNNNNNNNNNNNNNNNN

>Diaporthe_schimae_CFCC_53103

NNNNNNNNNNNNNNNNNNNNNNNNNNNNNNNNNNNNNNNNNNNNNNNNNNNNNNNN
NNNNNNNNNNNNNNNNNNNCCC-TTTGTGAACTTATACC-TT-
TTGTTGCCTCGGCGCATGCTGGTCTC--TAG-TAGGCCCCTCACCCC------
GGTGAGGAGACGGCACGCCGGCGGCCAAGTTAACTCTTGTTTTTACACTGAAACTCTGA
G--
AAAAAACACAAATGAATCAAAACTTTCAACAACGGATCTCTTGGTTCTGGCATCGATG
AAGAACGCAGCGAAATGCGATAAGTAATGTGAATTGCAGAATTCAGTGAATCATCGAA
TCTTTGAACGCACATTGCGCCCTCTGGTATTCCGGAGGGCATGCCTGTTCGAGCGTCATT
TCAACCCTCAAGCATTGCTTGGTGTTGGGGCACTGCTTTT--------
AACGAAGCAGGCCCTGAAATCTAGTGGCGAGCTCGCCAGGACCCCGAGCGCAGTAGTT
AAACCCTCGCTCTGGAAGGCCCTGG--
CGGTGNNNNNNNNNNNNNNNNNNNNNNNNNNNNNNNNN

>Diaporthe_searlei_BRIP_66528

AACAAGGTCTCCGTTGGTGAACCAGCGGAGGGATCATTGCTG-
GAACGCGCCCCAGGCGCACCCAGAAACCC-TTTGTGAACTTATACC-
TTACTGTTGCCTCGGCGCATGCTGGCCCC--TCT-GGGGTCCCCTG--GA------
GACAGGGAGCAGGCACGCCGGCGGCCAAGTTAACTCTTGTTTTTACACTGAAACTCTGA
G--
AAAAAACACAAATGAATCAAAACTTTCAACAACGGATCTCTTGGTTCTGGCATCGATG
AAGAACGCAGCGAAATGCGATAAGTAATGTGAATTGCAGAATTCAGTGAATCATCGAA
TCTTTGAACGCACATTGCGCCCTCTGGTATTCCGGAGGGCATGCCTGTTCGAGCGTCATT
TCAACCCTCAAGCATTGCTTGGTGTTGGGGCACTGCCTGT--------
AAAAGGGCAGGCCCTGAAATCTAGTGGCGAGCTCGCCAGGACCCCGAGCGTAGTAGTT
AAACCCTCGCTTTGGAAGGCCCTGG--
CGGTGCCCTGCCGTTAAACCCCCAACTTTTGAAAATT

>Diaporthe_sennae_CFCC_51636

NNNNNNNNNNNNNNNNNNNNNNNNNNNNNNNNNNNNNNNNNNNNNNNNNNNNNNNN
NNNNNNNNNNNNNNNNNNNCCC-TTTGTGAACTTATACC-TT-
TTGTTGCCTCGGCGCATGCTGGCCTC--TAG-TAGGCCCCTTACTTC----
ACAGTAAGGAGACGGCACGCCGGCGGCCAAATCAACTCTTGTTTTTACACTGAAACTCT
GAG--
AAAAAACACAAATGAATCAAAACTTTCAACAACGGATCTCTTGGTTCTGGCATCGATG

AAGAACGCAGCGAAATGCGATAAGTAATGTGAATTGCAGAATTCAGTGAATCATCGAA
TCTTTGAACGCACATTGCGCCCTCTGGTATTCCGGAGGGCATGCCTGTTCGAGCGTCATT
TCAACCCTCAAGCATTGCTTGGTGTTGGGGCACTGCTTTC--------
AACGAAGCAGGCCCTGAAATCTAGTGGCGAGCTCGCCAGGACCCCGAGCGCAGTAGTT
AAACCCTCGCTTTGGAAGGCCCTGG--
CGGTGCCCTGCCGTTAAACCCCCAACTTTTGAAANNN

>Diaporthe_africana_STMA18294

AACAAGGTCTCCGTTGGTGAACCAGCGGAGGGATCATTGCTG-
GAACGCGCCCCAGGCGCACCCAGAAACCC-TTTGTGAACTTATACC-
TTACTGTTGCCTCGGCGCATGCTGGCCCC--CCCGGGGGCCCCTCG--GA------
GACGAGGAGCAGGCACGCCGGCGGCCAAGTTAACTCTTGTTTTTACACTGAAACTCTGA
G--
AAAAAACACAAATGAATCAAAACTTTCAACAACGGATCTCTTGGTTCTGGCATCGATG
AAGAACGCAGCGAAATGCGATAAGTAATGTGAATTGCAGAATTCAGTGAATCATCGAA
TCTTTGAACGCACATTGCGCCCTCTGGTATTCCGGAGGGCATGCCTGTTCGAGCGTCATT
TCAACCCTCAAGCATTGCTTGGTGTTGGGGCACTGCTTTT------
ACCCAAAAGCAGGCCCTGAAATCTAGTGGCGAGCTCGCCAGGACCCCGAGCGCAGTA
GTTAAACCCTCGCTTTGGAAGGCCCTGG--
CGGTGCCCTGCCGTTAAACCCCCAACTTTTGAAAATT

>Diaporthe_africana_CBS_150080

AACAAGGTCTCCGTTGGTGAACCAGCGGAGGGATCATTGCTG-
GAACGCGCCCCAGGCGCACCCAGAAACCC-TTTGTGAACTTATACC-
TTACTGTTGCCTCGGCGCATGCTGGCCCC--CCCGGGGGCCCCTCG--GA------
GACGAGGAGCAGGCACGCCGGCGGCCAAGTTAACTCTTGTTTTTACACTGAAACTCTGA
G--
AAAAAACACAAATGAATCAAAACTTTCAACAACGGATCTCTTGGTTCTGGCATCGATG
AAGAACGCAGCGAAATGCGATAAGTAATGTGAATTGCAGAATTCAGTGAATCATCGAA
TCTTTGAACGCACATTGCGCCCTCTGGTATTCCGGAGGGCATGCCTGTTCGAGCGTCATT
TCAACCCTCAAGCATTGCTTGGTGTTGGGGCACTGCTTTT------
ACCCAAAAGCAGGCCCTGAAATCTAGTGGCGAGCTCGCCAGGACCCCGAGCGCAGTA
GTTAAACCCTCGCTTTGGAAGGCCCTGG--
CGGTGCCCTGCCGTTAAACCCCCAACTTTTGAAAATT

>Diaporthe_spinosa_PSCG_383

NNNNNNNNNNNNNNNNNNNNNNNNNNNNNNNNNNNNNNNNNNNNNNNNNNNNNNN
NNNNNNNNNNNNNNNNNNNNCCC-TTTGTGAACTTATACC-
TTACTGTTGCCTCGGCGCATGCTGGCCCC--CTC-GGGGTCCCCTG--GA------
GACAGGGAGCAGGCACGCCGGCGGCCAAGTTAACTCTTGTTTTTACACTGAAACTCTGA

G-
AAAAAAACACAAATGAATCAAAACTTTCAACAACGGATCTCTTGGTTCTGGCATCGAT
GAAGAACGCAGCGAAATGCGATAAGTAATGTGAATTGCAGAATTCAGTGAATCATCGA
ATCTTTGAACGCACATTGCGCCCTCTGGTATTCCGGAGGGCATGCCTGTTCGAGCGTCAT
TTCAACCCTCAAGCATTGCTTGGTGTTGGGGCACTGCCTGT--------
AAAAGGGCAGGCCCTGAAATCTAGTGGCGAGCTCGCTAGGACCCCGAGCGTAGTAGTT
AAACCCTCGCTTTGGAAGGCCCTGG--
CGGTGCCCTGCCGTTAAACCCCCAACTTTTGAAAATT

>Diaporthe_taoicola_MFLUCC_16_0117

AACAAGGTCTCCGTTGGTGAACCAGCGGAGGGATCATTGCTG-
GAACGCGCCCCAGGCGCACCCAGAAACCC-TTTGTGAACTTATACC-TT-
TTGTTGCCTCGGCGCATGCTGGTCTT--TAG-TAGGCCCCTCACCCC------
GGTGAGGAGACGGCACGCCGGCGGCCAAGTTAACTCTTGTTTTTACACTGAAACTCTGA
G--
AAAAAACACAAATGAATCAAAACTTTCAACAACGGATCTCTTGGTTCTGGCATCGATG
AAGAACGCAGCGAAATGCGATAAGTAATGTGAATTGCAGAATTCAGTGAATCATCGAA
TCTTTGAACGCACATTGCGCCCTCTGGTATTCCGGAGGGCATGCCTGTTCGAGCGTCATT
TCAACCCTCAAGCATTGCTTGGTGTTGGGGCACTGCTTTT--------
AACGAAGCAGGCCCTGAAATCTAGTGGCGAGCTCGCCAGGACCCCGAGCGCAGTAGTT
AAACCCTCGCTCTGGAAGGCCCTGG--CGGTGCCCTGCCGTTAAACCCCCAAC-
TCTGAAAATT

>Diaporthe_tectonigena_MFLUCC_12_0767

AACAAGGTCTCCGTTGGTGAACCAGCGGAGGGATCATTGCTG-
GAACGCGCCCCAGGCGCACCCAGAAACCC-TTTGTGAACTTATACC-
TTACTGTTGCCTCGGCGCATGCTGGCCCC--CCTGGGGGTCCCTCG--GA------
GACGAGGAGCAGGCACGCCGGCGGCCAAGTTAACTCTTGTTTTTACACTGAAACTCTGA
G-
AAAGAAACACAAATGAATCAAAACTTTCAACAACGGATCTCTTGGTTCTGGCATCGAT
GAAGAACGCAGCGAAATGCGATAAGTAATGTGAATTGCAGAATTCAGTGAATCATCGA
ATCTTTGAACGCACATTGCGCCCTCTGGTATTCCGGAGGGCATGCCTGTTCGAGCGTCAT
TTCAACCCTCAAGCATTGCTTGGTGTTGGGGCACTGCTTTT------
ACCCAAAAGCAGGCCCTGAAATCTAGTGGCGAGCTCGCCAGGACCCCGAGCGCAGTA
GTTAAACCCTCGCTCTGGAAGGCCCTGG--CGGTGCCCTGCCGTTAAACCCCCAACTTT-
GAAAATN

>Diaporthe_viniferae_JZB320071

NNNNNNNNNNNNNNNNNNNNNNNNNNNNNNNNNNNTGAAGCTG-GA--GCG-
CCCAGGCGCACCCAGAAACCC-TTTGTGAACTTATACC-TT-

TTGTTGCCTCGGCGCATGCTGGTCTC--TAG-TAGGCCCCTCACCCC------
GGTGAGGAGACGGCACGCCGGCGGCCAAATCAACTC-
TGTTTTTACACTGAAACTCTGAG--
AAAAAACACAAATGAATCAAAACTTTCAACAACGGATCTCTTGGTTCTGGCATCGATG
AAGAACGCAGCGAAATGCGATAAGTAATGTGAATTGCAGAATTCAGTGAATCATCGAA
TCTTTGAACGCACATTGCGCCCTCTGGTATTCCGGAGGGCATGCCTGTTCGAGCGTCATT
TCAACCCTCAAGCATTGCTTGGTGTTGGGGCACTGCCTGT--------
AAAAGGGCAGGCCCTGAAATCTAGTGGCGAGCTCGCCAGGACCCCGAGCGCAGTAGTT
AAACCCTCGCTTTGGAAGGCCCTGG--
CGGTGCCCTGCCGTTAAACCCCCAACTTTTGAAAATT

>Diaporthe_xishuangbanica_CGMCC_3_18282

NNNNNNNNNNNNNNNNNNNNNNNNNNNNNNNNNNNNNNNNNNNNNNNNNNNNNN
NNNNNNNNNNNNNNNNNNNCCC-TTTGTGAACTTATACC-
TTACTGTTGCCTCGGCGCATGCCGGCCCC--CCCGGGGGCCCCTCG--GA------
GACGAGGAGCAGGCACGCCGGCGGCCAAGTTAACTCTTGTTTTTACACTGAAACTCTGA
G-
AAAAAAACACAAATGAATCAAAACTTTCAACAACGGATCTCTTGGTTCTGGCATCGAT
GAAGAACGCAGCGAAATGCGATAAGTAATGTGAATTGCAGAATTCAGTGAATCATCGA
ATCTTTGAACGCACATTGCGCCCTCTGGTATTCCGGAGGGCATGCCTGTTCGAGCGTCAT
TTCAACCCTCAAGCATTGCTTGGTGTTGGGGCACTGCTTTT----
TTAACGAGAAGCAGGCCCTGAAATCTAGTGGCGAGCTCGCCAGGACCCCGAGCGCAGT
AGTTAAACCCTCGCTCTGGAAGGCCCTGG--
CGGTGCCCTGCCGTTAAACCCCCAACTTCTGAAAATT

>Diaporthe_fraxini-angustifoliae_BRIP_54781

AACAAGGTCTCCGTTGGTGAACCAGCGGAGGGATCATTGCTG-
GAACGCGCCCCAGGCGCACCCAGAAACCC-TTTGTGAACTTATACC-TT-
TTGTTGCCTCGGCGCATGCTGGTCTC--TAG-TAGGCCCCTCACCCC------
GGTGAGGAGACGGCACGCCGGCGGCCAAAACAACTC-
TGTTTTTACACTGAAACTCTGAG--
AAAAAACACAAATGAATCAAAACTTTCAACAACGGATCTCTTGGTTCTGGCATCGATG
AAGAACGCAGCGAAATGCGATAAGTAATGTGAATTGCAGAATTCAGTGAATCATCGAA
TCTTTGAACGCACATTGCGCCCTCTGGTATTCCGGAGGGCATGCCTGTTCGAGCGTCATT
TCAACCCTCAAGCATTGCTTGGTGTTGGGGCACTGCTTTT----
CTTGCGAGAAGCAGGCCCTGAAATCTAGTGGCGAGCTCGCCAGGACCCCGAGCGCAGT
AGTTAAACCCTCGCTCTGGAAGGCCCTGG—
CGGTGCCCTGCCGTTAAACCCCCAACTTTTGAAAATT

- Alignment of the *cal* sequences used in the phylogenetic study

>Diaporthe_acuta_PSCG_047
NNNNNNNNNNNNNNNNNNNNNNNNNNNNNNNNNNNNNNNNNNNNNNNNNNAGC
TCCCCGCCCT--
CCTCTGCTGTTGCGCATGATGCTAACGGACCGTTTTCGGTTTGTAGGATAAGGATGGCG
ATGGTTAGTGCGGCCGCCCTCGTTCTTCCTTCTCACACACGCACGCGTCATGCTCGATCC
TCCGCGACGGCCTGCGCGTGCCTAATTTCCAACCAAGCGATTATCACATCTATTGCGAG
TACCATGCTGAGATATGGCGTGCAGGACAAATCACCACCAAGGAGCTCGGCACCGTCA
TGCGATCCCTGGGCCAGAACCCTTCCGAGTCTGAGCTGCAAGATATGATTAACGAGGTC
GACGCCGACAACAACGGCACCATTGACTTCCCTGGTACGTCCAGATATACGCCCA-
TTGAAGTGG
>Diaporthe_amygdali_CBS_126679
CTTCTCCCTCTTTGTAAGTTA---------------
TTTTCAGGAATGATCCCGCAGCCCTCCTCGCCACTGTCGCGCATGATGCTAACGGACCG
TTCTCGGCCTCCAGGATAAGGATGGCGATGGTTAGTGCAGCTGC-
CTCTTCTTCACCTCCCAGCTTCGTACGCGTCACGATCGACCCGCCGCGACGGCTTGCGCG
TGCACATTTTCCAACCAAGC-ACCATAACATCTACTATGAGCTCGATGCTAAG--
ATGACGTGTAGGACAAATCACCACAAAGGAGCTCGGCACGGTCATGCGATCTCTGGGT
CAGAACCCGTCCGAGTCTGAGCTGCAGGATATGATCAACGAGGTCGACGCCGACAACA
ATGGAACCATCGACTTCCCTGGTACGTCCAGATGCTCGCTTGTTTGGAAGGA
>Diaporthe_anhuiensis_CNUCC_201902
NNNNNNNNNNNNNNNNNNCCGAACTATCTCAACAGCGGACACGAGTTCAAGCTCCCC
GCCCT--
CCTCTGCTGTTGCGCATGATGCTAACGGACCGTTTTCGGCTTGTAGGATAAGGATGGCG
ATGGTTAGTGCGACCGCCCTCGTTCTTCCTTCTCACACACGCACGCGTCATGCTCGATCC
TCCGCGACGGCCTGCGCGTGCCTGATTTCCAACCAAACGATTATCACATCTATTGCGAG
TATCATGCTGAGATATGGCGTGCAGGACAAATCACCACAAAGGAGCTCGGCACGGTCA
TGCGATCCCTGGGCCAGAACCCTTCCGAGTCTGAGCTGCAAGATATGATTAACGAGGTC
GACGCCGACAACAACGGCACCATTGACTTCCCTGGTACGTCCAGATATACGCCCA-
TCGAGGGGG
>Diaporthe_arecae_CBS_161_64
CTTCTCCCTCTTTGTAAGTACCATCTCCAACCAGCGGACACGAGTCCAAGCTCCCCGCC
CT--
CCTCTGCTGCTGCGCATGATGCTAACGGACCGTTTTCGGCTTGTAGGATAAGGATGGCG
ATGGTTAGTGCGGCCGCCCTCGTTCTTCCTTCTCACACACGCACGCGTCATGCTCGATCT
TCCGCGACGGCCTGCGCGTGCCTAACCTCCAACTAAGCGATTATCACATTTATTGCGAG
TATCATGCTGAGATATGGTGTGCAGGACAAATCACCACAAAGGAGCTCGGCACGGTCA
TGCGATCCCTGGGCCAGAACCCTTCCGAGTCCGAGCTGCAAGATATGATTAACGAGGTC
GACGCCGACAACAACGGCACCATTGACTTCCCTGGTACGTCCAGATATACGCCCA-
TTGAGAGGG
>Diaporthe_arengae_CBS_114979
NNNNNNNNNNNNNNNNNNNNNNNTCTCCAACCAACGGACACGAGTTCAAGCTCCC
CGC-----

CCTCTACTGTTGCGCATGATGCTAACGGACCGTTTTCGGCTTGTAGGATAAGGATGGCG
ATGGTTAGTGCGGCGGCCCTCGTTCTTCCTTCTCACACACGCACGCGTCATGCTCGATCC
TTCGCGACGGCCTGCGCGTGCCTAACCTCCAACCAAGCGATTATCACATCTATTGCGAG
TATCATGCTGAGATATGGCGTGCAGGACAAATCACCACCAAGGAGCTCGGCACGGTCA
TGCGATCCCTGGGCCAGAACCCTTCCGAGTCCGAGCTGCAAGATATGATTAACGAGGTC
GACGCCGACAACAACGGCACCATTGACTTCCCTGGTACGTCTAGATATACGCCCA-
TCGAGGGGG
>Diaporthe_aseana_MFLUCC_12_0299a
NNNNNNNNNNNNNNNNNNNNNNNNNNNNNNNNNNNNNNNNNNNNNNNNNGC
TCCCTGCCCC--
CCTCTGCCGTTGCGCATGATGCTAACGGACCGTTTTCGGCTTGTAGGATAAGGATGGCG
ATGGTTAGTGCGGCCGCCCTCTTTCTTCCCTCTCACACACGCACGCGTCACGCTCGATCC
GCCGCGACGGCCTGCGCGTGCCTAACTTCCAACCAAGCGATTATCATATCTATTGCGAG
TATCATGCTGAGATATGGCGTGTAGGACAAATCACCACCAAGGAGCTCGGCACGGTCA
TGCGATCCCTGGGACAGAACCCGTCCGAGTCTGAGCTGCAAGATATGATTAACGAGGT
CGACGCTGACAACAACGGCACCATTGACTTCCCTGGTACGTCCAGATATACGCCCG-
TTGAGGGGG
>Diaporthe_camelliaeoleiferae_HNZZ027
NNNNNNNNNNNNNNNNNNNNNNNNNNNNNNNNNNNNNNNNNNNNNNNNNNNN
NNNNNGCCCT--
CCTCTGCTGTTGCGCATGATGCTAACGGACCGTTTTCGGTTTGTAGGATAAGGATGGCG
ATGGTTAGTGCGGCCGCCCTCGTTCTTCCTTCTCACACACGCACGCGTCATGCTCGATCC
TCCGCGACGGCCTGCGCGTGCCTAATTTCCAACCAAGCGATTATCACATCTATTGCGAG
TACCATGCTGAGATATGGCGTGCAGGACAAATCACCACCAAGGAGCTCGGCACCGTCA
TGCGATCCCTGGGCCAGAACCCTTCCGAGTCTGAGCTGCAAGATATGATTAACGAGGTC
GACGCCGACAACAACGGCACCATTGACTTCCCTGGTACGTCCAGATATACGCCCA-
TTGAAGTGG
>Diaporthe_cercidis_CFCC_52565
NNNNNNNNNNNNNNNNNNNNNNNNNNNNNNNNNNNNNNNNNNNNNNNNNNNN
NNNCCGCTCT--
CCTCTGCTGCTGCGCATGATGCTAACGGACCGTTTTCGGCTTGCAGGATAAGGATGGCG
ATGGTTAGTGCGGCCGCCCTCGTTCTTCCTTCTCACACACGCACGCGTCATGCTCGATCC
TCCGCGACGGCCTGCGCGTGCCTAACTTCTAACAATGCGATTATCACATCTATTGCGAG
TATCATGCTGAGATATGGCGTGCAGGACAAATCACCACCAAGGAGCTCGGCACCGTCA
TGCGATCCCTGGGCCAGAACCCTTCCGAGTCTGAGCTGCAAGATATGATTAACGAGGTC
GACGCCGACAACAACGGCACCATTGACTTCCCTGGTACGTCCAGATATACGCCCA-
TTGAGGAGG
>Diaporthe_cf_heveae_2_CBS_681_84
NNNNNNNNNNNNNNNNNNNNNNNNNTCTCCACCAGCCGACACGAGTTCAAGCTCCC
CGCCCT--
CCTCTACTGTTGCGCATGATGCTAACGGACCGTTTTCGGCTTGTAGGATAAGGATGGCG
ATGGTTAGTGCGGCCGCCCTCTTTCTTCCCTCTCACACACGCACGCGTCATGCTCGATCC
GCCGCGATGGCCTGCGCGTGCCTAATTTCCAACCAAGCGATTATCACATCTATTGCGAG

TATCATGCTGAGATATGGCGTGCAGGACAAATCACCACCAAGGAGCTCGGCACGGTCA
TGCGGTCCCTGGGACAGAACCCGTCCGAGTCTGAGCTGCAAGATATGATTAACGAGGT
CGACGCCGACAACAACGGCACCATTGACTTCCCTGGTATGTCGAGATATTCGCCTG-
TTGGGGGGG
>Diaporthe_chrysalidocarpi_SAUCC194_35
NNNNNNNNTTTTTGTAAGTACCATTTCCAACCAGCGGACACGAGTTCAAGCTCCCCGC
CCT--
CCTCTGCTGTTGCGCATGATGCTAACGGACCGTTTTCGGTTTGTAGGATAAGGATGGCG
ATGGTTAGTGCGGCCGCCCTCGTTCTTCCTTCTCACACACGCACGCGTCATGCTCGATCC
TCCGCGACGGCCTGCGCGTGCCTAATTTCCAACCAAGCGATTATCACATCTATTGCGAG
TACCATGCTGAGATATGGGGTGCAGGACAAATTACCACCAAGGAGCTCGGCACCGTCA
TGCGATCCCTGGGCCAGAACCCTTCCGAGTCTGAGCTGCAAGATATGATTAACGAGGTC
GACGCCGACAACAACGGCACCATTGACTTCCCTGGTACGTCCAGATATACGCCCA-
TTGAAGTGG
>Diaporthe_endocitricola_ZHKUCC20_0012
NNNNNNNNNNNNNNNNNNNNNNNNNNNNNNNNNGCGGACTCGAGTTCAAGCTCC
CCGCCCT--
CCTCTGCTGTTGCGCATGATGCTAACGGACCGTTTTCGGCTTGTAGGATAAGGATGGCG
ATGGTTAGTGCGGCCGCCCTCGTTCTTCCTTCTCACACACGCACGCGTCATGCTCGATCC
TCCGCGACGGCCTGCGCGTGCCTAATTTCCAACCAAGCGATTATCACATCTATTGCGAG
TATCATGCTGAGATATGGCGTGCAGGACAAATCACCACCAAGGAGCTCGGCACGGTCA
TGCGATCCCTGGGCCAGAACCCTTCCGAGTCTGAGCTGCAAGATATGATCAACGAGGTC
GACGCCGACAACAACGGCACCATTGACTTCCCTGGTACGTCCAGATATACGCCCA-
TCGAGGGGG
>Diaporthe_eres_CBS_138594
CTTTTCCCTCTTTGTAAG--TCATTTCCAGCCGGCAGACATG------AGCTCCCCGCCCT--
CCTCTGCTGGTGCGCATGATGCTAACGGACCGTTTTCGGCTTGTAGGATAAGGATGGCG
ATGGTTAGTGCGGCCGC-
CTCTTTGCCCCCTCCCACCTACGCACGCGTCATGTTCGATCCGCCGCGACAGCCTGCGC
GTGCATAATTTCCAACCAAGCGATTATCACATCTATCACGAGTATCATGCTGAGATATG
GCGTGTAGGGCAAATCACCACCAAGGAGCTCGGCACGGTCATGCGATCCCTGGGTCAG
AACCCGTCCGAGTCTGAGCTGCAAGATATGATTAACGAGGTCGACGCCGACAACAATG
GCACCATTGACTTCCCTGGTACGTCCAGATGCTCGCGCT-CTNNNNNNN
>Diaporthe_eugeniae_CBS_444_82
NNNNNNNNNNNNNNNNNNNNNNNNNNNNNACCAGCGGACTCGAGTTCAAGCTCC
CCGCCCT--
CCTCTGCTGTTGCGCATGATGCTAACGGACCGTTTTCGGCTTGTAGGATAAGGATGGCG
ATGGTTAGTGCGGCCGCCCTCGTTCTTCCTTCTCACGCACGCACGCGTCATGCTCGATCC
TCCGCGACGGCCTGCGCGTGCCTAATTTCCAACCAAGCGATTATCACATCTATTGCGAG
TATCATGCTGAGATATGGCGTGCAGGACAAATCACCACCAAGGAGCTCGGCACCGTCA
TGCGATCCCTGGGCCAGAACCCTTCCGAGTCTGAGCTGCAAGATATGATTAACGAGGTC
GACGCCGACAACAACGGCACCATTGACTTCCCTGGTACGTCCAGATATACGCCCA-
TTGAAGGGG

>Diaporthe_fujianensis_JZB320149
NNNNNNNNNNNNNNNNNNNNNNNNNNNNNNNNNNNNNNNNNNNNNNNNNNNNN
NNNNNGCTCT--
CCTTTGCTGTTGCGCATGATGCTAACGGACCGTTTTCGGCTTGTAGGATAAGGATGGCG
ATGGTTAGTGCGGCCGCCCTCTTTCTTTTCTCTCACACACGCACGCGTCACGCTCGATCC
GCCGCGACGGCCTGCGCGTGCATAATTTCCAACCAAGCGATTATCACACATATTGCGAG
TATCATGCTGAGATATGGCGTGTAGGACAAATCACCACCAAGGAGCTCGGCACGGTCA
TGCGATCCCTGGGACAGAACCCGTCCGAGTCTGAGCTGCAAGACATGATTAACGAGGT
CGACGCCGACAACAACGGCACCATTGACTTCCCTGGTACGTCCAGAAGCACGCCCG-
TTGAGAAGG
>Diaporthe_fulvicolor_PSCG_051
NNNNNNNNNNNNNNNNNNNNNNNNNNNNNNNNNNNNNNNNNNNNNNNNNNNAGC
TCCCCGCCCT--
CCTCTGCTGTTGCGCATGATGCTAACGGACCGTTTTCGGCTCGTAGGATAAGGATGGCG
ATGGTTAGTGCGGCCGCCCTCGTTCTTCCTTCTCACACACGCACGCGTCATGCTCGATCC
TCCGCGACGGCCTGCGCGTGCCTAATTTCCCACCAAGCGATTATCACATCTATTGCGAG
TATCATGCTGAGATATGGCGTGCAGGACAAATCACCACCAAGGAGCTCGGCACAGTCA
TGCGTTCCCTGGGCCAGAACCCTTCCGAGTCTGAGCTGCAAGATATGATTAACGAGGTC
GACGCCGACAACAACGGCACCATTGACTTCCCTGGTACGTCCAGATATACGCCCA-
TCGAGGGGG
>Diaporthe_fusiformis_JZB320156
NNNNNNNNNNNNNNNNNNNNNNNNNNNNNNNNNNNNNNNNNNNNNNNNNNNNN
NNNNNGCTCT--
CCTTTGCTGTTGCGCATGATGCTAACGGACCGTTTTCGGCTTGTAGGATAAGGATGGCG
ATGGTTAGTGCGGCCGCCCTCTTTCTTTTCTCTCACACACGCACGCGTCACGCTCGATCC
GCCGCGACGGCCTGCGCGTGCATAATTTCCAACCAAGCGATTATCACACATATTGCGAG
TATCATGCTGAGATATGGCGTGTAGGACAAATCACCACCAAGGAGCTCGGCACGGTCA
TGCGATCCCTGGGACAGAACCCGTCCGAGTCTGAGCTGCAAGACATGATTAACGAGGT
CGACGCCGACAACAACGGCACCATTGACTTCCCTGGTACGTCCAGAAGCACGCCCG-
TTGAGAAGG
>Diaporthe_guangxiensis_JZB320094
NNNNNNNNNNNNNNNNNNNNNNNNNNNNNNNNNNNNNNNNNNNNNNNNNNNNN
NNNCCGCTCT--
CCTCTGCTGCTGCGCATGATGCTAACGGACCGTTTTCGGCTTGCAGGATAAGGATGGCG
ATGGTTAGTGCGGCCGCCCTCGTTCTTCCTTCTCACACACGCACGCGTCATGCTCGATCC
TCCGCGACGGCCTGCGCGTGCCTAACTTCTAACAATGCGATTATCACATCTATTGCGAG
TATCATGCTGAGATATGGCGTGCAGGACAAATCACCACCAAGGAGCTCGGCACCGTCA
TGCGATCCCTGGGCCAGAACCCTTCCGAGTCTGAGCTGCAAGATATGATTAACGAGGTC
GACGCCGACAACAACGGCACCATTGACTTCCCTGGTACGTCCAGATATACGCCCA-
TTGAGGAGG
>Diaporthe_hongkongensis_CBS_115448
CTTCTCCCTCTTTGTAAGTATCATCTCCAGCCAGCGGACACGAGTTCAAGCTCCCCGCCC
T--

CCTCTGCTGTTGCGCATGATGCTAACGGACCGTTTTCGGCTTGTAGGATAAGGATGGCG
ATGGTTAGTGCGGCCGCCCTCTTTCTTCCCTCTCACATACGCACGCGTCATGCTCGATCC
GCCGCGACGGCCTGCGCGTGCATAATTTCCAACCAAGCGATTATCACATATATTGCGAG
TATCATGCTGAGATATGGCGTGTAGGACAAATCACCACCAAGGAGCTCGGCACGGTCA
TGCGATCCCTGGGACAGAACCCGTCCGAGTCTGAGCTGCAAGATATGATTAACGAGGT
CGACGCCGACAACAACGGCACCATTGACTTCCCTGGTACGTCCAAAAGCACGCCCG-
TTGAGGAGG
>Diaporthe_hunanensis_HNZZ023
NNNNNNNNNNNNNNNNNNNNNNNNNNNNNNNNNNNNNNNNNNNNNNNNNNNNNN
NNNNNGCCCT--
CCTCTGCTGTTGCGCATGATGCTAACGGACCGTTTTCGGCTCGTAGGATAAGGATGGCG
ATGGTTAGTGCGGCCGCCCTCGTTCTTCCTTCTCACACACGCACGCGTCATGCTCGATCC
TCCGCGACGGCCTGCGCGTGCCTAATTTCCCACCAAGCGATTATCACATCTATTGCGAG
TATCATGCTGAGATATGGCGTGCAGGACAAATCACCACCAAGGAGCTCGGCACAGTCA
TGCGTTCCCTGGGCCAGAACCCTTCCGAGTCTGAGCTGCAAGATATGATTAACGAGGTC
GACGCCGACAACAACGGCACCATTGACTTCCCTGGTACGTCCAGATATACGCCCA-
TCGAGGGGG
>Diaporthe_limonicola_CBS_142549
NNNNNNNNNNNNNNNNNNNNNNNNNNNNNNNNNNNNNNNNNNNNNNNNNNNNNN
NNNNNNNNNNNNNNNNNNNNNNNNNNNNNNNNNNNNNCTAACGGACCGTTTTCGGCTT
GTAGGATAAGGATGGCGATGGTTAGTGCGGCCGCCCTCGTTCTTCCTTCTCACACACGC
ACGCGTCATGCTCGATCCCCCGCGACGGCCTGCGCGTGCCTAACCTCCAACCAAGCGAT
TATCACATCTATTGCGAGTATCATGCTGAGATATGGCGTGCAGGACAAATCACCACCAA
GGAGCTCGGCACGGTCATGCGATCCCTGGGCCAGAACCCTTCCGAGTCTGAGCTGCAA
GATATGATCAACGAGGTCGACGCCGACAACAACGGCACCATTGACTTCCCTGGTACGT
CCGGATATACGCCCA-TTGAGGGCG
>Diaporthe_melitensis_CBS_142551
NNNNNNNNNNNNNNNNNNNNNNNNNNNNNNNNNNNNNNNNNNNNNNNNNNNNNN
NNNNNNNNNNNNNNNNNNNNNNNNNNNNNNNNNNNNNCTAACGGACCGTTTTCGGCTT
GTAGGATAAGGATGGCGATGGTTAGTGCGGCCGCCCTCGTTCTTCCTTCTCACACACGC
ACGCGTCATGCTCGATCCCCCGCGACGGCCTGCGCGTGCCTAACCTCCAACCAAGCGAT
TATCACATCTATTGCGAGTATCATGCTGAGATATGGCGTGCAGGACAAATCACCACCAA
GGAGCTCGGCACGGTCATGCGATCCCTGGGCCAGAACCCTTCCGAGTCTGAGCTGCAA
GATATGATCAACGAGGTCGACGCCGACAACAACGGCACCATTGACTTCCCTGGTACGT
CCGGATATACGCCCA-TTGAGGGCG
>Diaporthe_millettiae_GUCC9167
NNNNNNNNNNNNNGTTCCGAATCATCTCCACCAGCGGACTCGAGTTCAAGCTCCCCG
CCCT--
CCTCTGCTGTTGCGCATGATGCTAACGGACCGTTTTCGGCTTGTAGGATAAGGATGGCG
ATGGTTAGTGCGGCCGCCCTCGTTCTTCCTTCTCACACACGCACGCGTCATGCTCGATCC
TCCGCGACGGCCTGCGCGTGCCTAATTTCCAACCAAGCGATTATCACATCTATTGCGAG
TATCATGCTGAGATATGGCGTGCAGGACAAATCACCACCAAGGAGCTCGGCACGGTCA
TGCGATCCCTGGGCCAGAACCCTTCCGAGTCTGAGCTGCAAGATATGATCAACGAGGTC

GACGCCGACAACAACGGCACCATTGACTTCCCTGGTACGTCCAGATATACGCCCA-
TCGAGGGGG
>Diaporthe_musigena_CBS_129519
NNNNNNNNNNNNNNNNNNNNNNNNNNNNNNNNNNNNNNNGGCAGACATGAGCT
CCCCGCCCT--
CCTCTGCTGGTGCGCATGATGCTAACGGACCGTTTTCGGCTTGTAGGATAAGGATGGCG
ATGGTTAGTGCGGCCGCCCTCGTTCTTCCTTCTCACACACGCACGCGTCATGCTCGATCC
TCCGCGACGGCCTGCGCGTGCCTAATTTCCAACCAAGCGATTATCACATCTATTGCGAG
TATCATGCTGAGATATGGCGTGCAGGACAAATCACCACCAAGGAGCTCGGCACGGTCA
TGCGATCCCTGGGCCAGAACCCTTCCGAGTCTGAGCTGCAAGATATGATTAACGAGGTC
GACGCCGACAACAACGGCACCATTGACTTCCCTGGTACGTCCAGATGCTCGCGCT-
CTGGGGGGG
>Diaporthe_osmanthi_GUCC9165
NNNNNNNNNNNNNNNNNNGCCGAACGTCTCACCAGCGGACACGAGTTCAAGCTCCCC
GCCCT--
CCTCTGCTGTTGCGCATGATGCTAACGGACCGTTTTCGGCTTGCAGGATAAGGATGGCG
ATGGTTAGTGCGGCCGCCCTCGTTCTTACTTCTCACACACGCACGCGTCATGCTCGATCC
TCCGCGACGGCCTGCGCGTGCCTAATTTCCAACCAAGCGATTATCACATCTATTGCGAG
TATCATGCTGAGATATGGCGTGCAGGACAAATCACCACCAAGGAGCTCGGCACCGTCA
TGCGATCCCTGGGCCAGAACCCTTCCGAGTCTGAGCTGCAAGATATGATTAACGAGGTC
GACGCCGACAACAATGGCACCATTGACTTCCCTGGTACGTCCAGATATACGTCCA-
TCGAGGGAG
>Diaporthe_perseae_CBS_151_73
CTTCTCCCTCTTTGTAAGTACCATCTCCAACCAGCGGACTCGAGTTCAAGCTCCCCGCCC
T--
CCTCTGCTGTTGCGCATGATGCTAACGGACCGTTTTCGGCTTGTAGGATAAGGATGGCG
ATGGTTAGTGCGGCCGCCCTCGTTCTTCCTTCTCACACACGCACGCGTCATGCTCGATCC
TCCGCGACGGCCTGCGCGTGGCTAGTTTCGAACCAAGCGATTATCACATCTATTGCGAG
TATCATGCTGAGATATGGCGTGCAGGACAAATCACCACCAAGGAGCTCGGCACGGTCA
TGCGATCCCTGGGCCAGAACCCTTCCGAGTCTGAGCTGCAAGATATGATTAACGAGGTC
GACGCCGACAACAACGGCACCATTGACTTCCCTGGTACGTCCAGATATACGCACA-
TCGAGGGGG
>Diaporthe_pescicola_MFLUCC_16_0105
CTTCTCCCTCTTTGTAAGTATCATCTCCAGCCAGCGGACATGAG---------CTCCCCGC--
CCTCTGCTGTTGCGCATGATGCTAACGGACCGTTTTCGG-
TTGGAAGATAAGGATGGAGATGGTTAGTGCGGCCGCCCTCTTTCTTCCTTCTCACACAC
GCGCGCGTCCTGCTCCATCCTCCGGGACGGGCTGCGCGTGTCTAATTTCCAACCAAGCG
ATTATCACATCTATTGCGAGTATCATGGTGAGATATGGCGTGCAGGACAAATCACCACC
AAGGAGCTCGGCACCGCCCTGGGATCCATGGGCCACAACCCTTCCGAGTCTGAACTGC
ATAATATGATTAACGACGACGACGCCGACAACAATGGCTCCCTTGACTTCCCTGGAAC
GTCCAGATATACGCCCA-TCGAGGGGG
>Diaporthe_podocarpi-macrophylli_CGMCC3_18281

NNNNNNNNNNNNNNNNNNNNNNNNNNNNNNNNAACGGACACGAGTTCAAGCTC
CCGGCCCT--
CCTCTGCTGTTGCGCATGATGCTAACGGACCGTTTTCGGCTTGTAGGATAAGGATGGCG
ATGGTTAGTGCGGCCGCCCTCGTTCTTCCTTCTCACACACGCACGCGTCATGCTCGATCC
TCCGCGACGGCCTGCGCGTGCCTAATTTCCAACCAAGCGATTATCACATCTATTGCGAG
TATCATGCTGAGATATGGCGTGCAGGACAAATCACCACCAAGGAGCTCGGCACGGTCA
TGCGATCCCTGGGCCAGAACCCTTCCGAGTCTGAGCTGCAAGATATGATTAACGAGGTC
GACGCCGACAACAACGGCACCATTGACTTCCCTGGTACGTCCGGATATACGCCCA-
TTGAGGGGG
>Diaporthe_pseudomangiferae_CBS_101339
NNNNNNNNNNNNNNNNNNNNNNNNCTCCAGCCAGCAGACACGAGTCCAAGCTCCC
CGCCCT--
CCTCTGCTGTTGCGCATGATGCTAACGGACCGTTTTCGGCTTGTAGGATAAGGATGGCG
ATGGTTAGTGCGGCCGCCCTCGTTCTTCCTTCTCACACACGCACGCGTCATGCTCGATCC
TCCGCGACGGCCTGCGCGTGCCTGATTTCCAACAAAGCGATCATCACATCTATTGCGAG
TATCATGCTGAGATATGGCGTGCAGGACAAATCACCACCAAGGAGCTCGGCACCGTCA
TGCGATCCCTGGGCCAGAACCCTTCCGAGTCTGAGCTGCAAGATATGATTAACGAGGTC
GACGCCGACAACAACGGCACCATTGACTTCCCTGGTACGTCCAGATATACGCCCA-
TTGAGGGGG
>Diaporthe_pseudophoenicicola_CBS_462_69
CTTCTCCCTCTTTGTAAGTACCATCTCCAACCAGCGGACTCGAGTTCAAGCTCCCCGCCC
T--
CCTCTGCTGTTGCGCATGATGCTAACGGACCGTTTTCGGCTTGTAGGATAAGGATGGCG
ATGGTTAGTGCGGCCGCCCTCGTTCTTCCTTCTCACACACGCACGCGTCATGCTCGATCC
TCCGCGACGGCCTGCGCGTGCCTAATTTCGAACCAAGCGATTATCACATCTATTGCGAG
TATCATGCTGAGATATGGCGTGCAGGACAAATCACCACCAAGGAGCTCGGCACCGTCA
TGCGATCCCTGGGCCAGAACCCTTCCGAGTCTGAGCTGCAAGATATGATTAACGAGGTC
GACGCCGACAACAACGGCACCATTGACTTCCCTGGTACGTCCAGATATACGCCCA-
TTGAGGGGG
>Diaporthe_pterocarpicola_MFLUCC_10_0580a
NNNNNNNNNNNNNNNNNNNNNNNNNNNNNNNNNNNNNNNNNNNNNNNNNNNNN
NNNNNGCCCT--
CCTCTACTGTTGCGCATGATGCTAACGGACCGTTTTCGGCTTGCAGGATAAGGATGGCG
ATGGTTAGTGCGGCCGCCCTCTTTCTTCCTTCTCACACACGCACGCGTCATGCTCGATTC
TCCGCGACGGCCTGCGCGTGCCTAATTTCCAACCAAGCGATTATCACATCTATTGCGAG
TATCATGCTGAGATATGGTGTGCAGGACAAATCACCACCAAGGAGCTCGGCACCGTCA
TGCGATCCCTGGGCCAGAACCCTTCCGAGTCTGAGCTGCAAGATATGATTAACGAGGTC
GACGCCGACAACAACGGCACCATTGACTTCCCCGGTACGTCCAGACATACGCCCA-
TTGAGGGGG
>Diaporthe_rhodomyrti_CFCC_53101
NNNNNNNNNCTTTGTAAGTATCATCTCCAGCCAGCGGACACGAGTTCAAGCTCCCCGC
TCT--
CCTCTGCTGCTGCGCATGATGCTAACGGACCGTTTTCGGCTTGTAGGATAAGGATGGCG

ATGGTTAGTGCGGCCGCCCTCGTTCTTCCTTCTCACACACGCACGCGTCATGCTCGATCC
TCCGCGACGGCCTGCGCGTGCCTAACTTCTAACAATGCGATTATCACATCTATTGCGAG
TATCATGCTGAGATATGGCGTGCAGGACAAATCACCACCAAGGAGCTCGGCACCGTCA
TGCGATCCCTGGGCCAGAACCCTTCCGAGTCTGAGCTGCAAGATATGATTAACGAGGTC
GACGCCGACAACAACGGCACCATTGACTTCCCTGGTACGTCCAGATATACGCCCA-
TTGAGGAGG
>Diaporthe_schimae_CFCC_53103
NNNNNNNNNCTTTGTAAGTATCATCTCCAGCCAGCGGACACGAGTTCAAGCTCCCCGC
TCT--
CCTTTGCTGTTGCGCATGATGCTAACGGACCGTTTTCGGCTTGTAGGATAAGGATGGCG
ATGGTTAGTGCGGCCGCCCTCTTTCTTCTCTCTCACACACGCACGCGTCACGCTCGATCC
GCCGCGACGGCCTGCGCGTGCATAATTTCCAACCAAGCGATTATCACACATATTGCGAG
TATCATGCTGAGATATGGCGTGTAGGACAAATCACCACCAAGGAGCTCGGCACGGTCA
TGCGATCCCTGGGACAGAACCCGTCCGAGTCTGAGCTGCAAGACATGATTAACGAGGT
CGACGCCGACAACAACGGCACCATTGACTTCCCTGGTACGTCCAGAAGCACGCCCG-
TTGAGAAGG
>Diaporthe_sennae_CFCC_51636
NNNNNNNNNNTTTGTAAGTACCATATCCAACCAGCGGACTCGAGTTCAAGCTCCCCG
CCCT--
CCTTTGCTGTTGCGCATGATGCTAACGGACCGTTTTTGGCTTGTAGGATAAGGATGGCGA
TGGTTAGTGCGGCCGCCCTCGTTCTTCCTTCTCACACACGCACGCGTCATGCTCGATCCT
CCGCGACGGCCTGCGCGTGCCTAATTTCCAACCAAGCGATTATCACATCTATTGCGAGT
ATCATGCTGAGATATGGCGTGCAGGACAAATCACCACCAAGGAGCTCGGCACGGTCAT
GCGATCCCTGGGCCAGAACCCTTCCGAGTCTGAGCTGCAAGATATGATCAACGAGGTC
GACGCCGACAACAACGGCACCATTGACTTCCCTGGTACGTCCAGATATACGCCCA-
TCGAGGGGG
> Diaporthe_africana_STMA18294

CTTCTCCCTCTTTGTAAGTATCATCTCCAGCCAGCGGACACGAGTTCAAGCTCCCCGCCC
T--
CCTCTGCTGTTGCGCATGATGCTAACGGACCGTTTTCGGCTTGTAGGATAAGGATGGCG
ATGGTTAGTGCGGCCGCCCTCTTTCTTCCCTCTCACACACGCACGCGTCACGCTCGATCC
GCCGCGACGGCCTGCGCGTGCATAATTTCCAACCAAGCGATTATCACATATATTGCGAG
TATCATGCTGAGATATGGCGTGTAGGACAAATCACCACCAAGGAGCTCGGCACGGTCA
TGCGATCCCTGGGACAGAACCCGTCCGAGTCTGAGCTGCAAGATATGATTAACGAGGT
CGACGCCGACAACAACGGCACCATTGACTTCCCTGGTACGTCCAAAAGCACGCCCG-
TTGAGGAGG
>Diaporthe_africana_CBS_150080
CTTCTCCCTCTTTGTAAGTATCATCTCCAGCCAGCGGACACGAGTTCAAGCTCCCCGCCC
T--
CCTCTGCTGTTGCGCATGATGCTAACGGACCGTTTTCGGCTTGTAGGATAAGGATGGCG
ATGGTTAGTGCGGCCGCCCTCTTTCTTCCCTCTCACACACGCACGCGTCACGCTCGATCC
GCCGCGACGGCCTGCGCGTGCATAATTTCCAACCAAGCGATTATCACATATATTGCGAG
TATCATGCTGAGATATGGCGTGTAGGACAAATCACCACCAAGGAGCTCGGCACGGTCA

TGCGATCCCTGGGACAGAACCCGTCCGAGTCTGAGCTGCAAGATATGATTAACGAGGT
CGACGCCGACAACAACGGCACCATTGACTTCCCTGGTACGTCCAAAAGCACGCCCG-
TTGAGGAGG
>Diaporthe_spinosa_PSCG_383
NNNNNNNNNNNNNNNNNNNNNNNNNNNNNNNNNNNNNNNNNNNNNNNNNAGC
TCCCCGCCCT--
CCTCTGCTGTTGCGCATGATGCTAACGGACCGTTTTCGGTTTGTAGGATAAGGATGGCG
ATGGTTAGTGCGGCCGCCCTCGTTCTTCCTTCTCACACACGCACGCGTCATGCTCGATCC
TCCGCGACGGCCTGCGCGTGCCTAATTTCCAACCAAGCGATTATCACATCTATTGCGAG
TACCATGCTGAGATATGGCGTGCAGGACAAATCACCACCAAGGAGCTCGGCACCGTCA
TGCGATCCCTGGGCCAGAACCCTTCCGAGTCTGAGCTGCAAGATATGATTAACGAGGTC
GACGCCGACAACAACGGCACCATTGACTTCCCTGGTACGTCCAGATATACGCCCA-
TTGAAGTGG
>Diaporthe_tectonigena_MFLUCC_12_0767
NNNNNNNNNNNNNNNNNNNNNNNNNCTCAGCCAGCGGGCATGAGTTCAAGCTCCC
TGCCCC--
CCTCTGCCGTTGCGCATGATGCTAACGGACCGTTTTCGGCTTGTAGGATAAGGATGGCG
ATGGTTAGTGCGGCCGCCCTCTTTCTTCCCTCTCACACACGCACGCGTCACGCTCGATCC
GCCGCGACGGCCTGCGCGTGCCTAACTTCCAACCAAGCGATTATCATATCTATTGCGAG
TATCATGCTGAGATATGGCGTGTAGGACAAATCACCACCAAGGAGCTCGGCACGGTCA
TGCGATCCCTGGGACAGAACCCGTCCGAGTCTGAGCTGCAAGATATGATTAACGAGGT
CGACGCTGACAACAACGGCACCATTGACTTCCCTGGTACGTCCAGATATACGCCCG-
TTGAGGGGG
>Diaporthe_viniferae_JZB320071
NNNNNNNNNNNNNNNNNNNNNNNNNNNNNNNNNNNNNNNGGCACGAGTTCAAGCTC
CCCGCTCT--
CCTCTGCTGCTGCGCATGATGCTAACGGACCGTTTTCGGCTTGCAGGATAAGGATGGCG
ATGGTTAGTGCGGCCGCCCTCGTTCTTCCTTCTCACACACGCACGCGTCATGCTCGATCC
TCCGCGACGGCCTGCGCGTGCCTAACTTCTAACAATGCGATTATCACATCTATTGCGAG
TATCATGCTGAGATATGGCGTGCAGGACAAATCACCACCAAGGAGCTCGGCACCGTCA
TGCGATCCCTGGGCCAGAACCCTTCCGAGTCTGAGCTGCAAGATATGATTAACGAGGTC
GACGCCGACAACAACGGCACCATTGACTTCCCTGGTACGTCCAGATATACGCCCA-
TTGAGGAGG

- Alignment of the *his3* sequences used in the phylogenetic study

>Diaporthe_acuta_PSCG_047
TCCGCGCCCTCCACCGGAGGTGTCAAGAAGCCTCACCGCTACAAGCCTGGTACCGTCGC
TCTGCGTGAGATCCGTCGCTACCAGAAGAGCACTGAGCTGCTGATCCGCAAGCTCCCCT
TCCAGCGTCTGGTATGTCGCCGCCCCCGTCTTCTGAATGCCCACCCGCGTACCTTTGCTG
ACCATCGCCTTCCACTTCCAGGTCCGTGAGATCGCCCAGGACTTCAAGTCCGACCTCCG
CTTCCAGTCCTCCGCCATCGGTGCCCTGCAGGAGTCCGTCGAGTCTTACCTCGTCTCCCT
CTTCGAGGACACCAACCTGTGCGCCATCCACGCCAAGCGTGTCACCATCCAGTCGGTAC
GTA

>Diaporthe_amygdali_CBS_126679
TCCGCGCCCTCCACCGGAGGTGTCAAGAAGCCTCACCGCTACAAGCCTGGTACCGTCGC
TCTGCGTGAGATTCGTCGCTACCAGAAGTCCACTGAGCTTCTGATCCGCAAGCTGCCCTT
CCAGCGTCTGGTACGC--
AGGTTCGTGAGATTGCCCAGGACTTCAAGTCCGACCTCCGCTTCCAGTCCTCCGCCATC
GGTGCCCTGCAGGAGTCCGTCGAGTCCTACCTCGTCTCCCTCTTCGAGGACACCAACCT
GTGCGCCATCCACGCCAAGCGTGTCACCATCCAGTCGGTATGTA
>Diaporthe_anhuiensis_CNUCC_201902
TCCGCGCCCTCCACCGGAGGTGTCAAGAAGCCTCACCGCTACAAGCCTGGTACCGTCGC
TCTGCGTGAGATCCGTCGCTACCAGAAGAGCACTGAGCTGCTGATCCGCAAGCTCCCCT
TCCAGCGTCTGGTATGTCGCCGCCCCCGTCTTCTGAATGCCCACCCGCGCACCTTTGCTG
ACCATCGCCTTCCACTTCCAGGTCCGTGAGATCGCCCAGGACTTCAAGTCCGACCTCCG
CTTCCAGTCTTCCGCCATCGGTGCCCTGCAGGAGTCCGTCGAGTCTTACCTCGTCTCCCT
CTTCGAGGACACCAACCTGTGCGCCATCCACGCCAAGCGTGTCACCATCCAGTCGGTAC
GTA
>Diaporthe_arecae_CBS_161_64
TCCGCGCCCTCCACCGGAGGTGTCAAGAAGCCTCACCGCTACAAGCCTGGTACCGTCGC
TCTGCGTGAGATCCGTCGCTACCAGAAGAGCACTGAGCTGCTGATCCGCAAGCTGCCCT
TCCAGCGTCTGGTATGC--
AGGTCCGTGAGATCGCCCAGGACTTCAAGTCCGACCTCCGCTTCCAGTCTTCCGCCATC
GGTGCCCTGCAGGAGTCCGTCGAGTCTTACCTCGTCTCCCTCTTCGAGGACACCAACCT
GTGCGCCATCCACGCCAAGCGTGTCACCATCCAGTCGGTACGTA
>Diaporthe_arengae_CBS_114979
TCCGCGCCCTCCACCGGAGGTGTCAAGAAGCCTCACCGCTACAAGCCTGGTACCGTCGC
TCTGCGTGAGATCCGTCGCTACCAGAAGAGCACTGAGCTGCTGATCCGCAAGCTCCCCT
TCCAGCGTCTGGTATGC--
AGGTCCGTGAGATCGCCCAGGACTTCAAGTCCGACCTCCGCTTCCAGTCCTCCGCCATC
GGTGCCCTGCAGGAGTCCGTCGAGTCTTACCTCGTCTCCCTCTTCGAGGACACCAACCT
GTGCGCCATCCACGCCAAGCGTGTCACCATCCAGTCGGTACGTA
>Diaporthe_camelliaeoleiferae_HNZZ027
TCCGCGCCCTCCACCGGAGGTGTCAAGAAGCCTCACCGCTACAAGCCTGGTACCGTCGC
TCTGCGTGAGATCCGTCGCTACCAGAAGAGCACTGAGCTGCTGATCCGCAAGCTCCCCT
TCCAGCGTCTGGTATGTCGCCGCCCCCGTCTTCTGAATGCCCACCCGCGTACCTTTGCTG
ACCATCGCCTTCCACTTCCAGGTCCGTGAGATCGCCCAGGACTTCAAGTCCGACCTCCG
CTTCCAGTCCTCCGCCATCGGTGCCCTGCAGGAGTCCGTCGAGTCTTACCTCGTCTCCCT
CTTCGAGGACACCAACCTGTGCGCCATCCACGCCAAGCGTGTCACCATCCAGTCGGTAC
GTA
>Diaporthe_cercidis_CFCC_52565
TCCGCGCCCTCCACCGGAGGTGTCAAGAAGCCTCACCGCTACAAGCCTGGTACCGTCGC
TCTGCGTGAGATCCGTCGCTACCAGAAGAGCACTGAGCTGCTGATCCGCAAGCTCCCCT
TCCAGCGTCTGGTATGTCGCCCACCCCGTCTTCTGAATGCCCACCCGCGTACCTTTGCTG
ACCATCGCCTTCCACTTCCAGGTCCGTGAGATCGCCCAGGACTTCAAGTCCGACCTCCG
CTTCCAGTCCTCCGCCATCGGTGCCCTGCAGGAGTCCGTCGAGTCTTACCTCGTCTCCCT

CTTCGAGGACACCAACCTGTGCGCCATCCACGCCAAGCGTGTCACCATCCAGTCGGTAC
GTA
>Diaporthe_cf_heveae_2_CBS_681_84
TCCGCGCCCTCCACCGGAGGTGTCAAGAAGCCTCACCGCTACAAGCCTGGTACCGTCGC
TCTGCGTGAGATCCGTCGCTACCAGAAGAGCACCGAGCTGCTGATCCGCAAGCTCCCCT
TCCAGCGTCTGGTATGC---
AGGTTCGTGAGATCGCCCAGGACTTCAAGTCCGACCTCCGCTTCCAGTCCTCCGCCATC
GGTGCCCTGCAGGAGTCTGTCGAGTCTTACCTCGTCTCCCTCTTCGAGGACACCAACCTG
TGCGCCATCCACGCCAAGCGTGTCACCATCCAGTCGGTACGTT
>Diaporthe_chrysalidocarpi_SAUCC194_35
TCCGCGCCCTCCACCGGAGGTGTCAAGAAGCCTCACCGCTACAAGCCTGGTACCGTCGC
TCTGCGTGAGATCCGTCGCTACCAGAAGAGCACTGAGCTGCTGATCCGCAAGCTCCCCT
TCCAGCGTCTGGTATGTCGCCGCCCCGTCTTCTGAATGCCCACCCGCACACCTTTGCTG
ACCATTGCCTTCCACTTCCAGGTCCGTGAGATCGCCCAGGACTTCAAGTCCGACCTCCG
CTTCCAGTCCTCCGCCATCGGTGCCCTGCAGGAGTCCGTCGAGTCTTACCTCGTCTCCCT
CTTTGAGGACACCAACCTGTGCGCCATCCACGCCAAGCGTGTCACCATCCAGTCGGTAC
GTA
>Diaporthe_eres_CBS_138594
TCCGCGCCCTCCACCGGAGGTGTCAAGAAGCCTCACCGCTACAAGCCTGGTACCGTCGC
TCTGCGTGAGATCCGTCGCTACCAGAAGAGCACCGAGCTGCTGATCCGCAAGCTCCCCT
TCCAGCGTCTGGTATGT---
AGGTCCGTGAGATCGCCCAGGACTTCAAGTCCGACCTCCGCTTCCAGTCTTCCGCCATC
GGTGCCCTGCAGGAGTCGGTTGAGTCTTACCTCGTCTCCCTCTTCGAGGACACCAACCT
GTGCGCCATCCACGCCAAGCGTGTCACCATCCAGTCGGTACGTT
>Diaporthe_eugeniae_CBS_444_82
TCCGCGCCCTCCACCGGAGGTGTCAAGAAGCCTCACCGCTACAAGCCTGGTACCGTCGC
TCTGCGTGAGATCCGTCGCTACCAGAAGAGCACTGAGCTGCTGATCCGCAAGCTGCCCT
TCCAGCGTCTGGTATGC---
AGGTCCGTGAGATCGCCCAGGACTTCAAGTCCGACCTCCGCTTCCAGTCTTCCGCCATC
GGTGCCCTGCAGGAGTCCGTCGAGTCTTACCTCGTCTCCCTCTTCGAGGACACCAACCT
GTGCGCCATCCACGCCAAGCGTGTCACCATCCAGTCGGTACGTA
>Diaporthe_fulvicolor_PSCG_051
TCCGCGCCCTCCACCGGAGGTGTCAAGAAGCCTCACCGCTACAAGCCTGGTACCGTCGC
TCTGCGTGAGATCCGTCGCTACCAGAAGAGCACTGAGCTGCTGATCCGCAAGCTCCCCT
TCCAGCGTCTGGTATGTCGCCGCCCCGTCTTCTGAATGCCCACCCGCGTACCTTTGCTG
ACCATCGCCTTCCACTTGCAGGTCCGTGAGATCGCCCAGGACTTCAAGTCCGACCTCCG
CTTCCAGTCCTCCGCCATCGGTGCCCTGCAGGAGTCCGTCGAGTCTTACCTCGTCTCCCT
CTTCGAGGACACCAACCTGTGCGCCATCCACGCCAAGCGTGTCACCATCCAGTCGGTAC
GTA
>Diaporthe_hongkongensis_CBS_115448
TCCGCGCCCTCCACCGGAGGTGTCAAGAAGCCTCACCGCTACAAGCCTGGTACCGTCGC
TCTGCGTGAGATCCGTCGCTACCAGAAGAGCACTGAGCTGCTGATCCGCAAGCTCCCCT
TCCAGCGTCTGGTATGC---

AGGTCCGTGAGATCGCCCAGGACTTCAAGTCCGACCTCCGCTTCCAGTCTTCCGCCATC
GGTGCCCTGCAGGAGTCCGTCGAGTCTTACCTCGTCTCCCTCTTCGAGGACACCAACCT
GTGCGCCATCCACGCCAAGCGTGTCACCATCCAGTCGGTATGTA
>Diaporthe_huangshanensis_CNUCC_201903
TCCGCGCCCTCCACCGGAGGTGTCAAGAAGCCTCACCGCTACAAGCCTGGTACCGTCGC
TCTGCGTGAGATCCGTCGCTACCAGAAGAGCACTGAGCTGCTGATCCGCAAGCTCCCCT
TCCAGCGTCTGGTATGTCGCCGCCCCCGTCTTCTGAATGCCCACCCGCGCAATCTTGCTG
ACCATCGCCTTCCACTTCCAGGTCCGTGAGATCGCCCAGGACTTCAAGTCCGACCTCCG
CTTCCAGTCCTCCGCCATCGGTGCCCTGCAGGAGTCCGTCGAGTCTTACCTCGTCTCCCT
CTTCGAGGACACCAACCTGTGCGCCATCCACGCCAAGCGTGTCACCATCCAGTCGGTAC
GTA
>Diaporthe_hunanensis_HNZZ023
TCCGCGCCCTCCACCGGAGGTGTCAAGAAGCCTCACCGCTACAAGCCTGGTACCGTCGC
TCTGCGTGAGATCCGTCGCTACCAGAAGAGCACTGAGCTGCTGATCCGCAAGCTCCCCT
TCCAGCGTCTGGTATGTCGCCGCCCCCGTCTTCTGAATGCCCACCCGCGTACCTTTGCTG
ACCATCGCCTTCCACTTTCAGGTCCGTGAGATCGCCCAGGACTTCAAGTCCGACCTCCG
CTTCCAGTCCTCCGCCATCGGTGCCCTGCAGGAGTCCGTCGAGTCTTACCTCGTCTCCCT
CTTCGAGGACACCAACCTGTGCGCCATCCACGCCAAGCGTGTCACCATCCAGTCGGTAC
GTA
>Diaporthe_limonicola_CBS_142549
TCCGCGCCCTCCACCGGAGGTGTCAAGAAGCCTCACCGCTACAAGCCTGGTACCGTCGC
TCTGCGTGAGATCCGTCGCTACCAGAAGAGCACTGAGCTGCTGATCCGCAAGCTCCCCT
TCCAGCGTCTGGTATGC--
AGGTCCGTGAGATCGCCCAGGACTTCAAGTCCGATCTCCGCTTCCAGTCTTCCGCCATC
GGTGCCCTGCAGGAGTCCGTCGAGTCTTACCTCGTCTCCCTCTTCGAGGACACCAACCT
GTGCGCCATCCACGCCAAGCGTGTCACCATCCAGTCGGTACGTA
>Diaporthe_meliae_CFCC_53089
TCCGCGCCCTCCACCGGGGGTGTCAAGAAGCCTCACCGCTACAAGCCTGGTACCGTCGC
TCTGCGTGAGATCCGTCGCTACCAGAAGAGCACTGAGCTGCTGATCCGCAAGCTCCCCT
TCCAGCGTCTGGTATGTCGCCGCCCCCGTCTTCTGAATGCCCACCCGCGCAACCTTGCTG
ACCATCGCCTTCCACTACCAGGTCCGTGAGATCGCCCAGGACTTCAAGTCCGACCTCCG
CTTCCAGTCTTCCGCCATCGGTGCCCTGCAGGAGTCCGTCGAGTCTTACCTCGTCTCCCT
CTTCGAGGACACCAACCTGTGCGCCATCCACGCCAAGCGTGTCACCATCCAGTCGGTAC
GTA
>Diaporthe_melitensis_CBS_142551
TCCGCGCCCTCCACCGGAGGTGTCAAGAAGCCTCACCGCTACAAGCCTGGTACCGTCGC
TCTGCGTGAGATCCGTCGCTACCAGAAGAGCACTGAGCTGCTGATCCGCAAGCTCCCCT
TCCAGCGTCTGGTATGC--
AGGTCCGTGAGATCGCCCAGGACTTCAAGTCCGATCTCCGCTTCCAGTCTTCCGCCATC
GGTGCCCTGCAGGAGTCCGTCGAGTCTTACCTCGTCTCCCTCTTCGAGGACACCAACCT
GTGCGCCATCCACGCCAAGCGTGTCACCATCCAGTCGGTACGTA
>Diaporthe_musigena_CBS_129519

TCCGCGCCCTCCACCGGAGGTGTCAAGAAGCCTCACCGCTACAAGCCTGGTACCGTCGC
TCTGCGTGAGATCCGTCGCTACCAGAAGAGCACTGAGCTGCTGATCCGCAAGCTCCCCT
TCCAGCGTCTGGTATGC--
AGGTCCGTGAGATCGCCCAGGACTTCAAGTCCGACCTCCGCTTCCAGTCCTCCGCCATC
GGTGCCCTGCAGGAGTCCGTCGAGTCTTACCTCGTCTCCCTCTTCGAGGACACCAACCT
GTGCGCCATCCACGCCAAGCGTGTCACCATCCAGTCGGTACGTA
>Diaporthe_perseae_CBS_151_73
TCCGCGCCCTCCACCGGAGGTGTCAAGAAGCCTCACCGCTACAAGCCTGGTACCGTCGC
TCTGCGTGAGATCCGTCGCTACCAGAAGAGCACTGAGCTGCTGATCCGCAAGCTGCCCT
TCCAGCGTCTGGTATGC--
AGGTCCGTGAGATCGCCCAGGACTTCAAGTCCGACCTCCGCTTCCAGTCTTCCGCCATC
GGTGCCCTGCAGGAGTCCGTCGAGTCTTACCTCGTCTCCCTCTTCGAGGACACCAACCT
GTGCGCCATCCACGCCAAGCGTGTCACCATCCAGTCGGTACGTA
>Diaporthe_podocarpi-macrophylli_CGMCC3_18281
TCCGCGCCCTCCACCGGAGGTGTCAAGAAGCCTCACCGCTACAAGCCTGGTACCGTCGC
TCTGCGTGAGATCCGTCGCTACCAGAAGAGCACCGAGCTGCTGATCCGCAAGCTCCCCT
TCCAGCGTCTGGTATGC--
AGGTCCGTGAGATCGCCCAGGACTTCAAGTCCGACCTCCGCTTCCAGTCCTCCGCCATC
GGTGCCCTGCAGGAGTCCGTCGAGTCCTACCTCGTCTCCCTCTTCGAGGACACCAACCT
GTGCGCCATCCACGCCAAGCGTGTCACCATCCAGTCGGTATGTA
>Diaporthe_pseudomangiferae_CBS_101339
TCCGCGCCCTCCACCGGAGGTGTCAAGAAGCCTCACCGCTACAAGCCTGGTACCGTCGC
TCTGCGTGAGATCCGTCGCTACCAGAAGAGCACTGAGCTGCTGATCCGCAAGCTGCCCT
TCCAGCGTCTGGTATGC--
AGGTCCGTGAGATCGCCCAGGACTTCAAGTCCGACCTCCGCTTCCAGTCTTCCGCCATC
GGTGCCCTGCAGGAGTCCGTCGAGTCTTACCTCGTCTCCCTCTTCGAGGACACCAACCT
GTGCGCCATCCACGCCAAGCGTGTCACCATCCAGTCGGTACGTA
>Diaporthe_pseudophoenicicola_CBS_462_69
TCCGCGCCCTCCACCGGAGGTGTCAAGAAGCCTCACCGCTACAAGCCTGGTACCGTCGC
TCTGCGTGAGATCCGTCGCTACCAGAAGAGCACTGAGCTGCTGATCCGCAAGCTCCCCT
TCCAGCGTCTGGTATGC--
AGGTCCGTGAGATCGCCCAGGACTTCAAGTCCGACCTCCGCTTCCAGTCTTCCGCCATC
GGTGCCCTGCAGGAGTCCGTCGAGTCTTACCTCGTCTCCCTCTTCGAGGACACCAACCT
GTGCGCCATCCACGCCAAGCGTGTCACCATCCAGTCGGTACGTA
>Diaporthe_rhodomyrti_CFCC_53101
TCCGCGCCCTCCACCGGAGGTGTCAAGAAGCCTCACCGCTACAAGCCTGGTACCGTCGC
TCTGCGTGAGATCCGTCGCTACCAGAAGAGCACTGAGCTGCTGATCCGCAAGCTCCCCT
TCCAGCGTCTGGTATGTCGCCGCACCCATCCTCTGAATCCTCACCC--
GCACCCTTACTGACCATCGCCTTTCACCACCAGGTCCGTGAGATCGCCCAGGACTTCAA
GTCCGACCTCCGCTTCCAGTCTTCCGCCATCGGTGCCCTGCAGGAGTCCGTCGAGTCTTA
CCTCGTCTCCCTCTTTGAGGACACCAACCTGTGCGCCATCCACGCCAAGCGTGTCACCA
TCCAGTCGGTATGTA
>Diaporthe_schimae_CFCC_53103

TCCGCGCCCTCCACCGGAGGTGTCAAGAAGCCTCACCGCTACAAGCCTGGTACCGTCGC
TCTGCGTGAGATCCGTCGCTACCAGAAGAGCACTGAGCTGCTGATCCGCAAGCTCCCCT
TCCAGCGTCTGGTATGTCGCCGCCCCGTCTTCTGAATGCCCACCCGCGTACCTTTGCTG
ACCATCGCCTTCCACTTTCAGGTCCGTGAGATCGCCCAGGACTTCAAGTCCGACCTCCG
CTTCCAGTCCTCCGCCATCGGTGCCCTGCAGGAGTCCGTCGAGTCTTACCTCGTCTCCCT
CTTCGAGGACACCAACCTGTGCGCCATCCACGCCAAGCGTGTCACCATCCAGTCGGTAC
GTA
> Diaporthe_africana_STMA18294

TCCGCGCCCTCCACCGGAGGTGTCAAGAAGCCTCACCGCTACAAGCCTGGTACCGTCGC
TCTGCGTGAGATCCGTCGCTACCAGAAGAGCACTGAGCTGCTGATCCGCAAGCTCCCCT
TCCAGCGTCTGGTATGTCGCCGCCCCTATCCTCTGAATGCTCACCC--
GCACCCTTGCTGACCATCGCCTTTCACCACCAGGTTCGTGAGATCGCCCAGGACTTCAA
GTCCGACCTCCGCTTCCAGTCTTCCGCCATCGGTGCCCTGCAGGAGTCTGTCGAGTCTTA
CCTCGTCTCCCTCTTCGAGGACACCAACCTGTGCGCCATCCACGCCAAGCGTGTCACCA
TCCAGTCGGTATGTA
>Diaporthe_africana_CBS_150080
TCCGCGCCCTCCACCGGAGGTGTCAAGAAGCCTCACCGCTACAAGCCTGGTACCGTCGC
TCTGCGTGAGATCCGTCGCTACCAGAAGAGCACTGAGCTGCTGATCCGCAAGCTCCCCT
TCCAGCGTCTGGTATGTCGCCGCCCCTATCCTCTGAATGCTCACCC--
GCACCCTTGCTGACCATCGCCTTTCACCACCAGGTTCGTGAGATCGCCCAGGACTTCAA
GTCCGACCTCCGCTTCCAGTCTTCCGCCATCGGTGCCCTGCAGGAGTCTGTCGAGTCTTA
CCTCGTCTCCCTCTTCGAGGACACCAACCTGTGCGCCATCCACGCCAAGCGTGTCACCA
TCCAGTCGGTATGTA
>Diaporthe_spinosa_PSCG_383
TCCGCGCCCTCCACCGGAGGTGTCAAGAAGCCTCACCGCTACAAGCCTGGTACCGTCGC
TCTGCGTGAGATCCGTCGCTACCAGAAGAGCACTGAGCTGCTGATCCGCAAGCTCCCCT
TCCAGCGTCTGGTATGTCGCCCACCCCGTCTTCTGAATGCCCACCCGCGTACCTTTGCTG
ACCATCGCCTTCCACTTCCAGGTCCGTGAGATCGCCCAGGACTTCAAGTCCGACCTCCG
CTTCCAGTCCTCCGCCATCGGTGCCCTGCAGGAGTCCGTCGAGTCTTACCTCGTCTCCCT
CTTCGAGGACACCAACCTGTGCGCCATCCACGCCAAGCGTGTCACCATCCAGTCGGTAC
GTA
>Diaporthe_xishuangbanica_CGMCC_3_18282
TCCGCGCCCTCCACCGGAGGTGTCAAGAAGCCTCACCGCTACAAGCCTGGTACCGTCGC
TCTGCGTGAGATCCGTCGCTACCAGAAGAGCACCGAGCTGCTGATCCGCAAGCTCCCCT
TCCAGCGTCTGGTATGC--
AGGTCCGTGAGATCGCCCAGGACTTCAAGTCCGACCTCCGCTTCCAGTCTTCCGCCATC
GGTGCCCTGCAGGAGTCCGTCGAGTCTTACCTCGTCTCCCTCTTCGAGGACACCAACCT
GTGCGCCATCCACGCCAAGCGTGTCACCATCCAGTCGGTATGTA

- Alignment of the *tef1* sequences used in the phylogenetic study

>Diaporthe_amygdali_CBS_126679
GAGAAGGAAGGTTAGTAAACATCAACCTCTACAATCGCAAC-ACATTCTGCATGCC-
CCTTCACATGCTCGACTTCATAAATCCTGGGCGTGCGCCCGTTCCCACAGCGTCATCAC

ACCTGGGGGCGCATTTTCACCCCTCGCTCTGGATTTTCCATTTTCAGTGCGGGTGCGGGG
TGCGCTTATCAGC----GAGCTTATCT-CCCAC-
CCGAAAACCCTGCACACACTACATCACTACATA---------------------------------------CCACC------
----
TTGGATCATGCTGACTTCCCATCTTCACAGCCGCTGAGCTCGGTAAGGG
>Diaporthe_eres_CBS_138594
GAGAAGGAAGGTTAGTAAATAT-------CACAGTCACGGA-ACATGCTACCTGGC-
CCTCCATACTGC---ACCTC--
AATCATCAGCCCGCAGCTGCTCGCGCGGCCTCGCCATGTCGGGGGGCGCATTTTCACCC
CTCGCTTTGGATTTTCAATTTTCAGTGCGAGTGCGGGGTGCGCTTATCAGGGGGCGGGCT
TATCT-CCTACAACCAAAACCCTG------TTACATCA-----------CTCACT---------CAATCCTTGT-
----CACCACCACC--
GAATATTATGCTGACCCTCTATCTACACAGCCGCCGAGCTTGGTAAGGG
>Diaporthe_acuta_PSCG_047
NNNNNNNNNNNNNNNNNNNNNNNNNNNNNNNNNNNNNNNNNNNNNNNNNNNTCCT
CTGC-CCTCAGCCTTTT---GCCTC-
AAATCATCAGTGTGCGGCCGTTGGTACGGCTTCGTCATACTGAGGGGCGCATTTTCACC
CCTCGCTCTGGATTTTCCATTTTCAGTGCGGGTGCGGGGTGCGCTTATCAGG----
GAGCTTATCT-CCTACACCCAAAACCCTG------TTGCAACA--ACACA----CCCACC--
CTACCGCTACTTCATTC--ATCCTCAACCCCC--------------------
ATCGACATCCTTGAATCAATCAAGCAAACCGCCAGGAAATCATGCTGACTTTCTATCTA
CATAGCCGCTGAGCTTGGTAAGGG
>Diaporthe_fraxini-angustifoliae_BRIP_54781
NNNNNNNNNNNNNNNNNNNNNNNNNNNNNNNNNNNNNNNNNNNNNNNNNGCTCTC
CTGC-CCTCAGCCTTTT---GCCTC-
AAATCATCAGTGTGCTGCTGTTGGTACGGCTTCGTCATACTGAGGGGCGCATTTTCACCC
CTCGCTCTGGATTTTCCATTTTCAGTGCGGGTGCGGGGTGCGCTTATCAGG----
GAGCTTATCT-CCTACACCCAAAACCCTG------TTGCAACA--ACACA----CCCACC-----------
TTCCTTGC--AGTTACTACCACC--
GGAAATCATGCTGACTTTCTATCTACATAGCCGCCGAGCTTGGCAAGGG
>Diaporthe_bounty_BRIP_59361a
NNNNNNNNNNNNNNNNNNNNNNNNNNNNNNNNNNNNNNNNNNNNNNNNNNNNNNNN
NNNNNNNNNNNNNNNNNNNNNNNNNNNNNNNNNNATCAGTGTGCGGCTGTTGGTAT
GGCTTCGTCATACTGAGGGGCGCATTTTCACCCCTCGCTCTGGATTTTCCATTTTCAGTGC
GGGTGCGGGGTGCGCTTATCAGG----GAGCTTATCT-CCTACATCCAAAACCCTG------
CTGCAACA----ACG----CCCACC-----------TTCCTTAC--
CGCTATTACCACCACTTCATTCACCCTCAAACCCCATCCACATCTTTGAATCGACCAAG
CAAATCACCAGGAAATCATGCTGACTTTTATCTATACAGCCGCCGAGCTTGGCAAGGG
>Diaporthe_chiangmaiensis_NI207
NNNNNNNNNNNNNNNNNNNNNNNNNNNNNNNNNNNNNNNNNNNNNNNNNNNNNNNN
NCTGC-CCTCAGCATTTT---GCCTC-
ATATCATCAGTGTGCGGCTGTTGGTATGGCTCCGTCATACTGAGGGGCGCATTTTCACCC
CTCGCTCTGGATTTTCCATTTTCAGTGCGGGTGCGGGGTGCGCTTATCAGG----

GAGCTTATCT-CCTACACCCAAAACCCTG------TTGCAACA----ACA----CCCACC-----------
TTTCTTGC--
TGCTATTACCACCACCTCATTCACCCTCAACCCCCATCGACATCTTTGAATCGATCAAGC
AAACCGCCAGGAAATAATGCTGACTTTCTATCTACACAGCCGCCGAGCTTGGTAANNN
>Diaporthe_fulvicolor_PSCG_051
NNNNNNNNNNNNNNNNNNNNNNNNNNNNNNNNNNNNNNNNNNNNNNNNNNNNTCCT
CTGC-CCTCAGCCTTTT---GCCTC-
AAATCATCAGTGTGCGGCCGTTGGTACGGCTTCGTCATACTGAGGGGCGCATTTTCACC
CCTCGCTCTGGATTTTCCATTTTCAGTGCGGGTGCGGGGTGCGCTTATCAGG----
GAGCTTATCT-CCTACACCCAAAACCCTG------TTGCAACA--ACACA----CCCACC--
CTACCGCTACTTCATTC--ATCCTCAACCCCC---------------------
ATCGACATTCCTGAATCAATCAAGCAAACCGCCAGGAAATCATGCTGACTTTCTATCTA
CATAGCCGCCGAGCTTGGTAAGGG
>Diaporthe_fusiformis_JZB320156
NNNNNNNNNNNNNNNNNNNNNNNNNNNNNNNNNNNNNNNNNACA-
GACTGTCACCCTGT-CCTCAGCATTTT---GCCTC-
AAATCATCAGTGTGCGGCTGTTGGTACGGCTTCGTCATACTGAGGGGCGCATTTTCACC
CCTCGCTCTGGATTTTCCA-TTTCAGTGCGGGTGCGGGGTGCGCTTATCAGG----
GAGCTTATCT-CCTACACCCAAAACCCTG------TTGCAACA----ACA----
ACCACACCATGCCGTTACCTCATTC--ACCCTCAAAACCC---------------------
CATCGATATCCTTGAATTGATCAAGCAAACCGCGTCGAAATCATGCTGACTCCGTATCT
ACACAGCCGCCGAGCTTGGTAAGGG
>Diaporthe_guangxiensis_JZB320094
NNNNNNNNNNNNNNNNNNNNNNNNNNNNNNNNNNCATCATAG--
ACATGCCACTCTGC-CCTCAGCATTTT---GCCTC-
AAATCATCAGTGTGCGGCTGTTGGTACAGCTTCGTCATACTGAGGGGCGCATTTTCACC
CCTCGCTCTGGATTTTCCATTTTCAGTGCGGGTGCGGGGTGCGCTTATCAGG----
GAGCTTATCT-CCTACACCCAAAACCCTG------TTGCAACA--ACACA----CCCACC-----------
TTCCTTGC--
AGTTACTACCACCACTGCATTCATCCTCAACCCCCATCGACATCCTTGAATCAATCAAG
CAAACCTCCAGGAAATCATGCTGACTTTCTATCTACATAGCCGCCGAGCTTGGTAAGGG
>Diaporthe_anhuiensis_CNUCC_201902
NNNNNNNNNNNTTGGGGTATCA-------TACAATCATGAA-ATATGCTCTCCTGC-
CCTCAGCCTTTT---GCCTC-
ATATCATCAGTGTGCGGCTGTTGGTACGGCTTCGTCATACTGAGGGGCGCATTTTCACCC
CTCGCTTTGGATTTTCCATTTTCAGTGCGGGTGCGGGGTGCGCTTATCAGG----
GAGCTTATCT-CCTACACCCAAAACCCTG------TTGCAACA----ACA----CCCACC-----------
TTCCTTAC--
CGCTACTACCACCACTACATTCACCCTCAACCCCCATCAACATTTTTGAATCAATTAAG
CAAACCGTCAGGAAATCATGCTGACTTTCTACCTACACAGCCGCCGAGCTTGGTAAGGG
>Diaporthe_annellsiae_BRIP_59731a
GAGAAGGAAGGTTAGTAAACAT-------CACAATCATAAACATGCTCTCCTCTGC-
CCTCAGCCCTTT---GCCTC-

AAATCATCAGTGTTCGGCTGTTGGTACGGCTTCGTCATACTGAGGGGCGCATTTTCACCC
CTCGCTCTGGATTTTCCATTTTCAGTGCGGGTGCGGGGTGCGCTTATCAGG----
GAGCTTATCT-CCTACACCCAAAACCCTG------TTGCTACA----GCA----CCCACC-----------
TTCCTTGC--
TGTTACTACCACCACTTCATTCACACTCACCCCCCATCGACATGCTTGAATCAATCAAGC
AAACCTCCAGGAAATCATGCTGACTTTCTATCTACACAGCCGCCGAGCTTGGTAANNN
>Diaporthe_arecae_CBS_161_64
GAGAAGGAAGGTTAGTAAACAT-------CACAATCATAAGCACGCTCTCCTCTGC-
CCTCAGCCCTTT---GCCTC-
AGATCATCAGTGTGCGGCTGTTGGTACAGCTTCGTCATACTGAGGGGCGCATTTTCACC
CCTCGCTCTGGATTTTCCATTTTCAGTGCGGGTGCGGGGTGCGCTTATCAGG----
GAGCTTATCT-CCTACACCCAAAACCCTG------TTGCAACA--ACACA----CCCACC----------
TTCCTTGC--AGTTATTACCACC--
GGAAATCATGCTGACTTTCTATCTACATAGCCGCCGAGCTTGGTAAGGG
>Diaporthe_arengae_CBS_114979
GAGAAGGAAGGTTAGTAAACAT-------CACAATCATAAA-ACATGCTCTCCTGC-
CCTCAGCCTTTT---GCCTC-
AAATCATCAGTGTGCGGCTGTTGGTACAGCTTCGTCATACTGAGGGGCGCATTTTCACC
CCTCGCTCTGGATTTTCCATTTTCAGTGCGGGTGCGGGGTGCGCTTATCAGG----
GAGCTTATCT-CCTACACCCAAAACCCTG------TTGCAACAACACACA----CCCACC----------
-TTCCTTGC--AGTTACTACCACC--
GGAAATCATGCTGACTTTCTATCTACATAGCCGCCGAGCTTGGTAAGGG
>Diaporthe_aseana_MFLUCC_12_0299a
GAGAAGGAAGGTTAGTAAACAT-------CACAATCATAGA-ACATGCTACCCTAT-
CCCCAGCACTTT---GCCTC-
AAATCATCAGTGTGCGGCTGTTGGTATGGCTTCGTCATACTGAGGGGCGCATTTTCACCC
CTCGCTTTGGATTTTCCA-TTTCAGTGCGGGTGCGGGGTGCGCTTATCAGG----
GAGCTTATCT-CCTACACC-AAAACCCTG------TTGCAACA----ACA----CCCACT-------------
CCTTGCCGCGCTACTACCACC--
GAAAATCATGCTGACGCTTTTTCTACACAGCCGCCGAGCTTGGTAAGGG
>Diaporthe_australiana_BRIP_66145
GAGAAGGAAGGTTAGTAAACAT-------CACAACTATAGA-ACATGCCACCCTGT-
CCTCAGCATTTT---GCCTC-
AAATCATCAGTGTGCGGCTGTTGGTACGGCTTCGTCATACTGAGGGGCGCATTTTCACC
CCTCGCTCTGGTTTTTCCA-TTTCAGTGCGGGTGCGGGGTGCGCTTATCAGG----
GAGCTTATCT-CCTACACCCAAAACCCTG------TTGCAACA----ATA----
ACCACACCATGCTGTTCCTTCATTC--ACCCTCAAATACC--------------------
TCATCGATATCCTTGAATTGATCAAGCAAACCGCCTCGAAATCATGCTGACATCGTATC
TACACAGCCGCCGAGCTTGGCAAGGG
>Diaporthe_camelliaeoleiferae_HNZZ027
NNNNNNNAAGGTTAGTAAACAC-------CACCATCATAGA-ACATGCCACTCTGC-
CCTCACCATTTT---GCCTC-
AAATCATCAGTGTGCGGCTGTTGGTACAGCTTCGTCATACTGAGGGGCGCATTTTCACC

CCTCGCTCTGGATTTTCCATTTTCAGTGCGGGTGCGGGGTGCGCTTATCAGG----
GAGCTTATCT-CCTACACCCAAAACCCTG------TTGCAACA--ACACA----CCCACC-----------
TTCCTTGC--
AGTTACTACCACCACTGCATTCATCCTCAACCCCCATCGACATCCTTGAATCAATCAAG
CAAACCTCCAGGAAATCATGCTGACTTTCTATCTACATAGCCGCCGAGCTTGGTAANNN
>Diaporthe_cercidis_CFCC_52565
GAGAAGGAAGGTTAGTAAACAT-------
CACAATCATAAACATGCTCTCCTCTGCCCCTCAGCCTTTT---GCCTC-
AAATCATCAGTGTGCGGCCGTTGGTACGGCTTCGTCATACTGAGGGGCGCATTTTCACC
CCTCGCTCTGGATTTTCCATTTTCAGTGCGGGTGCGGGGTGCGCTTATCAGG----
GAGCTTATCT-CCTACACCCAAAACCCTG------TTGCAACA--ACACA----CCCACC--
CTACCGCTACTTCATTC--ATCCTCAACCCCC---------------------
ATCGACATCCTTGAATCAATCAAGCAAACCGCCAGGAAATCATGCTGACTTTCTATCTA
CATAGCCGCTGAGCTTGGCAAGGG
>Diaporthe_cf_heveae_2_CBS_681_84
GAGAAGGAAGGTTAGTAAACAT-------CGCAATCATACA-ACTTGCTTTCCTGC-
CCTCAGCATTTT---
GCCTCAAAATCATCAGTGTGCGGCTGTTGGTACGGCTTCGTCATACTGAGGGGCGCATT
TTCACCCCTCGCTCTGGATTTTCCATTTTCAGTGCGGGTGCGGGGTGCGCTTATCAGG----
GAGCTTATCT-CCTACACCCAAAACCCTG------TTGCAACA----ACA----CCCACC-----------
TTTCTTGC--TGCTACTACCACC--
GGAAACCATGCTGACTTTCTATCTACACAGCCGCCGAGCTTGGTAAGGG
>Diaporthe_chrysalidocarpi_SAUCC194_35
NNNNNNNNNNNNNNNNNNGCGTA-------CTCCATCATAAACATGCTCTCCTCTGC-
CCTCAGCCTTTT---GCCTC-
AAATCATCAGTGTGCGGCTGTTGGTACGGCTTCGTCATACTGAGGGGCGCATTTTCACC
CCTCGCTCTGGATTTTCCATTTTCAGTGCGGGTGCGGGGTGCGCTTATCAGG----
GAGCTTATCT-CCTACACCCAAAACCCTG------TTGCAACA--ACACA----CCCACC--
CTACCGCTACTTCATTC--ATCCTCAACCCCC---------------------
ATCGACATCCTTGAATCAATCAAGCAAACCGCCAGGAAATCATGCTGACTTTCTATCTA
CATAGCCGCTGAGCTTNNNNNNNNN
>Diaporthe_drenthii_BRIP_66524
GAGAAGGAAGGTTAGTAAACAC-------CACAATCATAAA-ACATGCTCTCCTGC-
CCTCAGCCTTTT---GCCTC-
AAATCATCAGTGTGCGGCTGTTGGTACAGCTTCGTCATACTGAGGGGCGCATTTTCACC
CCTCGCTCTGGATTTTCCATTTTCAGTGCGGGTGCGGGGTGCGCTTATCAGG----
GAGCTTATCT-CCTACACCCAAAACCCTG------TTGCAACA--ACACA----CCCACC--
CTACCGCCACTTCATTC--ATCCTCAACCCCC---------------------
ATCGACATCCTTGAATCAATCAAGCAAACCACCAGGAAATCATGCTGACTTTTTATCTA
CACAGCCGCCGAGCTTGGCAAGGG
>Diaporthe_endocitricola_ZHKUCC20_0012
GAGAAGGAAGGTTAGTAAACAT-------CACAATCATAAACATGCTCTCCTTTGC-
ACTCAGCCTTTT---GCCTC-

AAACCATCAGTGTGCGGCTGTTGGTACGGCTTCGTCATACTGAGGGGCGCATTTTCACC
CCTCGCTCTGGATTTTCCATTTTCAGTGCGGGTGCGGGGTGCGCTTATCAGG----
GAGCTTATCT-CCTACACCCAAAACCCTG------TTGCAACA--ACACA----CCCACC--
CTACCGCCACTTCATTC--ATCCTCAACCCCC---------------------
ATCGACATCCTTGAATCAATCAAGCAAACCGCCAGGAAATCATGCTGACTTTCTATCTA
CATAGCCGCCGAGCTTGGTAAGGG
>Diaporthe_eugeniae_CBS_444_82
GAGAAGGAAGGTTAGTAAACAT-------CACAGTCATAAGCATGCTCTCCTCTGC-
CCTCAGCCCTTT---GCCTC-
AGACCATCAGTGTGCGGCTGTTGGTACAGCTTCGTCATACTGAGGGGCGCATTTTCACC
CCTCGCTCTGGATTTTCCATTTTCAGTGCGGGTGCGGGGTGCGCTTATCAGG----
GAGCTTATCT-CCTACACCCAAAACCCTG------TTGCAACA--ACACA----CCCACC----------
TTCCTTGC--AGTTATTACCACC---
GGAAATCATGCTGACTTTCTATCTACATAGCCGCCGAGCTTGGTAAGGG
>Diaporthe_gossiae_BRIP_59730a
GAGAAGGAAGGTTAGTAAACAT-------CACAATCATAAACATGCTCTCCTCTGC-
CCTCAGCCTTTT---GCCTC-
AGATCATCAGTGTGCGGCTGTTGGTACAGCTTCGTCATATTGAGGGGCGCATTTTCACCC
CTCGCTCTGGATTTTCCA-TTTCAGTGCGGGTGCGGGGTGCGCTTATCAGG----
GAGCTTATCT-CCTACACCCAAAACCCTG------TTGCAACA--ACACA----CCCACC-----------
TTTCTTGC--
AGTTACTACCACCACTTCATTCATCCTCAACCCCCATCGACATCCTTGAATCAATCAAG
CAAATCGACAGGAAATCATGCTGACTTTCTATCTACATAGCCGCCGAGCTTGGTAANN
N
>Diaporthe_hongkongensis_CBS_115448
GAGAAGGAAGGTTAGTAAACAT-------CACAATCATAGA-ACATGCCACCCTGT-
CCTCAGCATTTT---GCCTC-
AAATCATCAGTGTGCGGCTGTTGGTACGGCTTCGTCATACTGAGGGGCGCATTTTCACC
CCTCGCTCTGGATTTTCCA-TTTCAGTGCGGGTGCGGGGTGCGCTTATCAGG----
GAGCTTATCT-CCTACACCCAAAACCCTG------TTGCAACA----ACA----ACCACA--------
CCATGCCGTTC---
GAAATCATGCTGACATCGTATCTACACAGCCGCCGAGCTTGGTAAGGG
>Diaporthe_howardiae_BRIP_59697a
GAGAAGGAAGGTTAGTACATAT-------CACAATCATAGA-ACATGCCCCTCTAC-
CCTCAGCATTTT---GCCTC-
AAATCGTCAGTGTGCGGCTGTTGGTATGGCTTCGTCATACTGAGGGGCGCATTTTCACCC
CTCGCTCTGGATTTTCCATTTTCAGTGCGGGTGCGGGGTGTGCTTATCAGG----
GAGCTTATCT-CCTACACCCAAAACCCTG------TTGCAACA----ACA----CCGACT----------
TTCCTTAC--
CGCTACTACCACCACTACATTTACCCTCAACCCCCATCAACATTTTTGAATCAATCCAGC
AAACCGCCAGGAAATCATGCTGACTTTCTATCTTCATAGCCGCCGAGCTTGGCAAGGG
>Diaporthe_huangshanensis_CNUCC_201903

NNNNNNNNNNNNNNNGTTTTTCCT-------ACAAAAAATAAACATGCTCTCCTCTGC-
CCTCAGCATTTT---GCCTC-
AAATCATCAGTGTGCGGCTGTTGGTACGGCTTCGTCATACTGAGGGGCGCATTTTCACC
CCTCGCTCTGGATTTTCCATTTTCAGTGCGGGTGCGGGGTGCGCTTATCAGG----
GAGCTTATCT-CCTACACCCAAAACCCTG------TTGCAACA--ACACA----CCCACC--
CTACCGCCACTTCATTC--ATCCTCAACCCCC---------------------
ATCGACATCCTTGAATCAATCAAGCAAACCGCCAGGAAATCATGCTGACTTTCTATCTA
CATAGCCGCCGAGCTTGGTAAGGG
>Diaporthe_hunanensis_HNZZ023
NNNNNNNAAGGTTAGTAAACAT-------CACAATCATAAACATGCTCTCCTCTGC-
CCTCAGCCTTTT---GCCTC-
AAATCATCAGTGTGCGGCCGTTGGTACGGCTTCGTCATACTGAGGGGCGCATTTTCACC
CCTCGCTCTGGATTTTCCATTTTCAGTGCGGGTGCGGGGTGCGCTTATCAGG----
GAGCTTATCT-CCTACACCCAAAACCCTG------TTGCAACA--ACACA----CCCACC--
CTACCGCTACTTCATTC--ATCCTCAACCCCC---------------------
ATCGACATTCCTGAATCAATCAAGCAAACCGCCAGGAAATCATGCTGACTTTCTATCTA
CATAGCCGCCGAGCTTGGTAANNN
>Diaporthe_krabiensis_MFLUCC_17_2481
NNNNNNAACCGACAATAAAGGG--------ACAAAGATAAG-GT---------------------------------
AAAAAATAAGTGTGAGGCTGTTGGTACGGCTTCGTCATACTGAGGGGCGCATTTTCACC
CCTCGCTCTGGATTTTCCATTTTCAGTGCGGGTGCGGGGTGCGCTTATCAGG----
GAGCTTATCT-CCTACACCCAAAACCCTG------TTGCAACA--ACACA----CCCACC----------
TTTCTTGC--AGTTACTACCACAACTTCATCCAC-
CTCGACCCCCATCGACATCCTTGAATCAATCAAGCAAACCGCCAAGAAATCATGCTGA
CTTTCTATCTACATAGCCGCCGAGCTTGGTAAGGG
>Diaporthe_limonicola_CBS_142549
GAGAAGGAAGGTTAGTAAACAT-------CACAGTCATAAACATGCTCTCCTCTGC-
CCTCAGCCTTTT---GCCTC-
AGATCATCAGTGTGCGGCTGTTGGTACAGCTTCGTCATACTGAGGGGCGCATTTTCACC
CCTCGCTCTGGATTTTCCATTTTCAGTGCGGGTGCGGGGTGCGCTTATCAGG----
GAGCTTATCTCCCTACACCCAAAACCCTG------TTGCAACA--ACACA----CCCACC----------
TTTCTTGC--AGTTACTACCACC--
GGAAATCATGCTGACTTTCTATCTACATAGCCGCCGAGCTTGGTAAGGG
>Diaporthe_litchicola_BRIP_54900
NNNNNGGAAGGTTAGTAAACAT-------CACGATCATAAA-ACATGCTCTCCTGC-
CCTCAGCCTTTT---GCCTC-
AAATCATCAGTGTGCGGCTGTTGGTACGGCTTCGTCATACTGAGGGGCGCATTTTCACC
CCTCGCTCTGGATTTTCCATTTTCAGTGCGGGTGCGGGGTGCGCTTATCAGG----
GAGCTTATCT-CCTACACCCAAAACCCTG------TTGCAACA--ACACA----CCCACC----------
TTTCTTGC--CGCTATTACCACC--
CGAAACCATGCTGACTTTCTATCTACACAGCCGCCGAGCTTGGCAAGGG
>Diaporthe_lithocarpi_CGMCC_3_15175

GAGAAGGAAGGTTAGTAAACAT-------CACAATT---AA-GAACGTCACCCTGT-
CCTCAGCATTTT---GCCTC-
AAATCATCAGTGTGCGGCTGTTGGTACGGCTTCGTCATACTGAGGGGCGCATTTTCACC
CCTCGCTCTGGATTTTCCA-TTTCAGTGCGGGTGCGGGGTGCGCTTATCAGG----
GAGCTTATCT-CCTACACCCAAAACCCTG------TTGCAACA----ACA----ACCACA--------
CCATGCCGTTC--
GAAATCATGCTGACTCCGTATCTACACAGCCGCCGAGCTTGGTAAGGG
>Diaporthe_meliae_CFCC_53089
NNNNNNNNNNGTTAGTAAACAT-------CACAATCATAAACATGATCTCCTCTGC-
CCTCAGCCTTTT---GCCTC-
AAATCATCAGTGTGCGGCTGTTGGTATGGCTTCGTCATACTGAGGGGCGCATTTTCACCC
CTCGCTCTGGATTTTCCATTTTCAGTGCGGGTGCGGGGTGCGCTTATCAGG----
GAGCTTATCT-CCTACACCCAAAACCCTG------TTGCAACA----ACA----
CCCACCTTCCACCCACCTTCCTTAC--
CGCTACTACCACCACCTCATTCGCCCCCAACCCCCATCGACAT-
TTTCAATCGATCAAGCAAACCGTTAGGAAATCATGCTGACTTTCTATCTACACAGCCGC
CGAGCTTGGTAANNN
>Diaporthe_melitensis_CBS_142551
GAGAAGGAAGGTTAGTAAACAT-------CACAGTCATAAACATGCTCTCCTCTGC-
CCTCAGCCTTTT---GCCTC-
AGATCATCAGTGTGCGGCTGTTGGTACAGCTTCGTCATACTGAGGGGCGCATTTTCACC
CCTCGCTCTGGATTTTCCATTTTCAGTGCGGGTGCGGGGTGCGCTTATCAGG----
GAGCTTATCTCCCTACACCCAAAACCCTG------TTGCAACA--ACATA----CCCACC----------
TTTCTTGC--AGTTACTACCACC--
GGAAATCATGCTGACTTTCTATCTACATAGCCGCCGAGCTTGGTAAGGG
>Diaporthe_millettiae_GUCC9167
NNNNNNNNNNNNNNNNNGGTTAC------TCACA-TCATAAC-ATGCTCTCCTTTGC-
ACTCAGCCTTTT---GCCTC-
AAACCATCAGTGTGCGGCTGTTGGTACGGCTTCGTCATACTGAGGGGCGCATTTTCACC
CCTCGCTCTGGATTTTCCATTTTCAGTGCGGGTGCGGGGTGCGCTTATCAGG----
GAGCTTATCT-CCTACACCCAAAACCCTG------TTGCAACA--ACACA----CCCACC--
CTACCGCCACTTCATTC--ATCCTCAACCCCC---------------------
ATCGACATCCTTGAATCAATCAAGCAAACCGCCAGGAAATCATGCTGACTTTCTATCTA
CATAGCCGCCGAGCTTGGCAAGGG
>Diaporthe_musigena_CBS_129519
GAGAAGGAAGGTTAGTAAACAT-------CACGATCATAAA-ACATGCTCTCCTGC-
CCTCAGCCTTTT---GCCTC-
AAATCATCAGTGTGCTGCTGTTGGTACGGCTTCGTCATACTGAGGGGCGCATTTTCACCC
CTCGCTCTGGATTTTCCATTTTCAGTGCGGGTGCGGGGTGCGCTTATCAGG----
GAGCTTATCT-CCTACACCCAAAACCCTG------TTGCAACA--ACACA----CCCACC----------
TTCCTTGC--AGTTACTACCACC--
GGAAATCATGCTGACTTTCTATCTACATAGCCGCCGAGCTTGGCAAGGG
>Diaporthe_norfolkensis_BRIP_59718a

GAGAAGGAAGGTTAGTAAACAT-------CACAATCATAAA-ATATGCTCTCCTGC-
CTTCAGCCTTTT---GCCTC-
ATATCATCAGTGTGCGGCTGTTGGTATGGCTTCGTCATACTGAGGGGCGCATTTTCACCC
CTCGCTCTGGATTTTCCATTTTCAGTGCGGGTGCGGGGTGCGCTTATCAGG----
GAGCTTATCT-CCTACATCCAAAACCCTG------CTGCAACA----ACA----CCCACC-----------
TTCCTTAC--CGCTATTACCACTTTTTCATTCACCCTC-
ACCCCCATCAACATACTTGAATCAATCGAGCAAACCGCCAGGAAATCATGCTGACTTTT
TATCTACATAGCCGCCGAGCTTGGCAAGGG
>Diaporthe_oculi_HHUF_30565
NNNNNNGAAGGTTAGTAAACAT-------CACAATCATAAACATGCTCTCCTCTGC-
CCTCAGCCTTTT---GCCTC-
AAATCATCAGTGTGCGGCCGTTGGTACGGCTTCGTCATACTGAGGGGCGCATTTTCACC
CCTCGCTCTGGATTTTCCATTTTCAGTGCGGGTGCGGGGTGCGCTTATCAGG----
GAGCTTATCT-CCTACACCCAAAACCCTG------TTGCAACA--ACACA----CCCACC--
CTACCGCTACTTCATTC--ATCCTCAACCCCC---------------------
ATCGACATTCCTGAATCAATCAAGCAAACCGCCAGGAAATCATGCTGACTTTCTATCTA
CATAGCCGCCGAGCTTGNNNNNNN
>Diaporthe_osmanthi_GUCC9165
NNNNNNNNNNNNNNNNNGGTTAA----TTTCGCA-TCATAAA-ACATGCTCTCCTGC-
CCTCAGCCTGTT---GCTTC-
AAATCATCAGTGTGCGGCTGTTGGTACGGCTTCGTCATACTGAGGGGCGCATTTTCACC
CCTCGCTCTGGATTTTCCATTTTCAGTGCGGGTGCGGGGTGCGCTTATCAGG----
GAGCTTATCT-CCTACGCCCAAAACCCTG------TTGCAACA----ACA----CCCACC-----------
TTCCTTAC--
CGCTACTACCACCACTACATTCACCCTCAACTCCCATCAACATTTTCGAATCAATCAAG
CAAACCGCCTGGAAATCATGCTGACTTTCTATCTACACAGCCGCCGAGCTTGGCAAGGG
>Diaporthe_pascoei_BRIP_54847
NAGAAGGAAGGTTAGTACACAC-------CACAATCATAGA-ACATGCCACCCTGC-
CTCAAGCATTTT---GCATC-
AAATCATCAGTGTGCGGCTGTTGGTACGGCTTCGTCATACTGAGGGGCGCATTTTCACC
CCTCGCTCTGGATTTTCCATTTTCAGTGCGGGTGCGGGGTGCGCTTATCAGG----
GAGCTTATCT-CCTACACCCAAAACCCTG------TTGCAACA----ACA----CCCACC-----------
TTCCTTGC--CGCTACTCCCACC--
GGTAATCATGCTGACTTTCTATCTATACAGCCGCCGAGCTTGGCAAGGG
>Diaporthe_perseae_CBS_151_73
GAGAAGGAAGGTTAGTAAACAT-------CACGATCATAAA-ACATGCTCTCCTGC-
CCTCAGCCTTTT---GCCTC-
AAATCATCAGTGTGCGGCTGTTGGTACGGCTTCGTCATACTGAGGGGCGCATTTTCACC
CCTCGCTCTGGATTTTCCATTTTCAGTGCGGGTGCGGGGTGCGCTTATCAGG----
GAGCTTATCT-CCTACACCCAAAACCCTG------TTGCAACA--ACACA----CCCACC-----------
TTCCTTGC--AGTTATTACCACC--
GGGAATCATGCTGACTTTCTATCTACATAGCCGCCGAGCTTGGTAAGGG
>Diaporthe_pescicola_MFLUCC_16_0105

GAGAAGGAAGGTTAGTAAACAT-------CACAATCATAAACATGCTCTCCTCTGC-
CCTCAGCCTTTT---GCCTC-
AAATCATCAGTGTGCGGCCGTTGGTACGGCTTCGTCATACTGAGGGGCGCATTTTCACC
CCTCGCTCTGGATTTTCCATTTTCAGTGCGGGTGCGGGGTGCGCTTATCAGG----
GAGCTTATCT-CCTACACCCAAAACCCTG------TTGCAACA--ACACA----CCCACC-------------
-------------CTACCGCT---
GGAAATCATGCTGACCTTTTCTCTACATAGCCGCCGAGCTTGGTAAGGG
>Diaporthe_podocarpi-macrophylli_CGMCC3_18281
GAGAAGGAAGGTTAGTAAACAT-------CACCATCATAGA-ATATGCCACTCTGC-
CCTCAGCATTTT---GCCTC-
AAATCATCAGTGTGCGGCTGTTGGTATGGCTTCGTCATACTGAGGGGCGCATTTTCACCC
CTCGCTCTGGATTTTCCATTTTCAGTGCGGGTGCGGGGTGCGCTTATCAGG----
GAGCTTATCT-CCTACACCCAAAACCCTG------TTGCAACA----ACA----CCGACC-----------
TTCCTTAC--CGCTATTACCACC---
GGGAACCATGCTGACTTTCTATCTACACAGCCGCCGAGCTTGGTAAGGG
>Diaporthe_pseudomangiferae_CBS_101339
GAGAAGGAAGGTTAGTAAACAT-------CACAATCATAAACATGCTCTCCTCTGC-
CCTCAGCCTTTT---GCCTC-
AGATCACCAGTGTGCGGCTGTTGGTACAGCTTCGTCATACTGAGGGGCGCATTTTCACC
CCTCGCTCTGGATTTTCCATTTTCAGTGCGGGTGCGGGGTGTGCTTATCAGG----
GAGCTTATCT-CCTACACCCAAAACCCTG------TTGCAACA--ACACA----CCCACC-------------
-------------CTACCGCC---
GGAAATCATGCTGACCTTTTCTCTACATAGCCGCCGAGCTTGGTAAGGG
>Diaporthe_pseudooculi_HHUF_30617
GAGAAGGAAGGTTAGTAAACAT-------CACAATCATAAATATGCCCTCCTCTGC-
CCTCAGCCTTTT---GCCTC-
AAATCATCAGTGTGCGGCTGTTGGTACAGCTTCGTCATACTGAGGGGCGCATTTTCACC
CCTCGCTCTGGATTTTCCATTTTCAGTGCGGGTGCGGGGTGCGCTTATCAGG----
GAGCTTATCT-CCTACACCCAAAACCCTG------TTGCAACA--ACACA----CCCACC--
CTACCGCTACTTCATTC--ATCCTCAACCCCC----------------------
ATCGACATCCTTGAATCAATCAAGCAAACCGCCAGGAAATCATGCTGA--
TTCTATCTACATAGCCGCTGAGCTTGGTAAGGG
>Diaporthe_pseudophoenicicola_CBS_462_69
GAGAAGGAAGGTTAGTAAACAT-------CACAATCATGAACATGCTCTCCTCTGC-
CCTCAGCCTTTC---GCCTC-
AGATCATCAGTGTGCGGCTGTTTGTACAGCTTCGTCATACTGAGGGGCGCATTTTCACCC
CTCGCTCTGGATTTTCCATTTTCAGTGCGGGTGCGGGGTGCGCTTATCAGG----
GAGCTTATCTCCCTACACCCAAAACCCTG------TTGCAACA--ACACA----CCCACC-----------
TTTCTTGC--AGTTGCTACCACC---
GGAAATCATGCTGACTTTCTATCTACATAGCCGCCGAGCTTGGCAAGGG
>Diaporthe_pterocarpicola_MFLUCC_10_0580a
GAGAAGGAAGGTTAGTAAACAT-------CACAATCATAAGCATGCTCTCCTCTGC-
CCTCAGCCCTTT---GCCTC-

AGATCATCAGTGTGCGGCTGTTGGTACAGCTTCGTCATACTGAGGGGCGCATTTTCACC
CCTCGCTCTGGATTTTCCATTTTCAGTGCGGGTGCGGGGTGCGCTTATCAGG----
GAGCTTATCT-CCTACACCCAAAACCCTG------TTGCAACA--ACACA----CCCACC-----------
TTCCTTGC--AGTTATTACCACC--
GGAAATCATGCTGACTTTCTATCTACATAGCCGCCGAGCTTGGTAAGGG
>Diaporthe_rhodomyrti_CFCC_53101
NNNNNNNAAGGTTAGTAAACAT-------CACAATTACAGA-ACATGTCACCCTGT-
CCTCAGCATTTT---GCCTC-AAATCATCAGTGTGCGGCTGTTGGTACGGCTTC-------
TGAGGGGCGCATTTTCACCCCTCGTTCTGGATTTTCCA-
TTTCAGTGCGGGTGCGGGGTGCGCTTATCAGG----GAGCTTATCT-
CCTACACCCAAAACCCTG------TTGCAACA----ACA----
ACCACACCATGCCGTTACCTCATTC--ACCCTCAAAACCC--------------------
CATCGATATCCTTGAATTGATCAAGCAAACCGCGTCGAAATCATGCTGACTCCGTATCT
ACACAGCCGCCGAGCTTGGTAANNN
>Diaporthe_schimae_CFCC_53103
NNNNNNNAAGGTTAGTAAACAT-------CACCATCATAGA-ACATGCC--TCTGC-
CCTCACCATTTT---GCCTC-
AAATCATCAGTGTGCGGCTGTTGGTATGGCTTCGTCATACTGAGGGGCGCATTTTCACCC
CTCGCTCTGGATTTTCCATTTTCAGTGCGGGTGCGGGGTGCGCTTATCAGG----
GAGCTTATCT-CCTACACCCAAAACCCTG------TTGCAACA----ACA----CCGACC-----------
TTCCTTAC--
CGCTACCACCACCACTACATTCACCCTCAACCCCCATCAACATTTTTGAATCAATCAAG
CAAACCGCCAGGGTATCATGCTGACTTTCTATCTACATAGCCGCCGAGCTTGGTAANNN
>Diaporthe_sennae_CFCC_51636
NNNNNGGAAGGTTAGTAAACAC-------CACCATCATAGA-ACACGCCACTCTGC-
CCTCAGCATTTT---GCCTC-
AAATCATCAGTGTGCGGCTGTTGGTACAGCTTCGTCATACTGAGGGGCGCATTTTCACC
CCTCGCTCTGGATTTTCCATTTTCAGTGCGGGTGCGGGGTGCGCTTATCAGG----
GAGCTTATCT-CCTACACCCAAAACCCTG------TTGCAACA--ACACA----CCGACC-----------
TTCCTTAC--CGCTACTACCACC--
GGAAATCATGCTGACTTTCTATCTACACAGCCGCCGAGCTTGGCAANNN
>Diaporthe_spinosa_PSCG_383
NNNNNNNNNNNNNNNNNNNNNNNNNNNNNNNNNNNNNNNNNNNNNNNNNTCCT
CTGC-CCTCAGCCTTTT---GCCTC-
AAATCATCAGTGTGCGGCCGTTGGTACGGCTTCGTCATACTGAGGGGCGCATTTTCACC
CCTCGCTCTGGATTTTCCATTTTCAGTGCGGGTGCGGGGTGCGCTTATCAGG----
GAGCTTATCT-CCTACACCCAAAACCCTG------TTGCAACA--ACACA----CCCACC--
CTACCGCTACTTCATTC--ATCCTCAACCCCC---------------------
ATCGACATTCCTGAATCAATCAAGCAAACCGCCAGGAAATCATGCTGACTTTCTATCTA
CATAGCCGCCGAGCTTGGTAAGGG
>Diaporthe_taoicola_MFLUCC_16_0117
GAGAAGGAAGGTTAGTAAACAT-------CACAATCATAGACATGCTCTCCTCTGC-
CCTCAGCCTTTT---GCCTC-

AAATCATCAGTGTGCGGCTGTTGGTACGGCTTCGTCATACTGAGGGGCGCATTTTCACC
CCTCGCTCTGGATTTTCCATTTTCAGTGCGGGTGCGGGGTGCGCTTATCAGG----
GAGCTTATCT-CCTACACCTAAAACCCTG------TTGCAACA--ACACA----CCCACC----------
TTCCTTAC--CGCTACTACCACC---
GGAAATCATGCTGACTTTCTATCTACATAGCCGCCGAGATTGGCAAGGG
>Diaporthe_tectonigena_MFLUCC_12_0767
GAGAAGGAAGGTTAGTAAACAT-------CACAATCATAGA-ACATGCTACCCTAT-
CCCCAGTACTTT---GCCTC-
AAATCATCAGTGTGCGGCTGTTGGTATGGCTTCGTCATACTGAGGGGCGCATTTTCACCC
CTCGCTTTGGATTTTCCA-TTTCAGTGCGGGTGCGGGGTGCGCTTATCAGG----
GAGCTTATCT-CCTACACC-AAAACCCTG------TTGCAACA----ACA----CCCACT------------
CCTTGCCGCGCTACTACCACC---GAAAATC-
TGCTGACNNNNNNNNNNNNNNNNNNNNNNNNNNNNNN
>Diaporthe_viniferae_JZB320071
GAGAAGGAAGGTTAGTAAATAT-------CACAGTCACGGA-ACATGCCACTCTGC-
CCTCAGCATTTT---GCCTC-
AAATCATCAGTGTGCGGCTGTTGGTACAGCTTCGTCATACTGAGGGGCGCATTTTCACC
CCTCGCTCTGGATTTTCCATTTTCAGTGCGGGTGCGGGGTGCGCTTATCAGG----
GAGCTTATCT-CCTACACCCAAAACCCTG------TTGCAACA--ACACA----CCCACC----------
TTCCTTGC--
AGTTACTACCACCACTGCATTCATCCTCAACCCCCATCGACATCCTTGAATCAATCAAG
CAAACCTCCAGGAAATCATGCTGACTTTCTATCTACATAGCCGCCGAGCTTGGTAAGGG
>Diaporthe_xishuangbanica_CGMCC_3_18282
GAGAAGGAAGGTTAGTAAACAC-------CACAATCATAAA-ACATGCTACCCTGC-
CCCCAGCACTTT---GCCTC-
AAATCATCAGTGTGCGGCTGTTGGTACGGCTTCGTCATACTGAGGGGCGCATTTTCACC
CCTCGCTCTGGATTTTCCATTTTCAGTGCGGGTGCGGGGTGCGCTTATCAGG----
GAGCTTATCT-CCTACACC-AAAACCCTG------CTGTAACA--ACACAACACCACACC---------
CACTCACTGC--CGCTACTACCACC---
GAAAATCATGCTGACTCTTTTCCTACACAGCCGCCGAGCTTGGTAAGGN
>Diaporthe_salinicola_MFLU18_0533
NNNNNNNNNNNNNNNNNNNNNNNNNNNNNNNNNNNNNNNNNNNGCCAC
CCTGT-CCTCAGCATTTT---GCCTC-
AAATCATCAGTGTGCGGCTGTTGGTACCGCTTCGTCATACTGAGGGGCGCATTTTCACCC
CTCGCTCTGGATTTTCCA-TTTCAGTGCGGGTGCGGGGTGCGCTTATCAGG----
GAGCTTATCT-CCTACACCCAAAACCCTG------TTGCAACA--ACAAC----CACACC--
ATGCCGTTCCTTCATTC--ACCCTCAAATACC-------------------
CCATCGATATCCTTGAATTGATCAAGCAAACCGCCTCGAAATCATGCTGACATCGTATC
TACACAGCCGCCGAGCTTGGTAAGGG
> Diaporthe_africana_STMA18294

GAGAAGGAAGGTTAGTAAACAT-------CACAATTATAGA-ACATGCCACCCTGT-
CCTCAGCATTTT---GCCTC-

AAATCATCAGTGTGCGGCTGTTGGTACGGCTTCGTCATACTGAGGGGCGCATTTTCACC
CCTCGCTCTGGATTTTCCA-TTTCAGTGCGGGTGCGGGGTGCGCTTATCAGG----
GAGCTTATCT-CCTACACCCAAAACCCTG------TTGCAACA--ACAAC----CACACC--
ATGCCGTTCCTTCATTC--ACCCTCAAATACC-------------------
CCATCGATACACTTGAATTGATCAAGCAAACCGCCTCGAAATCATGCTGACATCGTATC
TACACAGCCGCCGAGCTTGGTAAGGN

>Diaporthe_africana_CBS_150080
GAGAAGGAAGGTTAGTAAACAT-------CACAATTATAGA-ACATGCCACCCTGT-
CCTCAGCATTTT---GCCTC-
AAATCATCAGTGTGCGGCTGTTGGTACGGCTTCGTCATACTGAGGGGCGCATTTTCACC
CCTCGCTCTGGATTTTCCA-TTTCAGTGCGGGTGCGGGGTGCGCTTATCAGG----
GAGCTTATCT-CCTACACCCAAAACCCTG------TTGCAACA--ACAAC----CACACC--
ATGCCGTTCCTTCATTC--ACCCTCAAATACC-------------------
CCATCGATACACTTGAATTGATCAAGCAAACCGCCTCNNNNNNNNNNNNNNNNNNN
NNNNNNNNNNNNNNNNNNNNNNNNNNNNN

- Alignment of the *tub2* sequences used in the second phylogenetic study

>Diaporthe_amygdali_CBS_126679

GTCGGCCCATGGCTGCTTTCGCATCCT------------CTGCCCCTGAGCCTA-
CCCCACCATCGCGACCA--CACCCACGATCGGGCCTC-AAAACACCAC-
AAATACCCTGAAACAA-GCACTTCCATGC-
CTTCGAAGACGCGTCAGATTGCTAACATGGCC-
TTTTTCTCGCCCACAGGTTCATCTCCAGACCGGCCAATGCGTAAGTTGCTCCTGTCAACA
CACCACCGCACCTTATCGCCG---
CCTGTAGCTGACACGTTTCCCAGGGTAACCAAATCGGTGCTGCTTTCTGGTGCGTCCCA
GC------------TCCATCACCGCGATACTC-
GACGCGCGACAACACGACCTCGCAACATCCTTACTGACCTCGACTCGTAGGCAAACCA
TCTCTGGCGAGCACGGCCTCGACACCAATGGCGTGTATGCACCTCCTATTCCATGCCCA
TCAATCTCGG----
CCTCGGGGATTGGCACTGACAATTGCACAGCTACAACGGCACTTCCGAGCTCCAGCTCG
AGCGCATGAACGTCTACTTCAACGAGGTAAGTCAATCAC--
CATGTCATGGCGTTAACGAGCCCCCAACACGATGCCTTCTTTTGTCGCTTGGGCTTTGCT
GACCGCTTATCGCCCTAGGCCTCCGGCAACAAGTATGTTCCCCGCGCCGTCCTCGTCGA
TCTCGAGCCCGGTACCATGGACGCCGTC

>Diaporthe_fraxini-angustifoliae_BRIP_54781

GTCGGCCCATGCTGC-TTTCGCATCCTCTGCC------CCTGAGCCTGAGCCTA-
CCCCACCATCGCGACCG--
CACCCACGGTCGGGCCTCAAAAACATCACCAAACACCCTGGGAAAA-
GCACCCAGATGCCCTTGAAAAACGCGTCAGATTGCTAACGTGACC-

TTTTTCTCGACTACAGGTTCACCTTCAGACCGGCCAATGCGTAAGTCGCTCCTGTCA--
ACACCGACGGACCTTATCATCGCCACCCGTAGCTGACACGTTTCCCAGGGTAACCAAAT
CGGTGCTGCTTTCTGGTGC-------------------GTCCACCACCGCGACGCTC-
GACGCGCGACAATACGACCTCGAAACATCGTTACTGACCTCGACTCTCAGGCAAACCA
TCTCTGGCGAGCACGGCCTCGACAGCAATGGCGTGTATGCACCTCCTATTCCCTACCTC
CAGATCTCGTCCTCCCTGCCGGCTTGGCACTGACAATCACACAGTTACAACGGCACTTC
CGAGCTCCAGCTCGAGCGCATGAACGTCTACTTCAACGAGGTAAGTCAACAGC--
CACGTCG-----TTTACCATCTG-CAGCACG--
GTTTCCTGCCGTCGCCAAGGCCTTGCTAACGCGTTATCGCAC-
AGGCCTCCGGCAACAAGTATGTTCCTCGCGCCGTCCTCGTCGATCTCGAGCCCGGTACC
ATGGACGCCGTC

>Diaporthe_annellsiae_BRIP_59731a

NNNNNCCCATGCTGC-TTTCGCATCCTCTGCC------CCTGAGCCTGAGCCTA-
CCCCACCATCGCGACCG--
CACCCACGGTCGGGCCTCAAAAACATCACCAATCACCCTGGGAAAA-
GCACCCAGATGCCCTTGAAAAACGCGTCAGATTGCTAACGTGACC-
TTTTTCTCGACTACAGGTTCACCTTCAGACCGGCCAATGCGTAAGTTGCTCCTGTCA--
ACACCGCCGGACCTTATCATCGCCACCCGTAGCTGACACGTTTCCCAGGGTAACCAAAT
CGGTGCTGCTTTCTGGTGC------------------
GTCCACCACCGCGACGCTCTGACGCGCGACAATACGACCTCGAGCCATCGTTACTGAC
CTCGACTCTCAGGCAAACCATCTCTGGCGAGCACGGCCTCGACAGCAATGGCGTGTATG
CACCTCCTATTCTATACCTCCAGATCTCGTCCTCCCTGCCGGCTTGGCACTGACAATCAT
ACAGTTACAACGGCACTTCCGAGCTCCAGCTCGAGCGCATGAACGTCTACTTCAACGA
GGTAAGTCGACAGC--CACGTCG-----TTGACCATCTG-CAGCACG--
ATTTTCTGCCGTCGCTGAGGCCTTGCTAACGCGTTATCGCTC-
AGGCCTCCGGCAACAAGTATGTCCCTCGCGCCGTCCTCGTCGATCTCGAGCCCGGTACC
ATGGACGCCGTC

>Diaporthe_arecae_CBS_161_64

GTCGGCCCATGCTGC-TTTCGCATCCTCTGCC------CCTGAGCATGAGCCTA-
CCCCACCATCGCGACCG--
CACCCACGGTCGGGCCTCAAAAACATCACCAAACACCCTGGGGAAA-
GGACCCAAATGCCCATGAAAAACGCGTCAGATTGCTAACGTGAGC-
TTTTTCTCGACTACAGGTTCACCTTCAGACCGGTCAATGCGTAAGTTGCTCCCGTCA--
ACACCTCCAGACCTTATCATCGCCACCCGTAGCTGACACGTTTCCCAGGGTAACCAAAT
CGGTGCTGCTTTCTGGTGC-------------------GTCCACCACCGCGACGCTC-
GACGCGCGACAATACGATCTCGAACCATCGTTACTGACCTCGACTCTCAGGCAAACCAT
CTCTGGCGAGCACGGCCTCGACAGCAATGGCGTGTATGCACCTCCTATTCCTGCCTTCA
GATCTCGTCCTCCCTGCCGGCTTGGCACTGACAATCATACAGTTACAACGGCACTTCCG

AGCTCCAGCTCGAGCGCATGAACGTCTACTTCAACGAGGTAAGCCAACAGC--
CACGTCG-----TTGACCATGTG-CAGCACG--ATTTCCTGCCGTCGCC-
AGGCTTTGCTGACGCGTTATCGCAC-
AGGCCTCCGGCAACAAGTATGTCCCTCGCGCCGTCCTCGTCGATCTCGAGCCCGGTACC
ATGGACGCCGTC

>Diaporthe_arengae_CBS_114979

GTCGGCCCATGCTGC-TTTCGCATCCTCTGCC------CCTGAGCCTGAGCCTA-
CCCCACCATCGCGACCG--
CACCCACGGTCGGGCCTCAAAAACATCACCAAACACCCTGGGAAAA-
GCACCCAGATGCCCTTGAAAAACGCGTCAGATTGCTAACGTGACC-
TTTTTCTCGACTACAGGTTCACCTTCAGACCGGCCAATGCGTAAGTTGCTCCTGTCA--
ACACCGACGGACCTTATCATCGCCACCCGTAGCTGACACGTTTCCCAGGGTAACCAAAT
CGGTGCTGCTTTCTGGTGC-------------------GTCCACCACCGCGACGCTC-
GACGCGCGACAATACGACCTCGAAACATCGTTACTGACCTCGACTCTCAGGCAAACCA
TCTCTGGCGAGCACGGCCTCGACAGCAATGGCGTGTATGCACCTCCTATTCCCTACCTTC
AGATCTCGTCCTCCCTGCCGGCTTGGCACTGACAATCATACAGTTACAACGGCACTTCC
GAGCTCCAGCTCGAGCGCATGAACGTCTACTTCAACGAGGTAAGTCAACAGC--
CACGTCG-----TTGGCCATCTG-CAGCACG--
GTTTGCTGCCGTCGCTGGGCCCTTGCTAACGCGTTATCGCAC-
AGGCCTCCGGCAACAAGTATGTCCCTCGCGCCGTCCTCGTCGATCTCGAGCCCGGTACC
ATGGACGCCGTC

>Diaporthe_australiana_BRIP_66145

GCCGGCCCATGCTGC-TTTCGCATCCTCTGCC------CCTGAGCCTGAGCCTA-
CCCCACCATCGCGACCG--
CACCCACGGTCGGGCCTCAAAAACATCACCAAACACCCTGGGAAAC-
GCAACCAGATGCCCTTGAAAAACGCGTCAGATTGCTAACGTGAACTTTTTCCCGCCTA
CAGGTTCACCTTCAGACCGGCCAATGCGTAAGTTGCTCCTGTCA--
ACACCGCCGGACCTTATCATCGCTACCCGTAGCTGACACGTTTCGCAGGGTAACCAAAT
CGGTGCTGCTTTCTGGTGC-------------------GTCCACCACCGCGACGCTC-
GACGCGCGACAATACGACCTCGAACCATCGTTACTGACCTCGACTCTCAGGCAAACCA
TCTCTGGCGAGCACGGCCTCGACAGCAATGGCGTGTATGCACCTCCTATTCCCTACCTC
CAGATCTCGTCCTCCCTGCCGGCTTGGCACTGACAATCATACAGTTACAACGGCAGTTC
CGAGCTCCAGCTCGAGCGCATGAATGTCTACTTCAACGAGGTAAGTAAACAGC--
CACGTCG-----TTGACCATGTG-CAGCACG--
GTTTCCTGCCGTCGCCAAGGCCTTGCTAACGCGTTATCGCCC-
AGGCCTCCGGCAACAAGTATGTCCCTCGCGCCGTCCTCGTCGATCTCGAGCCCGGTACC
ATGGACGCCGTC

>Diaporthe_bounty_BRIP_59361a

GTCGGCCCATGCTGC-TTTCGCATCCTCTGCC------CCTGAGCCTGAGCCTA-
CCCCACCATCGCGACCG--
CACCCACGGTCGGACCTCAAAAACATCACCAAACACCCTGGGAAAG-
GCACCCAGATGCCCTTGAAAAACGCGTCAGATTGCTAATGTGAAC-
TTTTTCTCGACCACAGGTTCACCTTCAGACCGGCCAATGCGTAAGTTGCTCCTGTCA--
ACACCGACGGACCTTATCATCGCCACCCGTAGCTGACACGTTTCCCAGGGTAACCAAAT
CGGTGCTGCTTTCTGGTGC-------------------GTCCACCACCGCGACGCTC-
GACGCGCGACAATACGACCTCGAAACATCGTTACTGACCTCGACTCTCAGGCAAACCA
TCTCTGGCGAGCACGGCCTCGACAGCAATGGCGTGTATGCACCTCCTATTCCCTACCTC
CAGAACTCGTCCTCCCTGCCGGCTTGGCACTGACAATCACACAGTTACAACGGCACTTC
CGAGCTCCAGCTCGAGCGCATGAACGTCTACTTCAACGAGGTAAGTCAACAGC--
CACGTCG-----TTGGCCATCTG-CAGCACG--
GTTTGCTGCCGTCGCTGGGGCCCTGCTAACGCGTTATCGCAC-
AGGCCTCCGGCAACAAGTATGTCCCTCGCGCCGTCCTCGTCGATCTCGAGCCCGGTACC
ATGGACGCCGNN

>Diaporthe_cercidis_CFCC_52565

NNNNGCCCATGCTGC-TTTCGCATCCTCTGCC------CCTGAGCCTGAGCCTA-
CCCCACCATCGCGACCG--
CACCCACGGTCGGGCCTCAAAAACATCACCAAACACCCTGGGAAAA-
GCACCCAAATGTCCTTGAAAAACGCGTCAGATTGCTAACGTGACC-
TTTTTCTCGACTACAGGTTCACCTTCAGACCGGCCAATGCGTAAGTTGCTCCTGTCA--
ACACCGACGGACCTTATCATCGCCACCCGTAGCTGACACGTTTCCCAGGGTAACCAAAT
CGGTGCTGCTTTCTGGTGC-------------------GTCCACCACCGCGACGCTC-
GACGCGCGACAACATGACCTCGAGCCATCGTTACTGACCTCGACTCTCAGGCAAACCA
TCTCTGGCGAGCACGGCCTCGACAGCAATGGCGTGTATGCACCTCCTATTTCCTGCCTTC
AGATCTCGTCCTCCCTGCCGGCTTGGCACTGACAATCACACAGTTACAACGGCACTTCC
GAGCTCCAGCTCGAGCGCATGAACGTCTACTTCAATGAGGTAAGTCAACAGC--
CACGTCA-----TTGACCATCTG-CAGCACG--
GTTTGCTGCCGTCGCTGGGGCCTTGCTAACGCGTTATCGCAC-
AGGCCTCCGGCAACAAGTATGTCCCTCGCGCCGTCCTCGTCGATCTCGAGCCCGGTACT
ATGGACGCCGNN

>Diaporthe_drenthii_BRIP_66524

NNNNNNNNNNNNNNNNNNNNNNNNNNNNNNNN------NNNNNNNNTGAGCCTA-
CCCCACCATCGCGACCG--
CACCCACGGTCGGGCCTCAAAAACATCACCAAACACCCTGGGCGAA-
GCACCCAGATGCCCTTGTAAAACGCGTCAAATTGCTAACGTGACC-
TTTTTCTCGACCACAGGTTCACCTTCAGACCGGCCAATGCGTAAGTTGCTCCTGTCA--
ACACCGACGGACCTTATCATCCCCACCCGTAGCTGACACCTTTCCCAGGGTAACCAAAT

CGGTGCTGCTTTCTGGTGC-------------------GTCCACCACCGCGGCGCTC-
GACGCGCGACATTACGACCTCTAACCATTGTTACTGACCTCGACTCTCAGGCAAACCAT
CTCTGGCGAGCACGGCCTCAACAGCAATGGCGTGTATGCACCTCCTATTCCCTGCCTAC
AGATCTCGTCCTCCCAGCCGGCTTGGCACTGACAATCATACAGTTACAACGGCACTTCC
GAGCTCCAGCTCGAGCGCATGAACGTCTACTTCAACGAGGTAAGTCAACAGT--
CACGTCG-----TTGGCCATCTG-CAGCACG--GTTTCCTGCTGTCGCC-
AGGCTTTGCTAACGCGTTATCGCAC-
AGGCCTCCGGCAACAAGTATGTCCCTCGCGCCGTCCTCGTCGATCTCGAGCCCGGTACC
ATGGACGCCGTC

>Diaporthe_cf_heveae_2_CBS_681_84

GTCGGCCCATGCTGC-TTTCGCATCCTCTGCC------CCTGAGCCTGAGCCTA-
CCCCACCATCGCGACCG--
CACCCACGGTCGGGCCTCAAAAACATCACCAAACAGCCTGGGAAAA-
GCACCCAGATGCCCTTGAAAAACGCGTCAGATTGCTAACGTGACC-
TTTTTCTCGACTACAGGTTCACCTTCAGACCGGCCAATGCGTAAGTTGCTCCTGTCA--
ACACCGCCGGGCCTTATCATCGCCACCTGTAGCTGACACGTTTCCCAGGGTAACCAAAT
CGGTGCTGCTTTCTGGTGC-------------------GTCCACCGCCGCGACGCTC-
GACGCGCGACCATACGACCTCGAACCATCGTTACTGACCTCGACTATCAGGCAAACCA
TCTCTGGCGAGCACGGCCTCGACAGCAATGGCGTGTATGCACCTCCTATTCCCTACCTC
CAGATCTCGTCCTCCCTGCCGGCTTGGCACTGACAATCACACAGTTACAACGGCACTTC
CGAGCTCCAGCTCGAGCGCATGAACGTCTACTTCAACGAGGTAAGTCAACAGC--
CACGTCG-----TTGACCATCTG-CAGCACG--
ATCTTCTGCCGTCGCCGGGGCCTTGCTAACGCGTTATCACCC-
AGGCCTCCGGCAACAAGTATGTCCCTCGCGCCGTCCTCGTCGATCTCGAGCCCGGTACC
ATGGACGCCGTC

>Diaporthe_acuta_PSCG_047

NNNNNNNNNNNNNNNNNNNNNNNNNNNNNNNN------
NNNNNNNNNNNNNNNNNNNNNNNNNNNNNNNNNNNNNNNNNNNNNNNNNNN
NNNNNNNNNNNNNNNNNNNNNNNNNNNNNNNNNNNNNNNNNNNNNNNNNNN
NNNNNNNNNNNNNNNNNNNNNNNNNNNNNNNNNNNNNNNNNNNNNNNNNNN
NNNNNNNNNNNNNNNNNNNNNNNNNNNNNNNNNNNNNNNNNNNNNNNNNNN
NNNNNNNNNNNNNNNNNNNNNNNNNNNNNNNNNNNNNNNNNNNNNNNNNNN
NNNNNNNNNNGTT-------------------GGGCTCCACCGCGACGCTC-
GACGCGCGACAACATGACCTCGAGCCATCGTTACTGACCTCGACTCTCAGGCAAACCA
TCTCTGGCGAGCACGGCCTCGACAGCAATGGCGTGTATGCACCTCCTATTTCCTGCCTTC
AGATCTCGTCCTCCCTGCCGGCTTGGCACTGACAATCACACAGTTACAACGGCACTTCC
GAGCTCCAGCTCGAGCGCATGAACGTCTACTTCAACGAGGTAAGTCAACAGC--
CACGTCG-----TTGATCATCTG-CAGCACG--

ATTTTCTGCCGTCGCCGGGGCCTTGCTAACGCGTTATCGCCC-
AGGCCTCCGGCAACAAGTATGTCCCTCGCGCCGTCCTCGTCGATCTCGAGCCCGGTACC
ATGGACGCCGTC

>Diaporthe_aseana_MFLUCC_12_0299a

NNNNNNNNNNNNNNNNNNNNNNNNNNNNNNNN------
NNNNNNNNNNNNNNNNNNNNNNNNNNNNNNNNNNNNNNNNNNNNNNNNNNNNNNNNNNN
NNNNNNNNNNNNNNNNNNNNNNNNNNNNNNNNNNNNNNNNNNNNNNNNNNNNNNNNNNN
NNNNNNNNNNNNNNNNNNNNNNNNNNNNNNNNNNNNNNNNNNNNNNNNNNNNNNNNNNN
NNNNNNNNNNNNNNNNNNNNNNNNNNNNNNNNNNNNNNNNNNNNNNNNNNNNNNNNNNN
NNNNNNNNNNNNNNNNNNNNNNNNNNNNNNNNNNNNNNNNNNNNNNNNNNNNAACGTTCT
TCTGTGGTCC-------------------TAGTCCCCACTCGACGCTC-GACGCGCGACAATACGA-
TTCGATCCATCGTCACTGACCTCGACTCTCAGGCAAACCATCTCTGGCGAGCACGGCCT
CGACAGCAATGGCGTGTATGCACCTCCTATTCCCTACCTCCAGATCTCGTCCTCCCTGCC
GGCTTGGCATTGACAATCATACAGTTACAACGGCACTTCCGAGCTCCAGCTCGAGCGCA
TGAACGTCTACTTCAACGAGGTAAGTCAACAGC--CACGTCG-----TTGACCATCTG-
CAGCACG--GTTTCCTGCCGTCGCCAAGGCTTTGCTAACGCGTTATCGCCC-
AGGCCTCCGGCAACAAGTATGTTCCTCGCGCCGTCCTTGTCGATCTCGAGCCCGGTACC
ATGGACGCCGTC

>Diaporthe_camelliaeoleiferae_HNZZ027

NNNNNNNNNNNNNNNNNNNNNNNNNNNNNNNN------
NNNNNNNNNNNNNNNNNNNNNNNNNNNNNNNNNNNNNNNNNNNNNNNNNNNNNNNNNNN
NNNNNNNNNNNNNNNNNNNNNNNNNNNNNNNNNNNNNNNNNNNNNNNNNNNNNNNNNNN
NNNNNNNNNNNNNNNNNNNNNNNNNNNNNNNNNNNNNNNNNNNNNNNNNNNNNNNNNNN
NNNNNNNNNNNNNNNNNNNNNNNNNNNNNNNNNNNNNNNNNNNNNNNNNNNNNNNNNNN
NNNNNNNNNNNNNNNNNNNNNNNNNNNNNNNNNNNNNNNNNNNNNNNNNNNNNTGCTGC
TTTCTGGTGC-------------------GTCCACCACCGCGACGCTC-
GACGCGCGACAACATGACCTCGAGCCATCGTTACTGACCTCGACTCTCAGGCAAACCA
TCTCTGGCGAGCACGGCCTCGACAGCAATGGCGTGTATGCACCTCCTATTTCCTGCCTTC
AGATCTCGTCCTCCCTGCCGGCTTGGCACTGACAATCACACAGTTACAACGGCACTTCC
GAGCTCCAGCTCGAGCGCATGAACGTCTACTTCAATGAGGTAAGTCAACAGC--
CACGTCA-----TTGACCATCTG-CAGCACG--
GTTTGCTGCCGTCGCTGGGGCCTTGCTAACGCGTTATCGCAC-
AGGCCTCCGGCAACAAGTATGTCCCTCGCGCCGTCCTCGTCGATCTCGAGCCCGGTACT
ATGGACGCCGTN

>Diaporthe_anhuiensis_CNUCC_201902

NNNNNNNNNNNNNNNNNNNNNNNNNNNNNNNN------
NNNNNNNNNNNNNNNNNNNNNNNNNNNNNNNNNNNNNNNNNNNNNNNNNNNNNNNNNNN

NNNNNNNNNNNNNNNNNNNNNNNNNNNNNNNNNNNNNNNNNNNNNNNNNNN
NNNNNNNNNNNNNNNNNNNNNNNNNNNNNNNNNNNNNNNNNNNNNNNNNNN
NNNNNNNNNNNNNNNNNNNNNNNNNNNNNNNNNNNNNNNNNNNNNNNNNNN
NNNNNNNNNNNNNNNNNNNNNNNNNNNNNNNNNNNNNNNNNNNNNNNNNNN
NNNNNNNNNNNNNNNNNNNNNNNNNNNNNNNNNTAACCGCGACGCTC-
GACGCGCGACAATATGACCTCGAGCCATCGTTACTGACCTCGACTCTCAGGCAAACCAT
CTCTGGCGAGCACGGCCTCGACAGCAATGGCGTGTATGCACCTCCTATTCCCTACCTTC
AGATCTCGTCCTCCCTGCCGGCTTGGCACTGACAATCATACAGTTACAACGGCAGCTCC
GAGCTCCAGCTCGAGCGCATGAACGTCTACTTCAACGAGGTAAGTCAACAGC--
CACGTCG-----TTAACCATCTG-CGGCACG--
ATTTCCTGCCGTCGCCGAGGCCTTGCTAACGCGTTATCGCCC-
AGGCCTCCGGCAACAAGTATGTCCCTCGCGCCGTCCTCGTCGATCTCGAGCCCGGTACC
ATGGACGCCGTC

>Diaporthe_chrysalidocarpi_SAUCC194_35

NNNNNNNNNNNNNNNNNNNNNNNNNNNNNNNN------
NNNNNNNNNNNNNNNNNNNNNNNNNNNNNNNNNNNNNNNNNNNNNNNNNNN
NNNNNNNNNNNNNNNNNNNNNNNNNNNNNNNNNNNNNNNNNNNNNNNNNNN
NNNNNNNNNNNNNNNNNNNNNNNNNNNNNNNNNNNNNNNNNNNNNNNNNNN
NNNNNNNNNNNNNNNNNNNNNNNNNNNNNNNNNNNNNNNNNNNNNNNNNNN
NNNNNNNNNNNNNNNNNNNNNNNNNNNNNNNNNNNNNNNNNNNNNNNNNNN
NNNNNNNNNNNNNNNNNNNNNNNNNNNNNNNNNNNNNNCGCGA-GCTC-
GACGCGCGAC-
ATATGACCTCGAGCCATCGTTACTGACCTCGACTCTCAGGCAAACCATCTCTGGCGAGC
ACGGCCTCGACAGCAATGGCGTGTATGCACCTCCTATTTCCTGCCTTCAGATCTCGTCCT
CCCTGCTGGCTTGGCACTGACAATCACACAGTTACAACGGCACTTCCGAGCTCCAGCTC
GAGCGCATGAACGTCTACTTCAATGAGGTAAGTCAACAGC--CATGTCG-----
TTGACCATCTG-CAGCACG--
GTTTGCTGCCGTCGCTGGGGCCTTGCTAACGCGTTATCGCAC-
AGGCCTCCGGCAACAAGTATGTCCCTCGCGCCGTCCTCGTCGATCTCGAGCCCGGTACT
ATGGACGCCGTC

>Diaporthe_delonicis_MFLU_16_1059

NNNNNNNNNNNNNNNNNNNNNNNNNNNNNNNN------
NNNNNNNNNNNNNNNNNNNNNNNNNNNNNNNNNNNNNNNNNNNNNNNNNNN
NNNNNNNNNNNNNNNNNNNNNNNNNNNNNNNNNNNNNNNNNNNNNNNNNNN
NNNNNNNNNNNNNNNNNNNNNNNNNNNNNNNNNNNNNNNNNNNNNNNNNNN
NNNNNNNNNNNNNNNNNNNNNNNNNNNNNNNNNNNNNNNNNNNNNNNNNNN
NNNNNNNNNNNNNNNNNNNNNNNNNNNNNNNNNNNNNNCCAAAACGGTGCTGCT
TTCTGGTGC------------------GTCCACCACTGCGACGCTC-

GACGCGYGACAATACGACCTCGAGCCATCTTTACTGACCTCGACTCTCAGGCAAACCAT
CTCTGGCGAGCACGGCCTCGACAGCAATGGCGTGTATGCACCTCCTATTTCCTGCCTTCA
GATCTCGTCCTCCCTGCCGGCTTGGCACTGACAATCATACAGTTACAACGGCACTTCCG
AGCTCCAGCTCGAGCGCATGAACGTCTACTTCAACGAGGTCAGTAAACAAT--
CAAGTCG-----TTGACCATCTG-CAGCACG--
TTTTTCTGCCGTCGCCGGGCCTTTGCTAACGCGTTATCGCCC-
AGGCCTCCGGCAACAAGTATGTCCCTCGCGCCGTCCTCGTCGATCTCGAGCCCGGTACC
ATGGACGCCGTC

>Diaporthe_endocitricola_ZHKUCC20_0012

NNNNNNNNNNNNNNNNNNNNNNNNNNNNNNNNNNNNNNNNNNNNNNNNNNNNNNNN
NNNNNNNNNNNNNNNNNNNNNNNNNNNNNNNNNNNNNNNNNNNNNNNNNNNNNNNN
NNNNNNNNNNNNNNNNNNNNNNNNNNNNNNNNNNNNNNNNNNNNNNNNNNNNNNNN
NNNNNNNNNNNNNNNNNNNNNNNNNNNNNNNNNNNNNNNNNNNNNNNNNNNNNNNN
NNNNNNNNNNNNNNNNNNNNNNNNNNNNNNNNNNNNNNNNNNNNNNNNNNNNNNNN
NNNNNNNNNNNNNNNNNNNNNGGTAACCAAATCGGTGCTGCTTTCTGGTGC----------------
---GTCCACCACCGCGACGCTC-
GACGCGCGACAATATGACCTCGAGCCATCGTTACTGACCTCGACTCTCAGGCAAACCAT
CTCTGGCGAGCACGGCCTCGACAGCAATGGCGTGTATGCACCTCCTATTCCCTACCTTC
AGATCTCGTCCTCCCTGCCGGCTTGGCACTGACAATCATACAGTTACAACGGCACTTCC
GAGCTCCAGCTCGAGCGCATGAACGTCTACTTCAACGAGGTAAGTCAACGGC--
CACGTCG-----CTGACCATCTG-CAGCACG--
GTTTGCTGCCGTCGCTGGGGCCTTGCTAACGCGTTATCGCAC-
AGGCCTCCGGCAACAAGTATGTCCCTCGCGCCGTCCTCGTCGATCTCGAGCCCGGTACC
ATGGACGCCGTC

>Diaporthe_eres_CBS_138594

NNNNNNNNNNNNNNNNNNNNNNNNNNNNNNNNNNNNNNNNNNNNNNNNNNNNNNNN
NNNNNNNNNNNNNNNNNNNNNNNNNNNNNNNNNNNNNNNNNNNNNNNNNNNNNNNN
NNNNNNNNNNNNNNNNNNNNNNNNNNNNNNNNNNNNNNNNNNNNNNNNNNNNNNNN
NNNNNNNNNNNNNNNNNNNNNNNNNNNNNNNNNNNNNNNNNNNNNNNNNNNNNNNN
NNNNNNNNNNNNNNNNNNNNNNNNNNNNNNNNNNNNNNNNNNNNNNNNNNNNNNNN
NNNNNNNNNNNNNNNNNNNNNNNNNNNNNNNNNNGGTGCTGCTTTCTGGTGCGTTCCA
GCTCGAGCTCCAAGTCCACCGCCGCGACGCTT-
GACACGCGACAATACGACCTCGAAGCATCGTTGCTGACCTCGACTTTTAGGCAAACCAT
CTCTGGCGAGCACGGCCTCGACAGCAATGGCGTGTATGCACCTCCTATGCCCTGTCCAC
TGATCTTGACCTCTCTTCCGGCTTGGCACTGACAATCGCACAGTTACAACGGCACTTCTG
AGCTCCAGCTCGAGCGCATGAACGTCTACTTCAACGAGGCAAGTCAATAACAGCACAA
CATTCATCCGACCATCTC-CAACACG--
GTTTACTGCCGTCGCCCGAAGTTCGCTAACGCGTTATCGCCC-

AGGCCTCCGGCAACAAGTATGTTCCTCGCGCCGTCCTCGTCGATCTCGAGCCCGGTACC
ATGGACGCCGTC

>Diaporthe_fujianensis_JZB320149

NNNNNNNNNNNNNNNNNNNNNNNNNNNNNNNNNNNNNNNNNNNNNNNNNNNNN
NNNNNNNNNNNNNNNNNNNNNNNNNNNNNNNNNNNNNNNNNNNNNNNNNNNNN
NNNNNNNNNNNNNNNNNNNNNNNNNNNNNNNNNNNNNNNNNNNNNNNNNNNNN
NNNNNNNNNNNNNNNNNNNNNNNNNNNNNNNNNNNNNNNNNNNNNNNNNNNNN
NNNNNNNNNNNNNNNNNNNNNNNNNNNNNNNNNNNNNNNNNNNNNNNNNNNNN
NNNNNNNNNNNNNNNNNNNNNNNNNNNNNNNNNNNNNNNNNNNNNNNNNNCAA------
-------------GTCCACCACCGCGACGCTC-
GACGCGCGACGATACGACCTCGAACCATCGTTACTGACCTTGACTCTCAGGCAAACCAT
CTCTGGCGAGCACGGCCTCGACAGCAATGGCGTGTATGCACCTCCTATTCCCTACCTCC
AGATCTCGTCCTCCCTGCCGGCTTGGCACTGACAATCACACAGTTACAACGGCAGTTCC
GAGCTCCAGCTCGAGCGCATGAACGTCTACTTCAACGAGGTAAGTCAACATC--
CACGTCG-----TTGACCATCTA-CAGCACG--
GTTTCCTGCCGTCGCCGGGGCCTTGCTAACGCGTTATCGCCC-
AGGCCTCCGGCAACAAGTATGTTCCTCGCGCCGTCCTTGTCGATCTCGAGCCCGGTACC
ATGGACGCCGTC

>Diaporthe_fulvicolor_PSCG_051

NNNNNNNNNNNNNNNNNNNNNNNNNNNNNNNNNNNNNNNNNNNNNNNNNNNNN
NNNNNNNNNNNNNNNNNNNNNNNNNNNNNNNNNNNNNNNNNNNNNNNNNNNNN
NNNNNNNNNNNNNNNNNNNNNNNNNNNNNNNNNNNNNNNNNNNNNNNNNNNNN
NNNNNNNNNNNNNNNNNNNNNNNNNNNNNNNNNNNNNNNNNNNNNNNNNNNNN
NNNNNNNNNNNNNNNNNNNNNNNNNNNNNNNNNNNNNNNNNNNNNNNNNNNNN
NNNNNNNNNNNNNNNNNNNNNNNNNNNNNNNNNNNNNNNNNNNNNNNNNNNA-----
--------------GGGGGCTAACGCGACGCTC-GACGCGCGAC-
ATATGACCTCGAGCCATCGTTACTGACCTCGACTCTCAGGCAAACCATCTCTGGCGAGC
ACGGCCTCGACAGCAATGGCGTGTATGCACCTCCTATTCCCTACCTCCAGATCTCGTCCT
CCCTGCCGGCTTGGCATTGACAATCACACAGTTACAACGGCACTTCCGAGCTCCAGCTC
GAGCGCATGAACGTCTACTTCAACGAGGTAAGTAAACTGC--CACGTCG-----
TTGGCCATCTG-CAGCACG--GTTTGCTGCCGTCGCC-
AGGCTTTGCTAACGTGTTATCGCAC-
AGGCCTCCGGCAACAAGTATGTCCCTCGCGCCGTCCTCGTCGATCTCGAGCCCGGTACC
ATGGACGCCGTC

>Diaporthe_fusiformis_JZB320156

NNNNNNNNNNNNNNNNNNNNNNNNNNNNNNNNNNNNNNNNNNNNNNNNNNNNN
NNNNNNNNNNNNNNNNNNNNNNNNNNNNNNNNNNNNNNNNNNNNNNNNNNNNN

NNNNNNNNNNNNNNNNNNNNNNNNNNNNNNNNNNNNNNNNNNNNNNNNNNN
NNNNNNNNNNNNNNNNNNNNNNNNNNNNNNNNNNNNNNNNNNNNNNNNNNN
NNNNNNNNNNNNNNNNNNNNNNNNNNNNNNNNNNNNNNNNNNNNNNNNNNN
NNNNNNNNNNNNNNNNNNNNNNNNNNNNNNNNNNNNNNNNNNNNNNNCA------
-------------GTCCACCACCGCGACGCTC-
GACGCGCGACAATACGACCTCGAGCCATCGTTACTGACATCGACTCTCAGGCAAACCA
TCTCTGGCGAGCACGGCCTCGACAGCAATGGCGTGTATGCACCTCCTATTCCCTACTCTC
ATATCTTGTCCTCCCTGCCGGCTTGGCACTGACAATCACACAGTTACAACGGCTCTTCCG
AGCTCCAGCTCGAGCGCATGAACGTCTACTTCAACGAGGTAAGTCAACAGC--
CACGTCG-----TTGACCATCTG-CAGCACG--
GTTTCCTGCCGTCGCCAAGGCCTTGCTAACGCGTTATCGCCC-
AGGCCTCCGGCAACAAGTATGTCCCTCGCGCCGTCCTCGTCGATCTCGAGCCCGGTACC
ATGGACGCCGTC

>Diaporthe_guangxiensis_JZB320094

NNNNNNNNNNNNNNNNNNNNNNNNNNNNNNNNNNNNNNNNNNNNNNNNNNN
NNNNNNNNNNNNNNNNNNNNNNNNNNNNNNNNNNNNNNNNNNNNNNNNNNN
NNNNNNNNNNNNNNNNNNNNNNNNNNNNNNNNNNNNNNNNNNNNNNNNNNN
NNNNNNNNNNNNNNNNNNNNNNNNNNNNNNNNNNNNNNNNNNNNNNNNNNN
NNNNNNNNNNNNNNNNNNNNNNNNNNNNNNNNNNNNNNNNNNNNNNNNNNN
NNNNNNNNNNNNNNNNNNNNNNNNNNNNNNNGGTGCTGCTTTCTGGTGCGTTCCA
GCTCGAGCTCCAAGTCCACCACCGCGACGCTC-
GACGCGCGACAACATGACCTCGAGCCATCGTTACTGACCTCGACTCTCAGGCAAACCA
TCTCTGGCGAGCACGGCCTCGACAGCAATGGCGTGTATGCACCTCCTATTTCCTGCCTTC
AGATCTCGTCCTCCCTGCCGGCTTGGCACTGACAATCACACAGTTACAACGGCACTTCC
GAGCTCCAGCTCGAGCGCATGAACGTCTACTTCAATGAGGTAAGTCAACAGC--
CACGTCA-----TTGACCATCTG-CAGCACG--
GTTTGCTGCCGTCGCTGGGGCCTTGCTAACGCGTTATCGCAC-
AGGCCTCCGGCAACAAGTATGTCCCTCGCGCCGTCCTCGTCGATCTCGAGCCCGGTACT
ATGGACGCCGTC

>Diaporthe_huangshanensis_CNUCC_201903

NNNNNNNNNNNNNNNNNNNNNNNNNNNNNNNNNNNNNNNNNNNNNNNNNNN
NNNNNNNNNNNNNNNNNNNNNNNNNNNNNNNNNNNNNNNNNNNNNNNNNNN
NNNNNNNNNNNNNNNNNNNNNNNNNNNNNNNNNNNNNNNNNNNNNNNNNNN
NNNNNNNNNNNNNNNNNNNNNNNNNNNNNNNNNNNNNNNNNNNNNNNNNNN
NNNNNNNNNNNNNNNNNNNNNNNNNNNNNNNNNNNNNNNNNNNNNNNNNNN
NNNNNNNNNNNNNNNNNNNNNNNNNNNNNNNNNNNNNNNNNNNNNNNNNNN
NNNNNNNNNNNNNNNNNNNNNNNTAACGCGACGCTC-GACGCGCGAC-
ATATGACCTCGAGCCATCGTTACTGACCTCGACTCTCAGGCAAACCATCTCTGGCGAGC

ACGGCCTCGACAGCAATGGCGTGTATGCACCTCCTATTCCCTACCTTCAGATCTCGTCCT
CCCTGCCGGCTTGGCACTGACAATCATACAGTTACAACGGCACTTCCGAGCTCCAGCTC
GAGCGCATGAACGTCTACTTCAACGAGGTAAGTAAACTGC--CACGTCG-----
TTGGCCATCTG-CAGCACG--
GTTTGCTGCCGTCGCCAAGGCTTTGCTAACGCGTTATCGCAC-
AGGCCTCCGGCAACAAGTATGTCCCTCGCGCCGTCCTCGTCGATCTCGAGCCCGGTACT
ATGGACGCCGTC

>Diaporthe_eugeniae_CBS_444_82

GTCGGCCCATGCTGC-TTTCGCATCCTCTGCC------CCTGAGCCTGAGCCTA-
CCCCACCATCGCGACCG--
CACCCACGGTCGGGCCTCAAAAACATCACCAAACACCCTGGGAAAA-
GCACCCAGATGCCCTTGAAAAACGCGTCAGATTGCTAACGTGACC-
TTTTCCTCGACTACAGGTTCACCTTCAGACCGGCCAATGCGTAAGTTGCTCCTGTCA--
ACACCGTCGGACCTTATCAACGCCACCTGTAGCTGACACGTTTCCCAGGGTAACCAAAT
CGGTGCTGCTTTCTGGTGC-------------------GTCCACTACCGCGACGCTC-
GACGCGCGACAATACGATCTCGAACCCTCGTTACTGACCTCGACTCTCAGGCAAACCAT
CTCTGGCGAGCACGGCCTCGACAGCAATGGCGTGTATGCACCTCCTATTCCCTACCTCC
AGATCTCGTCCTCCCTGCCGGCTTGGCACTGACAATCATACAGTTACAACGGCACTTCC
GAGCTCCAGCTCGAGCGCATGAACGTCTACTTCAACGAGGTAAGTAAACAGC--
CACGTCG-----TTGACCATGTG-CAGCACG--ATTTTCT---
GTCGCCGGGGCCTTGCTAACGCGTTATCGCAC-
AGGCCTCCGGCAACAAGTATGTCCCTCGCGCCGTCCTCGTCGATCTTGAGCCCGGTACC
ATGGACGCCGTC

>Diaporthe_gossiae_BRIP_59730a

GTCGGCCCATGCTGC-TTTCGCATCCTCTGCC------CCTGAGCCTGAGCCTA-
CCCCACCATCGCGACCG--
CACCCACGGTCGGGCCTCAAAAACATCACCAAACACCCTGGGAAGA-
GCACCCAGATGCCCTTGAAAAACGCGTCAGATTGCTAACGTGGTC-
TTTTTCTCGACTACAGGTTCACCTTCAGACCGGCCAATGCGTAAGTTGCTCCTGTCA--
ACACCGACGGACCTTATCATCGCCACCCGTAGCTGACACGTTTCCCAGGGTAACCAAAT
CGGTGCTGCTTTCTGGTGC-------------------GTCCACCACCGCGACGCTC-
GACGCGCGATAATACGACCTCGAACCATCGTTACTGACCTCGACTCTCAGGCAAACCAT
CTCTGGCGAGCACGGCCTCGACAGCAATGGCGTGTATGCACCTCCTATTCCCTACATCC
AGAACTCGTCCTCCCTGCCGGCTTGGCACTGACAATCACATAGTTACAACGGCACTTCC
GAGCTCCAGCTCGAGCGCATGAACGTCTACTTCAACGAGGTAAGTCGACAGC--
CACGTCG-----TTGGCCATCTG-CAGCACG--
GTTTGCTGCCGTCGCTGGGGCCTTGCTAACGCGTTATCGCAC-

AGGCCTCCGGCAACAAGTATGTCCCTCGCGCCGTCCTCGTCGATCTCGAGCCCGGTACC
ATGGACGCCGTC

>Diaporthe_hongkongensis_CBS_115448

GCCGGCCCATGCTGC-TTTCGCATCCTCTGCC------CCTGAGCCTGAGCCTA-
CCCCACCATCGCGACCG--
CACCCACGGTCGGGCCTCAAAAACATCACCAAACACCCTGGGAAAC-
GCACCCAGATGCCCTTGAAAAACGCGTCAGATTGCTAACGTGAAC-
TTTTTCTCGACTACAGGTTCACCTTCAGACCGGCCAATGCGTAAGTTGCTCCTGTCA--
ACACCGCCAGACCTTATCATCGCCACCCGTAGCTGACACGTTTCCCAGGGTAACCAAAT
CGGTGCTGCTTTCTGGTGCGTCCCAGCTCCGACTTCAAGTCCACCACCGCGACGCTC-
GACGCGCGACAATACGACCTCGAGCCATCGTTACTGACATCGACTCTCAGGCAAACCA
TCTCTGGCGAGCACGGCCTCGACAGCAATGGCGTGTATGCACCTCCTATTCCCTACTCTC
ATATCTTGTCCTCCCTGCCGGCTTGGCACTGACAATCACACAGTTACAACGGCTCTTCCG
AGCTCCAGCTCGAGCGCATGAACGTCTACTTCAACGAGGTAAGTCAACAGC--
CACGTCG-----TTGACCATCTG-CAGCACG--
GTTTCCTGCCATCGCCAAGGCCTTGCTAACGCGTTATCGCCC-
AGGCCTCCGGCAACAAGTATGTCCCTCGCGCCGTCCTCGTCGATCTCGAGCCCGGTACC
ATGGACGCCGTC

>Diaporthe_howardiae_BRIP_59697a

GGCGGCCCATGGTGC-TTTCGCATCCTCTGCC------CCTGAGCCTGAGCCTA-
CCCCACCATCGCGACCG--
CACCCACGGTCGGGCCTCAAAAACATCACCAAACACCCTGGGAAAA-
GCACCCAGATGCCCTTGAAAAACGCGTCAGATTGCTAACGTGACC-
TTTTTCTTGACTACAGGTTCACCTTCAGACCGGCCAATGCGTAAGTTGCCCCTGTCA--
ACACCGCCAGACCTTATCATCGCCACCCATAGCTGACACGTTTCCCAGGGTAACCAAAT
CGGTGCTGCCTTCTGGTGC-------------------GTCCACCACCGCGACGCTC-
GACGCGCGACAATACGACCTCGAACCATCGTTACTGACCTCGACTCTCAGGCAAACCA
TCTCTGGCGAGCACGGCCTCGACAGCAATGGCGTGTATGCACCTCCTATTTCCTACCTCC
AGATCT-
GTCCTCCCTGCCGGCTTGGCACTGACAATCATACAGTTACAACGGCACTTCCGAGCTCC
AGCTCGAGCGCATGAACGTCTACTTCAACGAGGTAAGTCAACAGC--CACGTCG-----
TTGGCCATCTG-
CAGCACGCAATTTGCTGCCGTCGCTGGGGCCTTGCTAACGCGTTATCGCAC-
AGGCCTCCGGCAACAAGTATGTCCCTCGCGCCGTCCTCGTCGATCTCGAGCCCGGTACC
ATGGACGCCGNN

>Diaporthe_hunanensis_HNZZ023

NNNNNNNNNNNNNNNNNNNNNNNNNNNNNNNNNNNNNNNNNNNNNNNNNNN
NNNNNNNNNNNNNNNNNNNNNNNNNNNNNNNNNNNNNNNNNNNNNNNNNNN
NNNNNNNNNNNNNNNNNNNNNNNNNNNNNNNNNNNNNNNNNNNNNNNNNNN
NNNNNNNNNNNNNNNNNNNNNNNNNNNNNNNNNNNNNNNNNNNNNNNNNNN
NNNNNNNNNNNNNNNNNNNNNNNNNNNNNNNNNNNNNNNNNNNNNNNNNNN
NNNNNNNNNNNNNNNNNNNNNNNNNNNNNNNNNTGCTGCTTTCTGGTGC-----------
-------GTCCACCACCGCGACGCTC-
GACGCGCGACAACATGACCTCGAGCCATCGTTACTGACCTCGACTCTCAGGCAAACCA
TCTCTGGCGAGCACGGCCTCGACAGCAATGGCGTGTATGCACCTCCTATTTCCTGCCTTC
AGATCTCGTCCTCCCTGCCGGCTTGGCACTGACAATCACACAGTTACAACGGCACTTCC
GAGCTCCAGCTCGAGCGCATGAACGTCTACTTCAATGAGGTAAGTCAACAGC--
CACGTCA-----TTGACCATCTG-CAGCACG--
GTTTGCTGCCGTCGCTGGGGCCTTGCTAACGCGTTATCGCAC-
AGGCCTCCGGCAACAAGTATGTCCCTCGCGCCGTCCTCGTCGATCTCGAGCCCGGTACT
ATGGACGCCGTN

>Diaporthe_krabiensis_MFLUCC_17_2481

NNNNNNNNNNNNNNNNNNNNNNNNNNNNNNNNNNNNNNNNNNNNNNNNNNN
NNNNNNNNNNNNNNNNNNNNNNNNNNNNNNNNNNNNNNNNNNNNNNNNNNN
NNNNNNNNNNNNNNNNNNNNNNNNNNNNNNNNNNNNNNNNNNNNNNNNNNN
NNNNNNNNNNNNNNNNNNNNNNNNNNNNNNNNNNNNNNNNNNNNNNNNNNN
NNNNNNNNNNNNNNNNNNNNNNNNNNNNNNNNNNNNNNNNNNNNNNNNNNN
NNNNNNNNNNNNNNNNNNNNNNNNNNNNNNNNNNNNNNNNNNNNNNNNN-----
--------------TGCCGTACACGCGACGCTC-GACGCGCGAC-ATATGACCTCGAA-
CATAATTACTGACATCGATTCTCAGGCAAACCATCTCTGGCGAGCACGGCCTCGACAGC
AATGGCGTGTATGCACCTCCTATTCCCTGCCTCCAGATCTCGTCCTCCCTGCCGGCTTGG
CACTGACAATCACACAGGTACAACGGCACTTCCGAGCTCCAGCTCGAGCGCATGAACG
TCTACTTCAACGAGGTAAGTCAACAGC--CACGTCG-----TTGACCATCTG-CAGCACG--
ATCTTCTGCCGTCGCCGGGGCTTTGCTAACGCGTTATCGCAC-
AGGCCTCCGGCAACAAGTATGTCCCTCGCGCCGTCCTCGTCGATCTCGAGCCCGGTACC
ATGGACGCCGTC

>Diaporthe_lithocarpi_CGMCC_3_15175

NNNNNNNNNNNNNNNNNNNNNNNNNNNNNNNNNNNNNNNNNNNNNNNNNNN
NNNNNNNNNNNNNNNNNNNNNNNNNNNNNNNNNNNNNNNNNNNNNNNNNNN
NNNNNNNNNNNNNNNNNNNNNNNNNNNNNNNNNNNNNNNNNNNNNNNNNNN
NNNNNNNNNNNNNNNNNNNNNNNNNNNNNNNNNNNNNNNNNNNNNNNNNNN
NNNNNNNNNNNNNNNNNNNNNNNNNNNNNNNNNNNNNNNNNNNNNNNNNNN
NNNNNNNNNNNNNNNNNNNNNNNNNNNNNNNNNTGCTGCTTTCTGGTGCGTCCC
AGCTCCGGCTCAAAGTCCACCACCGCGACGCTC-

GACGCGCGACAATACGACCTCGAACCATCGTTACTGACCTCGACTCTCAGGCAAACCA
TCTCTGGCGAGCACGGCCTCGACAGCAATGGCGTGTATGCACCTCCTATTCCCTACTCTC
ATATCTTGTCCTCCCTGCCGGCTTGGCACTGACAATCACACAGTTACAACGGCTCTTCCG
AGCTCCAGCTCGAGCGCATGAACGTCTACTTCAACGAGGTAAGTCAACAGC--
CACGTCG-----TTGACCATCTG-CAGCACG--
GTTTCCTGCCGTCGCCAAGGCCTTGCTAACGCGCCATCGCCC-
AGGCCTCCGGCAACAAGTATGTCCCTCGCGCCGTCCTCGTCGATCTCGAGCCCGGTACC
ATGGACGCCGTC

>Diaporthe_limonicola_CBS_142549

NNNNNNNNNNNNNNNNNNNNNNNNNNNNCACT------GTAGAGGTAACATCTA-
CCCCACCATCGCGACCG--
CACCCGCGGTCGGGCCTCAAAAACATCACCAAACACCCTTGGAAAA-
GCACCCAGATGCCCTTGAAAAACGCGTCAGATTGCTAACGTGACC-
TTTTTCTTGACTACAGGTTCACCTTCAGACCGGCCAATGCGTAAGTTGTCCCTGTCA--
ACACCGCCGGACCTTATCATCGCCACCCGTAGCTGACACGTTTCCCAGGGTAACCAAAT
CGGTGCTGCTTTCTGGTGC------------------GTCCACCGCCGCGACGCTC-
GACGCGCGACAATACGACCTCGAACCATCGTTACTGACCTCGACTCTCAGGCAAACCA
TCTCTGGCGAGCACGGCCTCGACAGCAATGGCGTGTATGCACCTCCTATTCCCTACCTTG
AGATCTCGTCCTCCCTGCCGGCTTGGCACTGACAATCACACAGTTACAACGGCACTTCC
GAGCTCCAGCTCGAGCGCATGAACGTCTACTTCAACGAGGTAAGTCAACAGC--
CACGTCG-----TTGGCCATCTG-CAGCACG--GTTTGCTGCCGTCGCC-
AGGCTTTGCTAACGCGTTATCGCAC-
AGGCCTCCGGCAACAAGTATGTCCCTCGCGCCGTCCTCGTCGATCTCGAGCCCGGTANN
NNNNNNNNNNNN

>Diaporthe_litchicola_BRIP_54900

NNNNNNNNNNNCTGC-TTTCGCATCCTCTGCC------CCTGAGCCTGAGCCTA-
CCCCACCATCGCGAACG--
CACCCACGGTCGGGCCTCAAAAACATCACCAAACACCCTGGGAAAAGGCACCCAGAT
GCCCTTGAAAAACGCGTCAGATTGCTAACGTGAGC-
TTTTTCTCGACTACAGGTTCACCTTCAGACCGGCCAATGCGTAAGTTGCTCCTGTCA--
ACATCGCCGGACCTTATCATCGCCACCCGTAGCTGACACGTTTCCCAGGGTAACCAAAT
CGGTGCTGCTTTCTGGTGC------------------GTCCACCACCGCGACGCTC-
GACGCGCGACAATATGACCTCGAAACATCGTTACTGACCTCGACTCTCAGGCAAACCA
TCTCTGGCGAGCACGGCCTCGACAGCAATGGCGTGTATGCACCTCCTATTCCCTACCTTC
AGATCTCGTCCTCCCTGCCGACTTGGCACTGACAATCATACAGTTACAACGGCACTTCC
GAGCTCCAGCTCGAGCGCATGAACGTCTACTTCAACGAGGTAAGTCAACAGC--
CACGTCG-----TTCACCATCTG-CAGCACG--
GTTTGCTGCCGTCGCTGGGGCCTTGCTAACGCGTTATCGCAC-

AGGCCTCCGGCAACAAGTATGTCCCTCGCGCCGTCCTCGTCGATCTCGAGCCCGGTACC
ATGGACGCCGTC

>Diaporthe_meliae_CFCC_53089

NNNNGCCCATGCTGC-TTTCGCATCCTCTGCC------CCTGAGCCTGAGCCTA-
CCCCACCATCGCGACCG--
CACCCACGGTCGGGCCTCAAAAACATCACCAAACACCCTTGGAAAA-
GCACCCAGATGCCCTTGAAAAACGCATCAGATTGCTAATGTGAAC-
TTTTCCCCGACTACAGGTTCACCTTCAGACCGGCCAATGCGTAAGTTGCTCCTTTCA--
ACACCGCCGGATCTTATCATCGCCACCCGTAGCTGACACGTTTCCCAGGGTAACCAAAT
CGGTGCTGCTTTCTGGTGC-------------------GTCCACCACCGCGACGCTC-
GACGCGCGACAATATGACCTCGAGCCATCGTTATTGACCTCGACTCTCAGGCAAACCAT
CTCTGGCGAGCACGGCCTCGACAGCAATGGCGTGTATGCACCTCCTATTCCCTACCTTC
AGATCTCGTCCTCCCTGCCGGCTTGGCACTGACAATCACACAGTTACAACGGCACCTCC
GAGCTCCAGCTCGAGCGCATGAACGTCTACTTCAACGAGGTAAGTAAACTGC--
CACGTCG-----TTGGCCATCTG-CAGTACG--GTTTGCTGCCGTCGCC-
AGGCTTTGCTAACGCGTTATCGCAC-
AGGCCTCCGGCAACAAGTATGTCCCTCGCGCCGTCCTCGTCGATCTCGAGCCCGGTACC
ATGGACGCCGTN

>Diaporthe_millettiae_GUCC9167

GTCGGCCCATGCTGC-TTTCGCATCCTCTGCCCCTGAGCCTGAGCCTGAGCCTA-
CCCCACCATCGCGACCG--
CACCCACGGTCGGGCCTCAAAAACATCACCAAACACCCTGGGAAAA-
GCACCCGAATGCCTTTGAAAAACGCGTCAGATTGCTAACGTGACC-
TTTTTCTCGACTACAGGTTCACCTTCAGACCGGCCAATGCGTAAGTTGCTCCTGTCA--
ACACCGCCGGACCTTATCATCGCCACCCGTAGCTGACACGTTTCCCAGGGTAACCAAAT
CGGTGCTGCTTTCTGGTGC-------------------GTCCACCACCGCGACGCTC-
GACGCGCGACAATACAACCTCGAACCATCGTTGCTGACCTCGACTCTCAGGCAAACCA
TCTCTGGCGAGCACGGCCTCGACAGCAATGGCGTGTATGCACCTCCTATTTCCTACCTCC
AGATCTCGTCCTCCCTGCCGGCTTGGCACTGACAATCATACAGTTACAACGGCACTTCC
GAGCTCCAGCTCGAGCGCATGAACGTCTACTTCAACGAGGTAAGTAAACAGC--
CACGTCG-----TTGGCCATCTG-CAGCACG--
GTTTGTTGCCGTCGCCAAGGCTTTGCTAACGCGTTATCGCCC-
AGGCCTCCGGCAACAAGTATGTCCCTCGCGCCGTCCTCGTCGATCTCGAGCCCGGTACC
ATGGACGCCGTC

>Diaporthe_musigena_CBS_129519

GTCGGCCCATGCTGC-TTTCGCATCCTCTGCC------CCTGAGCCTGAGCCTA-
CCCCACCATCGCGACCG--

CACCCACGGTCGGGCCTCAAAAACATCACCAAACACCCTGGAAAAA-
GCACCCGAATGCCCTTGAAAAACGCGTCAGATTGCTAACGTGAAC-
CTTTTCTCGACTACAGGTTCACCTTCAGACCGGCCAATGCGTAAGTTGCTCCTGTCA--
ACACCGCCGGACCTTATCATCGCCACCTGTAGCTGACACGTTTCCCAGGGTAACCAAAT
CGGTGCTGCTTTCTGGTGC-------------------GTCCACCACCGCGACGCTC-
GACGCGCGACTATACGACCTCGAAACATCGTTACTGACCTCGACTCTTAGGCAAACCAT
CTCTGGCGAGCACGGCCTCGACAGCAATGGCGTGTATGCACCTCCTATTTCCTGCCTCC
AGATCTCGTCCTCCCTGCCGGCTTGGCACTGACAATCACACAGTTACAACGGCACTTCC
GAGCTCCAGCTCGAGCGCATGAACGTCTACTTCAACGAGGTAAGTCAACAGC--
CACGTCG-----TTGACCATCTG-CAGCACG--
GTTTCCTGCCGTCGCCAAGGCTTTGCTAATGCGTTATCGCCC-
AGGCCTCCGGCAACAAGTATGTCCCTCGCGCCGTCCTCGTCGATCTCGAGCCCGGTACC
ATGGACGCCGTC

>Diaporthe_norfolkensis_BRIP_59718a

GTCGGCCCATGCTGC-TTTCGCATCCTCTGCC------CCTGAGCCTGAGCCTA-
CCCCACCATCGCGACCG--
CACCCACGGTCGGACCTCAAAAACATCACCAAACACCCTGGGAAAG-
GCACCCAGATGCCCTTGAAAAACGCGTCAGATTGCTAATGTGAAC-
TTTTTCTCGACCACAGGTTCACCTTCAGACCGGCCAATGCGTAAGTTGCTCCTGTCA--
ACACCGACGGACCTTATCATCGCCACCCGTAGCTGACACGTTTCCCAGGGTAACCAAAT
CGGTGCTGCTTTCTGGTGC-------------------GTCCACCACCGCGACGCTC-
GACGCGCGACAATACGACCTCGAAACATCGTTACTGACCTCGACTCTCAGGCAAACCA
TCTCTGGCGAGCACGGCCTCGACAGCAATGGCGTGTATGCACCTCCTATTCCCTACCTC
CAGAACTCGTCCTCCCTGCCGGCTTGGCACTGACAATCACACAGTTACAACGGCACTTC
CGAGCTCCAGCTCGAGCGCATGAACGTCTACTTCAACGAGGTAAGTCAACAGC--
CACGTCG-----TTGGCCATCTG-CAGCACG--
GTTTGCTGCCGTCGCTGGGGCCCTGCTAACGCGTTATCGCAC-
AGGCCTCCGGCAACAAGTATGTCCCTCGCGCCGTCCTCGTCGATCTCGAGCCCGGTACC
ATGGACGCCGNN

>Diaporthe_oculi_HHUF_30565

GTCGGCCCATGCTGC-TTTCGCATCCTCTGCC------
CCTGAGCCTGAGCCTACCCCCACCATCGCGACCG--
CACCCACGGTCGGGCCTCAAAAACATCACCAAACACCCTGGGAAAA-
GCACCCAGATGCCCTTGAAAAACGCGTCAGATTGCTAACGTGACC-
TTTTTCTCGACTACAGGTTCACCTTCAGACCGGCCAATGCGTAAGTTGCTCCTGTCA--
ACACCGCCGGACCTTATCATCGCCACCTGTAGCTGACACGTTTCCCAGGGTAACCAAAT
CGGTGCTGCTTTCTGGTGC-------------------GTCCACCACCGCGACGCTC-
GACGCGCGACAATATGACCTCGAGCCATCGTTACTGACCTCGACTCTCAGGCAAACCAT

CTCTGGCGAGCACGGCCTCGACAGCAATGGCGTGTATGCACCTCCTATTCCCTACCTCC
AGATCTCGTCCTCCCTGCCGGCTTGGCATTGACAATCACACAGTTACAACGGCACTTCC
GAGCTCCAGCTCGAGCGCATGAACGTCTACTTCAACGAGGTAAGTCAACAGC--
CACGTCG-----TTGGCCATCTG-CAGCACG--GTTTGCTGCCGTCGCC-
AGGCTTTGCTAACGTGTTATCGCAC-
AGGCCTCCGGCAACAAGTATGTCCCTCGCGCCGTCCTCGTCGATCTCGAGCCCGGTACC
ATGGACGCCGTC

>Diaporthe_osmanthi_GUCC9165

GTCGGCCCATGGTGC-TTTCGCATCCTCTGCC------CCTGAGCCTGAGCCTA-
CCCCACCATCGCGACCG--
CACCCACGGTCGGGCCTCAAAAACATCACCAAACACCCTGGGAAAA-
GCACCCAGATGCCCTTGAAAAACGCGTCAGATTGCTAACGTGAAC-
TTTTTCTCGACTACAGGTTCACCTTCAGACCGGCCAATGCGTAAGTTGCTCCTGTCA--
ACACCGACGGACCTTATCATCGCCACCCGTAGCTGACACGTTTCCCAGGGTAACCAAAT
CGGTGCTGCTTTCTGGTGC-------------------GTCCACCACCGCGACGCTC-
GACGCGCGACAATACGACCTCGAACCATCGTTACTGACCTCGACTCTCAGGCAAACCA
TCTCTGGCGAGCACGGCCTCGACAGCAATGGCGTGTATGCACCTCCTATTTCCTACCTCC
AGATCTCGTCCTCCCTGCCGGCTTGGCACTGACAATCATACAGTTACAACGGCACTTCC
GAGCTCCAGCTCGAGCGCATGAACGTCTACTTCAACGAGGTAAGTCAACAGC--
CACGTCG-----TTGACCATCTG-CAGCACG--
AATCTCTGCCGTCACCGGGGCCTTGCTAACGCGTTATCGCCC-
AGGCCTCCGGCAACAAGTATGTCCCTCGCGCCGTCCTCGTCGATCTCGAGCCCGGTACT
ATGGACGCCGTC

>Diaporthe_pascoei_BRIP_54847

GTCGGCCCATGCTGC-TTTCGCACCCTCTGCC------
CCTGAGCCTGAGCCTACCCCCACCATCGCGACCG--
CACCCACGGTCGGGCCTCAAAAACATCACCAAACACCCTGGGAAAA-
GCACCCAGATGCCCTTGAAAAACGCGTCAGATTGCTAACGTGAGC-
TTTTTCTCGACTACAGGTTCACCTTCAGACCGGCCAATGCGTAAGTTGCTCCTGTCA--
ACACCGACGGACCTTATCATCGCCACCTGTAGCTGACACGTTTCCCAGGGTAACCAAAT
CGGTGCTGCTTTCTGGTGC-------------------GTCCACCACCGCGGCGCTC-
GACGCGCGACAATACGAACTCGAACCATCGTTACTGACCTCAACTCTCAGGCAAACCA
TCTCTGGCGAGCACGGCCTCGACAGCAATGGCGTGTATGCACCTCCTATTCTCTACCTCC
AGATCTCGTCCTCCCTGCCGGCTTGGCACTGACAATCACACAGTTACAACGGCACTTCC
GAGCTTCAGCTCGAGCGCATGAACGTCTACTTCAACGAGGTAAGGAAACAGC--
CACGTCG-----TTGACCATCTG-CAGCACG--
GTTTCCTGCCGTCGCCAAGGCTTTGCTAACGCGTTATCGCCC-

AGGCCTCCGGCAACAAGTATGTCCCTCGCGCCGTCCTCGTCGATCTCGAGCCCGGTACC
ATGGACGCCGTC

>Diaporthe_perseae_CBS_151_73

GTCGGCCCATGCTGC-TTTCGCACCCTCTGCC------CCTGAGCCTGAGCCTA-
CCCCACCATCGCGACCG--CACCCACGGTCGGGCC-
CAAAAACATCACCAAACACCCTGGGAAAA-
GCACCCAAATGCCCTTGAAAAACGCGTCAGATTGCTAACGTGATC-
TTTTTCTTGACTACAGGTTCACCTTCAGACCGGCCAATGCGTAAGTTGCTCCTGTCA--
ACACCGCCGGACCTTATCATCGCCACCCGTAGCTGACACGTTTCCCAGGGTAACCAAAT
CGGTGCTGCTTTCTGGTGC-------------------GTCCACCGCCGCGACGCTC-
GACGCGCGACAATACGACCTCGAAACATCGTTACTGACCTCGACTCTCAGGCAAACCA
TCTCTGGCGAGCACGGCCTCGACAGCAATGGCGTGTATGCACCTCCTATTTCCTACCTCC
AGATCTCGTCCTCCCTGCCGGCTTGGCACTGACAATCATACAGTTACAACGGCACTTCC
GAGCTCCAGCTCGAGCGCATGAACGTCTACTTCAACGAGGTAAGTAAACAGC--
CACGTCG-----TTGGCCATCTG-CAGCACG--GTTTGCTGCCGTCGCC-
AGGCTTTGCTAACGCGTTATCGCAC-
AGGCCTCCGGCAACAAGTATGTCCCTCGCGCCGTCCTCGTCGATCTCGAGCCCGGTACC
ATGGACGCCGTC

>Diaporthe_pseudomangiferae_CBS_101339

GTCGGCCCATGCTGC-TTTCGCATCCTCTGCC------CCTGAGCCTGAGCCTA-
CCCCACCATCGCGACCG--
CACCCACGGTCGGGCCTCAAAAACATCACCAAACACCCTGGGAAAA-
GCACCCAGATGCCCTTGAAAAACGCGTCAGACTGCTAACATGAAC-
TTTTTCTCGACTACAGGTTCACCTTCAGACCGGCCAATGCGTAAGTTGCTCCTGTCA--
ACACCGACGGACCTTATCATCGCCACTCGTAGCTGACACGTTTCCCAGGGTAACCAAAT
CGGTGCTGCTTTCTGGTGC-------------------GTCCACCACCGCGACGCTC-
GACGCGCGACAATACGACCTTGAACCATCGTTACTGACCTCGACTCTCAGGCAAACCAT
CTCTGGCGAGCACGGCCTCGACAGCAATGGCGTGTATGCACCTCCTATCCCCTACCTCC
AGAACTCGTCCTCCCTGCCGGCTTGGCACTGACAATCATACAGTTACAACGGCACTTCC
GAGCTCCAGCTCGAGCGCATGAACGTCTACTTCAACGAGGTAAGTCAACAGC--
CACGTCG-----TTGGCCATCTG-CAGCACG--
GTTTGCTGCCGTCACTGGAGCCTTGCTAACGCGTTATCGCAC-
AGGCCTCCGGCAACAAGTATGTCCCTCGCGCCGTCCTCGTCGATCTCGAGCCCGGTACC
ATGGACGCCGTC

>Diaporthe_pseudooculi_HHUF_30617

NNNNNNCCATGCTGC-TTTCGCATCCTCTGCC------CCTGAGCCTGAGCCTA-
CCCCACCATCGCGACCG--

CACCCACGGTCGGGCCTCAAAAACATCACCAAACACCCTGGGAAAA-
GCACCCAAATGTCCTTGAAAAACGCGTCAGATTGCTAACGTGACC-
TTTTTCTCGACTACAGGTTCACCTTCAGACCGGCCAATGCGTAAGTTGCTCCTGTCA--
ACACCGACGGACCTTATCATCGCCACCCGTAGCTGACACGTTTCCCAGGGTAACCAAAT
CGGTGCTGCTTTCTGGTGC-------------------GTCCACCACCGCGACGCTC-
GACGCGCGACAACATGACCTCGAGCCATCGTTACTGACCTCGACTCTCAGGCAAACCA
TCTCTGGCGAGCACGGCCTCGACAGCAATGGCGTGTATGCACCTCCTATTTCCTGCCTTC
AGATCTCGTCCTCCCTGCCGGCTTGGCACTGACAATCACACAGTTACAACGGCACTTCC
GAGCTCCAGCTCGAGCGCATGAACGTCTACTTCAACGAGGTAAGTCAACAGC--
CACGTCG-----TTGATCATCTG-CAGCACG--
ATTTTCTGCCGTCGCCGGGGCCTTGCTAACGCGTTATCGCCC-
AGGCCTCCGGCAACAAGTATGTCCCTCGCGCCGTCCTCGTCGATCTCGAGCCCGGTACC
ATGGACGCCGTC

>Diaporthe_pseudophoenicicola_CBS_462_69

GTCGGCCCATGCTGC-TTTCGCATCCTCTGCC------CCTGAGCCTGAGCCTA-
CCCCACCATCGCGACCG--
CACCCACGGTCGGGCCTCAAAAACATCACCAAACACCCTTGGAAAA-
GCACACAGATGCCCTTG-
AAAACGCGTCAGATTGCTAACGTGACCTTTTTCTCGACTACAGGTTCACCTTCAGACCG
GCCAATGCGTAAGTTGCTCCTGTCA--
ACACCGCCGGACCTTATTATCGCCACTCGTAGCTGACACGTTTCCCAGGGTAACCAAAT
CGGTGCTGCTTTCTGGTGC-------------------GTCCACCGCCGCGACGCTC-
GACGCGCGACAATACGGCCTCGAACCATCTTTACTGACCTCGACTCTCAGGCAAACCAT
CTCTGGCGAGCACGGCCTCGACAGCAATGGCGTGTATGCACCTCCTATTCCCTGCCTTC
AGATCTCGTCCTCCCTGCCGGCTTGGCACTGACAATCATACAGTTACAACGGCACTTCC
GAGCTCCAGCTCGAGCGCATGAACGTCTACTTCAACGAGGTAAGTCAACAGC--
CACGTCG-----TTGGCCATCTG-CAGCACG--
GTTTGCTGCCGTCGCCGGGGCCTTGCTAACGCGTTATCGCAC-
AGGCCTCCGGCAACAAGTATGTCCCTCGCGCCGTCCTCGTCGATCTCGAGCCCGGTACC
ATGGACGCCGTC

>Diaporthe_rhodomyrti_CFCC_53101

NNNNGCCCATGCTGC-TTTCGCATCCTCTGCC------CCTGAGCCTGAGCCTA-
CCCCACCATCGCGACCG--
CACCCACGGTCGGGCCTCAAAAACATCACCAAACACCCTGGGAAAC-
GCACCCAGATGCCCTTGAAAAACGCGTCAGATTGCTAACGTGAAC-
TTTTTCTCGACTACAGGTTCACCTTCAGACCGGCCAATGCGTAAGTTGCTCCTGTCA--
ACACCGCCAGACCTTATCATCGCCACCCGTAGCTGACACGTTTCCCAGGGTAACCAAAT
CGGTGCTGCTTTCTGGTGCGTCCCAGCTCCGACTTCAAGTCCACCACCGCGACGCTC-

GACGCGCGACAATACGACCTCGAGCCATCGTTACTGACATCGACTCTCAGGCAAACCA
TCTCTGGCGAGCACGGCCTCGACAGCAATGGCGTGTATGCACCTCCTATTCCCTACTCTC
ATATCTTGTCCTCCCTGCCGGCTTGGCACTGACAATCACACAGTTACAACGGCTCTTCCG
AGCTCCAGCTCGAGCGCATGAACGTCTACTTCAACGAGGTAAGTCAACAGC--
AACGTCG-----TTGACCATCTG-CAGCACG--
GTTTCCTGCCGTCGCCAAGGCCTTGCTAACGCGTTATCGCCC-
AGGCCTCCGGCAACAAGTATGTCCCTCGCGCCGTCCTCGTCGATCTCGAGCCCGGTACC
ATGGACGCCGTN

>Diaporthe_schimae_CFCC_53103

NNNNGCCCATGCTGC-TTTCGCATCCTCTGCC------CCTGAGCCTGAGCCTA-
CCCCACCATCGCGACCG--
CACCCACGGTCGGGCCTCAAAAACATCACCAAACACCCTGGGAAAA-
GCACCCAAATGTCCTTGAAAAACGCGTCAGATTGCTAACGTGACC-
TTTTTCTCGACTACAGGTTCACCTTCAGACCGGCCAATGCGTAAGTTGCTCCTGTCA--
ACACCGACGGACCTTATCATCGCCACCCGTAGCTGACACGTTTCCCAGGGTAACCAAAT
CGGTGCTGCTTTCTGGTGC-------------------GTCCACCACCGCGACGCTC-
GACGCGCGACAACATGACCTCGAGCCATCGTTACTGACCTCGACTCTCAGGCAAACCA
TCTCTGGCGAGCACGGCCTCGACAGCAATGGCGTGTATGCACCTCCTATTTCCTGCCTTC
AGATCTCGTCCTCCCTGCCGGCTTGGCACTGACAATCACACAGTTACAACGGCACTTCC
GAGCTCCAGCTCGAGCGCATGAACGTCTACTTCAACGAGGTAAGTCAACAGC--
CACGTCG-----TTGATCATCTG-CAGCACG--
ATTTTCTGCCGTCGCCGGGGCCTTGCTAACGCGTTATCGCCC-
AGGCCTCCGGCAACAAGTATGTCCCTCGCGCCGTCCTCGTCGATCTCGAGCCCGGTACC
ATGGACGCCGTN

>Diaporthe_searlei_BRIP_66528

GTCGGCCCATGCTGC-TTTCGCATCCTCTGCC------CCTGAGCCTGAGCCTA-
CCCCACCATCACGACCG--
CACCCACGGTCGGGCCTCAAAAACATCACCAAACACCCTGGGAAAA-
GCACCCGAATGCCCTTGAAAAACGCGTCAGATTGCTAATGTGACC-
TTTTTCTCGACTACAGGTTCACCTTCAGACCGGCCAATGCGTAAGTTGCTCCTGTCA--
ACACCGCCGGACCTTATCATCGCCACCAGTAGCTGACACGTTTCCCAGGGTAACCAAAT
CGGTGCTGCTTTCTGGTGC-------------------GTCCACCACCGCGACGCTC-
GACGCGCGACAATACGACCTCGAACCATCGTTACTGACCTCGACTCTCAGGCAAACCA
TCTCTGGCGAGCACGGCCTCGACAGCAATGGCGTGTATGCACCTCCTATTTCCTGCCTTC
AGATCTCGTCCTCCCTGCCGGCTTGGCATTGACAATCATACAGTTACAACGGCACTTCC
GAGCTCCAGCTCGAGCGCATGAACGTCTACTTCAACGAGGTAAGTCAACAGC--
CACGCCG-----TTGGCCATCTG-CAGCACG--
GTTTGCTGCCGTCGCCGGGGCCTTGCTAACGCGTTATCGCCC-

AGGCCTCCGGCAACAAGTATGTCCCTCGCGCCGTCCTCGTCGATCTCGAGCCCGGTACC
ATGGACGCCGTC

>Diaporthe_sennae_CFCC_51636

GTCGGCCCATGCTGC-TTTCGCATCCTCTGCC------CCTGAGCCTGAGCCTA-
CCCCACCATCGCGACCG--
CACCCACGGTCGGGCCTCAAAAACATCACCAAACACCCTGGGAAAA-
GCACCCAGATGCCCTTGAAAAACGCGTCAGATTGCTAACGTGACG-
TTTTTCTCGACTACAGGTTCACCTTCAGACCGGCCAATGCGTAAGTTGCTCCTGTCA--
ACACCGACGGACCTTATCATCGCCACCCGTAGCTGACACGTTTCCCAGGGTAACCAAAT
CGGTGCTGCTTTCTGGTGC------------------GTCCACCACCGCGACGCTC-
GACGCGCGACAACACGACCTCGAAACATCGTTACTGACCTTTACTCTCAGGCAAACCA
TCTCTGGCGAGCACGGCCTCGACAGCAATGGCGTGTATGCACCTCCTATTTTCTGCCTTC
AGATCTCGTCCTCCCTGCCGGCTTGGCACTGACAATCACACAGTTACAACGGCACTTCC
GAGCTCCAGCTCGAGCGCATGAACGTCTACTTCAACGAGGTAAGTCAACAGC--
CACGTCG-----TTGACCATCTG-CAGCACG--
GTTTGCTGCCGTCGCTGGGGCCTTGCTAACGCGTTATCGCAC-
AGGCCTCCGGCAACAAGTATGTCCCTCGCGCCGTCCTCGTCGATCTCGAGCCCGGTACT
ATGGACGCCGTC

> Diaporthe_africana_STMA18294

NNNNNNNNNNNNNNNNNNNNNNNNNNNNNNNNNNNNNNNNNNNNNNNNNNN
NNNNNNNNNNNNNNNNNNNNNNNNNNNNNNNNNNNNNNNNNNNNNNNNNNN
NNNNNNNNNNNNNNNNNNNNNNNNNNNNNNNNNNNNNNNNNNNNNNNNNNN
NNNNNNNNNNNNNNNNNNNNNNNNNNNNNNNNNNNNNNNNNNNNNNNNNNN
NNNNNNNNNNNNNNNNNNNNNNNNNNNNNNNNNNNNNNNNNNNNNNNNNNN
NNNNNNNNNNNNNNNNNNNNNNNNNNNNNNCGGTGCTGCTTTCTGGTGCGTCCCA
GCTCCGGCTCAAAGTCCACCACCGCGACGCTC-
GACGCGCGACAATACGACCTCGAACCATCGTTACTGACATCGACTCTCAGGCAAACCA
TCTCTGGCGAGCACGGCCTCGACAGCAATGGCGTGTATGCACCTCCTATTCCCTACTCTC
ATATCTTGTCCTCCCTGCCGGCTTGGCACTGACAATCACACAGTTACAATGGCTCTTCCG
AGCTCCAGCTCGAGCGCATGAACGTCTACTTCAACGAGGTAAGTCAACAGC--
CACGTCG-----TTGACCATCTG-CAGCACG--
GTTTCCTGCCGTCGCCAAGGCCTTGCTAACGCGTTATCGTCC-
AGGCCTCCGGCAACAAGTATGTTCCTCGCGCCGTCCTCGTCGATCTCGAGCCCGGTACC
ATGGACGCCGTC

>Diaporthe_africana_CBS_150080

GTCGGCCCATGCTGC-TTTCGCATCCTCTGCC------CCTGAGCCTGAGCCTA-
CCCCACCATCGCGACCG--

CACCCACGGTCGGGCCTCAAAAACATCACCAAACACCCTGGGAAAA-
GCACCCAAATGTCCTTGAAAAACGCGTCAGATTGCTAACGTGACC-
TTTTTCTTGACTACAGGTTCACCTTCAGACCGGCCAATGCGTAAGTCGCTCCTGTCA--
ACACCGCCGGACCTTATCATCGCCACCCGTAGCTGACACGTTTCCCAGGGTAACCAAAT
CGGTGCTGCTTTCTGGTGCGTCCCAGCTCCGGCTCAAAGTCCACCACCGCGACGCTC-
GACGCGCGACAATACGACCTCGAACCATCGTTACTGACATCGACTCTCAGGCAAACCA
TCTCTGGCGAGCACGGCCTCGACAGCAATGGCGTGTATGCACCTCCTATTCCCTACTCTC
ATATCTTGTCCTCCCTGCCGGCTTGGCACTGACAATCACACAGTTACAATGGCTCTTCCG
AGCTCCAGCTCGAGCGCATGAACGTCTACTTCAACGAGGTAAGTCAACAGC--
CACGTCG-----TTGACCATCTG-CAGCACG--
GTTTCCTGCCGTCGCCAAGGCCTTGCTAACGCGTTATCGTCC-
AGGCCTCCGGCAACAAGTATGTTCCTCGCGCCGTCCTCGTCGATCTCGAGCCCGGTACC
ATGGACGCCGTC

>Diaporthe_melitensis_CBS_142551

NNNNNNNNNNNNNNNNNNNCAAGGGTCACTACA------CTGAGGGTAACATCCG-
TGAGATTGTAAGTATACCCCACCCTCGGTCGGGCCTCAAAAACATCACCAAACACCCT
GGGAAAA-GCACCCAGATGCCCTTGAAAAACGCGTCAGATTGCTAACGTGAAC-
TTTTTCTCGACTACAGGTTCACCTTCAGACCGGCCAATGCGTAAGTTGCTCCTGTCA--
ACACCGCCGGACCTTGTCATCGCCACCCGTAGCTGACACGTTTCCCAGGGTAACCAAAT
CGGTGCTGCTTTCTGGTGC------------------GTCCACCACCGCGACGCTC-
GACGCGCGACAATACGACCTCGAGCCATCGTTACTGACCTCGACTCTCAGGCAAACCA
TCTCTGGCGAGCACGGCCTCGACAGCAATGGCGTGTATGCACCTCCTATTCCCTACCTC
CAGATCTCGTCCTCCCTGCCGGCTTGGCACTGACAATCATACAGTTACAACGGCACTTC
CGAGCTCCAGCTCGAGCGCATGAACGTCTACTTCAACGAGGTAAGTCAACAGC--
CACGTCG-----TTGATCATCTG-CAGCACG---
TATCCTGCCGTCGCCGGGGCCTTGCTAACGCGTTATCGCCC-
AGGCCTCCGGCAACAAGTATGTCCCTCGCGCCGTCCTCGTCGATCTCGAGCCCAAGGGT
CACTACACTGNN

>Diaporthe_pescicola_MFLUCC_16_0105

NNNNNNNNNNNNNNNNNNNNNNNNNNNNNNNNNNNNNNNNNNNNNNNNNNNNNNN
NNNNNNNNNNNNNNNNNNNNNNNNNNNNNNNNNNNNNNNNNNNNNNNNNNNNNNN
NNNNNNNNNNNNNNNNNNNNNNNNNNNNNNNNNNNNNNNNNNNNNNNNNNNNNNN
NNNNNNNNNNNNNNNNNNNNNNNNNNNNNNNNNNNNNNNNNNNNNNNNNNNNNNN
NNNNNNNNNNNNNNNCTGTCA--
ACACCACCGGACCTTATCATCGCCACCTGTAGCTGACACGTTTCCCAGGGTAACCAAAT
CGGTGCTGCTTTCTGGTGCGTCCCAGCTCCAGCTCCAAGCCCACCACCGCGGCGCTC-
GACGCGCGAC-
ACATGACCTCGAGCCATCGTTACTGACCTCGACTCTCAGGCAAACCATCTCTGGCGAGC

ACGGCCTCGACAGCAATGGCGTGTATGCACCTCCTATTTCCTGCCTTCAGATCTCGTCCT
CCCTGCCGGCTTGGCACTGACAATCACACAGTTACAACGGCACTTCCGAGCTCCAGCTC
GAGCGCATGAACGTCTACTTCAACGAGGTAAGTCAACAGC--CACGTCG-----
TTGATCATCTG-CAGCACG--
ATTTTCTGCCGTCGCCGGGGCCTTGCTAACGCGTTATCGCCC-
AGGCCTCCGGCAACAAGTATGTCCCTCGCGCCGTCCTCGTCGATCTCGAGCCCGGTACC
ATGGACGCCGTC

>Diaporthe_taoicola_MFLUCC_16_0117

NNNNNNNNNNNNNNNNNNNNNNNNNNNNNNNNNNNNNNNNNNNNNN
NNNNNNNNNNNNNNNNNNNNNNNNNNNNNNNNNNNNNNNNNNNNNN
NNNNNNNNNNNNNNNNNNNNNNNNNNNNNNNNNNNNNNNNNNNNNN
NNNNNNNNNNNNNNNNNNNNNNNNNNNNNNNNNNNNNNNNNNNNNN
NNNNNNNNNNNNNNNNNCTGTCA--
ACACCGCCGGACCTTATCATCGCCACCTGTAGCTGACACGTTTCCCAGGGTAACCAAAT
CGGTGCTGCTTTCTGGTGC-------------------GTCCACCACCGCGACGCTC-
GACGCGCGACTATACAATCTCAGACCATCGTTACTGACCTCGACTCTCAGGCAAACCAT
CTCTGGCGAGCACGGCCTCGACAGCAATGGCGTGTATGCACCTCCTATTCCCTACCTTC
AGATCTCGTCCTCCCTGCCGGCTTGGCACTGACAATCATACAGTTACAACGGCACTTCC
GAGCTCCAGCTCGAGCGCATGAACGTCTACTTCAACGAGGTAAGTCAACAGA--
CACGTCG-----TTGACCATCTG-CAGCACG--
GTTTGCTGCCGTCGCTGGGGCCTTGCTAACGCGTTCTCGCCC-
AGGCCTCCGGCAACAAGTATGTCCCTCGCGCCGTCCTCGTCGATCTCGAGCCCGGTACT
ATGGACGCCGTC

>Diaporthe_pterocarpicola_MFLUCC_10_0580a

NNNNNNNNNNNNNNNNNNNNNNNNNNNNNNNNNNNNNNNNNNNNNN
NNNNNNNNNNNNNNNNNNNNNNNNNNNNNNNNNNNNNNNNNNNNNN
NNNNNNNNNNNNNNNNNNNNNNNNNNNNNNNNNNNNNNNNNNNNNN
NNNNNNNNNNNNNNNNNNNNNNNNNNNNNNNNNNNNNNNNNNNNNN
NNNNNNNNNNNNNNNNNNNNNNNNNNNNNNNNNNNNNNNNNNNNNN
NNNNNNNNNNNNNNNNNNNNNNNNNNNNNNNNNNNNNNNNNNNNNN
NNNNNNNNNNNNNNNNNNNNNNNNNNNNNNNNNNNNNNNNNNNNNN
NNACCTCGAACCATCGTTACTGACCTCGACTCTCAGGCAAACCATCTCTGGCGAGCAC
GGCCTCGACAGCAATGGCGTGTATGCACCTCCTATTTCCTACCTTCAGATCTCGTCCTCC
CTGCCGGCTTGGCACTGACAATCACACAGTTACAACGGCACTTCCGAGCTCCAGCTCGA
GCGCATGAACGTCTACTTCAACGAGGTAAGTCAACAGC--CACGTCG-----
TTGGCCATCTG-CAGCACG--
GTTTGCTGCCGTCGCTGGGGCCTTGCTAACGCGTTATCGCAC-

AGGCCTCCGGCAACAAGTATGTCCCTCGCGCCGTCCTCGTCGATCTCGAGCCCGGTACC
ATGGACGCCGTC

>Diaporthe_pandanicola_MFLUCC_17_0607

NNNNNNNNNNNNNNNNNNNNNNNNNNNNNNNNNNNNNNNNNNNNNNNNNNN
NNNNNNNNNNNNNNNNNNNNNNNNNNNNNNNNNNNNNNNNNNNNNNNNNNN
NNNNNNNNNNNNNNNNNNNNNNNNNNNNNNNNNNNNNNNNNNNNNNNNNNN
NNNNNNNNNNNNNNNNNNNNNNNNNNNNNNNNNNNNNNNNNNNNNNNNNNN
NNNNNNNNNNNNNNNNNNNNNNNNNNNNNNNNNNNNNNNNNNNNNNNNNNN
NNNNNNNNNNNNNNNNNNNNNNNNNNNNNNNNNNNNNNNNNNNNNNNNNNN
NNNNNNNNNNNNNNNNNNNNNNNNNNNNNNNNNNNNNNNNNNNNNNNNNNN
NNACCTCGTACCATCGTTACTGACCTCGACTCTCAGGCAAACCATCTCTGGCGAGCACG
GCCTCGACAGCAATGGCGTGTATGCACCTCCTATTCCCTACCTCCAGATCTCGTCCTCCC
TGCCGGCTTGGCACTGACAATCATACAGTTACAACGGCACTTCCGAGCTCCAGCTCGAG
CGCATGAACGTCTACTTCAACGAGGTAAGTCAACAGC--CACGTCG-----TTGGCCATCTG-
CAGCACG--GTTTGCTGCCGTCGCTGGGGCCTTGCTAACGCGTTATCGCAC-
AGGCCTCCGGCAACAAGTATGTCCCTCGCGCCGTCCTCGTCGATCTCGAGCCTGGTACC
ATGGACGCCGTC

>Diaporthe_podocarpi-macrophylli_CGMCC3_18281

NNNNNNNNNNNNNNNNNNNNNNNNNNNNNNNNNNNNNNNNNNNNNNNNNNN
NNNNNNNNNNNNNNNNNNNNNNNNNNNNNNNNNNNNNNNNNNNNNNNNNNN
NNNNNNNNNNNNNNNNNNNNNNNNNNNNNNNNNNNNNNNNNNNNNNNNNNN
NNNNNNNNNNNNNNNNNNNNNNNNNNNNNNNNNNNNNNNNNNNNNNNNNNN
NNNNNNNNNNNNNNNNNNNNNNNNNNNNNNNNNNNNNNNNNNNNNNNNNNN
NNNNNNNNNNNNNNNNNNNNNNNNNNNNNNNGGTGCTGCTTTCTGGTGC------------
-------GTCCACCACCGCGACGCTC-
GACGCGCGACAATACGACCTCGAGCCATCGTTACTGACCTCGACTCTCAGGCAAACCA
TCTCTGGCGAGCACGGCCTCGACAGCAATGGCGTGTATGCACCTCCTATTCCCTACCTTC
AGATCTCGTCCTCCCTGCCGGCTTGGCACTGACAATCATACAGTTACAACGGCACTTCC
GAGCTCCAGCTCGAGCGCATGAACGTCTACTTCAACGAGGTAAGTCAACAGC--
CACGTCG-----TTGATCATCTG-CAGCACG--
ATTTTCTGCCGTCGCCGGGGCCTTGCTAACGCGTTATCGCCC-
AGGCCTCCGGCAACAAGTATGTCCCTCGCGCCGTCCTCGTCGATCTCGAGCCCGGTACC
ATGGACGCCGTC

>Diaporthe_spinosa_PSCG_383

NNNNNNNNNNNNNNNNNNNNNNNNNNNNNNNNNNNNNNNNNNNNNNNNNNN
NNNNNNNNNNNNNNNNNNNNNNNNNNNNNNNNNNNNNNNNNNNNNNNNNNN
NNNNNNNNNNNNNNNNNNNNNNNNNNNNNNNNNNNNNNNNNNNNNNNNNNN

NNNNNNNNNNNNNNNNNNNNNNNNNNNNNNNNNNNNNNNNNNNNNNNNNNNNNN
NNNNNNNNNNNNNNNNNNNNNNNNNNNNNNNNNNNNNNNNNNNNNNNNNNNNNN
NNNNNNNNNNNNNNNNNNNNNNNNNNNNNNNNNNNNNNNNNNNNNNNNNNGA-----
--------------GTGGGTTCACGCGACGCTC-GACGCGCGAC-
ATATGACCTCGAGCCATCGTTACTGACCTCGACTCTCAGGCAAACCATCTCTGGCGAGC
ACGGCCTCGACAGCAATGGCGTGTATGCACCTCCTATTCCCTACCTTCAGATCTCGTCCT
CCCTGCCGGCTTGGCACTGACAATCATACAGTTACAACGGCACTTCCGAGCTCCAGCTC
GAGCGCATGAACGTCTACTTCAACGAGGTAAGTAAACTGC--CACGTCG-----
TTGGCCATCTG-CAGCACG--GTTTGCTGCCGTCGCC-
AGGCTTTGCTAACGCGTTATCGCAC-
AGGCCTCCGGCAACAAGTATGTCCCTCGCGCCGTCCTCGTCGATCTCGAGCCCGGTACT
ATGGACGCCGTC

>Diaporthe_tectonigena_MFLUCC_12_0767

NNNNNNNNNNNNNNNNNNNNNNNNNNNNNNNNNNNNNNNNNNNNNNNNNNNNNN
NNNNNNNNNNNNNNNNNNNNNNNNNNNNNNNNNNNNNNNNNNNNNNNNNNNNNN
NNNNNNNNNNNNNNNNNNNNNNNNNNNNNNNNNNNNNNNNNNNNNNNNNNNNNN
NNNNNNNNNNNNNNNNNNNNNNNNNNNNNNNNNNNNNNNNNNNNNNNNNNNNNN
NNNNNNNNNNNNNNNNNNNNNNNNNNNNNNNNNNNNNNNNNNNNNNNNNNNNNN
NNNNNNNNNNNNNNNNNNNNNNNNNNNNNNNNNNNNNNNNNNNNNNNNNNNNNN
NNNNNNNNNNNNNNNNNNNNNNNNNNNNCGACGCTC-
GACGCGCGACAATACGACTTCGATCCATCGTCACTGACCTCGACTCTCAGGCAAACCAT
CTCTGGCGAGCACGGCCTCGACAGCAATGGCGTGTATGCACCTCCTATTCCCTACCTCC
AGATCTCGTCCTCCCTGCCGGCTTGGCACTGACAATCATACAGTTACAACGGCACTTCC
GAGCTCCAGCTCGAGCGCATGAACGTCTACTTCAACGAGGCAAGTCAACAGC--
CACGTCG-----TTGACCATCTG-CAGCACG--
GTTTCCTGCCGTCGCCAAGGCTTTGCTAACGCGTTATCGCCC-
AGGCCTCCGGCAACAAGTATGTTCCTCGCGCCGTCCTTGTCGATCTCGAGCCCGGTACC
ATGGACGCCGTC

>Diaporthe_viniferae_JZB320071

NNNNNNNNNNNNNNNNNNNNNNNNNNNNNNNNNNNNNNNNNNNNNNNNNNNNNN
NNNNNNNNNNNNNNNNNNNNNNNNNNNNNNNNNNNNNNNNNNNNNNNNNNNNNN
NNNNNNNNNNNNNNNNNNNNNNNNNNNNNNNNNNNNNNNNNNNNNNNNNNNNNN
NNNNNNNNNNNNNNNNNNNNNNNNNNNNNNNNNNNNNNNNNNNNNNNNNNNNNN
NNNNNNNNNNNNNNNNNNNNNNNNNNNNNNNNNNNNNNNNNNNNNNNNNNNNNN
NNNNNNNNNNNNNNNNNNNNNNNNNNNNNNNGGTGCTGCTTTCTGGTGCGTTCCA
GCTCGAGCTCCAAG--GTTACACGCGACGCTC-GACGCGCGAC-
ATATGACCTCGAGCCATCGTTACTGACCTCGACTCTCAGGCAAACCATCTCTGGCGAGC
ACGGCCTCGACAGCAATGGCGTGTATGCACCTCCTATTCCCTACCTCTAGATCTCGTCCT

CCCTGCCGGCTTGGCACTGACAATCATACAGTTACAACGGCACTTCCGAGCTCCAGCTC
GAGCGCATGAACGTCTACTTCAACGAGGTAAGTCAACAGC--CACGTCG-----
TTGACCATCTG-CAGCACG--
GTTTGCTGCCGTCGCTGGGGCCTTGCTAACGCGTTATCGCCC-
AGGCCTCCGGCAACAAGTATGTCCCTCGCGCCGTCCTCGTCGATCTCGAGCCCGGTACC
ATGGACGCCGTC

>Diaporthe_xishuangbanica_CGMCC_3_18282

NNNNNNNNNNNNNNNNNNNNNNNNNNNNNNNNNNNNNNNNNNNNNNNNNNNNNNNNNNNN
NNNNNNNNNNNNNNNNNNNNNNNNNNNNNNNNNNNNNNNNNNNNNNNNNNNNNNNNNNNN
NNNNNNNNNNNNNNNNNNNNNNNNNNNNNNNNNNNNNNNNNNNNNNNNNNNNNNNNNNNN
NNNNNNNNNNNNNNNNNNNNNNNNNNNNNNNNNNNNNNNNNNNNNNNNNNNNNNNNNNNN
NNNNNNNNNNNNNNNNNNNNNNNNNNNNNNNNNNNNNNNNNNNNNNNNNNNNNNNNNNNN
NNNNNNNNNNNNNNNNNNNNNNNNNNNNNNNNNNNGGTGCTGCTTTCTGGTGCGTCCT--
----------AAGTCCACCACCGC--------
GACGCGCGACAATACGACCTCGAATCATCGTTACTGACCTCGACTCTCAGGCAAACCAT
CTCTGGCGAGCACGGCCTCGACAGCAATGGCGTGTATGCACCTCCTATTCCCTACCTCC
AGATCTCGTCCTCCCTGCCGGCTTGGCACTGACAATCATGCAGTTACAACGGCACTTCC
GAGCTTCAGCTCGAGCGCATGAACGTCTACTTCAACGAGGTAAGTCAACAGC--
CACGTCG-----TTGATCATCTG-CAGCACG--
ATTTGCTGCCGTCGCCGGGGCCTTGCTAACGCGTTATCGCCC-
AGGCCTCCGGCAACAAGTATGTCCCTCGCGCCGTCCTCGTCGATCTCGAGCCCGGTACC
ATGGACGCCGTC

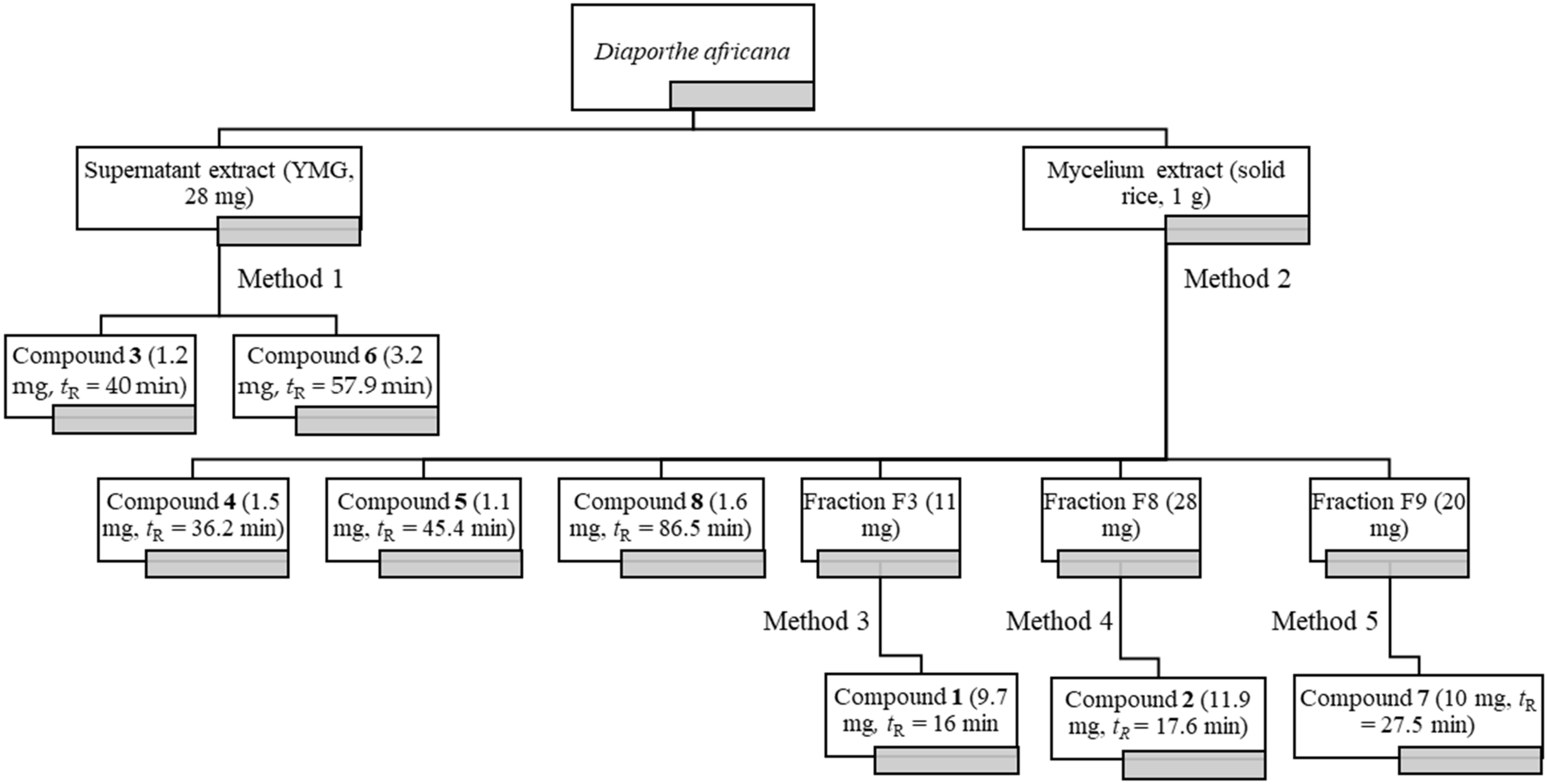
Diaporthe africana
Supernatant extract (YMG, 28 mg)
Mycelium extract (solid rice, 1 g)
Method 1
Method 2
Compound 3 (1.2 mg, t_R = 40 min)
Compound 6 (3.2 mg, t_R = 57.9 min)
Compound 4 (1.5 mg, t_R = 36.2 min)
Compound 5 (1.1 mg, t_R = 45.4 min)
Compound 8 (1.6 mg, t_R = 86.5 min)
Fraction F3 (11 mg)
Fraction F8 (28 mg)
Fraction F9 (20 mg)
Method 3
Method 4
Method 5
Compound 1 (9.7 mg, t_R = 16 min
Compound 2 (11.9 mg, t_R = 17.6 min)
Compound 7 (10 mg, t_R = 27.5 min)

Methods	Columns	Flow rate (mL/min)	Gradient elution<br><br>(B= MeCN with 0.1% Formic acid, A= H_2O with 0.1 % Formic acid)
1	VP 250/21 Nucleodur C18 Htec 10 µm column (Machery-Nagel, Düren, Germany)	15	20−45% solvent B in 20 min, 45−65% B in 30 min, 65−100% B in 10 min and thereafter isocratic conditions at 100% B for 10 min.
2	VP 250/40 Nucleodur C18 Htec 10 µm column, (Machery-Nagel, Düren, Germany)	30	5−80% solvent B in 70 min, 80−100% B in 20 min, and finally isocratic conditions at 100% B for 5 min
3	XBridge™ Trifunctional C18, 250 × 19mm, 135 Å, 5 µm column (Waters, Eschborn, Germany)	15	5–10% solvent B in 10 min, then isocratic condition at 50% B for 30 min and finally 50–100% B in 10 min
4	XBridge™ Trifunctional C18, 250 × 19mm, 135 Å, 5 µm column (Waters, Eschborn, Germany)	15	5-60% solvent B in 10 min, then isocratic condition at 60% B for 30 min and finally 60 – 100% B in 5 min
5	XBridge™ Trifunctional C18, 250 × 19mm, 135 Å, 5 µm column (Waters, Eschborn, Germany)	15	5−65% solvent B in 10 min, followed by isocratic condition at 65% B for 30 min and finally 65 – 100% B in 10 min

Figure S76: Flow chart of the purification procedure
